# Identification of Nogo-B as a potential therapeutic target of osteosarcoma via stereochemically selective covalent probes

**DOI:** 10.1038/s41419-025-07765-z

**Published:** 2025-07-19

**Authors:** Jian Xue, Meng Li, Li Kang, Meiting Wang, Jiabin Yin, Donghui Sun, Yaqi Deng, Qinghua Wei, Jiemin Wong, Tong Zhu, Shunying Liu

**Affiliations:** 1 Shanghai Engineering Research Center of Molecular Therapeutics and New Drug Development, School of Chemistry and Molecular Engineering, East China Normal University, Shanghai, 200062 China; 2 Shanghai Key Laboratory of Regulatory Biology, Fengxian District Central Hospital-ECNU Joint Center of Translational Medicine, Institute of Biomedical Sciences and School of Life Sciences, East China Normal University, Shanghai, 200241 China

**Keywords:** Target identification, Target validation

## Abstract

Osteosarcoma (OS) has been defined as one of the most intricate and formidable malignant bone tumors, and there has been no significant improvement in targeted therapies for OS over the past 50 years. Therefore, it is crucial to identify new potential drug targets for OS. Here, we have developed a label-free activity-based protein profiling (ABPP) using a stereochemically selective probe from an in-house patrimonial library of covalent small molecule compounds to identify an anti-OS target. Phenotypic screening resulted in the discovery of a selective inhibitor (*S*,*R*)-**4v** that potently suppresses the proliferation of OS 143B cells with an IC_50_ value of 0.28 µM. Subsequent label-free ABPP studies identified neurite outgrowth inhibitor B (Nogo-B) as the primary cellular target for (*S*,*R*)-**4v** via a rapid relatively quantitative analysis using its inactive isomer as control. This finding was validated by interaction assays including pull-down, cellular thermal shift assay (CETSA), molecular docking and functional studies. Mechanistic investigations revealed that the apoptotic effect induced by (*S*,*R*)-**4v** was mediated through Nogo-B inhibition of the PI3K/AKT-dependent NF-κB pathway. Altogether, this study presents a novel strategy that couples anti-OS compound screening with target identification and successfully identifies Nogo-B as a potential candidate for targeted OS therapy.

## Introduction

Osteosarcoma is the most common malignant bone tumor in children and adolescents [1, 2]. Recently, the five-year survival rate of the non-metastatic patients has reached to approximate 70% because of the improvement in early diagnosis and surgical treatment [3, 4]. However, currently the patients with metastatic OS have a five-year survival rate < 20% and various treatments including immunotherapy remain ineffective for them [5, 6]. Hence, the identification and characterization of new therapeutic targets is crucial to improve the efficacy and survival rate of metastatic OS patients.

With the progress of various emerging technologies and the continuous efforts of scientists, several key components of the PI3K/Akt [7], Wnt [8], CDK4 [9], STAT3 [10] pathways have been reported as the candidate targets for the treatment of OS. Even though some of these candidates are targetable by available agents, none of the compounds has been approved for clinical treatment of OS. Different immunotherapeutic strategies have been tested in OS but clinical benefits were disappointed [11]. In addition, genomic profiling has been used to find new targets for OS and several genes have been identified as possible drivers of OS, including TP53 [12], RB1 [13], CDKN2A [14] and MYC[15]. However, their low incidence and the extensive chromatin fragment fragmentations and intensive mutations make it difficult to confirm the alterations [16]. Therefore, it is crucial to develop new approaches for identifying new potential drug targets for OS.

In recent years, driven by the groundbreaking research conducted by Cravatt’s and other groups [17–23], activity-based protein profiling (ABPP) has emerged as a widely adopted technique for identifying new targets. ABPP makes use of functionally active small molecules to enrich for candidate protein(s) with a high affinity for these molecules, followed by their identification through mass spectrometry, enabling direct identification and measurement of protein function within complex systems. For a target identification of OS by ABPP, this strategy requires novel probe molecules with selective biological activity, high affinity as well as chemically modifiable site. Covalent inhibitors bind to amino acid residues (-NH_2_, -SH, -OH) of target proteins through covalent bonds via mildly reactive electrophilic groups (warheads) [24]. A covalent probe can provide a high affinity with the target protein for ABPP without additional label procedures, which will greatly improve the efficacy of the protein enrichment and purification.

**Fig. 1:**
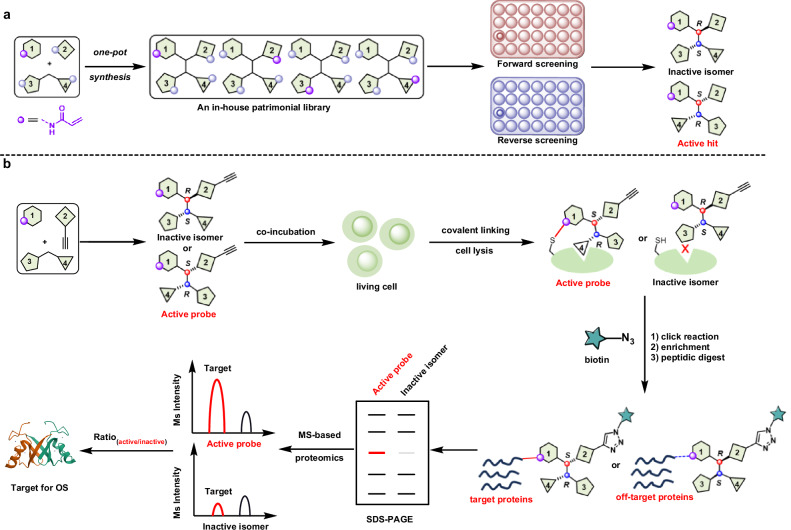
The designed strategy for searching targets of OS. **a** Probe search based on one-pot synthesis of stereochemical diverse covalent molecular library. **b** Stereochemical diverse covalent probe-based ABPP for target identification.

The covalent drug molecules currently available in the market are mainly designed for ATP-competitive binding pockets of kinases and typically possess planar molecular structures [25]. These planar covalent molecules failed to yield any significant clinical outcomes in OS, suggesting that potential target proteins in OS might have different active binding pockets compared to kinases. Hence, we envision that covalent molecules with stereochemical architectures might offer some opportunities for the discovery of novel target proteins which possess some a more open or tridimensional (3D) active binding package. Based on the one-pot multi-component reactions [26–29], covalent warheads can be easily integrated into molecules with quaternary carbon centers by varying the synthetic blocks, thereby it will provide a rapid way for constructing complex covalent molecular libraries with stereo-structural diversity (Fig. 1a), which may subsequently provide hit compound with structural novelty for OS through phenotypic screening. Further, based on the stereochemical selectivity of the resulting hit to its target proteins, the off-target effect may be overcome by using its unactive stereoisomer as a control through a label-free relatively quantitative ABPP strategy to conveniently identify the promising anti-OS targets (Fig. 1b).

## Results

### Library synthesis and phenotypic screening reveal (*S*,*R*)-4v as a selective inhibitor for OS in 143B cell

To obtain the probe molecules for OS target search, a 37-member library containing acrylamide warhead was efficiently synthesized by our previously developed one-pot synthetic methods [26–29] at room temperature in air (Supplementary Fig. S1). Briefly, our previously established one-pot reaction method involving diazo compounds (**1**), amides/alcohols (**2**), and imines (**3**) was employed to generate amino acid derivatives containing acrylamide warheads as the desired products. Gratifyingly, it was found that the active carbene intermediate, which readily undergoes an electrophilic addition to electrophiles, had a good compatibility with the electrophilic warheads to deliver the desired molecular scaffolds containing quaternary carbon center and acrylamide warhead. Compared with the established covalent drug molecules with a planar molecular structure [30], the designed covalent molecules possess a spherical (3D) molecular structure may show a remarkable selectivity towards the potential target due to its stereochemical structure.

**Fig. 2:**
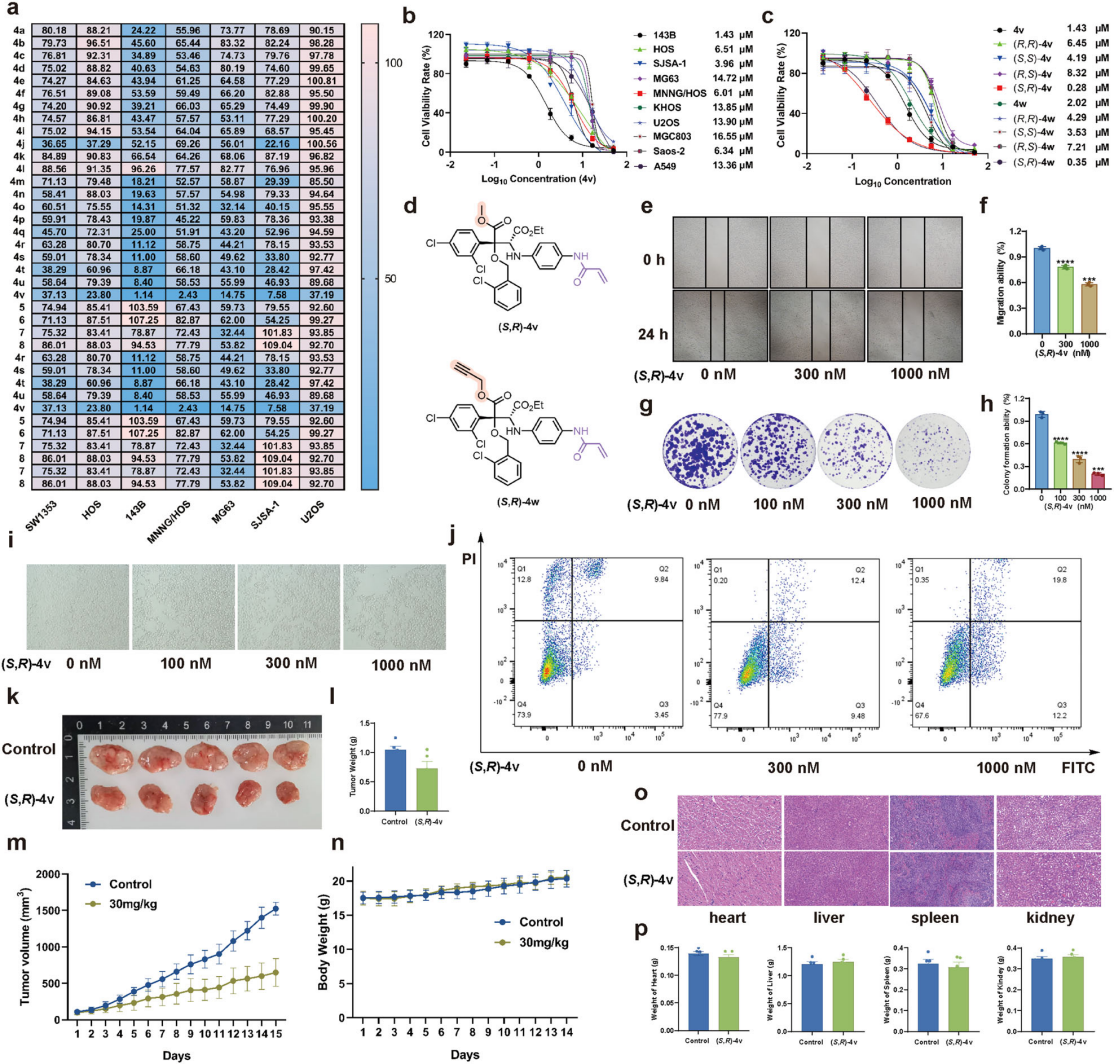
(*S*, *R*)-4v significantly inhibits the proliferation and migration of 143B cells in vitro and significantly suppressed the growth of xenograft tumors in vivo. **a** Forward screening. CCK-8 assay showed the cell viability rate (%) of 37 compounds in 7 OS cells at 20 µΜ. **b** Reverse screening. The IC_50_ values of against 10 cancer cells of the compound **4v**. **c** The IC_50_ values of against 143B cells of the four corresponding isomers of compounds **4v** and **4w**. **d** The structure of compounds (*S*,*R*)-**4v** and (*S*,*R*)-**4w**. **e** Wound healing assay to determine the effects of (*S*,*R*)-**4v** on 143B cells migration. **f** Quantification of data in wound healing assay. **g** The effect of (*S*,*R*)-**4v** on colony formation was monitored. **h** Quantification of data in colony formation assay. **i** Cell morphology was observed to detect apoptosis at different concentrations of (*S*,*R*)-**4v**. **j** 143B cells were treated with DMSO or (*S*,*R*)-**4v** for 24 h, the apoptosis rate was analyzed by flow cytometry. **k** Nude mice bearing 143B-derived xenografts were orally administered (*S*,*R*)-**4v** (30 mg/kg) or vehicle once every day (n = 5 mice per group). Representative images of the tumors showed that (*S*,*R*)-**4v** significantly suppressed the growth of xenograft tumors. **l** Tumor weight changes following the treatment of compound (*S*,*R*)-**4v** (30 mg/kg) or vehicle. **m** Tumor volume changes following the treatment of compound (*S*,*R*)-**4v** (30 mg/kg) or vehicle once every day. **n** Average body weights for compound (*S*,*R*)-**4v** or vehicle-treated mouse groups during the treatment. **o** Representative images of HE staining of heart, liver, kidney and spleen. In the anti-tumor experiments, hearts, livers, kidneys, and spleens of the vehicle-treated and compound (*S*,*R*)-**4v**-treated mice were removed, weighted, sliced, and analyzed by HE staining. **p** The corresponding weight of the organs. The data are reported as the mean ± SD. All data are representative of three independent experiments. ***P < 0.005; ****P < 0.001.

With this compound library in hand, we initially conducted a cell counting kit 8 (CCK-8) assay to randomly screen the inhibitory activity of all products on various OS cell lines including 143B, Saos-2, HOS, SJSA-1, MG63, MNNG/HOS and U2OS (Fig. 2a). Encouragingly, it was found that most of the compounds exhibited a significantly and specifically inhibitory activity against 143B cell line over other tested cells. Notably, product **4** demonstrated improved activity by introducing the warhead into the benzene ring compared with product **5** that directly assembled the acrylamide warhead group on the quaternary carbon center. Among all products **4**, compound **4v** displayed a superior activity and selectivity. Based on these inhibition rates obtained from forward screening results, we subsequently evaluated the selective antiproliferative activity of compound **4v** on various cell lines as a reverse screening (Fig. 2b). The results demonstrate that compound **4v** exhibited the most potent antiproliferative activity against 143B cells with an IC_50_ value of 1.43 µM over other 9 cell lines including MNNG/HOS (IC_50_ = 6.01 µM), Saos-2 (IC_50_ = 6.34 µM), U2OS (IC_50_ = 13.90 µM), KHOS (IC_50_ = 13.85 µM), MG63 (IC_50_ = 14.72 µM), HOS (IC_50_ = 6.51 µM), A549 (IC_50_ = 13.36 µM) and MGC803 (IC_50_ = 16.55 µM). Compound **4v** also displayed a similar inhibitory activity on SJSA-1 cells at a concentration of approximately 3.6 µM. These results further illustrate the selectivity of **4v** for OS treatment. Based on the preliminary SARs study mentioned above, compound **4v** was selected as the hit molecule for the subsequent ABPP procedure to identify novel therapeutic targets for OS.

To identify the isomer as control for the relative quantitative analysis of LC-MS in the ABPP procedure, the inhibitory activity of the four isomers of **4v** was respectively checked on 143B cells. The four corresponding isomers of **4v** were isolated and obtained by chiral high performance liquid chromatography (Supplementary Fig. S2). The absolute configuration of the four isomers was determined by the experimental and theoretical circular dichroism analysis (Supplementary Fig. S3). The results of the inhibitory activity of four isomers, (*S*,*R*)-**4v**, (*R*,*S*)-**4v**, (*S*,*S*)-**4v** and (*R*,*R*)-**4v**, are summarized in Fig. 2c. (*S*,*R*)-**4v** exhibits the most potent inhibitory activity (IC_50_ = 0.28 µM) in 143B cells compared to the other three isomers, including (*R*,*S*)-**4v** (IC_50_ = 8.32 µM), (*R*,*R*)-**4v** (IC_50_ = 6.45 µM) and (*S*,*S*)-**4v** (IC_50_ = 4.19 µM). This observation further highlights the significant contribution of the stereochemical center to the selective bioactivity of the covalent probe. The assembly of the alkynyl group (used as a handle for the next coupling with biotin tag via click reaction [31]) to **4v** led to the intermediate **4w** (Fig. 2d), which is used as probe precursor for the subsequent target enrichment by ABPP. The introduction of the handle in **4w** had no obvious effect on the inhibitory activity over 143B cells (Fig. 2c). All four isomers of intermediate **4w** (Fig. 2c) gave a similar inhibitory activity as **4v** on 143B cells (IC_50_ = 0.35, 7.21, 4.39 and 3.53 µM for (*S*,*R*)-**4w**, (*R*,*S*)-**4w**, (*R*,*R*)-**4w** and (*S*,*S*)-**4w**, respectively). These results illustrate that the designed covalent hit compound is a promising probe for ABPP.

We further investigated the impact of compound (*S*,*R*)-**4v** on 143B cells through wound healing [32], morphological observations [33] and colony formation experiments [21]. The wound healing reveals that as the concentration of (*S*,*R*)-**4v** increased, a significant decrease in the percentage of intercellular wound healing distance was observed compared to the control group (Fig. 2e, f). These findings demonstrate that compound (*S*,*R*)-**4v** effectively inhibits the migration of OS 143B cells. The colony formation experiment further showed a significant decrease in the colony growth of 143B cells with increasing concentrations of compound (*S*,*R*)-**4v** (Fig. 2g, h). These findings established that (*S*,*R*)-**4v** exerts an inhibitory effect on 143B cell proliferation. In addition, the cell morphology was examined after 143B cells were treated with varying concentrations of (*S*,*R*)-**4v** for 24 h. In the control group, the cells exhibited well-adhered characteristics to the substrate, normal shape, regular arrangement, and high cell density (Fig. 2i). However, in the experimental group, abnormal cell morphology was observed with cellular shrinkage and disordered arrangement. Furthermore, there was a significant decrease in both the number and density of cells as the concentration of (*S*,*R*)-**4v** increased. Meanwhile, cell apoptosis was examined in cells treated with (*S*,*R*)-**4v**, and it was found (*S*,*R*)-**4v** induced notable apoptosis of 143B cells compared with that of the control group (Fig. 2j). These results demonstrated that the designed covalent hit compound (*S*,*R*)-**4v** is a suitable probe for the OS target identification by ABPP.

To further illustrate the alterations of OS by treated with (*S*,*R*)-**4v**, we examined the therapeutic efficacy of (*S*,*R*)-**4v** in vivo. We established a 143B xenograft model in 2-month-old BALB/c nude mice. Once the tumor volume reached approximately 50 mm^3^, we divided the mice into two groups and treated them with either a vehicle or compound (*S*,*R*)-**4v** via intraperitoneal administration at dosages of 30 mg/kg once every day. The results showed that treatment with (*S*,*R*)-**4v** significantly suppressed tumor growth (Fig. 2k–m). During the treatment period, the body weight of the mice was monitored, and no marked difference among the groups was observed (Fig. 2n). These findings underscore the potent in vivo antitumor effects of compound (*S*,*R*)-**4v**. In addition, we conducted Hematoxylin and Eosin (HE) staining on select organs collected at the conclusion of the animal studies. The results reveal no discernible morphological changes in the heart, liver, spleen, or kidney tissues of the compound (*S*,*R*)-**4v**-treated compared to the control (Fig. 2o). Besides, compound (*S*,*R*)-**4v** did not induce significant alterations in the weight of heart, liver, spleen and kidney (Fig. 2p). This further supports the safety and efficacy of compound (*S*,*R*)-**4v** in vivo. Collectively, these results indicate that (*S*,*R*)-**4v** suppresses OS progression in vitro and in vivo without obvious toxicity. Thus, (*S*,*R*)-**4w** was used as the tool molecule for the subsequent ABPP procedure and target identification. The less active enantiomer (*R*,*S*)-**4w** was selected as the control to perform the relative quantitative analysis of LC-MS/MS.

### Identification of a ~ 50 kDa protein as (*S*,*R*)-4w target by gel-based ABPP profiling

**Fig. 3:**
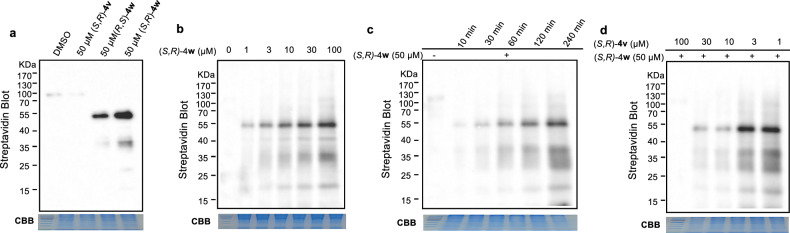
Identification of a ~ 50 kDa protein as (*S*,*R*)-4w target by gel-based ABPP profiling. **a** In situ labeling profiles of 143B cells were incubated with DMSO, 50 μM (*S*,*R*)-**4v**, (*S*,*R*)-**4w** and (*R*,*S*)-**4w**, respectively. **b** Concentration-dependent labeling profiles of (*S*,*R*)-**4w** in 143B cells. **c** Time-dependent labeling profiles of (*S*,*R*)-**4w** in 143B cells. **d** Competitive labeling profiles of (*S*,*R*)-**4w** in the presence or absence of (*S*,*R*)-**4v** in 143B cells.

In light of these aforementioned results, we initially investigated whether (*S*,*R*)-**4w** and (*R*,*S*)-**4w** can enter cells and efficiently label proteins in living cells. 143B cells were treated with DMSO, 50 μM of (*S*,*R*)-**4v**, (*S*,*R*)-**4w** and (*R*,*S*)-**4w** for a duration of 2 h. Subsequently, the cells were lysed using sonication and separated through centrifugation at 13,000 rpm. The labeled proteins were then subjected to a click reaction with biotin-N_3_, and affinity purification was performed using streptavidin beads which were subsequently separated by SDS-PAGE followed by detected via streptavidin blotting [34]. It is worth noting that only those proteins covalently bound by the probe **4w** can be successfully labeled and detected using streptavidin blotting method. As anticipated, after treatment with DMSO and (*S*,*R*)-**4v**, no protein labeling were observed due to the absence of covalent warheads or handles for the biotin enrichment process. To our delight, a selective labeling effect was observed on the ~50 kDa protein upon treatment with either (*S*,*R*)-**4w** or (*R*,*S*)-**4w** as shown in Fig. 3a.

To further assess the reactivity of the probes, various concentrations of (*S*,*R*)-**4w** was utilized to treat 143B living cells in labeling experiments. As shown in Fig. 3b, the labeling intensity of ~50 kDa probe-labeled protein increased proportionally with the increasing concentration of (*S*,*R*)-**4w**, indicating a dose-dependent relationship between the ~50 kDa probe-labeled protein and (*S*,*R*)-**4w**. An intense labeling of this band was observed at the concentration as low as 1 μM, suggesting a high-affinity binding between the ~50 kDa probe-labeled protein and (*S*,*R*)-**4w**. These results also demonstrated a unique selectivity of (*S*,*R*)-**4w** for the ~50 kDa protein, because even at 100 μM, the ~50 kDa protein remained as the unequivocal major target.

To examine the labeling efficiency, we incubated 143B cells with (*S*,*R*)-**4w** for various times (Fig. 3c). Remarkably, the 50 kDa-labeled protein was observed within 10 min. Furthermore, the labeling intensity of the ∼50 kDa increased over time. To assess the interaction specificity between probes and the ~50 kDa probe-labeled protein, we performed competitive experiments by treating 143B cells with varying concentrations of (*S*,*R*)-**4v** for 2 h prior to adding 50 μM (*S*,*R*)-**4w** (Fig. 3d). The labeling of ~50 kDa protein exhibited a concentration-dependent decrease upon preincubation of increasing levels of (*S*,*R*)-**4v**, and preincubation with 100 μM (*S*,*R*)-**4v** resulted in complete loss of labeling signal. These findings unequivocally demonstrate that the ∼50 kDa protein was specifically targeted by the probe rather than a product of non-specific labeling.

### Identification of the ~ 50 kDa target protein as Nogo-B

**Fig. 4:**
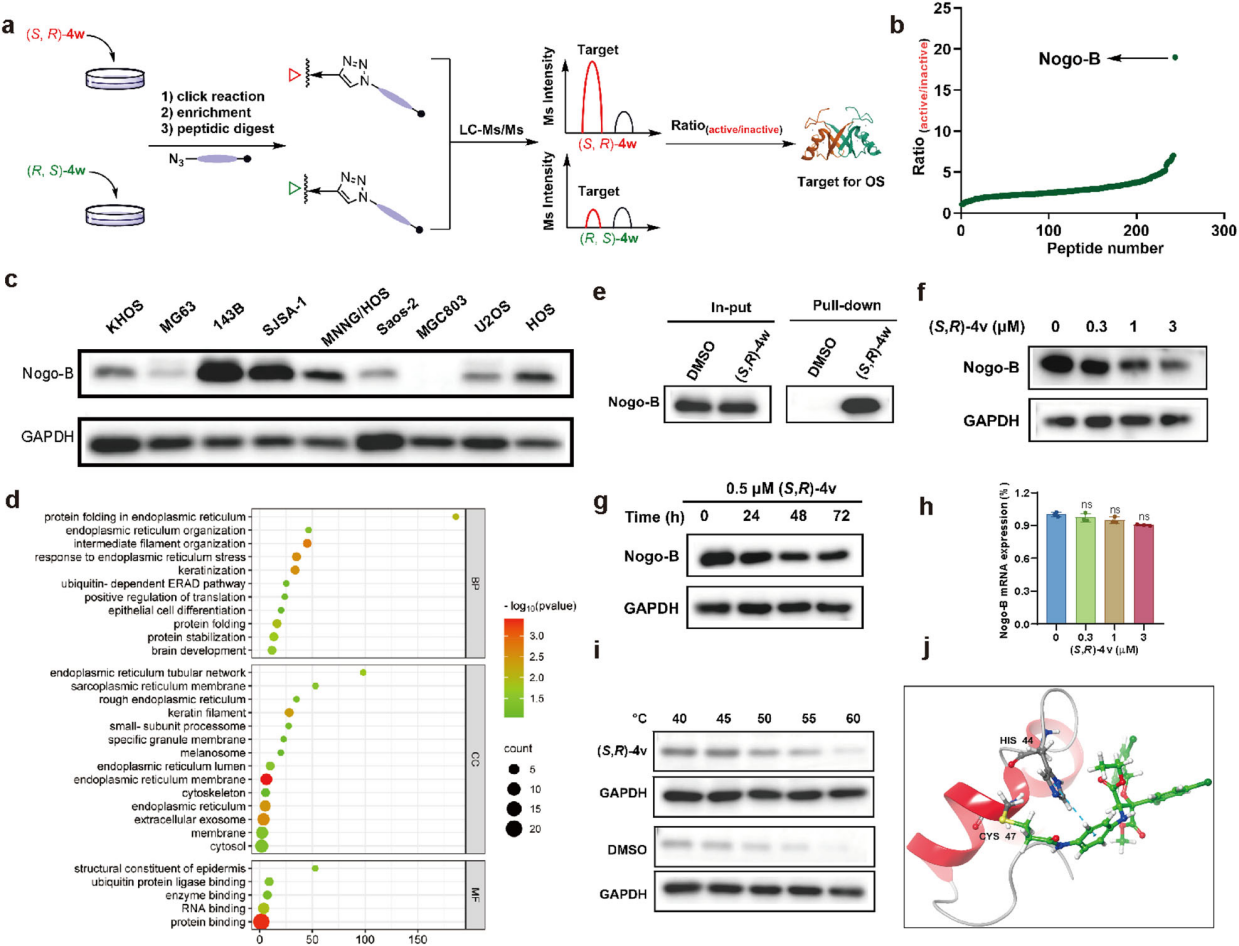
(*S*,*R*)-4v exerts anticancer bioactivity by directly binding to the Nogo-B protein. **a** New strategies for search targets with label-free ABPP. **b** The enrichment ratios of (*S*,*R*)-**4w**/(*R*,*S*)-**4w**. **c** The Nogo-B expression in 9 different cell lines. **d** GO classification of the top 20 specific targets based on BP, CC and MF. **e** Pull-down and input experiment analysis using Nogo-B antibody confirmed that the protein pulled down by (*S*,*R*)-**4w** was Nogo-B. **f** The expression of Nogo-B protein was detected in 143B cells treated with different concentrations of (*S*,*R*)-**4v** for 24 h. **g** The expression of Nogo-B protein was detected in 143B cells were treated with (*S*, *R*)-**4v** for different time. **h** mRNA expression of Nogo-B was detected in 143B cells treated with different concentrations of (*S*,*R*)-**4v** for 24 h. **i** CETSA assay evaluation of the binding between the compound (*S*, *R*)-**4v** and Nogo-B. **j** The binding of Nogo-B with (*S*, *R*)-**4v** was investigated by molecular docking simulation. The data are reported as the mean ± SD. All data are representative of three independent experiments. ns=no significant.

After these initial experiments, the covalent binding targets of (*S*,*R*)-**4w** were identified using its less bioactive isomer from the one-pot reaction as control for the relative quantitative analysis of LC-MS/MS (Fig. 4a). In brief, the experimental group was treated with 50 μM (*S*,*R*)-**4w** and the blank control was treated with the same equivalent DMSO and (*R*,*S*)-**4w**. After cell lysis and click reaction, labeled proteins were then enriched by streptavidin beads and digested by trypsin, and the resulting peptides were identified by LC-MS/MS analysis. A total of about 471 proteins were identified as the potential targets of (*S*,*R*)-**4w** in three independent experiments. They were ranked by enrichment ratios of (*S*,*R*)-**4w**/DMSO group (Supplementary Fig. S4). To minimize the disturbance of nonspecific binding proteins, only proteins with the enrichment ratios > 5 was selected for further analysis as potential targets of the covalently-acting probe. The identified proteins were then analyzed by the a relatively quantitative ratio of the proteins fished by more bioactive (*S*,*R*)-**4w** and less bioactive (*R*,*S*)-**4w**. In these experiments, Nogo (RTN4), a ∼50 kDa protein was identified as the first top ranked protein with a relatively quantitative ratio of about 19 (Fig. 4b). This suggests that Nogo-B is most likely the ∼50 kDa (*S*,*R*)-**4w** target protein identified by gel-based ABPP profiling. Previous studies [35, 36] have demonstrated that Nogo-A, Nogo-B, and Nogo-C are three isoforms of RTN4/Nogo protein family. Subsequently, we investigated the expression of Nogo-B in 9 distinct cell lines (Fig. 4c). The findings revealed that 143B cells exhibited the highest level of Nogo-B expression, followed by SJSA-1 cells, which was consistent with the tested IC_50_ values and indicated the selective action of (*S*,*R*)-**4v** on Nogo-B protein resulting in cellular apoptosis.

To gain further insights into the biological functions of the proteins enriched by the probe, we classified the top 20 targets with the highest enrichment ratio using the gene ontology (GO) annotation database (Fig. 4d). Regarding biological processes (BP), these targets predominantly participate in metabolic and cellular processes. Concerning cellular components (CC), they are primarily localized in organelles such as endoplasmic reticulum and cell membranes. In terms of molecular function (MF) classification, these targets mainly exhibit binding towards diverse protein. Among these results, Nogo-B are reported [37, 38] to be involved in most of these processes, which imply that Nogo-B is a potential target of (*S*,*R*)-**4v**.

To validate the MS results, we implemented target enrichment by a pull-down experiment that was then applied to western blot analysis using the Nogo-B antibody. As shown in Fig. 4e, an intense labeling band appeared in the probe-treated sample but not in the DMSO control, demonstrating that Nogo-B indeed bound (*S*,*R*)-**4v**. To understand the mechanism underlying the regulation of Nogo-B by (*S*,*R*)-**4v**, we measured the RNA and protein levels of Nogo-B in the 143B cells treated with (*S*,*R*)-**4v**. Interestingly, we found that (*S*,*R*)-**4v** downregulated Nogo-B protein level in 143B cells in a concentration (Fig. 4f) and time-dependent manner (Fig. 4g) but did not result in an obvious change in the mRNA level (Fig. 4h). These results illustrate that (*S*,*R*)-**4v** can efficiently bound to Nogo-B and down-regulate its expression in 143B cells. To confirm whether Nogo-B is a direct binding target of (*S*,*R*)-**4v**, the cellular thermal shift assay (CETSA) was conducted to test the direct interaction between Nogo-B and (*S*,*R*)-**4v** [39]. The temperature-dependent CETSA indicated that (*S*,*R*)-**4v** enhanced the stability of Nogo-B (Fig. 4i). The data demonstrated that the inhibitory effect of (*S*,*R*)-**4v** on 143B cells to certain extent depends on Nogo-B.

Recently, acrylamide probes have been shown to covalently modify specific cysteine residue 1101 (Cys1101) on RTN4 by LC-MS/MS assay, thereby impairing colorectal cancer cell growth [37]. To further identify the binding site for (*S*,*R*)-**4v** in Nogo-B, we conducted molecular docking simulation (AutoDock4) to predict the interaction of compound (*S*,*R*)-**4v** with the Nogo-B protein. The results indicate that (*S*,*R*)-**4v** may bind to the Nogo-B protein by forming a covalent bond with the residue cysteine 47 (Cys47) and a π–π stacking interaction with the residue histidine 44 (His44) in Nogo-B (Fig. 4j).

### Nogo-B is a potential therapeutic target for OS

**Fig. 5:**
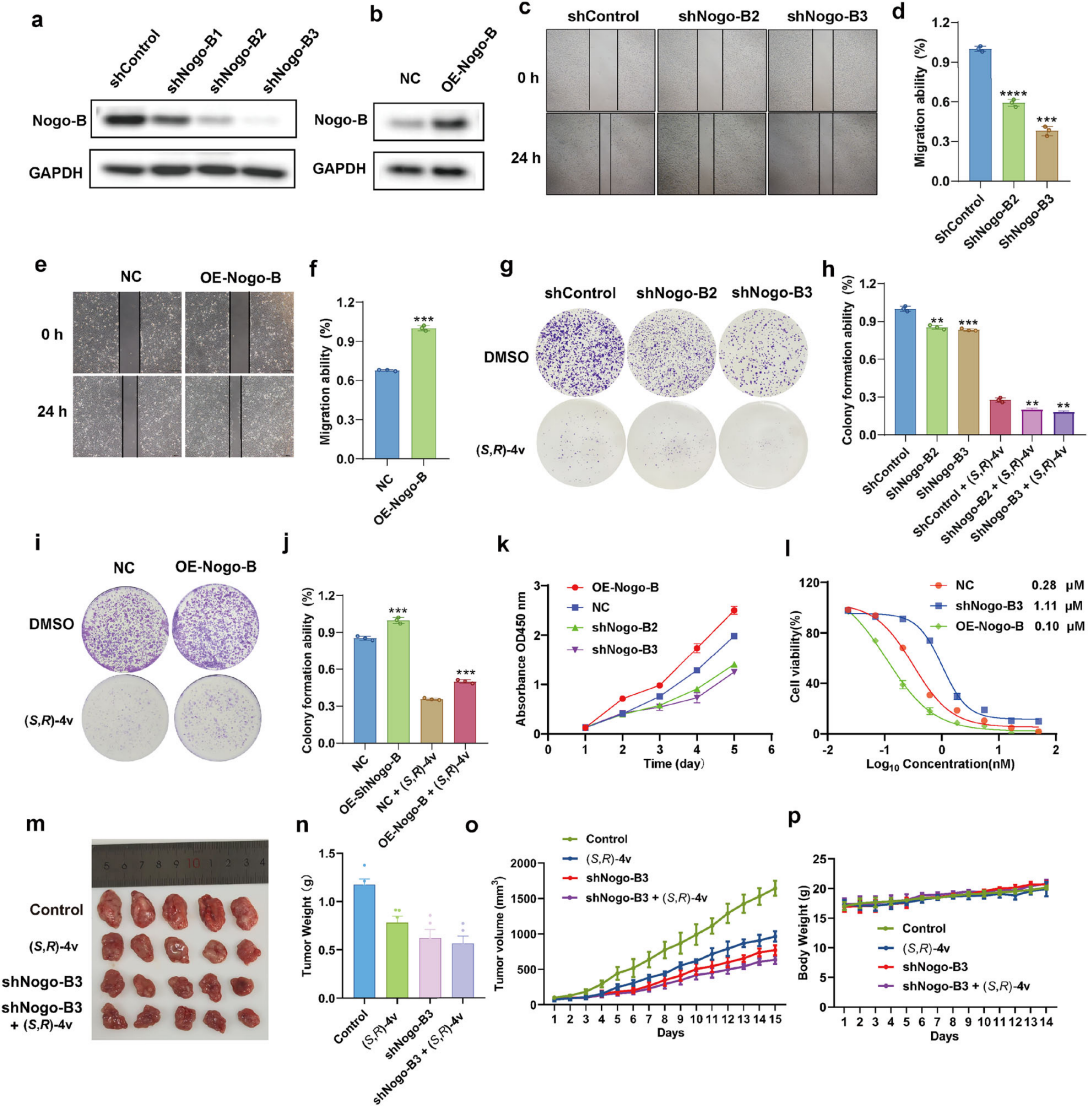
The (*S*,*R*) -4v-induced apoptosis is mediated by Nogo-B inhibition of the PI3K/AKT-dependent NF-κB pathway. **a** Nogo-B knockdown to verify experiment. **b** Nogo-B overexpression to verify experiment. **c** Cells migration of shControl, shNogo-B2 and shNogo-B3 group were tested by cell wound healing assay. **d** Quantitative results of wound healing assay. **e** Cells migration of NC and OE-Nogo-B group were tested by cell wound healing assay. **f** Quantitative results of wound healing assay. **g** Cells proliferation of shControl, shNogo-B2 and shNogo-B3 group were tested by colony formation assay. **h** Quantitative results of colony formation assay. **i** Cells proliferation of NC and OE-Nogo-B group were tested by colony formation assay. **j** Quantitative results of colony formation assay. **k** The effect of Nogo-B knockdown and overexpression on 143B cell activity was detected by CCK-8 assay. **l** Analysis of the cytotoxicity of treatment with (*S*,*R*)-**4v** in shNogo-B3 and OE-Nogo-B 143B cells. **m**-**p** Examination on tumors formed by 143B and shNogo-B3 cells in Balb/c mice: **m** Representative picture of tumors derived from mice. **n** Tumor weight. **o** Tumor volume. **p** Average body weights. The data are reported as the mean ± SD. All data are representative of three independent experiments. Ns=no significant, **P < 0.01; ***P < 0.005; ****P < 0.001.

To detect the role of Nogo-B in OS 143B cells, we performed the Nogo-B knockdown (Fig. 5a) and Nogo-B overexpression (Fig. 5b) assay to test whether the inhibitory effect of (*S*,*R*)-**4v** on 143B cells is dependent on Nogo-B. The migration ability of control, Nogo-B knockdown and Nogo-B overexpression 143B cells were compared by cell wound healing, and it was found that the percentage of wound healing distance between cells with knockdown of Nogo-B was significantly lower than that in the control group (Fig. 5c), and overexpression group exerted the opposite effect (Fig. 5e). In addition, colony formation experiments showed that the colony growth of 143B cells with knockdown of Nogo-B was significantly reduced compared with the control group (Fig. 5g), while overexpression of Nogo-B significantly increased the colony growth (Fig. 5i). Finally, we used CCK-8 assay to assess the cell proliferation rate when Nogo-B was knocked down and overexpressed. Compared with the control group, the proliferation rate of 143B cells with Nogo-B knockdown was significantly reduced (Fig. 5k), and overexpression group exerted the opposite effect (Fig. 5k). These results suggest that Nogo-B is essential for the proliferation and migration of OS 143B cells.

To investigate whether the antiproliferative properties of (*S*,*R*)-**4v** are Nogo-B-dependent, we treated the Nogo-B knockdown and the Nogo-B overexpression 143B cells with (*S*,*R*)-**4v**, respectively (Fig. 5l). Notably, down-regulated Nogo-B substantially reduced the inhibitory effect of (*S*,*R*)-**4v** on cell proliferation (IC_50_ = 0.28 vs 1.11 µM), and the Nogo-B overexpression cells were more sensitive to (*S*,*R*)-**4v** compared to the control group (IC_50_ = 0.28 vs 0.10 µM). Although the antiproliferative properties of (*S*,*R*)-**4v** are dependent on Nogo-B, the effect is not particularly pronounced, and these results suggest that Nogo-B may not be the only target that causes phenotypic changes. Recently, Duan group reported a 6-methyl flavone inhibitor [40], which can effectively inhibit Nogo-B expression in the liver to treat metabolic syndrome caused by glycolipid metabolism dysregulation, with a IC_50_ value of 15.85 μM. Then, we tested the activity of the 6-methyl flavone inhibitor in 143B cells. Unfortunately, due to the differences between cell lines, this inhibitor did not have a good inhibitory effect on 143B cells even at a concentration of 50 μM ( < 5%), whereas the inhibitory activity of compound (*S*,*R*)-**4v** was 99% at a concentration of 50 μM (Supplementary Fig. S6). Therefore, 6-methyl flavone is not helpful to be used as a reference compound for this study. Finally, we used Nogo-B knockdown 143B cells to construct a xenograft tumor model to examine the effect of Nogo-B on tumor progression in mice. The results showed that Nogo-B knockdown effectively suppressed tumor volume and tumor weight in mice without significantly influencing mouse weight (Fig. 5m–p). We also conducted tumor formation experiments to examine the effect of (*S*,*R*)-**4v** on mice with 143B cells with Nogo-B knockdown. The results showed that (*S*,*R*)-**4v** significantly inhibited tumor volume and tumor weight in mice injected with control 143B cells (Fig. 5m–p). However, the inhibitory effect of (*S*,*R*)-**4v** was greatly weakened in mice injected with shNogo-B3 cells followed by (*S*,*R*)-**4v** treatment (Fig. 5m–p). In addition, neither (*S*,*R*)-**4v** treatment nor Nogo-B knockdown affected the weight of mice (Fig. 5m–p). Collectively, the knockdown of Nogo-B eliminated the anti-tumor effect of (*S*,*R*)-**4v**, indicating that Nogo-B played an important role in the anti-tumor effect of (*S*,*R*)-**4v**.

### (*S*,*R*)-4v promotes apoptosis of OS cells by inhibiting the PI3K/AKT/NF-κB signaling pathway regulated by Nogo-B

**Fig. 6:**
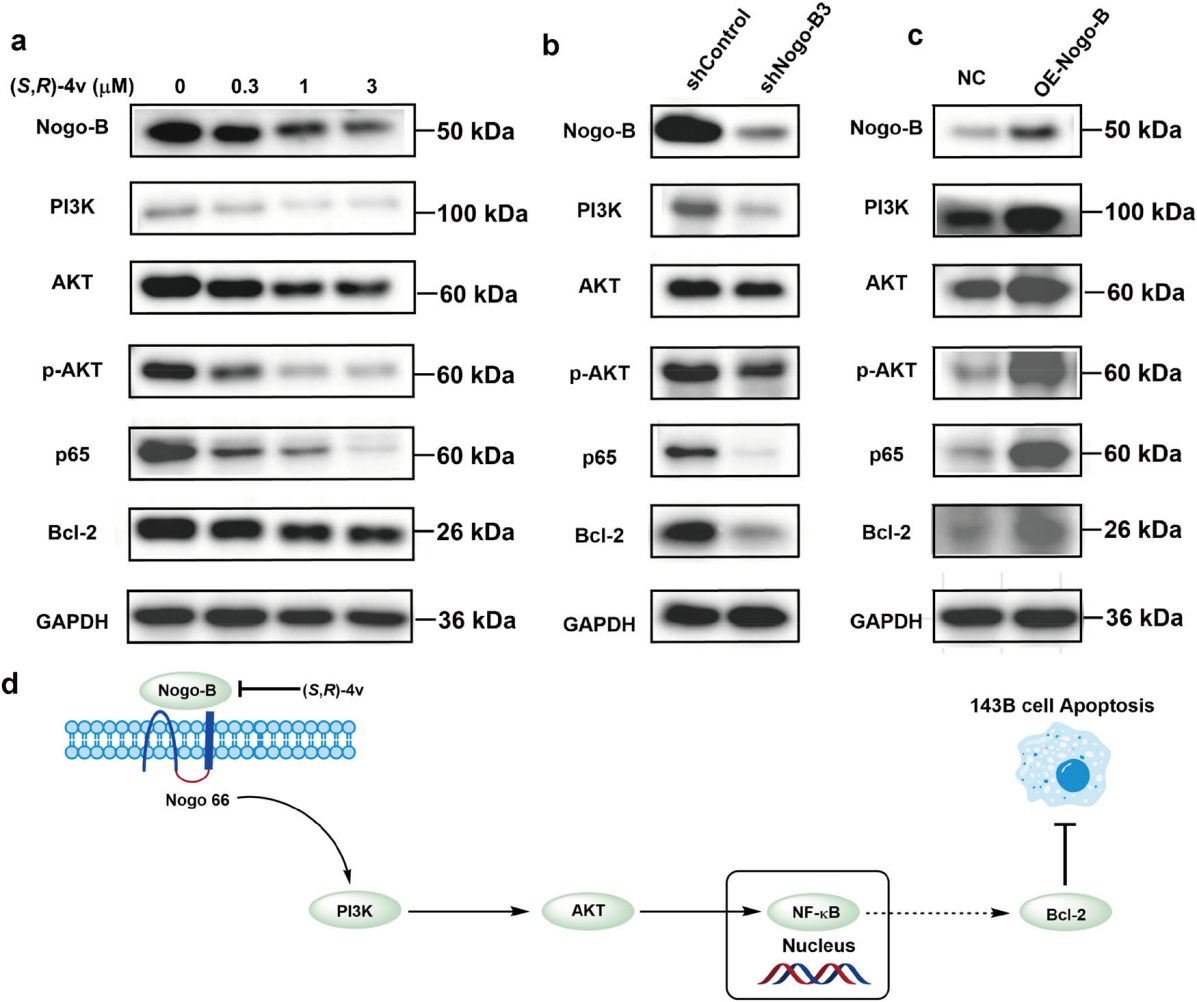
(*S*,*R*)-4v inhibits activation of the PI3K/AKT/NF-κB signaling pathway regulated by Nogo-B. **a** Western blot showing the expression of PI3K/AKT/NF-κB pathway-related proteins, including Nogo-B, PI3K, AKT, p-AKT, p65 and Bcl-2. **b**, **c** Western blot showing the expression of the Nogo-B, PI3K, AKT, p-AKT, NF-κB and Bcl-2 proteins in the shNogo-B3 and OE-Nogo-B 143B cells. **d** Schematic diagram summarizing how (*S*,*R*)-**4v** suppresses Nogo-B expression and tumorigenesis. The data are reported as the mean ± SD. All data are representative of three independent experiments.

It has been shown that Nogo-B can activate the PI3K/AKT pathway [41, 42] and the PI3K/AKT pathway is closely involved in regulation of NF-κB activity [43, 44]. The NF-κB plays key roles in diverse physiologic and pathologic processes, including apoptosis, proliferation, migration, invasion, and metastasis [45–47]. A number of studies showed that the inhibition of the NF-κB pathway affects the proliferation or apoptosis of OS cells [48, 49]. Therefore, we investigated whether compound (*S*,*R*)-**4v** inhibits proliferation and migration of OS by modulating the NF-κB pathway. Western blotting analysis, as shown in Fig. 6a, revealed that treatment with different concentrations of (*S*,*R*)-**4v** resulted in a dose-dependent decrease of p65 protein in 143B cells. Further, (*S*,*R*)-**4v** markedly reduced the protein level of Bcl-2, which is a critical downstream target of the NF-κB pathway and closely related to the progression of cancers [21]. These results demonstrate that (*S*,*R*)-**4v** can effectively bound to Nogo-B and inhibit the NF-κB signaling pathway.

To determine how (*S*,*R*)-**4v** blunted the Nogo-B-induced activation of NF-κB in 143B cells, we examined the effects of different concentrations of (*S*,*R*)-**4v** on PI3K/AKT pathway in 143B cells. Interestingly, (*S*,*R*)-**4v** inhibited the activation of PI3K, AKT and p-AKT in a dose-dependent manner (Fig. 6a). Next, we explored whether (*S*,*R*)-**4v** could inhibit the activation of PI3K/AKT pathway via Nogo-B. As shown in Fig. 6b, shNogo-B3 cells contained significantly lower levels of PI3K, AKT, p-AKT p65 and Bcl-2 compared to the respective controls. Contrary to the Nogo-B knockdown group, with the increase of Nogo-B expression, the expression levels of PI3K, AKT, p-AKT, p65 and Bcl-2 in cells were significantly higher than those in the control group (Fig. 6c). These results confirm that a role of Nogo-B in activating the PI3K/AKT pathway. Together, these results imply that (*S*,*R*)-**4v** can blunt Nogo-B, which in turn impairs PI3K/AKT and NF-κB activities in 143B cells [50].

## Discussion

In the field of cancer therapy, the identification and verification of novel targets have consistently been the core of research, which holds immense potential for transforming cancer treatment approaches and enhancing patient prognosis. OS is one of the most complex malignant bone tumors [1, 2]. Due to its high mutation rate, there has been no significant improvement in the targeted therapy of OS for decades. Although several potential therapeutic targets for osteosarcoma have been identified [7–15], their clinical efficacy remains unsatisfactory. Activity-based proteomics is widely employed in the identification of novel cancer targets [17–23]. However, the application of this strategy in discovering therapeutic targets for osteosarcoma is severely limited due to a lack of highly bioactive probe molecules. In this study, we utilize a one-pot multi-component reaction to rapidly construct a library of 3D covalent molecules exhibiting stereostructural diversity. Subsequently, through phenotypic screening, small molecule (*S*,*R*)-**4v** was found to have good anti-OS activity from our in house patrimonial library. (*S*,*R*)-**4v** selectively inhibited cell proliferation and has excellent anticancer activity in OS cell lines, in particular 143B and SJSA-1, and it showed low activity in human gastric cancer cells MGC803 and human non-small cell lung cancer cells A549. Additionally, during in vivo treatment, the small molecule (*S*,*R*)-**4v** can significantly repress tumor growth without manifesting obvious toxicity. Subsequently, via label-free ABPP studies using a relative quantitative analysis with (*S*,*R*)-**4w** as probe and (*R*,*S*)-**4w** as control, we successfully identified Nogo-B as the primary cellular target of (*S*,*R*)-**4v**. CETSA experiments, Nogo-B knockdown experiments, Nogo-B overexpression experiments and molecular docking experiments have further validated that Nogo-B is the principal target of (*S*,*R*)-**4v**. These findings suggest that the compound (*S*,*R*)-**4v** can selectively bind to Nogo-B, effectively suppressing the proliferation and migration of tumor cells by down-regulating the expression level of Nogo-B.

Nogo-B is widely expressed across various tissues, where it regulates important cellular events including angiogenesis, macrophage movement, vascular modeling, sphingolipid homeostasis, inflammation, and immune responses [51–53]. Moreover, a lower expression of Nogo-B in multiple types of cancers predicts better overall survival for patients. Particularly, inhibition of Nogo-B expression is effective in preventing the development of hepatocellular carcinoma, colorectal cancer and Breast cancer, indicating Nogo-B may be a possible therapeutic target for improving these cancers [54–56]. However, involvement of Nogo-B in OS growth and survival remains poorly understood to date. Our study shows that Nogo-B is essential for the proliferation and survival of OS cells. This not only provides a new target for the targeted treatment of OS, but also offers a new perspective for understanding the functional diversity of Nogo-B in different cancers. We discovered that the downregulation of Nogo-B expression results in reduced AKT activation that is an important signaling event associated with tumorigenesis and metastatis [57]. In addition, AKT has been shown to disable its pro-apoptotic function by directly phosphorylating pro-apoptotic proteins [58]. Nogo-B knockdown impairs AKT expression, which further supports the pro-survival role of Nogo-B in OS cells.

In summary, we found and demonstrated that the compound(*S*,*R*)-**4v** covalently bound to Nogo-B and inhibited the progression of OS by suppressing Nogo-B activity and PI3K/AKT/NF-κB/Bcl-2 signaling pathway. These findings not only emphasize Nogo-B as a potential therapeutic target for OS, but also support the utilization of compound (*S*,*R*)-**4v** as a precursor compound for OS treatment, facilitating subsequent drug development. In conclusion, our strategy offers a rapid and efficient covalent probe discovery and holds significant value in the identification of novel targets for rare cancers.

## Methods

### Cell culture

143B, HOS, SJSA-1, MG63, MNNG/HOS, KHOS, U2OS, MGC803, Saos-2 and A549 cell lines were all purchased from the Type Culture Collection of the Chinese Academy of Sciences (Shanghai, China). The absence of mycoplasma contamination in the cells was verified through pre-experimental mycoplasma testing. The cells were cultured in a controlled environment at 37 °C with 5% CO_2_ and maintained under humid conditions, and subcultured when 80% confluent was reached, cultured in MEM, DMEM, RPMI-1640, or McCoy’s 5 A media (Basal media Technolo-gies Co, Ltd) with 10% (v/v) fetal bovine serum (BI) and 1% (v/v) penicillin-streptomycin (Basal media Technologies Co, Ltd.).

### Cell viability assay

The in vitro inhibitory effect on cell proliferation was measured using CCK-8 assay (Beyotime, C0043). Different kinds of cells (2500 or 3000 per well) were seeded into 96-well plates over-night and then treated with various concentrations of the tested compound (the concentration of DMSO was kept at <0.1%). After 24, 48 or 72 h, 100 mL medium containing 10% CCK-8 was added into each well for 2 h at 37 °C. The optical density of each well was measured at 450 nm using a microplate reader (Molecular Devices Corporation, USA). IC_50_ values were calculated using GraphPad Prism 8.0 software.

### Apoptosis analysis by flow cytometry

An Annexin V-FITC/PI Apoptosis Detection Kit (KeyGen, Nanjing, China) was used to evaluate apoptosis. 143B cells were seeded in six-well plates at 1.5 × 10^5^ cells/mL and cultured at 37 °C in 5% incubator for 24 h before adding 0 μM, 0.3 μM, and 1 μM concentrations of the compound for 24 h. Cells were incubated with 5 μl of FITC-conjugated Annexin V and 5 μl of PI for 15 min at room temperature in the dark, and 400 μl of binding buffer was then added (Beyotime, C1062M). Apoptotic cells were analyzed in a FACSCalibur flow cytometer (BD Biosciences).

### In situ proteome labeling

For in situ proteome labeling, 143B cells were grown to 90% confluency under the condition of 37 °C with 5% CO_2_. The medium was removed and washed twice with PBS and then treated with 3 mL probe-containing fresh medium in the presence or absence of excessive competitors (diluted from DMSO stocks whereby DMSO never exceeded 1% in the final solution). After 2 h of incubation, the medium was aspirated and cells were washed twice with PBS to remove excessive probe. The cells were lysed with RIPA buffer (Thermo Scientific^TM^, #89900) including 1× phosphatase inhibitors (Thermo Scientific^TM^, #A32955) on ice for 30 min and centrifuged for 30 min (13000 rpm, 4 °C) to get a soluble protein solution. Eventually, the protein concentrations were determined by BCA protein assay (Beyotime, P0009) and then diluted to 2 mg/mL with RIPA buffer. A freshly premixed click chemistry reaction cocktail was added (50 μM Biotin-N_3_ from 25 mM stock solution in DMSO, 100 μM TBTA from 50 mM freshly prepared stock solution in DMSO, 1 mM TCEP from 1 M freshly prepared stock solution in deionized water, and 1 mM CuSO_4_ from 1 M freshly prepared stock solution in deionized water). The reaction was further incubated for 1 h prior to addition of pre-chilled methanol (−20 °C). The precipitated proteins were subsequently collected by centrifugation (6000 rpm, 10 min at 4 °C) and washed twice with 500 μL of prechilled methanol. The samples were dissolved in 1 × SDS loading buffer and heated for 10 min at 95 °C. 20 μg proteins for each lane were loaded on SDS − PAGE (10% gel) and then visualized by in-gel fluorescence scanning (Typhoon FLA 9500).

### Streptavidin blot

Proteins on the SDS gel were transferred to a polyvinylidene fluoride (PVDF) membrane at room temperature for 90 min. The membrane was further blocked in 5% nonfat dried milk solution for 2 h at room temperature. After blocking, membranes were washed with TBST (3 × 10 min) and then the membrane was incubated with streptavidin-horseradish peroxidase (HRP, Beyotime, A0303, with 1:5000 dilution in 5% nonfat dried milk PBST solution) for 2 h at room temperature. After incubation, membranes were washed with TBST (3 × 10 min) and detected by enhanced chemiluminescence (ECL) western blotting detection reagents (BioRad). The PVDF membranes were scanned using the ChemiDoc XRS+ system (Bio-Rad).

### Pull down and targets validation

To identify the interacting cellular targets of (*S*,*R*)-**4w**, pull-down (PD) experiments were carried out, and followed by Western blotting (WB) and LC-MS/MS. 143B cells were grown to 90% confluency under the condition of 37 °C with 5% CO_2_. The medium was removed and the cells were washed 3 times with PBS and treated with probe-containing medium in the presence or absence of corresponding competitors (final concentration of the probe was 50 μM, DMSO never exceeded 1% in the final solution). After 2 h of incubation, the medium was aspirated, and cells were washed twice with PBS to remove excessive probe. The cells were lysed with RIPA buffer (Thermo Scientific^TM^, #89900) including 1× phosphatase inhibitors (Thermo Scientific^TM^, #A32955) on ice for 30 min and centrifuged for 30 min (13000 rpm, 4 °C) to get a soluble protein solution. Eventually, the protein concentrations were determined by BCA protein assay (Beyotime, P0009) and then diluted to 2 mg/mL with RIPA buffer. A freshly premixed click chemistry reaction cocktail was added (50 μM Biotin-N_3_ from 25 mM stock solution in DMSO, 100 μM TBTA from 50 mM freshly prepared stock solution in DMSO, 1 mM TCEP from 1 M freshly prepared stock solution in deionized water, and 1 mM CuSO_4_ from 1 M freshly prepared stock solution in deionized water). The reaction was further incubated for 1 h with gentle mixing prior to precipitation by addition of pre-chilled methyl alcohol (−20 °C). Precipitated proteins were subsequently collected by centrifugation (6000 rpm, 10 min at 4 °C) and washed twice with 500 μL of prechilled methanol. The samples were dissolved in PBS containing 1% SDS. Upon incubation with 100 μL streptavidin beads (Thermo Scientific^TM^, #65001) for 4 h at rt, the beads were washed with PBS containing 1% SDS (2 ×1 mL ×5 min) and 0.1% SDS (2 × 1 mL × 5 min), PBS (2 × 1 mL × 5 min). The enriched proteins were eluted by 1× loading buffer at 95 °C for 10 min and separated by SDS-PAGE (10%). The enriched bands were visualized by Coomassie Blue staining (Beyotime, P0017). Control pull-down experiments using the DMSO were carried out concurrently with live cells. Next, cut the excised gel slice into 1 mm cubes and transfer the gel cubes to microcentrifuge tube. To destain gel pieces, add sufficient amount of 50 mM NH_4_CO_3_ containing 50% ACN, vortex for 5 min, suck out the liquid, repeat for 3 times. Then dehydrate, add enough ACN, vortex for 5 min, discard the liquid, and repeat 3 times. At reduction and alkylation step, add sufficient amount of 20 mM DTT, standing at 55 °C for 45 min, centrifuge after cooling, and suck out the liquid, then add enough IAA of 55 mM for 45 min at room temperature. Remove liquid, add enough ACN, swirl it for 5 min, suck out the liquid, and repeat 3 times. And remove ACN, speed vac to dryness. Add 100 uL of 50 mM NH_4_CO_3_ and 2 μg of modified Gold Trypsin (Mass Spectrometry Grade, Promega) to tube for digestion at 37 °C for 12 ~ 16 h. Finally, add 30 uL 0.1% FA and centrifuge at 14,000 × *g* for 15 min. The supernatant is transferred to sample tube for mass spectrometry detection.

### LC-MS/MS analysis

The solution containing peptides (3 μL) was subjected to online nano-flow liquid chromatography via the easy-nano LC system (Thermo Fisher Scientific). The LC system was connected to a 2-cm pre-column with an internal diameter of 100 μm, filled with 5 μm C18 resin (Thermo Fisher Scientific). After the pre-column reaction, a 75 μm × 15 cm capillary column was filled with 3 μm C18 resin (Thermo Fisher Scientific). The mobile component was composed of two phases: solution A, 2% ACN/0.1% FA in water, and solution B, 2% water/0.1% FA in ACN. Peptides were eluted from the pre-column and analytical column by stepwise gradient elution encompassing the following steps: incubation for 0 min in 5% solution B, followed by 10% solution B for 4 min; 20% solution B for 50 min, 35% solution B for 20 min, and 95% solution B for 6 min, followed by a column wash with 95% solution B for 10 min. A constant flow rate of 300 nL/min was maintained. Mass spectra were acquired with an Orbitrap Q-Exactive mass spectrometer in a data-dependent manner, with automatic switching between MS and MS/MS scans using the top-20 method. MS spectra were obtained at a resolution of 70,000 with a target value of 3e6 or a maximum injection time of 20 ms. The scan range was limited to 350–2000 m/z. Peptide fragmentation was performed via higher-energy collision dissociation (HCD) with the energy set at normalized collision energy of 27. The MS/MS spectra were acquired at a resolution of 17,500, with a target value of 2e5 ions or a maximum inject time of 60 ms, and the isolation window was set at 2.0 m/z.

### Western blot analysis

After the enrichment experiment by streptavidin beads, proteins on the SDS gel were transferred to a polyvinylidene fluoride (PVDF) membrane at room temperature for 90 min The membrane was further blocked in 5% nonfat dried milk solution for 2 h at room temperature. After blocking, membranes were washed with TBST (3 × 10 min) and the membrane was incubated with primary rabbit polyclonal anti-Nogo-B antibody (dissolved in 5% milk in PBST) for 12 h at 4 °C, followed by TBST washing. Subsequently, goat anti-rabbit immunoglobulin G (IgG) HRP-conjugated secondary antibody (Bio-Rad, with 1:3000 dilution in 5% nonfat dried milk PBST solution) was added, and the membrane was incubated at room temperature for 2 h. Finally, the membrane was washed with TBST (3 × 10 min), and signals were detected by ECL western blotting detection reagents (Bio-Rad) using the ChemiDoc XRS+ system (BioRad). HRP-labeled Streptavidin (A0305, Beyotime); anti-RTN4/Nogo-B (10950-1, Proteintech); anti-PI3K (4263S, Cell Signaling Technology); anti-AKT (4685S, Cell Signaling Technology); anti-p-AKT (9271,Cell Signaling Technology); anti-p65 (AF0246, Beyotime); anti-Bcl-2 (AB112, Beyotime); anti-GAPDH (AF0006, Beyotime); β-actin (4970S, Cell Signaling Technology); HRP-labeled Goat Anti-Rabbit IgG (H + L) (A0208, Beyotime); HRP-labeled Goat Anti-Mouse IgG (H + L) (A0216, Beyotime).

### Cellular thermal shift assay (CETSA)

For cellular thermal shift assay (CETSA), experiments were carried out similarly as published protocols. 143B cells were seeded in 10 cm cell culture dishes and grown till ~90% confluence. Subsequently, the cells were incubated with (*S*,*R*)-**4v** (50 μM in fresh growth medium), for 2 h in the CO_2_ incubator at 37 °C. The same volume of DMSO was used to serve as a negative control in a separate dish. The medium was subsequently aspirated and cells were washed with PBS (3 × 1 mL), then harvested by scraping in fresh PBS (1 mL). Cell pellets were isolated by centrifugation (800 rpm, 5 min, 4 °C), resuspended in 550 μL of PBS and distributed into 5 different 1.5 mL of tubes with 100 μL of cell suspension in each tube for both DMSO control and inhibitor-treated cells. The tubes were heated at the designated temperature endpoints (40–60 °C) for 3 min on a heating block. After heating, the tubes were removed and incubated at room temperature for another 3 min. After that, cells were freeze-thawed thrice by using liquid nitrogen and a heating block set at 25 °C to ensure a uniform temperature between tubes. 50 μL of supernatant were collected by centrifugation (Eppendorf centrifuge 5415 R, 13,000 rpm × 1 h) from each resulting cell lysate at 4 °C. Then the residue was mixed with 5 × standard SDS-loading buffer, after heating for 10 min at 95 °C with gentle mixing and separation by SDS-PAGE, the samples were transferred to a PVDF membrane for WB analysis.

### Quantitative real-time PCR assay

Total RNA in CRC was extracted using RNAeasy™ Animal RNA Isolation Kit with Spin Column (Beyotime, R0024) according to the manufacturer’s protocol. Relative quantitation by real-time PCR involved SYBR Green detection of PCR productsin real-time with the ABl PRlSM 7700 Sequence Detection System (Applied Biosystems). GAPDH RNA was amplified as a reference standard. The reactions were performed in triplicateby heating the reactant to 95 °C for 5 min, followed by 40 cycles of 94 °C for 30 s, 58 °C for 30 s, and 72 °C for 30 s, respectively. Primers were as follows:

Nogo-B forward: GTTGACCTCCTGTACTGGAGA,

Nogo-B reverse: CTGTTACGCTCACAATGCTGA,

GAPDH forward: CAAATTCCATGGCACCGTCA,

GAPDH reverse: GGAGTGGGTGTCGCTGTTGA.

### Wound healing assay

143B cells (1 × 10^5^) were grown in a 6-well plate to a 90% confluent, the cell monolayer was scratched to the same width using a yellow pipette tip. After washing with PBS three times (500 μL × 3) and incubated in MEM. The wound area was imaged at 0- and 24-h time points and the area was analyzed by ImageJ software (National Institutes of Health, MD).

### CCK-8 for cell proliferation assay

CCK-8 Kit (Solabio, Beijing, China) was used to measure the proliferation of 143B cells. ShConrtol, shNogo-B1, shNogo-B2 or shNogo-B3, 1000 cells from each group were seeded in 96-well plates in triplicate with a final volume of 100 µL complete medium and cultured at 37 °C. Viable cell numbers were measured daily over 5 days. At indicated time, each well was added into 10 μL CCK8 solution and incubated for 2 h at 37 °C. The absorbance was detected at 450 nm using an ultraviolet spectrophotometer (Implen, Germany).

### Colony formation assay

ShConrtol, shNogo-B1, shNogo-B2, shNogo-B3 or OE-Nogo-B cells were seeded in 6-well plates in three duplicate wells (1000 cells/well) and treated with the indicated concentration of (*S*,*R*)-**4v** or vehicle control. After 10 days, cells were washed 3 times with PBS and fixed with 4% paraformaldehyde (Beyotime, P0099), and stained with crystal violet (Beyotime, C0121) for 15 min. The colonies that contained more than 50 cells were counted.

### Morphological observation

143B cells were inoculated into 6-well plates at a concentration of 1 × 10^5^ cells/ml. 24 h later, (*S*,*R*)-**4v** with different concentrations (0, 200, 400, 800 nmol) was added and cultured in a 37 °C, 5% CO_2_ incubator for 24 h. The cell adhesion and growth were observed under an inverted microscope and photographed.

### Plasmids cell transfection

Human Nogo-B overexpression plasmid was designed and constructed by Genechem Company (Shanghai, China). The pcDNA3.1 carrier was used to construct the Nogo-B overexpression and control plasmid. The GV493 construct (hU6-MCS-CBh-gcGFP-IRES-puromycin) was used to express the shRNA. Cells in the logarithmic growth stage were seeded into 6-well plates. After reaching 80% confluence, cells were transfected with plasmid using the transfection reagent (Lipofectamine™ 2000, Invitrogen™, Thermo Scientific^TM^, #11668500) according to the manufacturer’s instructions. 48 h later, collect cells for the subsequent experiment.

### Molecular docking

In this study, all molecular docking experiments were conducted using the AutoDock4.1 (AD4) software [43]. Initially, water and cofactors were removed from these protein crystal structures (2G31), and receptor structures were hydrogenated using AutoDockTools (ADT). Gasteiger charges were calculated, and PDBQT files were generated accordingly. For compounds (*S*,*R*)-**4v**, a flexible docking approach was employed to enhance the accuracy of the results, allowing for a more precise prediction of possible binding modes between the receptor and ligand. To reduce unnecessary computational complexity, specific amino acid residues in the protein binding sites were set as flexible, while the rest remained fixed. Additionally, the ligand was hydrogenated using ADT, Gasteiger charges were computed, and torsion centers and torsion bonds were detected to construct a torsion tree, resulting in the generation of the ligand PDBQT file. Subsequently, AutoGrid4 was utilized to generate a receptor grid with dimensions of 60 Å × 60 Å × 60 Å and a spacing of 0.375 Å, using the original ligand coordinates in the protein crystal structure as the center. The receptor interaction energy was calculated using ligand atomic types as probes. Finally, the Lamarckian Genetic Algorithm (LGA) was employed for docking simulations, outputting the top 50 docking poses. The optimal docking structure was selected based on the analysis of docking poses, scoring results, and interactions between the ligand and the receptor.

### In vivo anti-tumor assay

Female Balb/c nude mice at the age of 3 months and ICR mice at 2 months were purchased from Shanghai JIHUI Laboratory Animal Co Ltd. (China). Human 143B xenografts were created by subcutaneously injecting 5 ×10^6 cells into female BALB/c nude mice. Once the tumors reached an average volume of 50 mm^3, the mice were randomly divided into control and treatment groups for subsequent interventions. Compound (*S*,*R*)-**4v** was administered via intraperitoneal injection at a dosage of 30 mg/kg in a solution composed of 5% DMSO, 45% PEG400, and 50% water, once daily for a duration of 23 days. Throughout the experiment, the body weight and tumor volume of the mice were measured every 2 days. Tumor volume was calculated using the formula (A x B^2^)/2, where A represents the long diameter and B represents the short diameter. The experiment was terminated when the maximum tumor size reached 1000 mm^3^. At that point, the mice were humanely euthanized by cervical dislocation, and their tumors were removed and weighed. Tumor Growth Inhibition (TGI) Calculation: Tumor growth inhibition (TGI) was determined using the formula TGI = (1 - TW_treatment_ / TW_control_) x 100%, where TW_treatment_ represents the tumor weight in the treatment groups, and TW_control_ represents the tumor weight in the control group at the time of being sacrificed. Statistical analysis was carried out using GraphPad Prism 8.0 software, and the significance of differences in tumor volume and body weight on the final day was assessed using a one-way ANOVA model followed by Tukey’s multiple comparisons test.

### Statistical analysis

Data are presented as the mean ± SD (vertical error bars) from three independent experiments was considered statistically significant. The t tests were used to compare the levels of numerical variables between three experimental groups, and no data points in our study were excluded. Statistical analyses were performed using GraphPad Prism 9. Analysis with p value < 0.05 was considered statistically significant. Statistical significance differences defined as *P < 0.05, **P < 0.01, ***P < 0.001 and ****P < 0.0001.

## Supplementary information

western blots

Supplementary information

## Data Availability

All data will be available upon reasonable request.
