# Supplementary figures and images for: Identification of Nogo-B as a potential therapeutic target of osteosarcoma via stereochemically selective covalent probes
Source: Cell Death Dis. 2025 Jul 19;16(1):537. doi: 10.1038/s41419-025-07765-z (PMC12276343; doi:10.1038/s41419-025-07765-z)

**Figure 3 a-d**


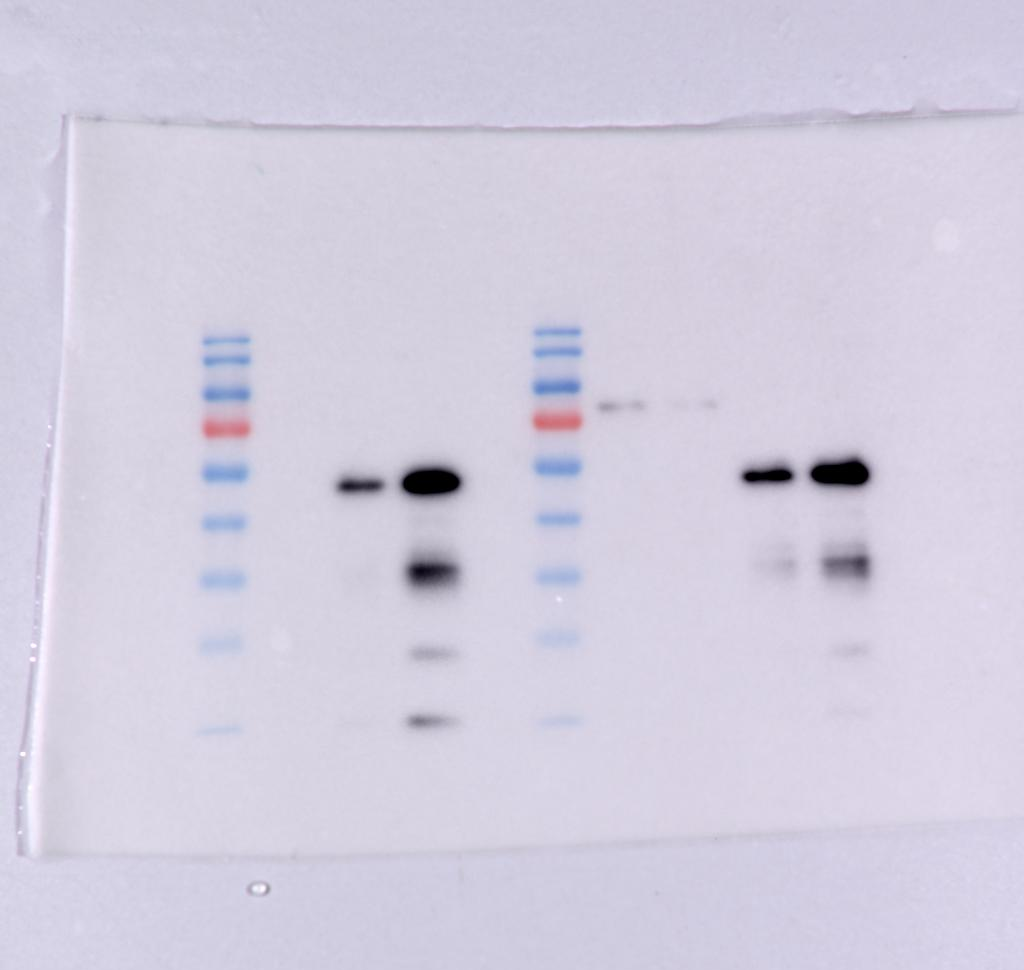

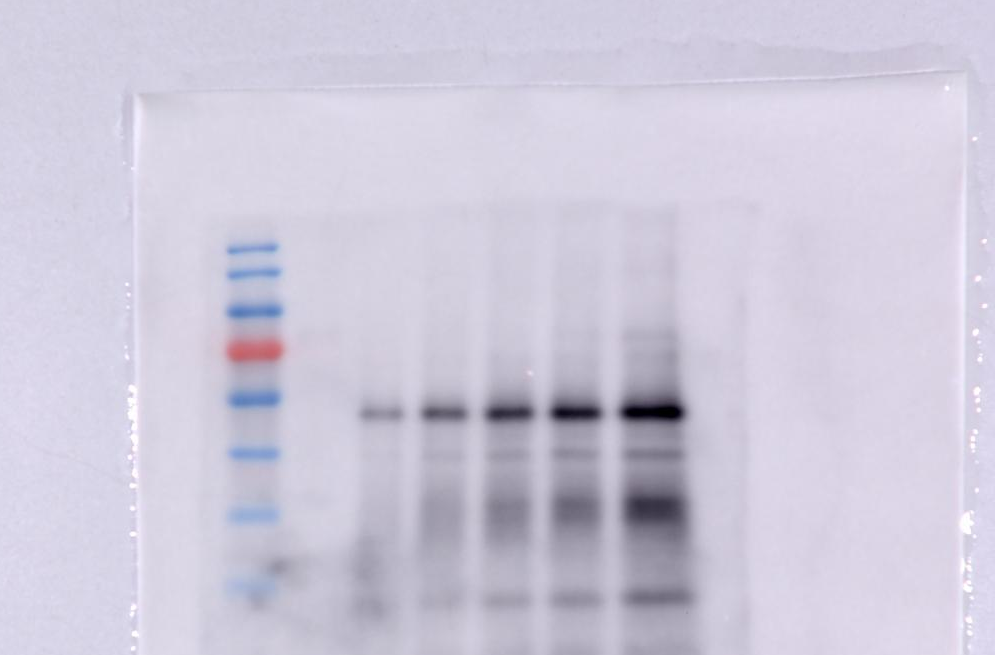


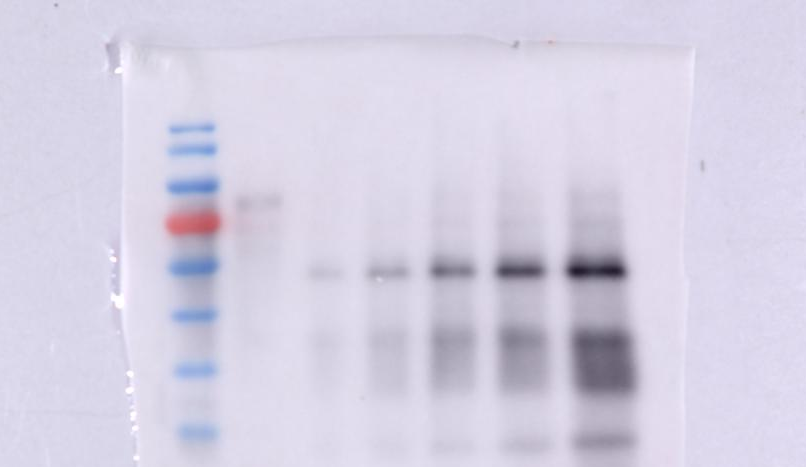

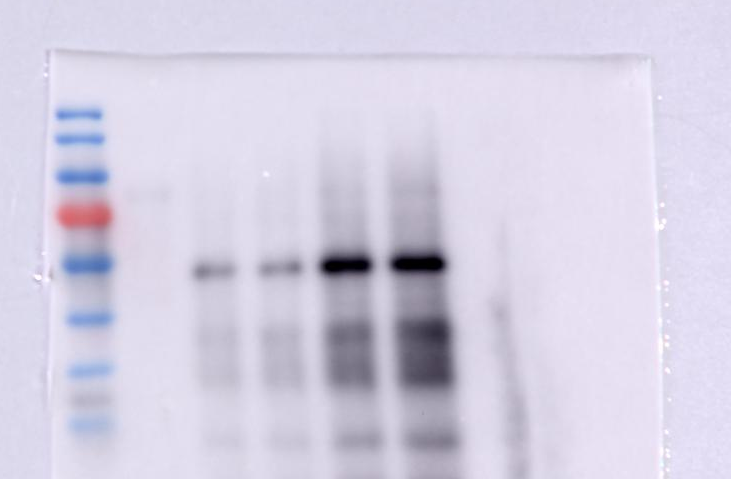


**Figure 4 c**


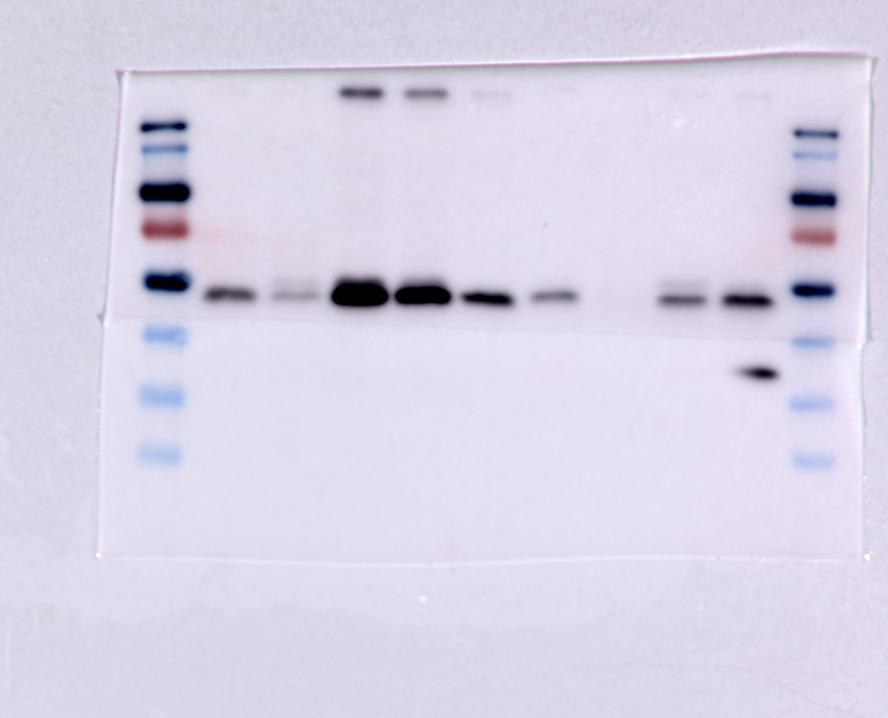

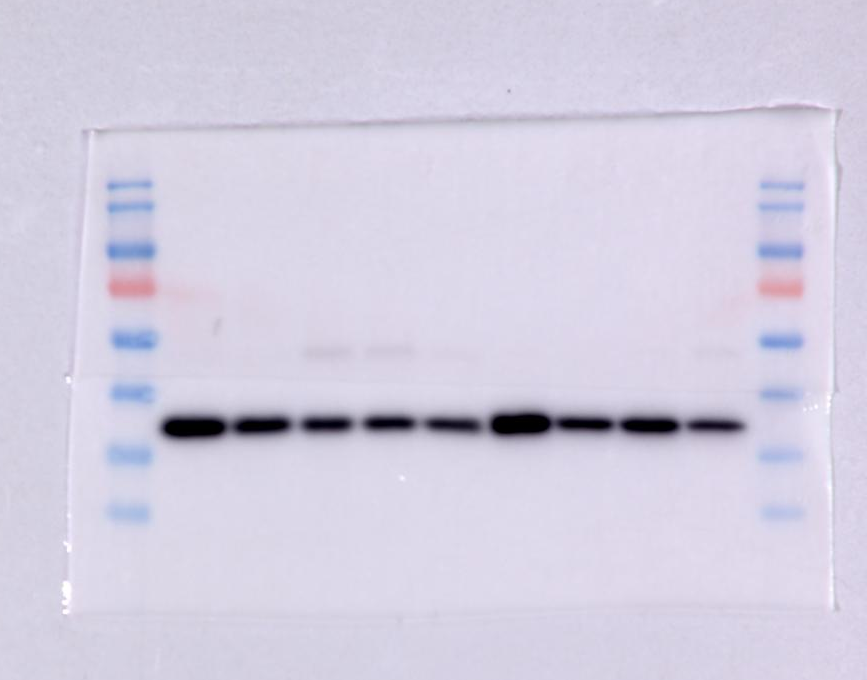


**Figure 4 e**


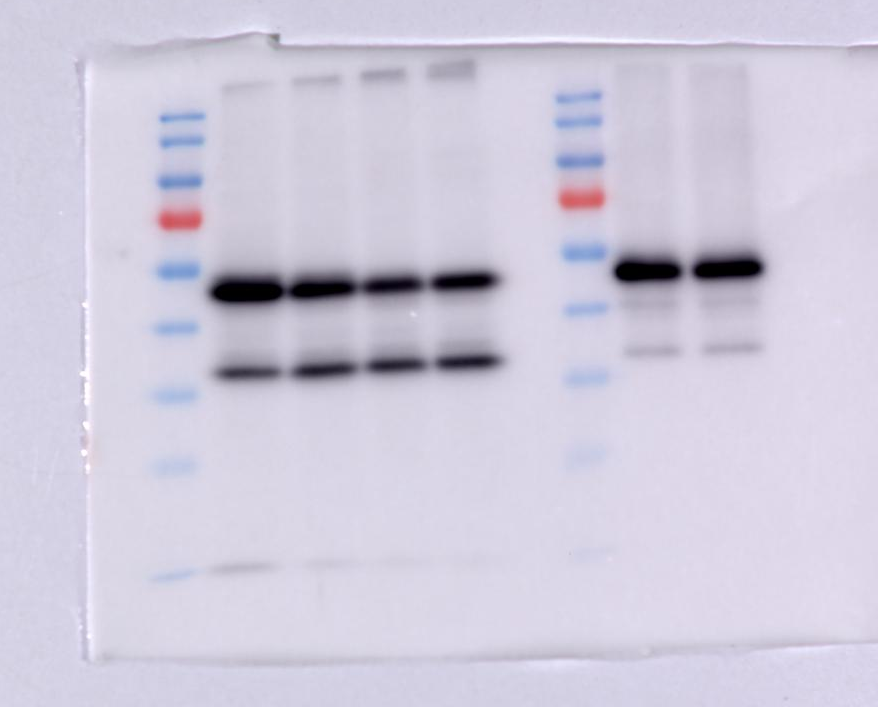

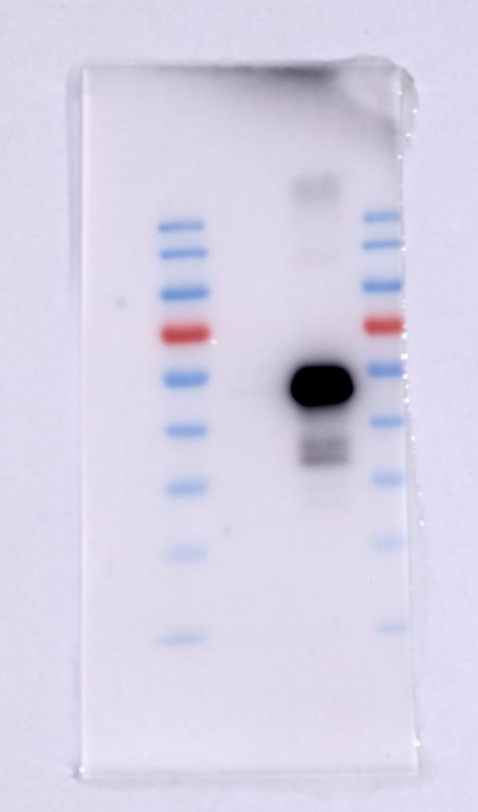


**Figure 4 f**


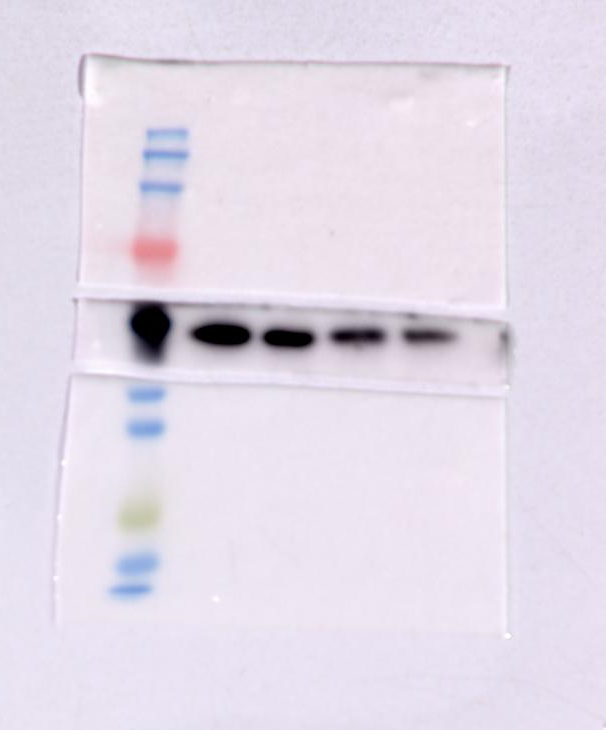

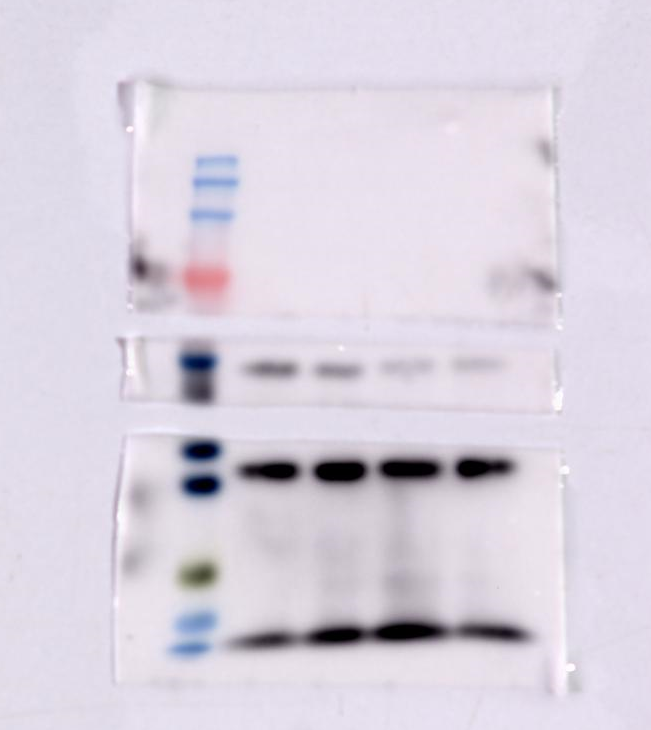


**Figure 4 g**


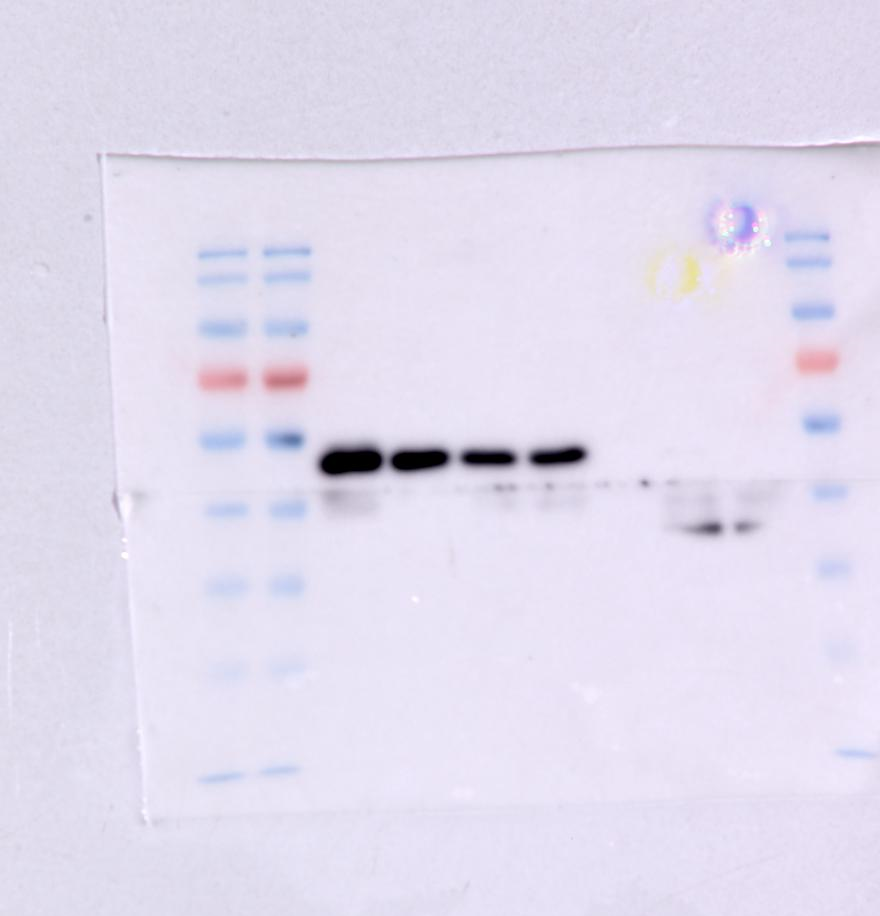

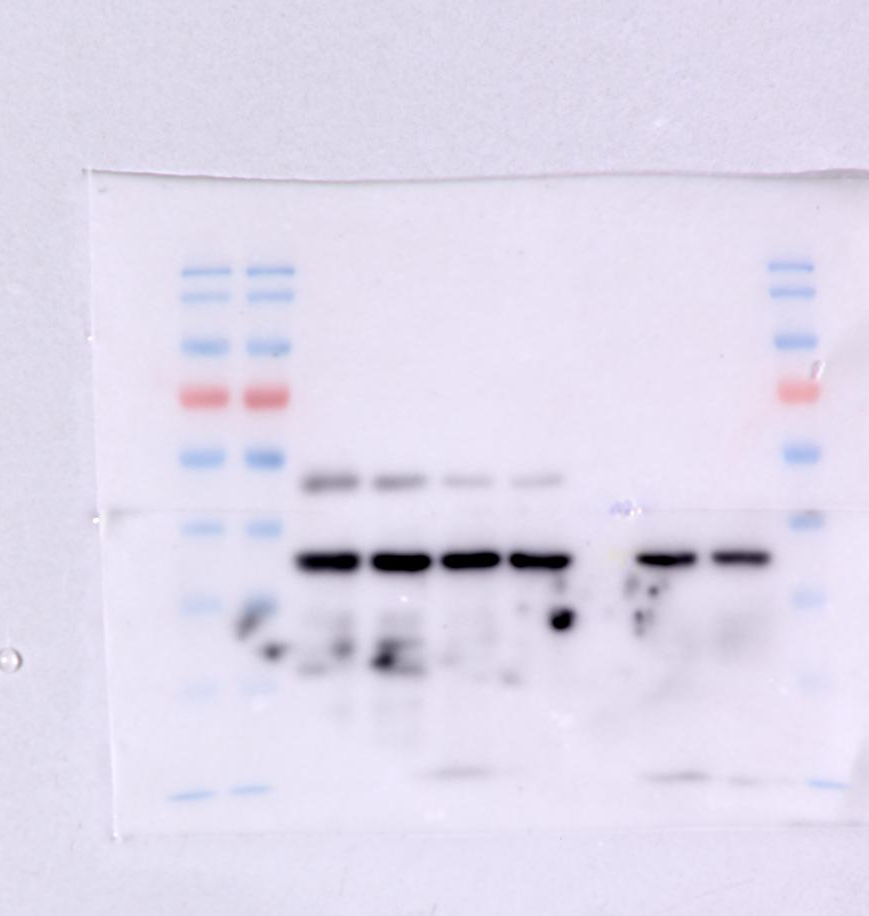


**Figure 4 i**


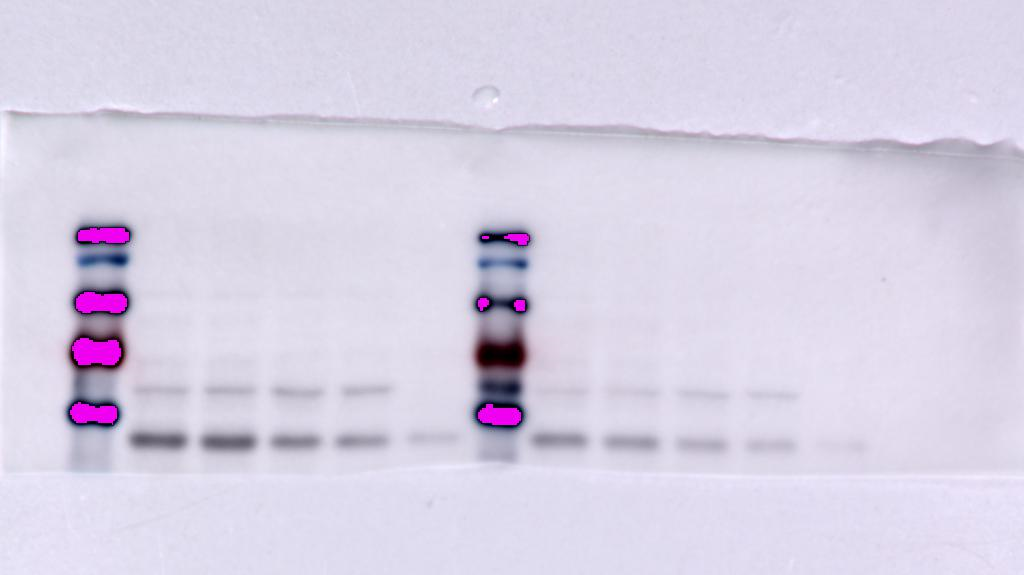

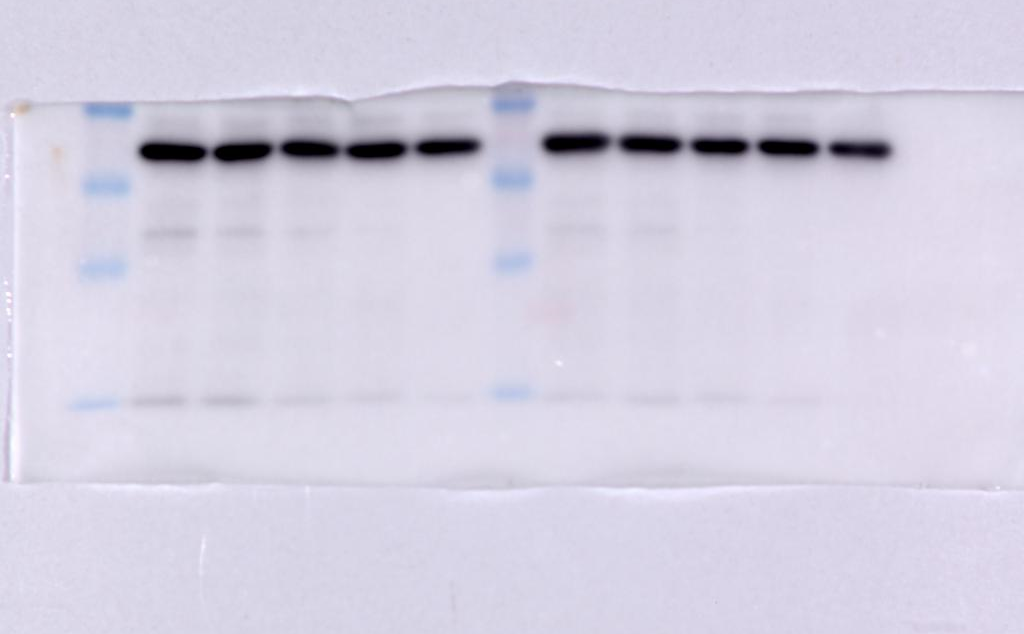


**Figure 5 a**


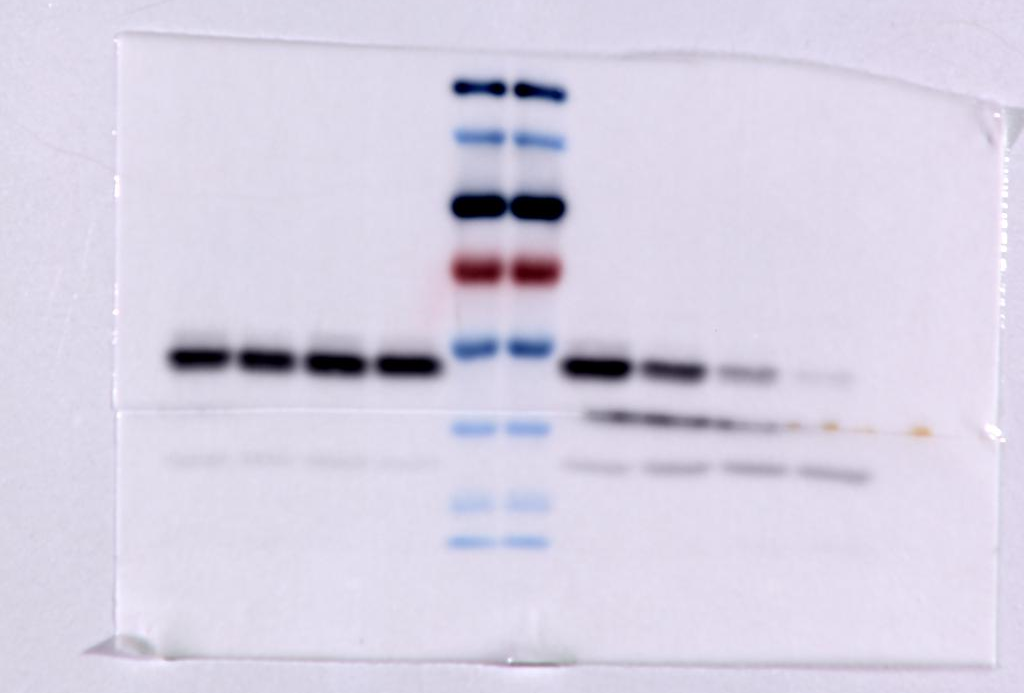

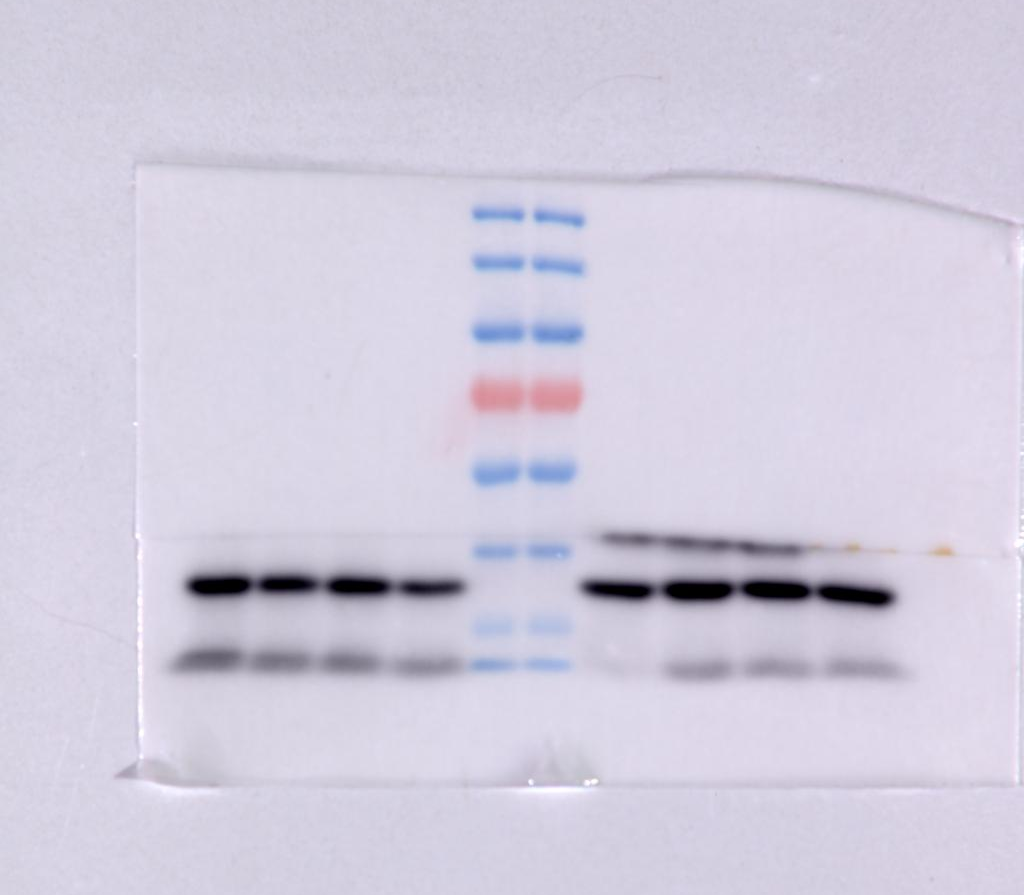


**Figure 5 b**


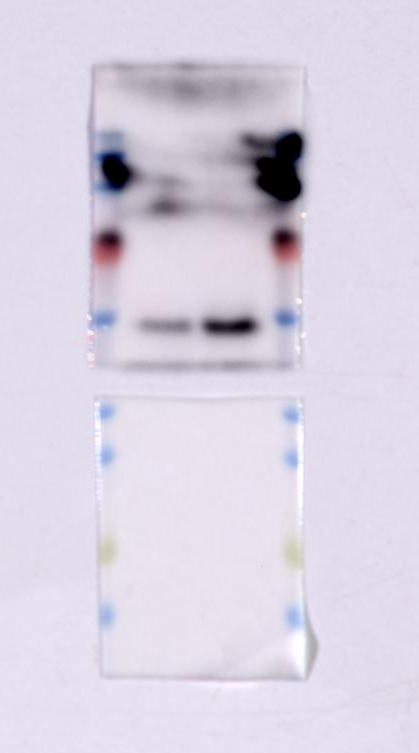

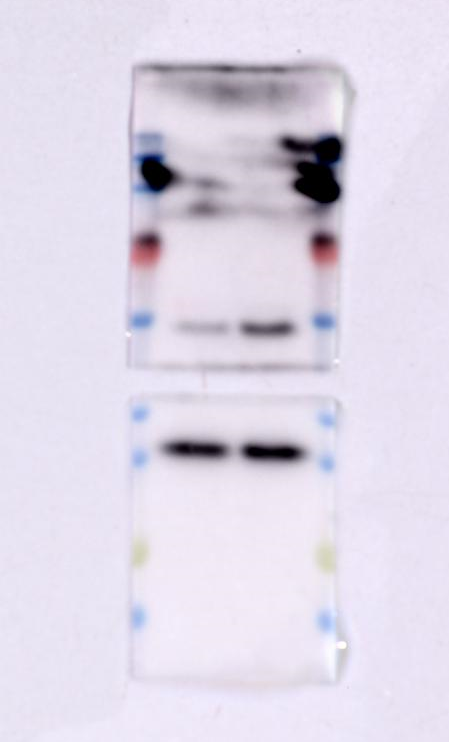


**Figure 5 m**


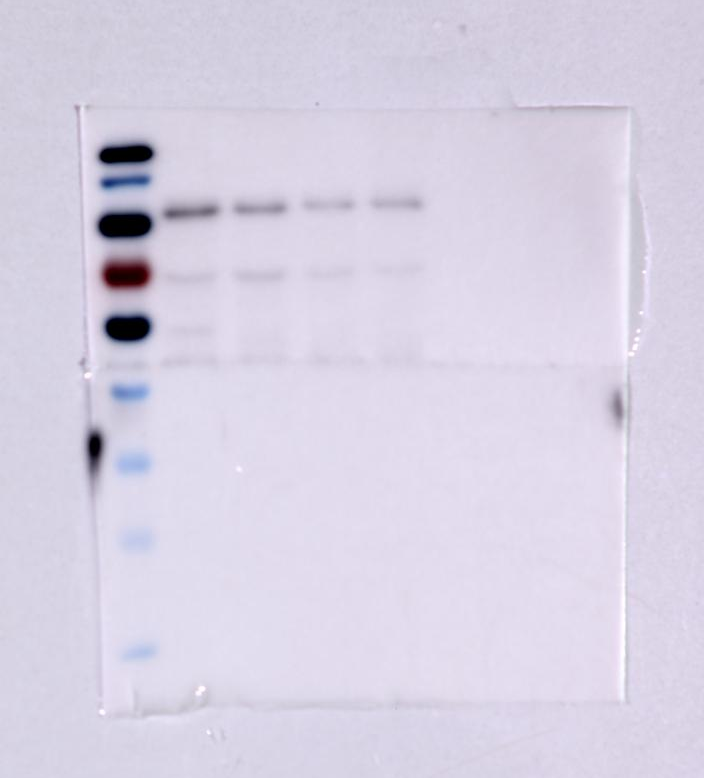

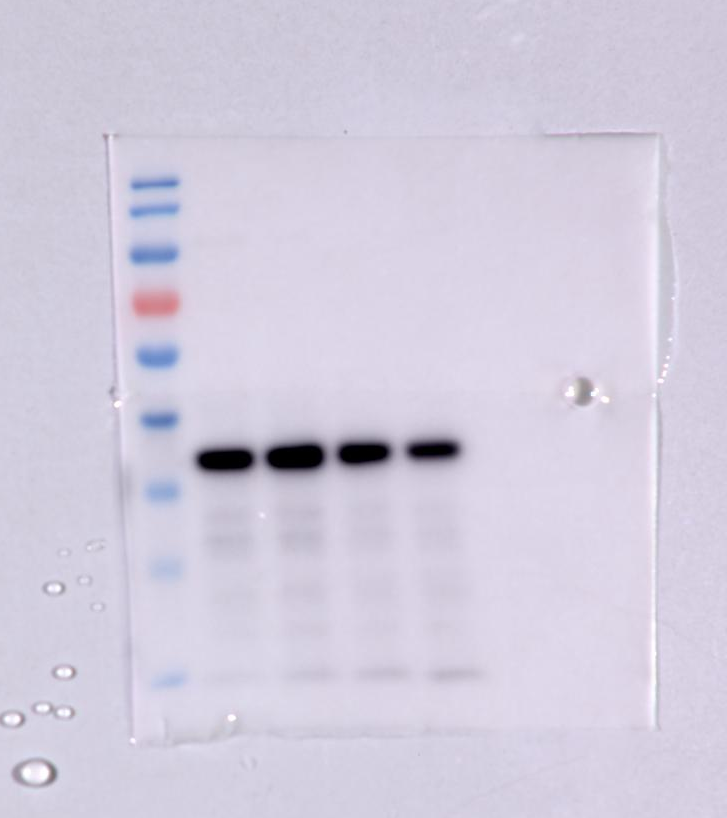


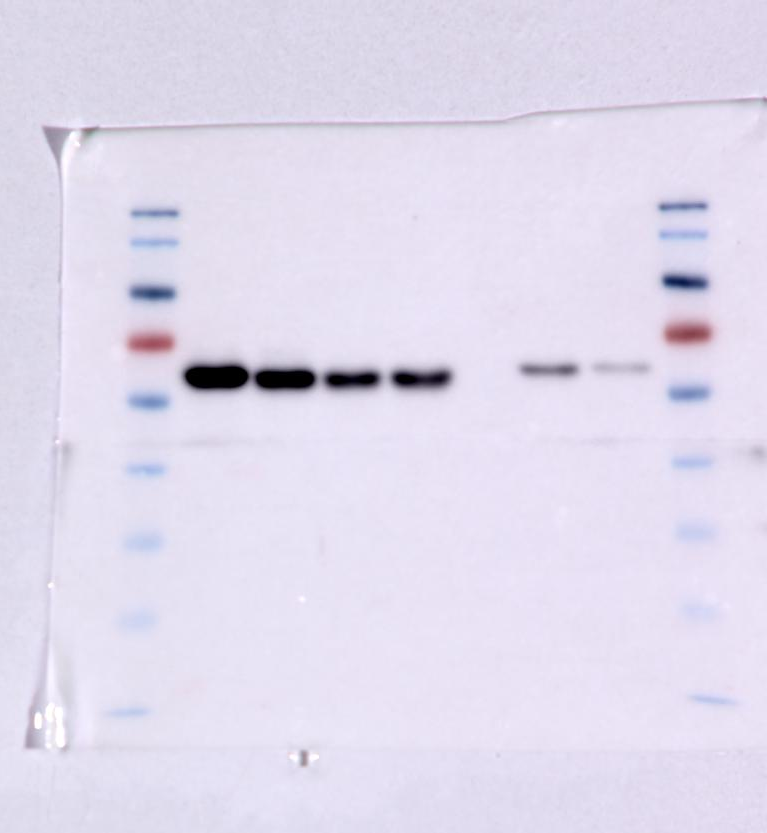

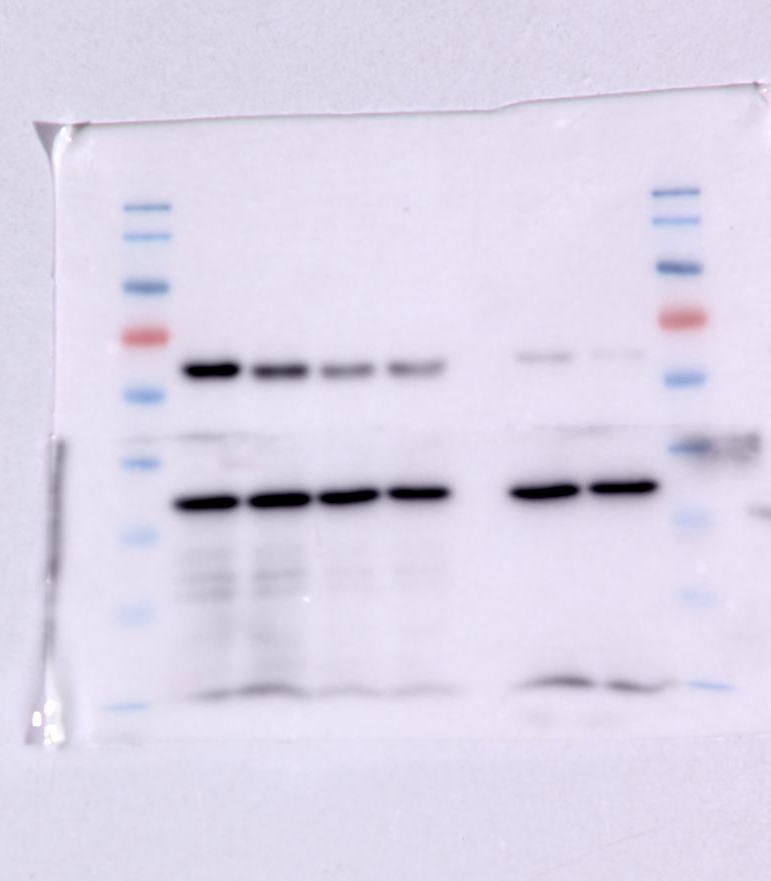


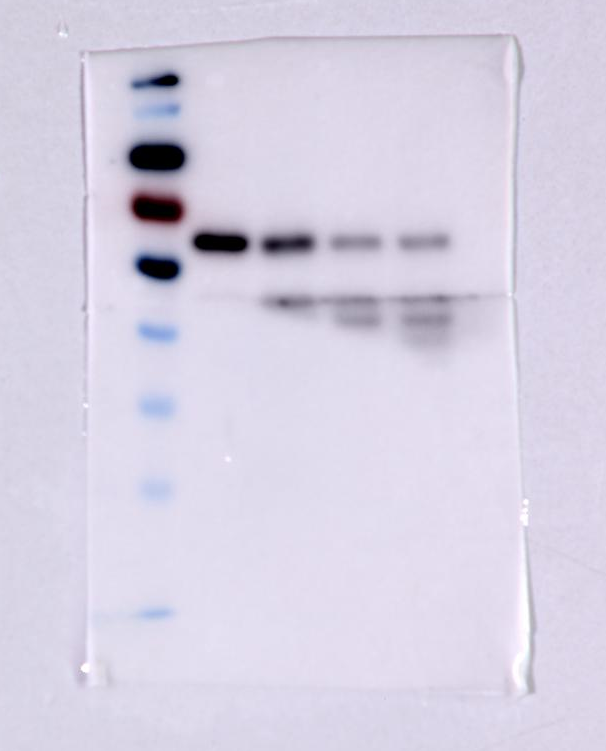

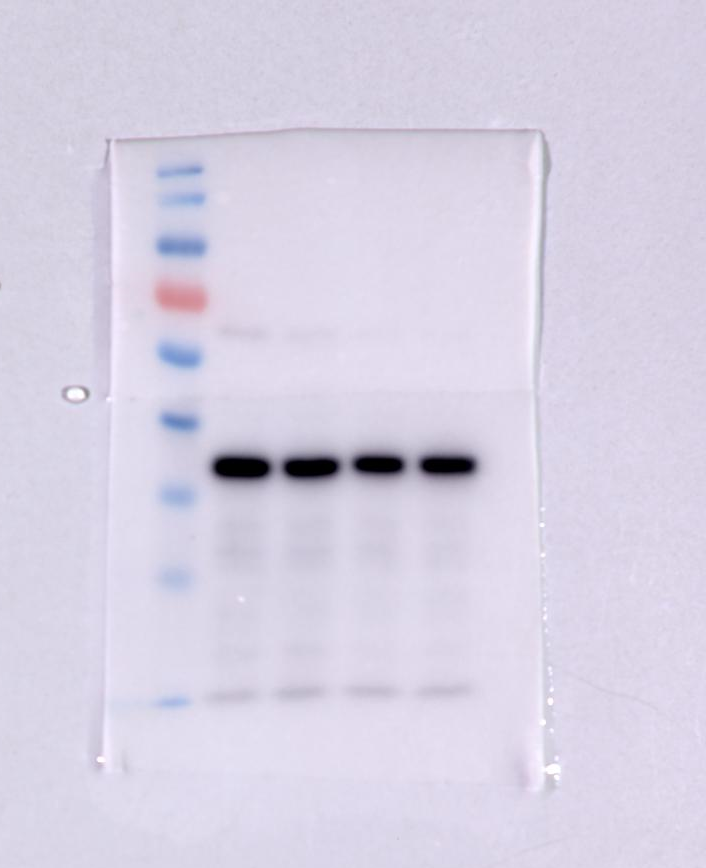


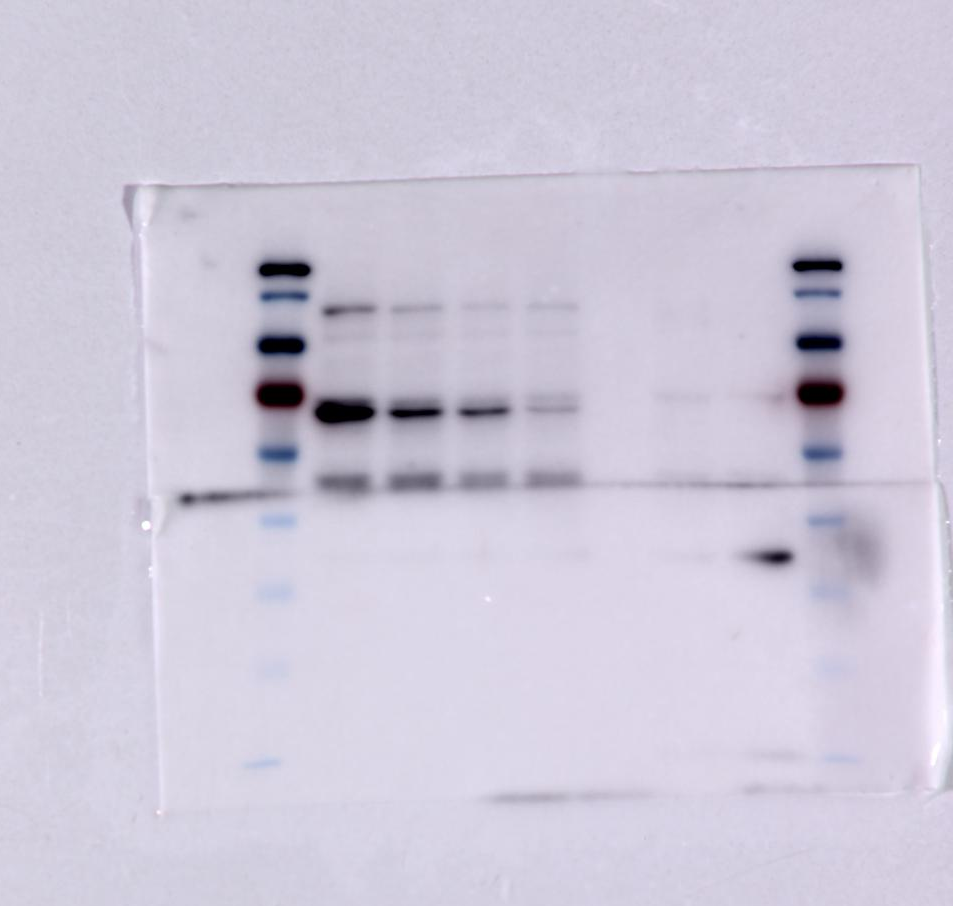

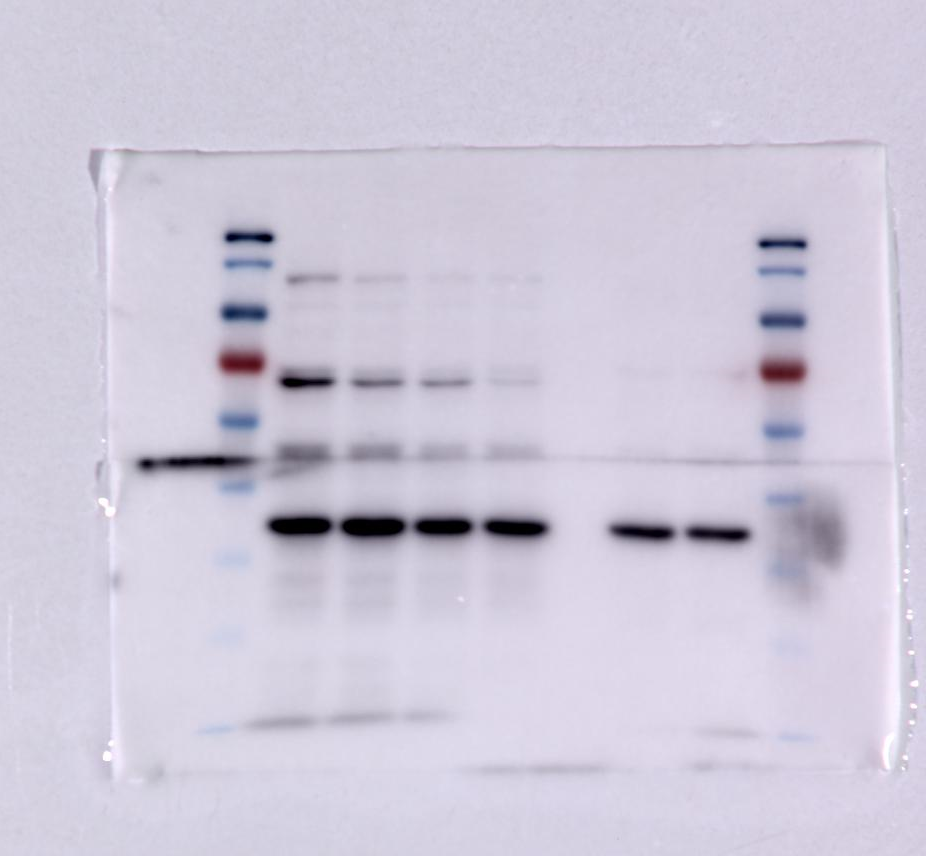


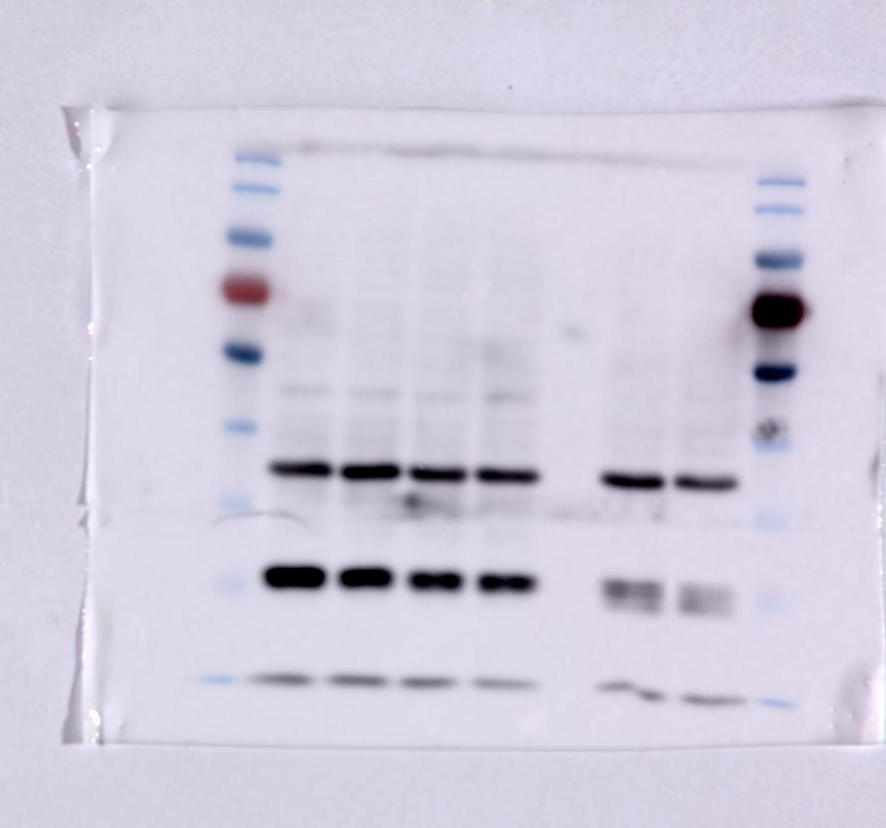


**Figure 5 C**


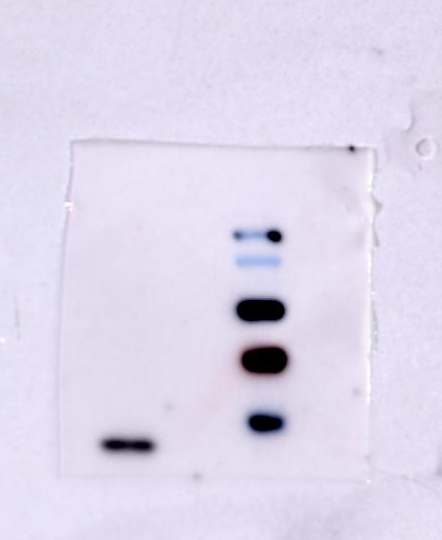

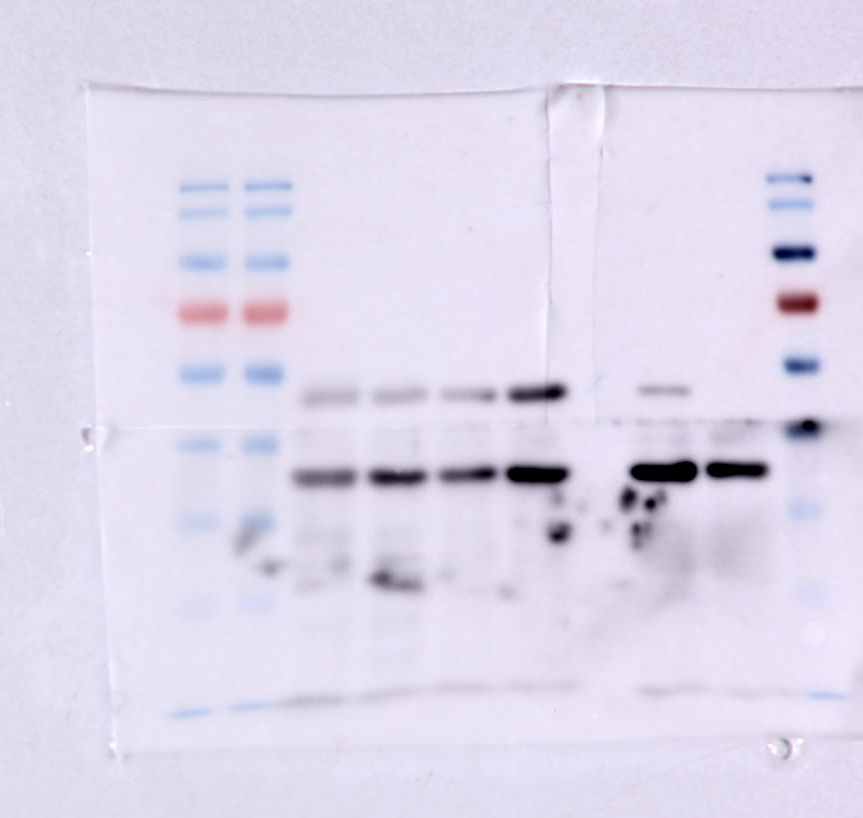


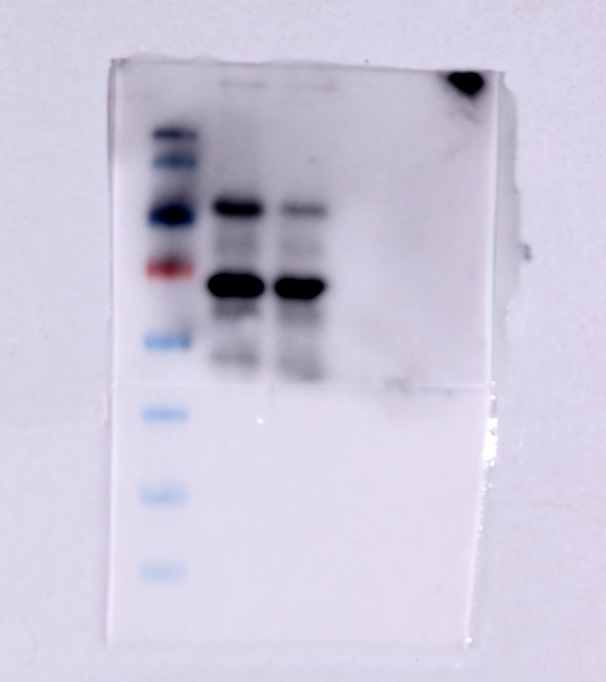

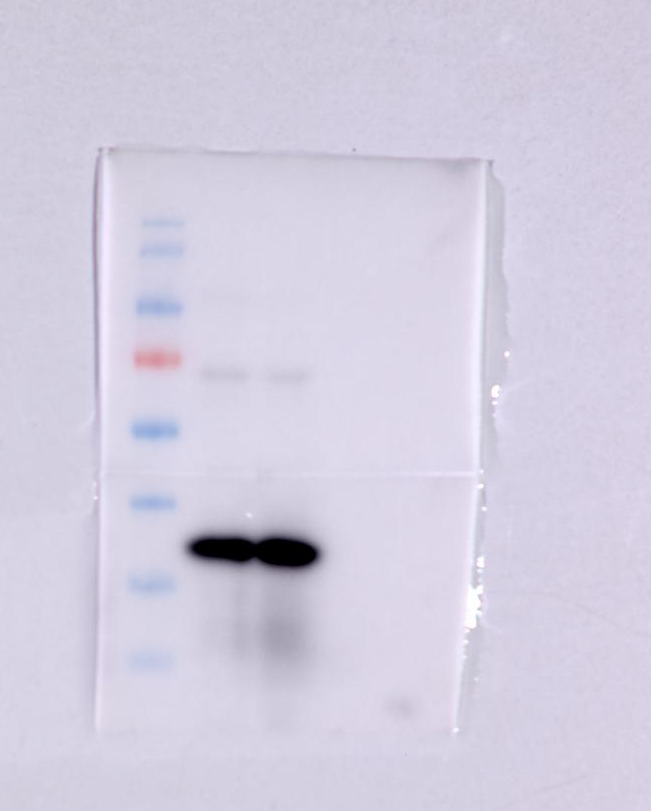


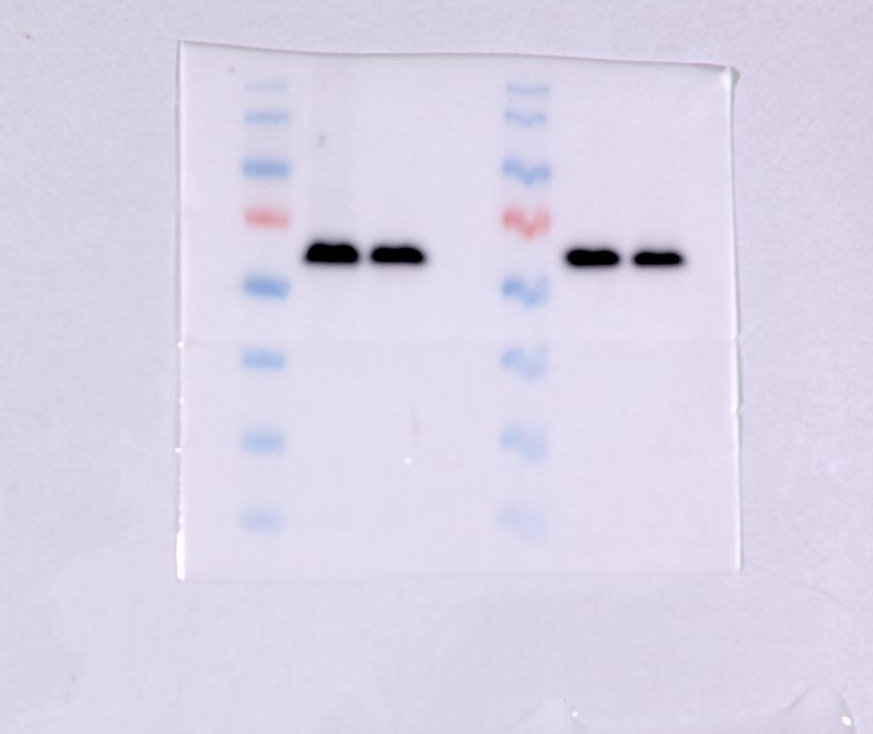

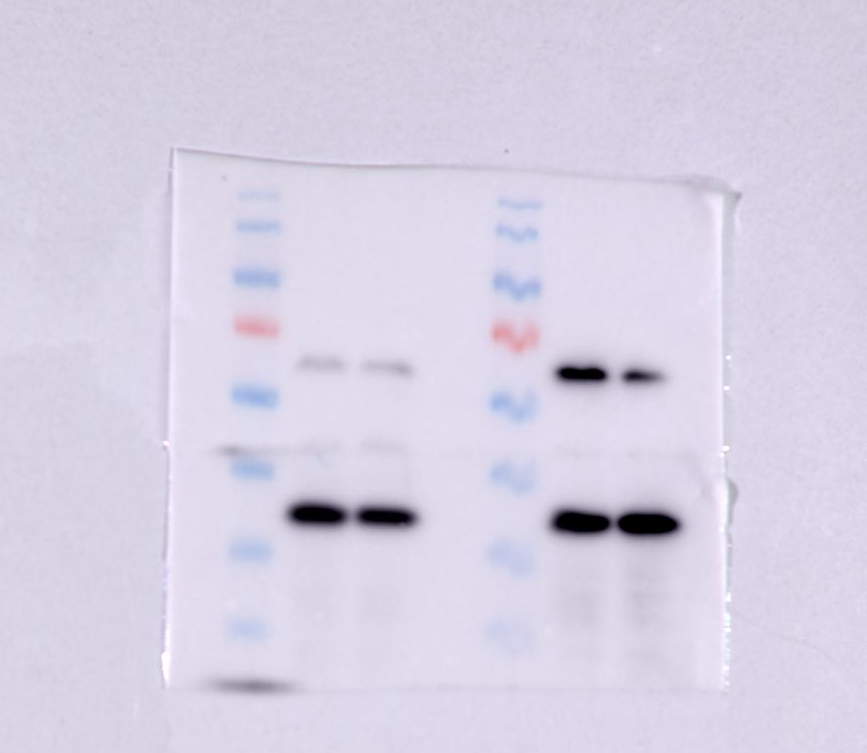


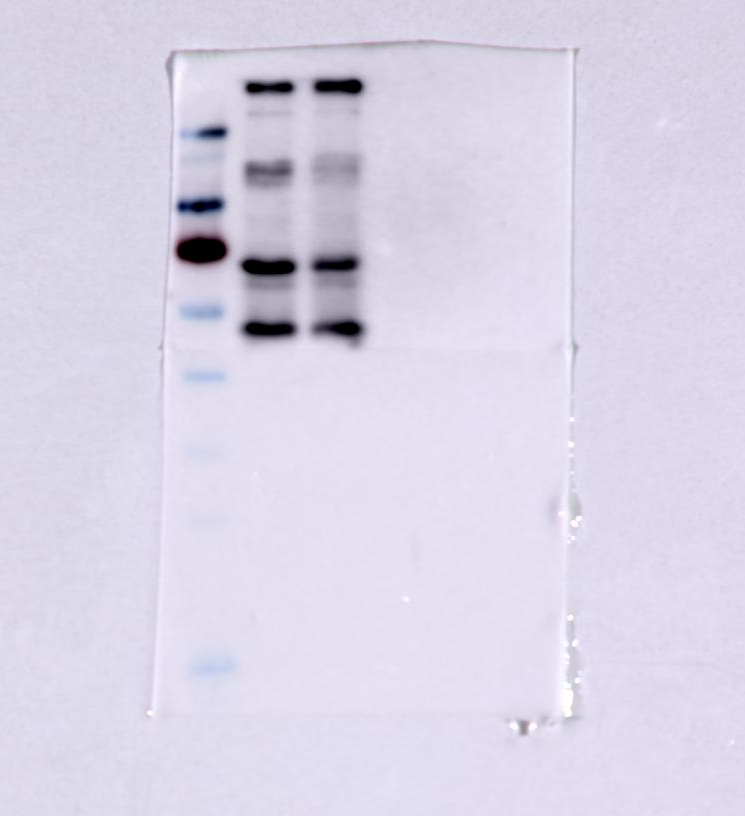

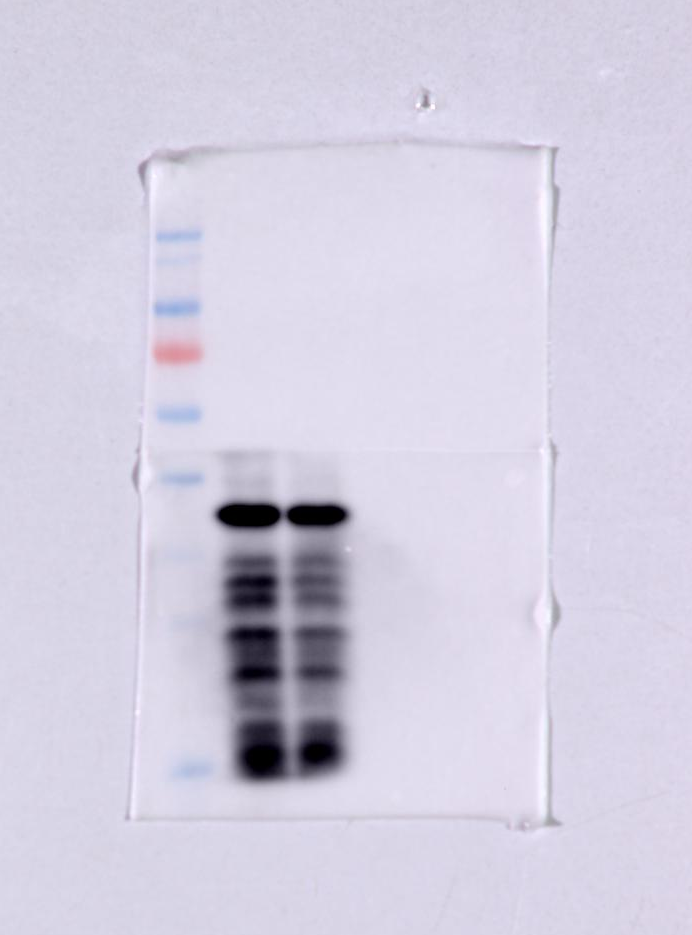


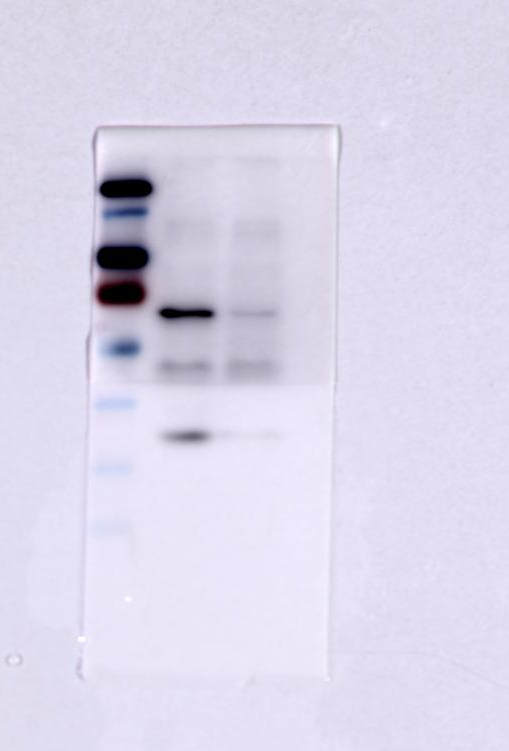

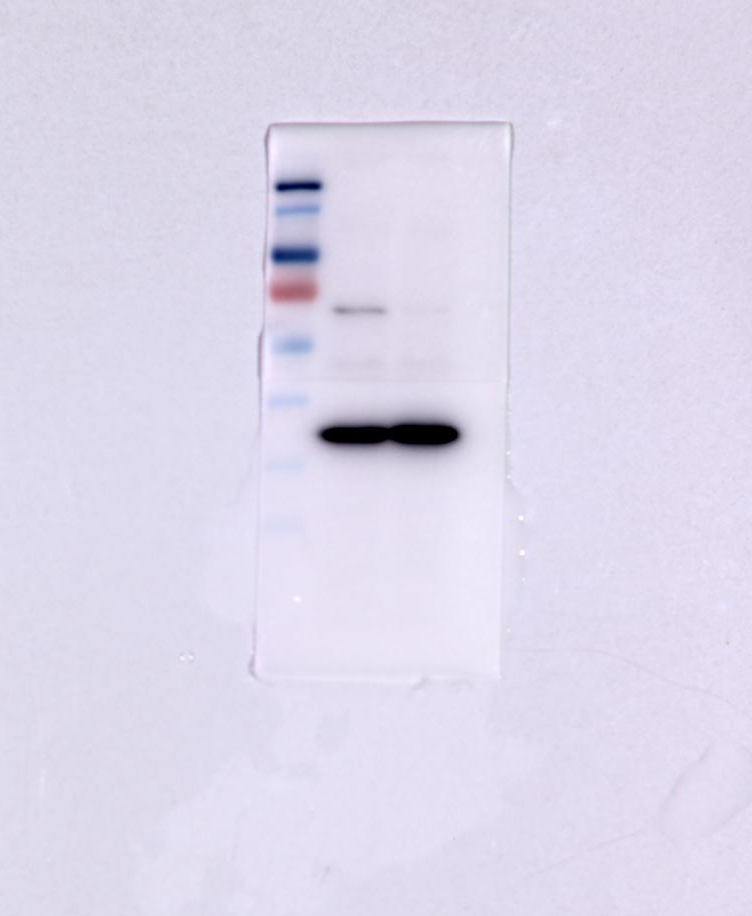


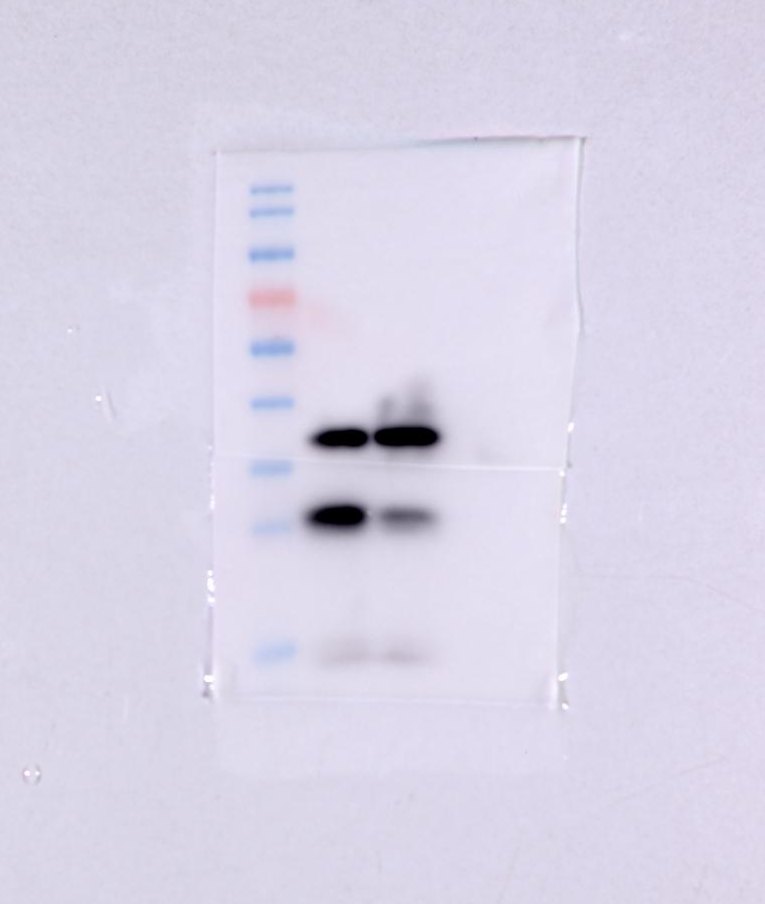


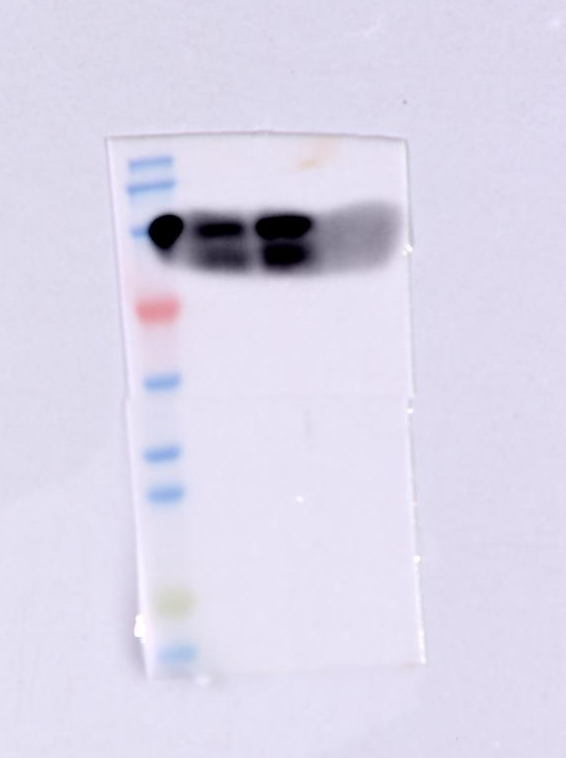

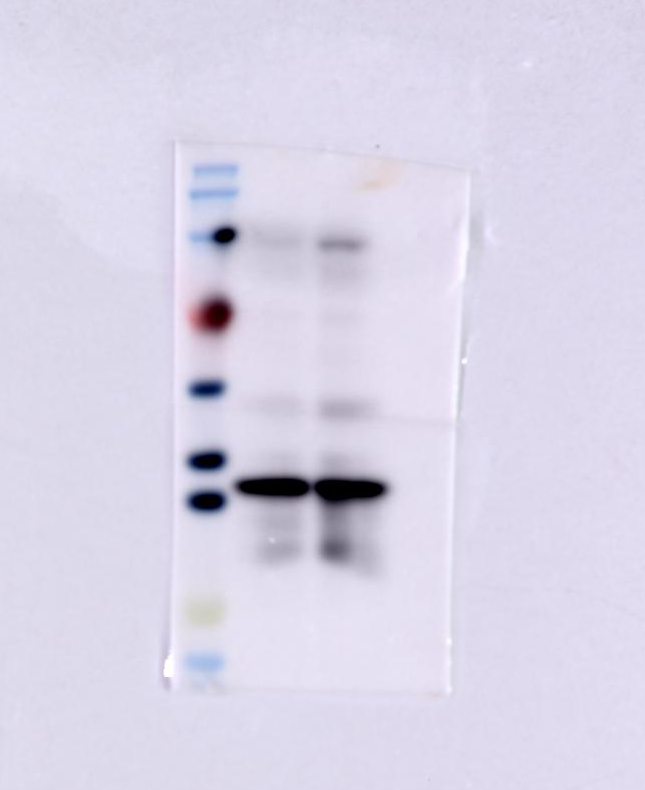


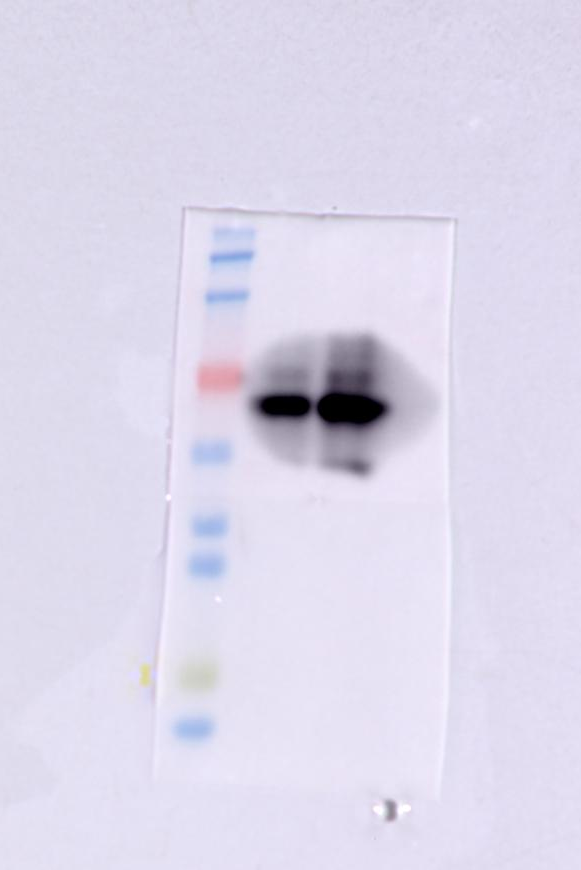

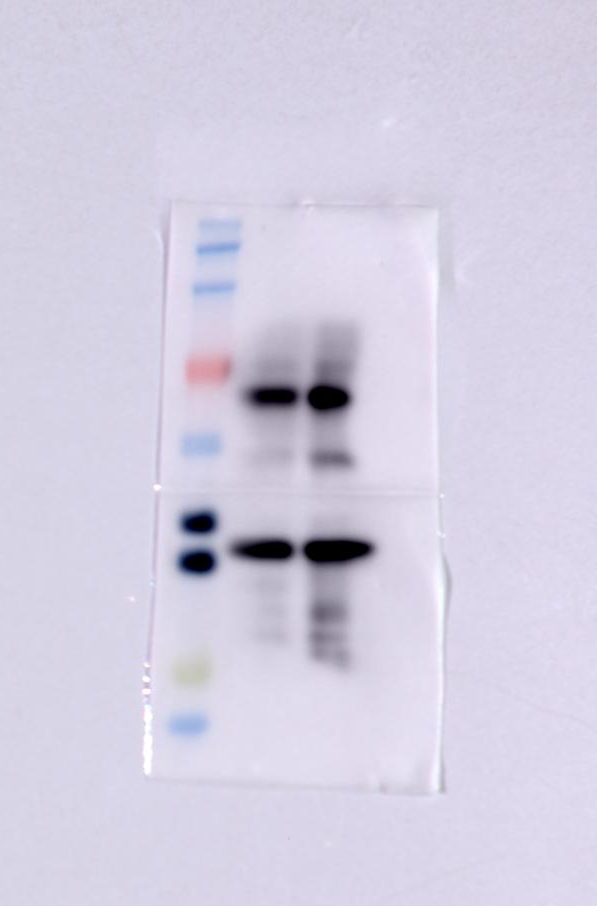


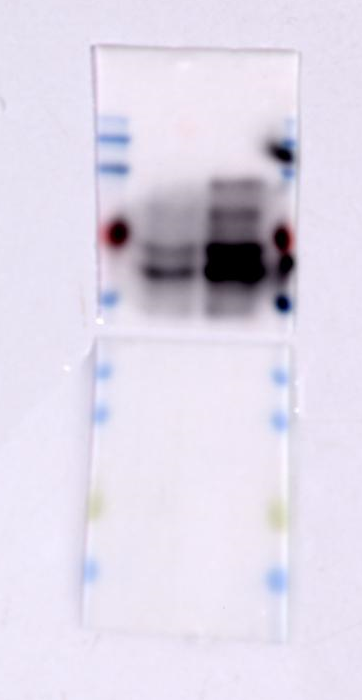

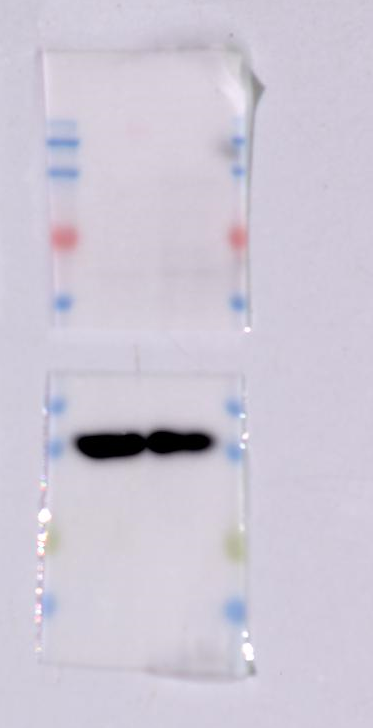


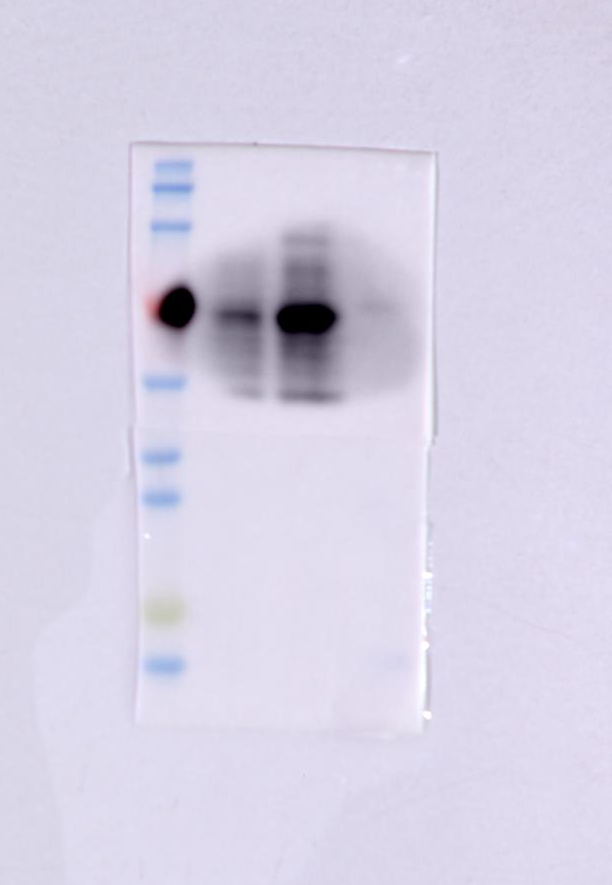

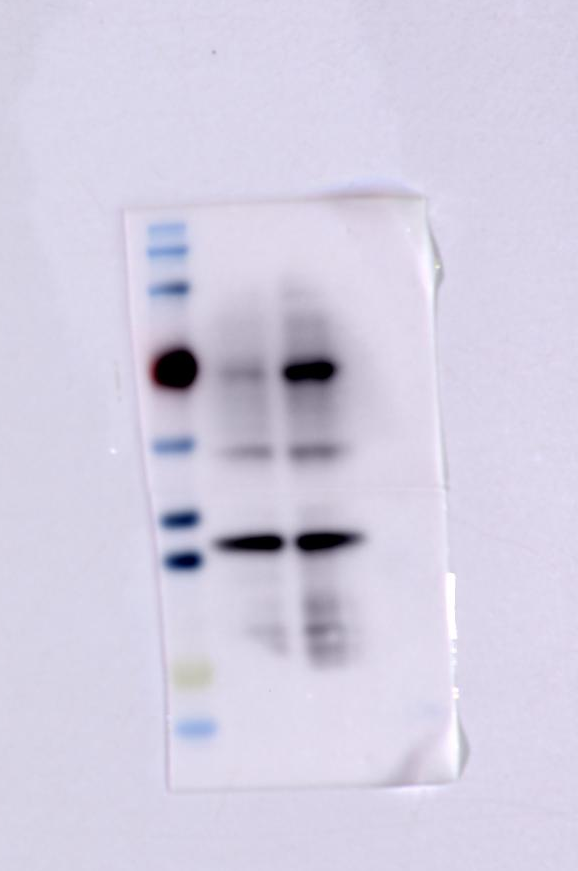


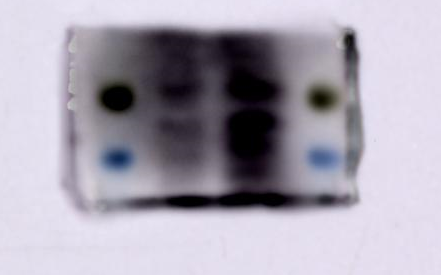

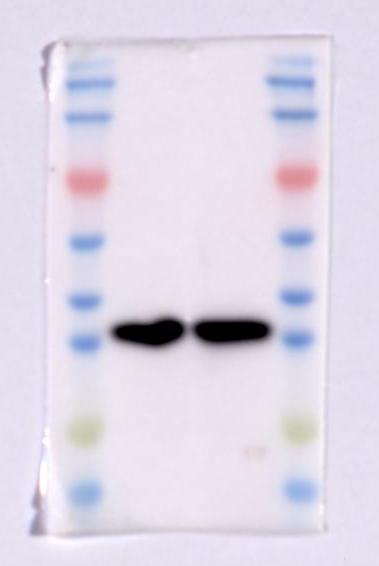

Supplement: Supplementary file 1 — western blots [file 41419_2025_7765_MOESM1_ESM.docx]
