# Supplementary material for: Identification of Nogo-B as a potential therapeutic target of osteosarcoma via stereochemically selective covalent probes
Source: Cell Death Dis. 2025 Jul 19;16(1):537. doi: 10.1038/s41419-025-07765-z (PMC12276343; doi:10.1038/s41419-025-07765-z)
Supplement: Supplementary file 2 — Supplementary information [file 41419_2025_7765_MOESM2_ESM.pdf]

Supplementary Materials for

Identification of Nogo-B as a Potential Therapeutic Target of Osteosarcoma via Stereochemically Selective Covalent Probes

Jian Xue, Meng Li, Li Kang, Meiting Wang, Jiabin Yin, Donghui Sun, Yaqi Deng, Qinghua Wei, Jiemin Wong, Tong Zhu & Shunying Liu*

Correspondence to: syliu@sist.ecnu.edu.cn

This PDF file includes:

Figures S1 to S6
Tables S1 to S3
Characterization Data
NMR Spectra

General Information

All ^{1}H NMR (400 MHz) and ^{13}C NMR (101 MHz) spectra. Tetramethylsilane (TMS) served as an internal standard (δ = 0) for ^{1}H NMR, and $CDCl_3$ was used as internal standard (δ = 77.0) for ^{13}C NMR. Chemical shifts are reported in parts per million as follows: chemical shift, multiplicity (s =singlet, d = doublet, t = triplet, q = quartet, m = multiplet, br = broad). High-resolution mass spectrometry (HRMS) was performed on IonSpec FT-ICR or Waters Micromass Q-TOF micro Synapt High Definition Mass Spectrometer.

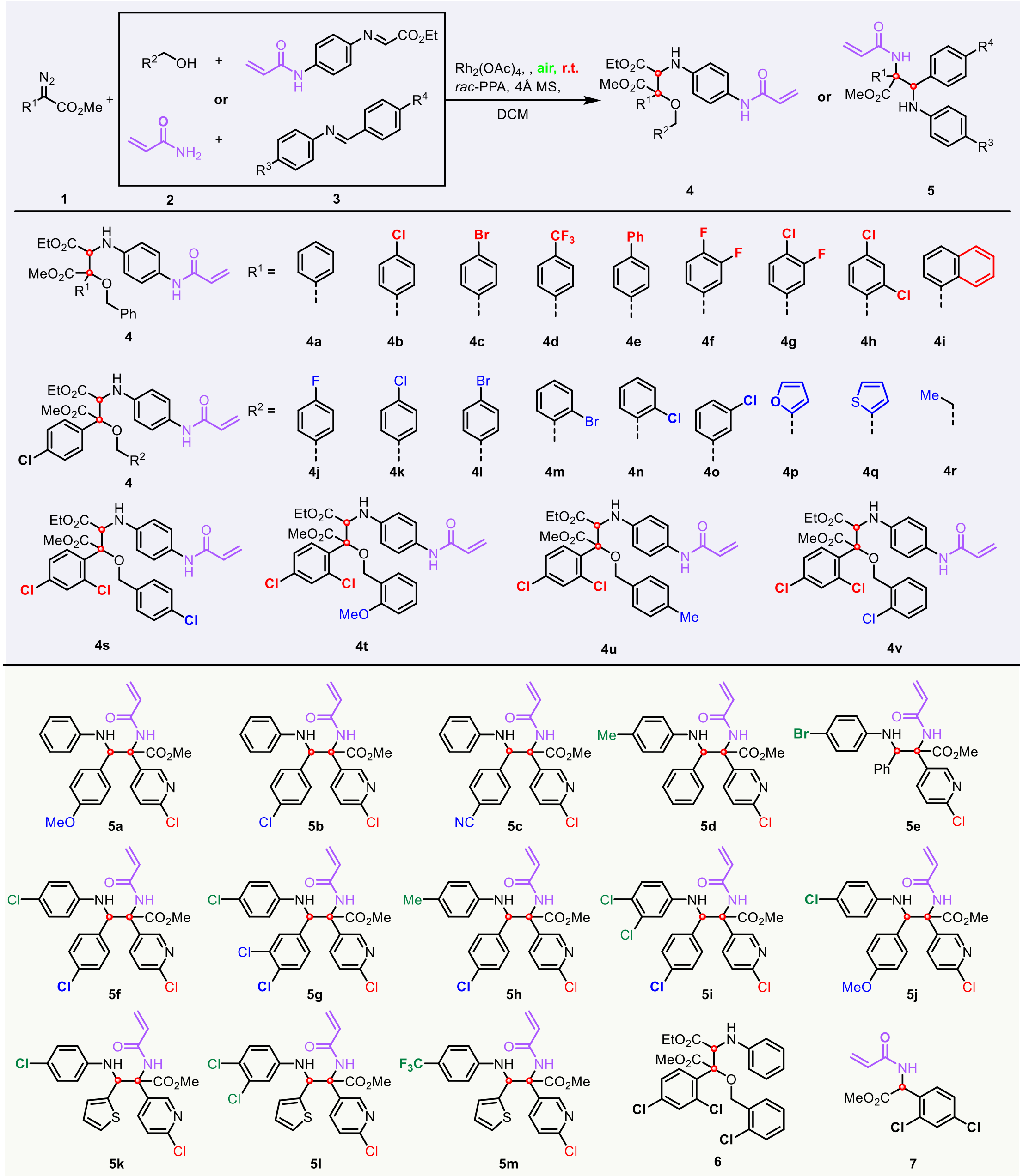

Figure S1. Chemical structure of small molecule in molecular library. Conditions: Unless otherwise noted, all reactions were conducted on a 0.2 mmol scale of **3**, **1**:**2**:**3** = 1.5: 1.2: 1. DCM: dichloromethane; *rac*-PA: racemic phosphoric acid; 4Å MS: 4Å molecular sieve.

a

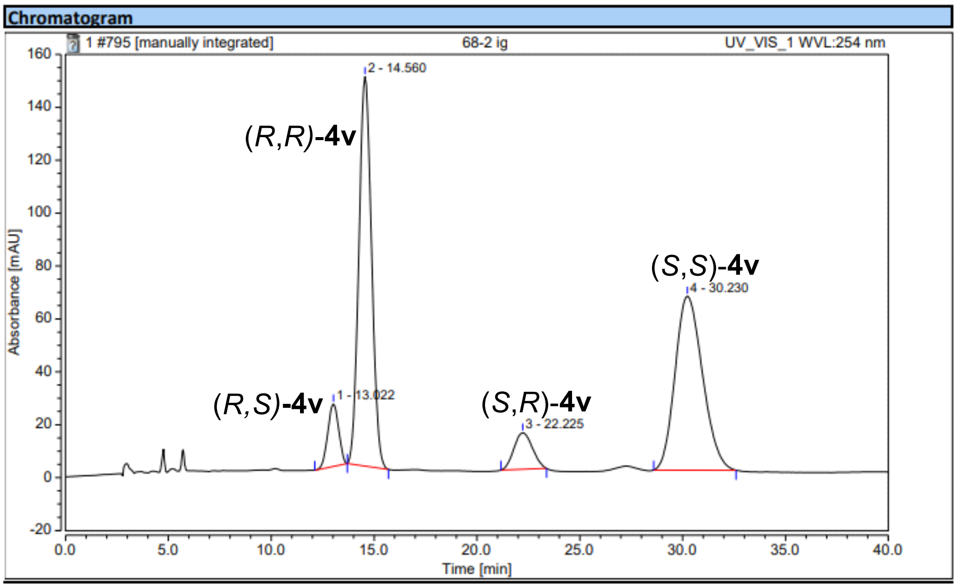

Integration Results

No.	Peak Name	Retention Time min	Area mAU*min	Height mAU	Relative Area %	Relative Height %	Amount n.a.
1		13.022	14.398	23.565	6.11	9.41	n.a.
2		14.560	104.937	147.218	44.55	58.80	n.a.
3		22.225	14.451	13.783	6.13	5.51	n.a.
4		30.230	101.770	65.795	43.20	26.28	n.a.
**Total:**			**235.556**	**250.361**	**100.00**	**100.00**	

b

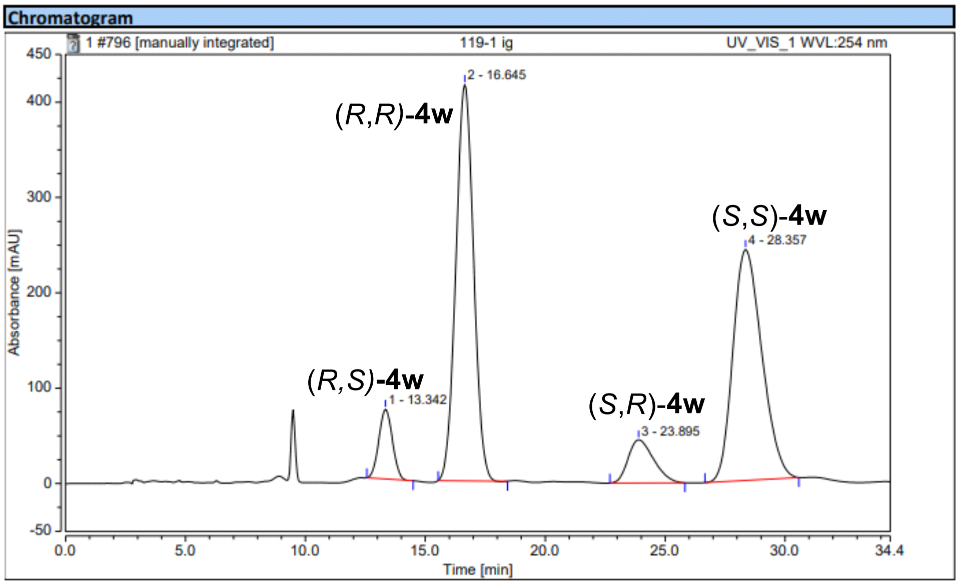

Integration Results

No.	Peak Name	Retention Time min	Area mAU*min	Height mAU	Relative Area %	Relative Height %	Amount n.a.
1		13.342	47.770	72.931	5.99	9.40	n.a.
2		16.645	355.668	415.676	44.58	53.57	n.a.
3		23.895	55.699	45.205	6.98	5.83	n.a.
4		28.357	338.764	242.173	42.46	31.21	n.a.
**Total:**			**797.900**	**775.986**	**100.00**	**100.00**	

Figure S2. **a** The four corresponding isomers of compound **4v** were isolated and obtained by HPLC (IC, hexane/isopropanol = 80/20, flow rate = 1.0 mL/min, λ = 254 nm) t_R = 13.022 min, 14.560 min, 22.225 min, 30.230 min. **b** The four corresponding isomers of compound **4w** were isolated and obtained by HPLC (IC, hexane/isopropanol = 80/20, flow rate = 1.0 mL/min, λ = 254 nm) t_R = 13.342 min, 16.645 min, 23.895 min, 28.375 min.

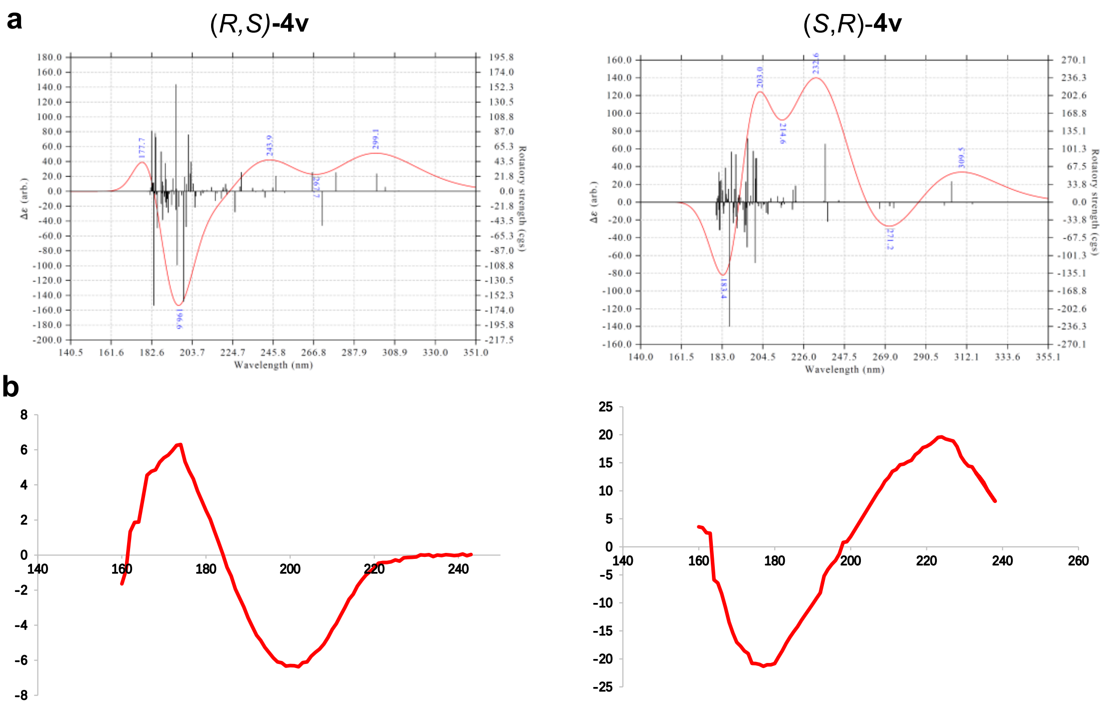

Figure S3. **a** The absolute configuration of the (*S*,*R*)-**4v** and (*R*,*S*)-**4v** was determined by the theoretical circular dichroism analysis. **b** The absolute configuration of the (*S*,*R*)-**4v** and (*R*,*S*)-**4v** was determined by the experimental analysis.

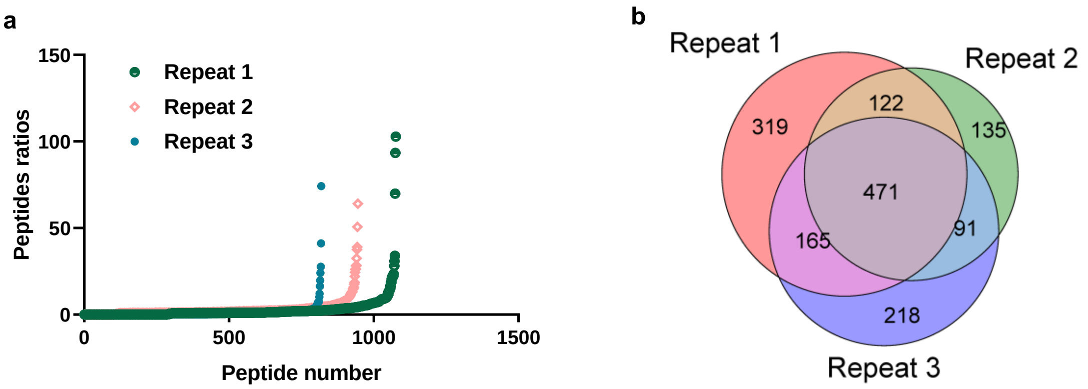

Figure S4. **a** The enrichment ratios of (*S*,*R*)-**4w**/DMSO in three independent experiment. **b** Venn diagram showing the number of high-confidence target sites of (*S*,*R*)-**4w** from three replicates.

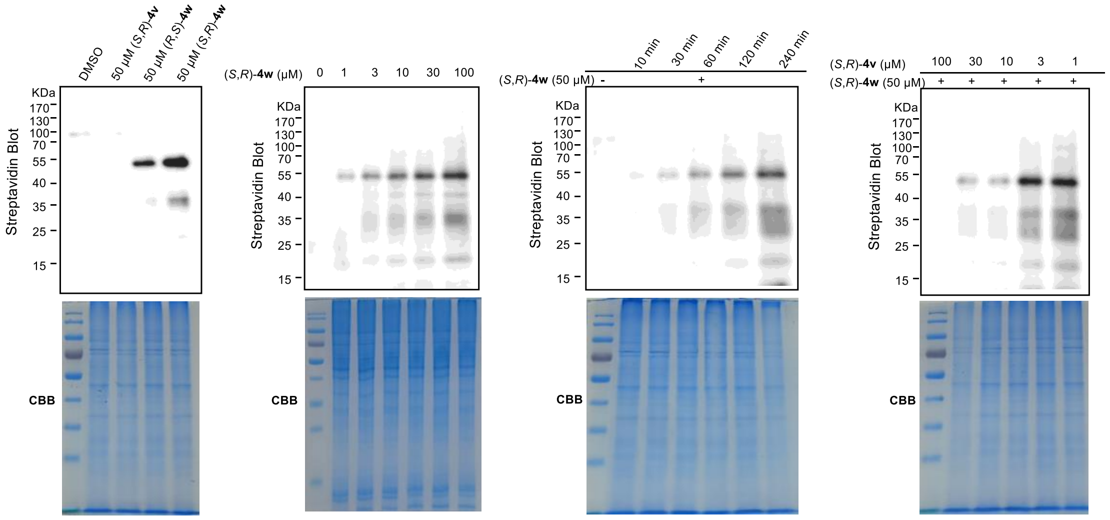

Figure S5. In situ labeling of 143B cells with DMSO, 50 μM (*S*,*R*)-**4v**, (*S*,*R*)-**4w** and (*R*,*S*)-**4w**, respectively; in situ labeling of 143B cells by (*S*,*R*)-**4w** at the concentrations of 0-100 μM; in situ labeling of 143B cells by (*S*,*R*)-**4w** at the times of 0-240 min; in situ labeling of 143B cells by 50 μM (*S*,*R*)-**4w** under the competition of inhibitor (*S*,*R*)-**4v** (0-100 μM).

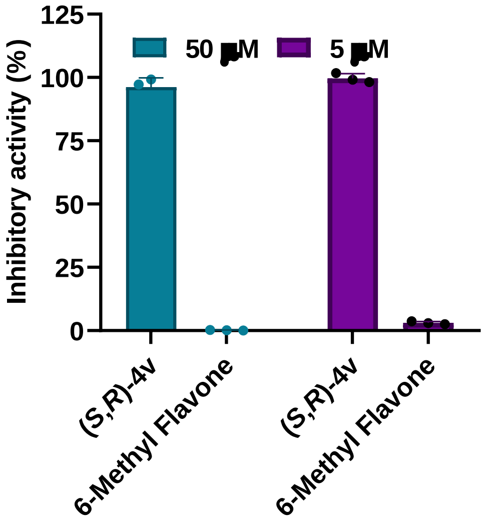

Figure S6. CCK-8 assays showed the inhibitory activity (%) of (*S*,*R*)-**4v** and 6-methyl flavone inhibitor in 143B cells.

Table S1 The list of top 20 potential target proteins of (*S*,*R*)-**4w**.

Number	Name	Enrichment ratio	Competition ratio
1	Q9NQC3	80.3	19.0
2	Q07065	61.7	6.7
3	Q00765	45.5	5.5
4	Q9HCC0	31.8	5.4
5	Q9H3N1	27.6	4.9
6	P45880	24.3	4.6
7	P30519	21.2	4.1
8	P07237	20.0	3.9
9	P16615	18.8	3.6
10	Q05639	17.9	3.5
11	P04259	17.1	3.5
12	P46379	15.9	3.4
13	P68363	15.4	3.2
14	Q56VL3	14.4	3.2
15	O43169	14.0	3.1
16	Q9NX40	13.0	3.1
17	P62280	12.5	3.1
18	P08621	11.5	3.0
19	Q8TC12	11.3	2.9
20	Q53EU6	10.8	2.6

Table. S2. The target sequences of shRNA.

Name	Sequence
shNogo-B1	CCTGTTATTTATGAACGGCAT
shNogo-B2	GCATATCTGGAATCTGAAGTT
shNogo-B3	GAAGTACAGTAATTCTGCTCT

Table S3. The list of indicated antibodies.

Antibody	Cat No.	Manufacturer
HRP-labeled Streptavidin	A0305	Beyotime
RTN4/Nogo-B	10950-1	Proteintech
PI3K	4263S	Cell Signaling Technology
AKT	4685S	Cell Signaling Technology
p-AKT	9271	Cell Signaling Technology
p65	AF0246	Beyotime
Bcl-2	AB112	Beyotime
GAPDH	AF0006	Beyotime
β-actin	4970S	Cell Signaling Technology
HRP-labeled Goat Anti-Rabbit IgG (H+L)	A0208	Beyotime
HRP-labeled Goat Anti-Mouse IgG (H+L)	A0216	Beyotime

Characterization Data of Compounds

4-ethyl 1-methyl 3-((4-acrylamidophenyl)amino)-2-(benzyloxy)-2-phenylsuccinate (4a)

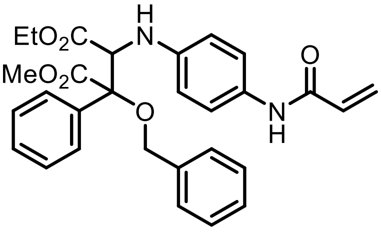

4a *syn/anti* isomer (2:1) ratio

White solid, 82 mg, 82% yield, R_f = 0.2 (PE/EA = 2/1), *syn*/*anti* isomer (2:1) ratio. Mixture of (*syn*/*anti*) isomers of **4a**: ^{1}H NMR (400 MHz, Chloroform-*d*) δ 7.50 (dd, *J* = 20.77, 3.89 Hz, 4H), 7.38 – 7.24 (m, 17H), 6.64 (d, *J* = 8.40 Hz, 1H), 6.50 (d, *J* = 8.41 Hz, 2H), 6.41 – 6.31 (m, 2H), 6.19 (d, *J* = 9.89 Hz, 2H), 5.68 (dd, *J* = 10.27, 3.07 Hz, 2H), 4.96 (d, *J* = 9.58 Hz, 2H), 4.86 – 4.73 (m, 2H), 4.52 (d, *J* = 11.79 Hz, 1H), 4.41 (d, *J* = 12.06 Hz, 1H), 4.33 (d, *J* = 9.68 Hz, 1H), 4.16 – 4.07 (m, 2H), 4.02 (m, 1H), 3.90 (s, 2H), 3.86 (s, 3H), 1.15 (t, *J* = 7.13 Hz, 3H), 1.07 (t, *J* = 7.09 Hz, 2H). ^{13}C NMR (101 MHz, $CDCl_3$) δ 171.4, 170.9, 170.6, 170.3, 163.3, 163.2, 143.9, 143.5, 138.5, 138.3, 134.5, 134.3, 131.2, 129.3, 128.9, 128.7, 128.4, 128.3, 128.2, 127.9, 127.5, 127.3, 127.2, 127.0, 126.9, 126.7, 121.7, 121.5, 114.5, 114.4, 86.7, 86.2, 67.9, 67.8, 65.2, 64.4, 61.7, 61.2, 52.6, 52.4, 13.9, 13.8. **HRMS(ESI)** Calcd. for $C_{29}H_{31}N_2O_6$ $(M+H)^+$ 503.2177, found 503.2179.

4-ethyl 1-methyl 3-((4-acrylamidophenyl)amino)-2-(benzyloxy)-2-(4-chlorophenyl)succinate (4b)

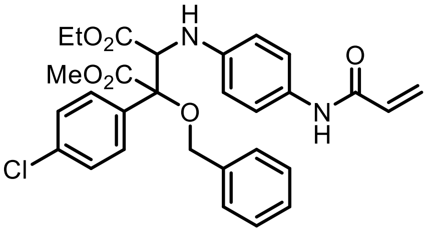

4b *syn/anti* isomer (2:1) ratio

White solid, 87 mg, 81% yield, R_f = 0.2 (PE/EA = 2/1), *syn*/*anti* isomer (2:1) ratio. Mixture of (*syn*/*anti*) isomers of **4b**: ^{1}H NMR (400 MHz, Chloroform-*d*) δ 7.62 (d, *J* = 8.08 Hz, 1H), 7.46 (dd, *J* = 13.74, 8.35 Hz, 3H), 7.31 (m, 6.45 Hz, 15H), 6.64 (d, *J* = 8.48 Hz, 1H), 6.50 (d, *J* = 8.43 Hz, 2H), 6.34 (dd, *J* = 16.85, 4.16 Hz, 2H), 6.19 (dd, *J* = 16.70, 10.17, 5.83 Hz, 2H), 5.69 – 5.60 (m, 2H), 4.94 (d, *J* = 7.38 Hz, 1H), 4.90 – 4.70 (m, 3H), 4.42 (dd, *J* = 32.07, 11.86 Hz, 2H), 4.24 (d, *J* = 9.61 Hz, 1H), 4.18 – 3.98 (m, 3H), 3.87 (d, *J* = 12.39 Hz, 5H), 1.16 (t, *J* = 7.12 Hz, 3H), 1.08 (t, *J* = 7.10 Hz, 2H). ^{13}C NMR (101 MHz, $CDCl_3$) δ 171.0, 170.5, 170.3, 170.1, 163.4, 143.6, 143.3, 138.1, 137.9, 134.9, 134.8, 133.2, 132.9, 131.3, 129.7, 129.6, 129.1, 128.6, 128.4, 128.3, 128.1, 127.5, 127.3, 127.0, 126.8, 126.6, 121.7, 121.6, 114.7, 114.5, 86.3, 85.9, 68.0, 67.9, 65.4, 64.4, 61.8, 61.4, 52.8, 52.6, 14.0, 13.9. **HRMS(ESI)** Calcd. for $C_{29}H_{30}ClN_2O_6$ $(M+H)^+$ 537.1787, found 537.1788.

4-ethyl 1-methyl 3-((4-acrylamidophenyl)amino)-2-(benzyloxy)-2-(4-bromophenyl)succinate (4c)

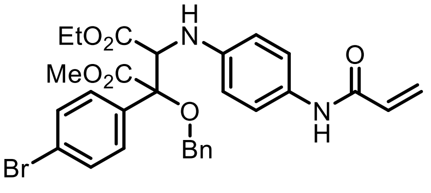

4c *syn/anti* isomer (2:1) ratio

White solid, 93 mg, 80% yield, R_f = 0.2 (PE/EA = 2/1), *syn*/*anti* isomer (2:1) ratio. Mixture of (*syn*/*anti*) isomers of **4c**: ^{1}H NMR (400 MHz, Chloroform-*d*) δ 7.67 (d, *J* = 7.95 Hz, 2H), 7.45 (dd, *J* = 16.38, 7.10 Hz, 6H), 7.31 (m, 13H), 6.64 (d, *J* = 8.38 Hz, 1H), 6.51 (d, *J* = 8.38 Hz, 2H), 6.34 (d, *J* = 16.57 Hz, 2H), 6.20 (m, 2H), 5.64 (d, *J* = 10.19

Hz, 2H), 4.93 (s, 1H), 4.76 (d, J = 28.57 Hz, 2H), 4.42 (dd, J = 32.44, 11.70 Hz, 2H), 4.10 (d, J = 7.48 Hz, 3H), 3.86 (d, J = 13.42 Hz, 5H), 1.15 (t, J = 7.29 Hz, 3H), 1.09 (t, J = 6.93 Hz, 2H). ^{13}C NMR (101 MHz, $CDCl_3$) δ 170.9, 170.5, 170.3, 170.2, 163.6, 163.5, 143.6, 143.4, 138.1, 137.9, 133.7, 133.5, 131.5, 131.3, 131.0, 130.0, 129.7, 129.5, 129.4, 129.3, 128.5, 128.48, 128.43, 128.40, 128.36, 128.33, 128.1, 128.0, 127.5, 127.3, 127.1, 127.0, 126.9, 126.8, 126.7, 126.6, 123.2, 123.1, 121.8, 121.6, 114.7, 114.5, 86.3, 86.0, 68.1, 68.0, 65.3, 65.2, 64.3, 61.8, 61.5, 52.8, 52.6, 14.0, 13.9. **HRMS(ESI)** Calcd. for $C_{29}H_{30}BrN_2O_6$ $(M+H)^+$ 581.1282, found 581.1287.

4-ethyl 1-methyl 3-((4-acrylamidophenyl)amino)-2-(benzyloxy)-2-(4-(trifluoromethyl)phenyl)succinate (4d)

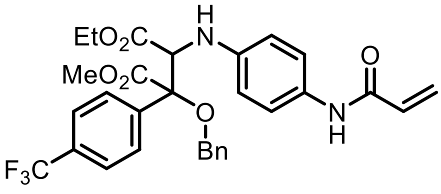

4d *syn/anti* isomer (2:1) ratio

White solid, 88 mg, 77% yield, R_f = 0.2 (PE/EA = 2/1), *syn*/*anti* isomer (2:1) ratio. Mixture of (*syn*/*anti*) isomers of **4d**: ^{1}H NMR (400 MHz, Chloroform-*d*) δ 7.68 (d, J = 7.97 Hz, 4H), 7.60 (q, J = 8.76, 8.02 Hz, 5H), 7.31 (m, 13H), 6.66 (d, J = 8.47 Hz, 1H), 6.53 (d, J = 8.41 Hz, 2H), 6.38 – 6.27 (m, 2H), 6.19 (s, 2H), 5.65 (d, J = 10.13 Hz, 2H), 4.98 (s, 1H), 4.87 – 4.71 (m, 3H), 4.46 (dd, J = 29.68, 11.74 Hz, 2H), 4.17 – 3.98 (m, 4H), 3.88 (d, J = 14.43 Hz, 5H), 1.15 (t, J = 7.07 Hz, 3H), 1.05 (t, J = 7.22 Hz, 2H). ^{13}C NMR (101 MHz, $CDCl_3$) δ 170.7, 170.2, 170.1, 170.0, 163.4, 143.5, 143.3, 138.8, 138.6, 137.9, 137.7, 131.2, 129.9, 129.7, 128.7, 128.4, 128.3, 128.1, 127.6, 127.4, 127.0, 126.9, 126.6, 125.3, 125.2, 124.8, 124.7, 121.7, 121.6, 114.8, 114.7, 86.3, 86.1, 68.2, 65.6, 64.3, 61.8, 52.8, 52.6, 13.9, 13.7. **HRMS(ESI)** Calcd. for $C_{20}H_{30}F_3N_2O_6$ $(M+H)^+$ 571.2050, found 571.2043.

4-ethyl 1-methyl 2-([1,1'-biphenyl]-4-yl)-3-((4-acrylamidophenyl)amino)-2-(benzyloxy)succinate (4e)

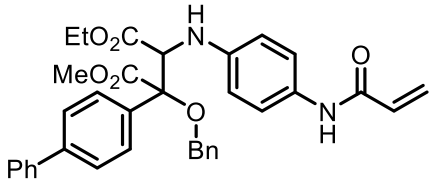

4e *syn/anti* isomer (2:1) ratio

White solid, 98 mg, 85% yield, R_f = 0.2 (PE/EA = 2/1), *syn*/*anti* isomer (2:1) ratio. Mixture of (*syn*/*anti*) isomers of **4e**: ^{1}H NMR (400 MHz, Chloroform-*d*) δ 7.65 – 7.48 (m, 14H), 7.47 – 7.24 (m, 19H), 6.65 (d, J = 8.54 Hz, 1H), 6.53 (d, J = 8.37 Hz, 2H), 6.32 (d, J = 16.90 Hz, 2H), 6.19 (s, 2H), 5.62 (d, J = 10.22 Hz, 2H), 5.00 (s, 1H), 4.92 – 4.74 (m, 3H), 4.52 (dd, J = 41.05, 11.70 Hz, 2H), 4.17 – 3.95 (m, 4H), 3.89 (d, J = 15.96 Hz, 5H), 1.15 (t, J = 7.12 Hz, 3H), 1.06 (t, J = 7.13 Hz, 2H). ^{13}C NMR (101 MHz, $CDCl_3$) δ 171.4, 170.9, 170.6, 170.3, 163.3, 143.8, 143.5, 141.7, 141.5, 141.4, 140.3, 140.1, 138.5, 138.3, 133.5, 133.4, 131.3, 128.9, 128.8, 128.7, 128.3, 128.2, 128.0, 127.5, 127.4, 127.2, 127.1, 126.9, 126.7, 126.6, 121.7, 121.6, 114.6, 114.5, 86.6, 86.2, 68.0, 67.9, 65.3, 64.4, 61.7, 61.3, 52.7, 52.5, 14.0, 13.9. **HRMS(ESI)** Calcd. for $C_{35}H_{35}N_2O_6$ $(M+H)^+$ 579.2490, found 579.2488.

4-ethyl 1-methyl 3-((4-acrylamidophenyl)amino)-2-(benzyloxy)-2-(3,4-difluorophenyl)succinate (4f)

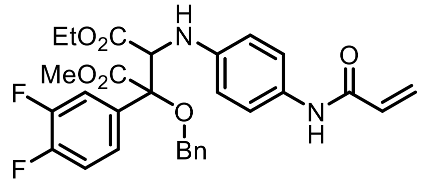

4f *syn/anti* isomer (2:1) ratio

White solid, 85 mg, 79% yield, R_f = 0.2 (PE/EA = 2/1), *syn*/*anti* isomer (2:1) ratio. Mixture of (*syn*/*anti*) isomers of **4f**: ^{1}H NMR (400 MHz, Chloroform-*d*) δ 7.56 (m, 11H), 7.47 – 7.38 (m, 5H), 7.35 (t, J = 7.68 Hz, 10H), 7.27 (d, J = 8.12 Hz, 3H), 6.66 (d, J = 8.32 Hz, 1H), 6.54 (d, J = 8.32 Hz, 2H), 6.39 – 6.29 (m, 2H), 6.23 – 6.09 (m, 2H), 5.65 (dd, J = 10.30, 3.88 Hz, 2H), 5.00 (s, 1H), 4.89 – 4.75 (m, 3H), 4.57 (d, J = 11.74 Hz, 1H), 4.47 (d, J = 12.07 Hz, 1H), 4.17 – 4.09 (m, 2H), 4.07 – 3.97 (m, 1H), 3.91 (s, 2H), 3.87 (s, 3H), 1.16 (t, J = 7.14 Hz, 3H), 1.07 (t, J = 7.12 Hz, 2H). ^{13}C NMR (101 MHz, $CDCl_3$) δ 171.4, 170.9, 170.6, 170.3, 163.2, 143.8, 143.5, 141.7, 141.4, 140.3, 140.1, 138.5, 138.3, 133.5, 133.3, 131.3, 128.9, 128.8, 128.7, 128.3, 128.2, 128.0, 127.6, 127.5, 127.4, 127.2, 127.14, 127.11, 127.0, 126.9, 126.7, 126.6, 121.7, 121.5, 114.6, 114.5, 86.6, 86.2, 68.0, 67.9, 65.3, 64.4, 61.7, 61.3, 52.7, 52.5, 14.0, 13.9. **HRMS(ESI)** Calcd. for $C_{29}H_{29}F_2N_2O_6$ $(M+H)^+$ 539.1988, found 539.1986.

4-ethyl 1-methyl 3-((4-acrylamidophenyl)amino)-2-(benzyloxy)-2-(4-chloro-3-fluorophenyl)succinate (4g)

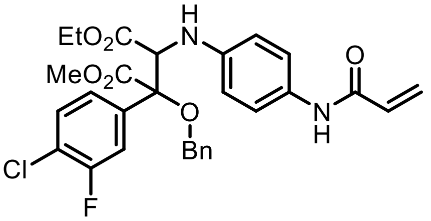

4g *syn/anti* isomer (2:1) ratio

White solid, 86 mg, 78% yield, R_f = 0.2 (PE/EA = 2/1), *syn*/*anti* isomer (2:1) ratio. Mixture of (*syn*/*anti*) isomers of **4g**: ^{1}H NMR (400 MHz, Chloroform-*d*) δ 7.76 – 7.60 (m, 2H), 7.34 (m, 17H), 6.65 (d, J = 8.37 Hz, 1H), 6.51 (d, J = 8.35 Hz, 2H), 6.40 – 6.30 (m, 2H), 6.20 (s, 2H), 5.64 (d, J = 10.22 Hz, 2H), 4.92 (s, 1H), 4.76 (d, J = 12.98 Hz, 3H), 4.47 (d, J = 11.55 Hz, 2H), 4.14 – 3.99 (m, 3H), 3.87 (d, J = 9.71 Hz, 5H), 1.17 (t, J = 7.20 Hz, 3H), 1.09 (t, J = 7.26 Hz, 2H). ^{13}C NMR (101 MHz, $CDCl_3$) δ 170.5, 170.1, 169.9, 163.5, 163.4, 159.1, 158.8, 156.6, 156.3, 143.4, 143.3, 137.8, 137.6, 135.6, 135.5, 131.3, 130.4, 129.9, 129.7, 128.5, 128.4, 128.3, 127.6, 127.5, 127.0, 126.9, 126.9, 126.7, 126.6, 124.8, 124.7, 124.1, 124.1, 121.8, 121.7, 121.6, 121.5, 121.3, 117.1, 116.9, 116.5, 116.3, 114.7, 114.6, 86.0, 85.7, 68.2, 65.7, 64.4, 61.9, 61.6, 52.9, 52.7, 14.0, 13.9. **HRMS(ESI)** Calcd. for $C_{29}H_{29}ClFN_2O_6$ $(M+H)^+$ 555.1693, found 555.1699.

4-ethyl 1-methyl 3-((4-acrylamidophenyl)amino)-2-(benzyloxy)-2-(2,4-dichlorophenyl)succinate (4h)

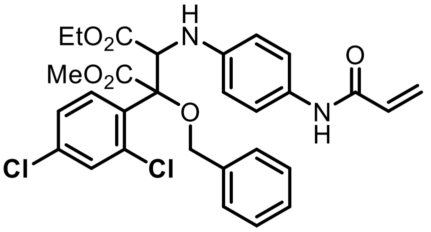

4h *syn/anti* isomer (3:1) ratio

White solid, 89 mg, 78% yield, R_f = 0.2 (PE/EA = 2/1), *syn*/*anti* isomer (2:1) ratio. Mixture of (*syn*/*anti*) isomers of **4h**: ^{1}H NMR (400 MHz, Chloroform-*d*) δ 7.58 – 7.52 (m, 2H), 7.44 – 7.21 (m, 12H), 6.63 (dd, J = 13.03, 8.55 Hz, 3H), 6.34 (d, J = 16.82 Hz, 1H), 6.19 (dd, J = 16.84, 10.15 Hz, 1H), 5.64 (d, J = 10.15 Hz, 1H), 5.14 (d, J = 23.84 Hz, 1H), 4.74 (d, J = 11.08 Hz, 1H), 4.51 (d, J = 11.11 Hz, 1H), 4.12 (q, J = 6.98 Hz, 2H), 4.03 – 3.90 (m, 1H), 3.83 (s, 1H), 3.73 (s, 3H), 1.13 (t, J = 7.12 Hz, 3H), 1.04 (t, J = 7.11 Hz, 1H). ^{13}C NMR (101 MHz, $CDCl_3$) δ 170.6, 170.5, 170.1, 169.0, 163.4, 163.3, 143.4, 142.8, 137.2, 135.5, 135.0, 134.7, 133.6, 133.3, 132.5, 131.3, 131.1, 131.0, 131.1, 130.6, 129.4, 128.4, 128.3, 127.8,

127.6, 127.4, 127.2, 127.0, 126.9, 126.8, 121.9, 121.6, 114.5, 114.0, 85.1, 83.4, 68.6, 67.7, 61.5, 61.4, 61.3, 60.2, 53.0, 52.8, 13.9, 13.8. **HRMS(ESI)** Calcd. for $C_{29}H_{29}Cl_2N_2O_6$ $(M+H)^+$ 571.1397, found 571.1393.

4-ethyl 1-methyl 3-((4-acrylamidophenyl)amino)-2-(benzyloxy)-2-(naphthalen-1-yl)succinate (4i)

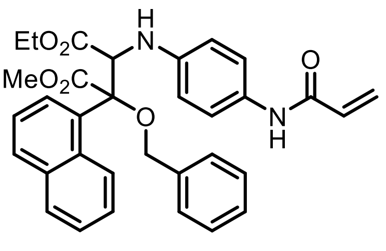

4i *syn/anti* isomer (2:1) ratio

White solid, 84 mg, 76% yield, R_f = 0.2 (PE/EA = 2/1), *syn*/*anti* isomer (2:1) ratio. Mixture of (*syn*/*anti*) isomers of **4i**: ^{1}H NMR (400 MHz, Chloroform-*d*) δ 8.01 (s, 1H), 7.80 (dd, J = 9.20, 5.25 Hz, 4H), 7.64 (d, J = 8.65 Hz, 1H), 7.51 – 7.45 (m, 3H), 7.36 (d, J = 5.69 Hz, 4H), 7.25 (d, J = 3.96 Hz, 2H), 6.53 (d, J = 8.43 Hz, 2H), 6.36 – 6.29 (m, 1H), 6.14 (dd, J = 16.86, 10.17 Hz, 1H), 5.67 – 5.62 (m, 1H), 5.04 (d, J = 8.61 Hz, 1H), 4.80 (d, J = 12.48 Hz, 1H), 4.54 (d, J = 11.73 Hz, 1H), 4.41 – 4.30 (m, 1H), 4.12 – 4.05 (m, 2H), 3.90 (s, 3H), 1.11 (t, J = 7.14 Hz, 3H), 1.01 (t, J = 7.16 Hz, 2H). ^{13}C NMR (101 MHz, $CDCl_3$) δ 171.5, 170.9, 170.7, 170.3, 163.2, 163.1, 143.8, 138.5, 138.2, 133.2, 133.1, 132.8, 132.6, 132.1, 131.2, 129.8, 129.2, 128.5, 128.3, 128.2, 127.8, 127.6, 127.5, 127.48, 127.44, 127.2, 127.0, 126.9, 126.8, 126.7, 126.4, 126.2, 125.7, 124.4, 121.6, 121.5, 114.7, 114.5, 86.9, 86.4, 68.1, 68.0, 65.2, 64.7, 61.7, 61.3, 52.7, 52.5, 13.9, 13.8. **HRMS(ESI)** Calcd. for $C_{33}H_{33}N_2O_6$ $(M+H)^+$ 553.2333, found 553.2332.

4-ethyl 1-methyl 3-((4-acrylamidophenyl)amino)-2-(4-chlorophenyl)-2-((4-fluorobenzyl)oxy)succinate (4j)

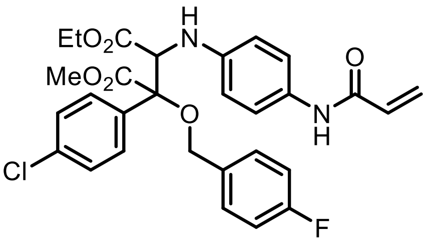

4j *syn/anti* isomer (3:2) ratio

White solid, 83 mg, 77% yield, R_f = 0.2 (PE/EA = 2/1), *syn*/*anti* isomer (2:1) ratio. Mixture of (*syn*/*anti*) isomers of **4j**: ^{1}H NMR (400 MHz, Chloroform-*d*) δ 7.75 (s, 2H), 7.46 (dd, J = 14.38, 8.31 Hz, 4H), 7.30 (dd, J = 15.38, 8.91 Hz, 15H), 6.63 (d, J = 8.31 Hz, 1H), 6.50 (d, J = 8.64 Hz, 2H), 6.33 (d, J = 16.61 Hz, 2H), 6.26 – 6.13 (m, 2H), 5.62 (d, J = 10.19 Hz, 2H), 4.94 (s, 1H), 4.82 – 4.72 (m, 2H), 4.42 (dd, J = 32.20, 11.78 Hz, 2H), 4.10 (d, J = 7.81 Hz, 4H), 3.86 (d, J = 12.50 Hz, 5H), 1.15 (t, J = 7.36 Hz, 3H), 1.09 (t, J = 7.47 Hz, 2H). ^{13}C NMR (101 MHz, $CDCl_3$) δ 171.0, 170.5, 170.3, 170.2, 163.5, 143.6, 143.3, 138.1, 137.9, 134.9, 134.8, 133.2, 133.0, 131.3, 129.8, 129.7, 129.1, 128.6, 128.4, 128.3, 128.1, 127.5, 127.3, 126.9, 126.8, 126.6, 121.7, 121.6, 114.6, 114.5, 86.3, 85.9, 68.0, 68.0, 65.4, 64.4, 61.8, 61.4, 52.7, 52.5, 14.1, 14.0, 13.9. **HRMS(ESI)** Calcd. for $C_{29}H_{29}ClFN_2O_6$ $(M+H)^+$ 555.1693.1787, found 555.1699.

4-ethyl 1-methyl 3-((4-acrylamidophenyl)amino)-2-((4-chlorobenzyl)oxy)-2-(4-chlorophenyl)succinate (4k)

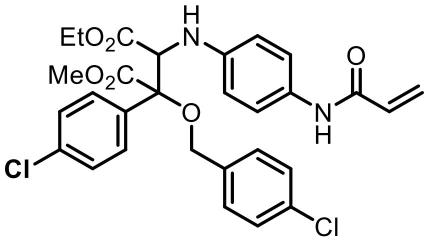

4k *syn/anti* isomer (3:1) ratio

White solid, 96 mg, 84% yield, R_f = 0.2 (PE/EA = 2/1), *syn*/*anti* isomer (2:1) ratio. Mixture of (*syn*/*anti*) isomers of **4k**: ^{1}H NMR (400 MHz, Chloroform-*d*) δ 7.62 (d, *J* = 7.47 Hz, 1H), 7.51 (d, *J* = 8.55 Hz, 1H), 7.41 (d, *J* = 2.22 Hz, 1H), 7.32 (dd, *J* = 7.89, 4.61 Hz, 3H), 7.28 – 7.21 (m, 4H), 7.13 (s, 1H), 6.67 (d, *J* = 8.35 Hz, 2H), 6.38 (dd, *J* = 16.82, 5.55 Hz, 1H), 6.19 (dd, *J* = 16.84, 10.14 Hz, 1H), 5.71 (dd, *J* = 10.42, 4.69 Hz, 1H), 5.15 (d, *J* = 8.38 Hz, 1H), 4.82 (d, *J* = 12.68 Hz, 1H), 4.69 – 4.60 (m, 1H), 4.35 (d, *J* = 10.04 Hz, 1H), 4.16 – 4.05 (m, 2H), 3.73 (s, 3H), 1.12 (t, *J* = 7.14 Hz, 3H), 1.03 (t, *J* = 7.13 Hz, 1H). ^{13}C NMR (101 MHz, $CDCl_3$) *δ* 170.4, 168.9, 163.1, 143.5, 135.2, 133.8, 132.9, 132.1, 131.2, 131.1, 130.7, 129.0, 128.8, 128.7, 127.0, 126.9, 126.8, 121.6, 114.6, 65.7, 61.4, 58.5, 52.7, 13.8. **HRMS(ESI)** Calcd. for $C_{29}H_{29}Cl_2N_2O_6$ $(M+H)^+$ 571.1397, found 571.1393.

4-ethyl 1-methyl 3-((4-acrylamidophenyl)amino)-2-((4-bromobenzyl)oxy)-2-(4-chlorophenyl)succinate (4l)

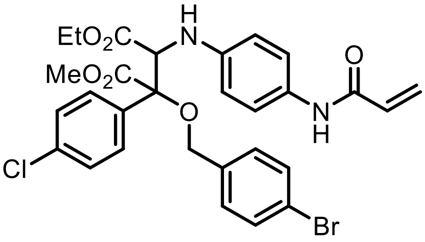

4l *syn/anti* isomer (3:2) ratio

White solid, 99 mg, 81% yield, R_f = 0.2 (PE/EA = 2/1), *syn*/*anti* isomer (2:1) ratio. Mixture of (*syn*/*anti*) isomers of **4l**: ^{1}H NMR (400 MHz, Chloroform-*d*) *δ* 7.64 (d, *J* = 13.54 Hz, 2H), 7.45 (m, *J* = 9.86, 9.00, 3.78 Hz, 6H), 7.41 – 7.32 (m, 3H), 7.28 (dd, *J* = 8.74, 5.16 Hz, 5H), 7.19 (t, *J* = 7.92 Hz, 4H), 6.64 (d, *J* = 8.51 Hz, 1H), 6.47 (d, *J* = 8.45 Hz, 2H), 6.35 (dd, *J* = 16.87, 5.42 Hz, 2H), 6.20 (m, 2H), 5.65 (dd, *J* = 10.36, 4.02 Hz, 2H), 4.89 (d, *J* = 8.49 Hz, 1H), 4.79 (s, 1H), 4.76 – 4.66 (m, 2H), 4.39 (d, *J* = 12.03 Hz, 1H), 4.32 (d, *J* = 11.97 Hz, 2H), 4.11 (dd, *J* = 7.16, 4.41 Hz, 4H), 3.88 (d, *J* = 8.61 Hz, 5H), 1.16 (t, *J* = 7.13 Hz, 3H), 1.08 (t, *J* = 7.13 Hz, 2H). ^{13}C NMR (101 MHz, $CDCl_3$) *δ* 170.8, 170.5, 170.2, 170.0, 163.5, 163.4, 143.6, 143.2, 137.2, 137.1, 135.1, 134.9, 133.0, 132.8, 131.5, 131.4, 131.3, 129.8, 129.7, 129.6, 129.0, 128.6, 128.5, 128.3, 128.1, 127.0, 121.7, 121.6, 121.3, 121.1, 114.5, 86.4, 86.0, 67.4, 67.3, 65.3, 64.6, 61.9, 61.5, 52.8, 52.6, 14.0, 13.9. **HRMS(ESI)** Calcd. for $C_{29}H_{29}BrClN_2O_6$ $(M+H)^+$ 615.0892, found 615.0890.

4-ethyl 1-methyl 3-((4-acrylamidophenyl)amino)-2-((2-bromobenzyl)oxy)-2-(4-chlorophenyl)succinate (4m)

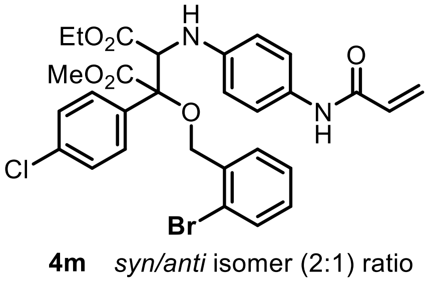

4m *syn/anti* isomer (2:1) ratio

White solid, 95 mg, 77% yield, R_f = 0.2 (PE/EA = 2/1), *syn*/*anti* isomer (2:1) ratio. Mixture of (*syn*/*anti*) isomers of **4m**: ^{1}H NMR (400 MHz, Chloroform-*d*) *δ* 7.62 (t, *J* = 8.06 Hz, 2H), 7.50 (dd, *J* = 11.17, 8.11 Hz, 4H), 7.43 (d, *J* = 8.39 Hz, 1H), 7.35 (m, 3H), 7.31 – 7.25 (m, 5H), 7.24 – 7.11 (m, 3H), 6.67 (d, *J* = 8.38 Hz, 1H), 6.50 (d, *J* = 8.32 Hz, 2H), 6.37 (dd, *J* = 16.82, 5.04 Hz, 1H), 6.19 (m, 2H), 5.70 (dd, *J* = 10.39, 3.85 Hz, 1H), 4.93 (s, 1H), 4.89 – 4.73 (m, 2H), 4.52 (d, *J* = 13.14 Hz, 1H), 4.43 (d, *J* = 13.51 Hz, 1H), 4.18 – 4.09 (m, 2H), 4.07 – 3.95 (m, 1H), 3.89 (d, *J* = 7.30 Hz, 4H), 1.18 (t, *J* = 7.12 Hz, 3H), 1.09 (t, *J* = 7.14 Hz, 1H). ^{13}C NMR (101 MHz, $CDCl_3$) *δ* 170.7, 170.4, 170.0, 163.2, 143.7, 143.4, 137.5, 137.4, 134.9, 132.6, 132.3, 132.2, 131.2, 129.6, 128.8, 128.7, 128.7, 128.6, 128.3, 128.2, 127.5, 127.4, 127.1, 121.8, 121.7, 121.5, 114.6, 114.6, 86.4, 86.2, 67.5, 67.5, 65.6,

64.6, 61.9, 61.4, 52.8, 52.6, 13.9, 13.8. **HRMS(ESI)** Calcd. for $C_{29}H_{29}BrClN_2O_6$ $(M+H)^+$ 615.0892, found 615.0890.

4-ethyl 1-methyl 3-((4-acrylamidophenyl)amino)-2-((2-chlorobenzyl)oxy)-2-(4-chlorophenyl)succinate (4n)

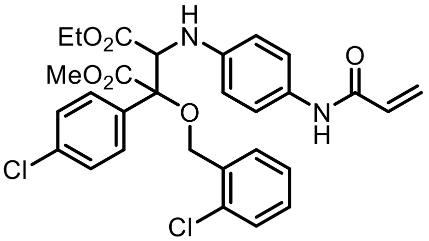

4n *syn/anti* isomer (2:1) ratio

White solid, 87 mg, 76% yield, R_f = 0.2 (PE/EA = 2/1), *syn*/*anti* isomer (2:1) ratio. Mixture of (*syn*/*anti*) isomers of **4n**: ^{1}H NMR (400 MHz, Chloroform-*d*) δ 7.62 (t, *J* = 7.61 Hz, 2H), 7.53 (d, *J* = 11.80 Hz, 1H), 7.49 – 7.42 (m, 5H), 7.37 (d, *J* = 7.94 Hz, 2H), 7.30 (d, *J* = 8.54 Hz, 4H), 7.27 – 7.16 (m, 2H), 6.66 (d, *J* = 8.30 Hz, 1H), 6.50 (d, *J* = 8.33 Hz, 2H), 6.35 (dd, *J* = 16.77, 4.58 Hz, 2H), 6.18 (dd, *J* = 17.16, 9.17 Hz, 2H), 5.66 (d, *J* = 10.15 Hz, 1H), 4.99 – 4.75 (m, 3H), 4.52 (dd, *J* = 37.19, 13.33 Hz, 2H), 4.20 – 3.94 (m, 3H), 3.88 (d, *J* = 7.88 Hz, 5H), 1.17 (t, *J* = 7.23 Hz, 3H), 1.08 (t, *J* = 7.25 Hz, 1H). ^{13}C NMR (101 MHz, $CDCl_3$) δ 170.7, 170.3, 170.1, 163.3, 143.6, 143.3, 135.8, 133.2, 131.9, 131.7, 131.3, 131.1, 129.9, 129.1, 129.0, 128.4, 128.2, 128.0, 127.0, 126.9, 126.8, 123.2, 121.7, 121.6, 114.6, 86.5, 86.2, 65.5, 65.2, 64.6, 61.9, 61.5, 52.8, 52.6, 13.9, 13.8. **HRMS(ESI)** Calcd. for $C_{29}H_{29}Cl_2N_2O_6$ $(M+H)^+$ 571.1397, found 571.1393.

4-ethyl 1-methyl 3-((4-acrylamidophenyl)amino)-2-((3-chlorobenzyl)oxy)-2-(4-chlorophenyl)succinate (4o)

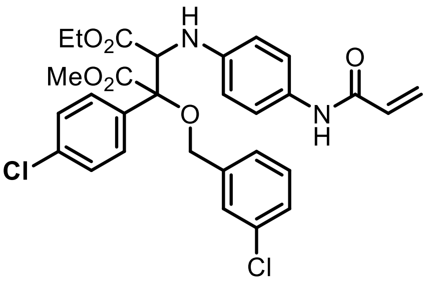

4o *syn/anti* isomer (3:2) ratio

White solid, 87 mg, 76% yield, R_f = 0.2 (PE/EA = 2/1), *syn*/*anti* isomer (2:1) ratio. Mixture of (*syn*/*anti*) isomers of **4o**: ^{1}H NMR (400 MHz, Chloroform-*d*) δ 7.52 (t, *J* = 9.23 Hz, 3H), 7.41 (d, *J* = 9.29 Hz, 2H), 7.32 (m, 6H), 7.14 (d, *J* = 9.04 Hz, 1H), 6.63 (d, *J* = 8.42 Hz, 1H), 6.50 (d, *J* = 8.32 Hz, 2H), 6.40 – 6.26 (m, 5H), 6.18 (m, 2H), 5.70 (d, *J* = 10.09 Hz, 2H), 4.85 (dd, *J* = 10.20, 4.96 Hz, 2H), 4.74 (dd, *J* = 22.30, 11.87 Hz, 2H), 4.40 (d, *J* = 12.10 Hz, 1H), 4.25 (d, *J* = 9.65 Hz, 1H), 4.06 (dd, *J* = 9.61, 5.72 Hz, 2H), 3.91 (d, *J* = 3.05 Hz, 4H), 1.14 (t, *J* = 7.22 Hz, 3H), 1.06 (t, *J* = 7.14 Hz, 2H). ^{13}C NMR (101 MHz, $CDCl_3$) δ 171.0, 170.5, 170.3, 170.1, 163.4, 163.4, 143.6, 143.3, 138.1, 137.9, 134.9, 134.8, 133.2, 132.9, 131.3, 129.7, 129.6, 129.1, 128.6, 128.3, 128.3, 128.1, 127.5, 127.3, 127.0, 126.8, 126.6, 121.7, 121.6, 114.7, 114.5, 86.3, 85.9, 68.0, 67.9, 65.4, 64.4, 61.8, 61.4, 52.8, 52.6, 14.0, 13.9. **HRMS(ESI)** Calcd. for $C_{29}H_{29}Cl_2N_2O_6$ $(M+H)^+$ 571.1397, found 571.1393.

4-ethyl 1-methyl 3-((4-acrylamidophenyl)amino)-2-(4-chlorophenyl)-2-(furan-2-ylmethoxy)succinate (4p)

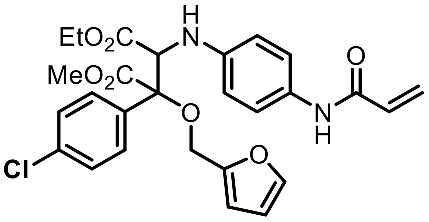

4p *syn/anti* isomer (2:1) ratio

White solid, 75 mg, 71% yield, R_f = 0.2 (PE/EA = 2/1), *syn*/*anti* isomer (2:1) ratio. Mixture of (*syn*/*anti*) isomers of **4p**: ^{1}H NMR (400 MHz, Chloroform-*d*) δ 7.52 (t, *J* = 9.32 Hz, 3H), 7.41 (d, *J* = 9.59 Hz, 2H), 7.36 – 7.25 (m, 8H), 6.62 (d, *J* = 8.45 Hz, 1H), 6.49 (d, *J* = 8.37 Hz, 2H), 6.40 – 6.25 (m, 5H), 6.23 – 6.13 (m, 2H), 5.68 (d, *J* = 10.17 Hz, 2H), 4.86

(s, 2H), 4.71 (d, J = 11.71 Hz, 2H), 4.40 (d, J = 12.11 Hz, 2H), 4.19 – 4.10 (m, 1H), 4.08 – 3.97 (m, 2H), 3.90 (d, J = 2.78 Hz, 4H), 1.14 (t, J = 7.18 Hz, 3H), 1.05 (t, J = 7.16 Hz, 2H). ^{13}C NMR (101 MHz, $CDCl_3$) δ 171.0, 170.5, 170.1, 170.0, 163.3, 163.2, 151.3, 151.2, 143.7, 143.4, 142.7, 142.6, 135.0, 134.8, 133.1, 132.8, 131.2, 129.8, 129.5, 129.1, 128.6, 128.1, 127.0, 121.6, 121.5, 114.6, 110.4, 110.3, 109.2, 108.9, 86.2, 85.8, 65.3, 64.4, 61.7, 61.3, 61.1, 60.9, 52.8, 52.6, 13.9, 13.8. **HRMS(ESI)** Calcd. for $C_{27}H_{28}ClN_2O_7$ $(M+H)^+$ 527.1580, found 527.1587.

4-ethyl 1-methyl 3-((4-acrylamidophenyl)amino)-2-(4-chlorophenyl)-2-(thiophen-2-ylmethoxy)succinate (4q)

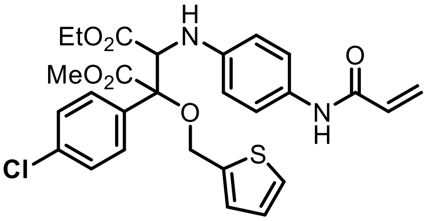

4q *syn/anti* isomer (2:1) ratio

White solid, 81 mg, 75% yield, R_f = 0.2 (PE/EA = 2/1), *syn*/*anti* isomer (2:1) ratio. Mixture of (*syn*/*anti*) isomers of **4q**: ^{1}H NMR (400 MHz, Chloroform-*d*) δ 7.50 (dd, J = 14.35, 8.31 Hz, 3H), 7.40 (s, 2H), 7.30 (m, 8H), 6.99 – 6.91 (m, 3H), 6.63 (d, J = 8.35 Hz, 1H), 6.46 (d, J = 8.39 Hz, 2H), 6.33 (s, 2H), 6.18 (m, 2H), 5.67 (d, J = 9.84 Hz, 2H), 4.94 – 4.84 (m, 3H), 4.64 – 4.51 (m, 2H), 4.19 – 4.08 (m, 2H), 4.06 – 3.98 (m, 1H), 3.91 (s, 4H), 1.18 (t, J = 7.14 Hz, 3H), 1.08 (t, J = 7.13 Hz, 2H). ^{13}C NMR (101 MHz, $CDCl_3$) δ 170.9, 170.6, 170.2, 169.9, 163.4, 163.3, 143.7, 143.3, 140.7, 140.6, 135.0, 134.9, 133.1, 132.8, 131.2, 129.8, 129.6, 129.5, 129.0, 128.6, 128.1, 127.0, 126.5, 126.4, 125.7, 125.68, 125.63, 125.5, 121.7, 121.5, 114.6, 114.4, 86.5, 86.0, 65.5, 64.6, 63.9, 63.8, 61.9, 61.4, 52.8, 52.6, 14.0, 13.9. **HRMS(ESI)** Calcd. for $C_{27}H_{28}ClN_2O_6S$ $(M+H)^+$ 543.1351, found 543.1354.

4-ethyl 1-methyl 3-((4-acrylamidophenyl)amino)-2-(4-chlorophenyl)-2-methoxysuccinate (4r)

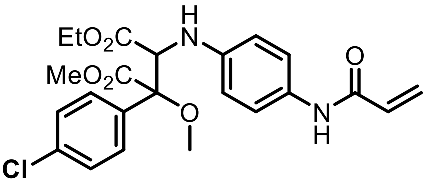

4r *syn/anti* isomer (3:2) ratio

White solid, 63 mg, 68% yield, R_f = 0.2 (PE/EA = 2/1), *syn*/*anti* isomer (2:1) ratio. Mixture of (*syn*/*anti*) isomers of **4r**: ^{1}H NMR (400 MHz, Chloroform-*d*) δ 7.44 (dd, J = 11.77, 8.42 Hz, 4H), 7.37 – 7.27 (m, 7H), 7.15 (d, J = 8.67 Hz, 2H), 6.64 (d, J = 8.41 Hz, 1H), 6.51 (d, J = 8.37 Hz, 2H), 6.38 (dd, J = 16.76, 3.45 Hz, 2H), 6.19 (m, 2H), 5.71 (d, J = 10.18 Hz, 2H), 4.83 (dd, J = 14.08, 10.16 Hz, 2H), 4.69 (d, J = 10.56 Hz, 1H), 4.32 – 4.00 (m, 5H), 3.89 (d, J = 5.61 Hz, 5H), 3.38 (s, 3H), 3.34 (s, 2H), 1.19 (t, J = 7.16 Hz, 3H), 1.09 (t, J = 7.16 Hz, 2H). ^{13}C NMR (101 MHz, $CDCl_3$) δ 171.7, 163.3, 142.7, 136.0, 134.2, 131.2, 130.3, 129.7, 129.1, 128.9, 128.6, 127.0, 121.9, 113.7, 60.2, 58.4, 53.0, 18.4, 14.1. **HRMS(ESI)** Calcd. for $C_{23}H_{26}ClN_2O_6$ $(M+H)^+$ 461.1474, found 461.1476.

4-ethyl 1-methyl 3-((4-acrylamidophenyl)amino)-2-((4-chlorobenzyl)oxy)-2-(2,4-dichlorophenyl)succinate (4s)

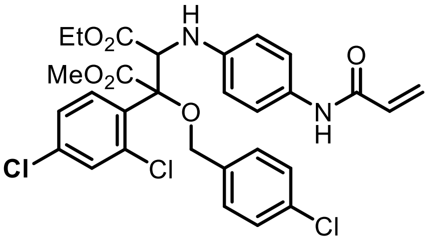

4s *syn/anti* isomer (5:1) ratio

White solid, 98 mg, 81% yield, R_f = 0.2 (PE/EA = 2/1), *syn*/*anti* isomer (2:1) ratio. Mixture of (*syn*/*anti*) isomers of **4s**: ^{1}H NMR (400 MHz, Chloroform-*d*) δ 7.50 – 7.45 (m, 2H), 7.43 – 7.38 (m, 2H), 7.31 (d, J = 9.12 Hz, 4H), 7.28 – 7.23 (m, 2H), 6.63 (t, J = 7.23 Hz, 2H), 6.36 (dd, J = 16.83, 7.06 Hz, 1H), 6.19 (dd, J = 16.89, 9.94 Hz, 1H), 5.66 (dd, J = 10.13, 1.40 Hz, 1H), 5.11 (d, J = 8.08 Hz, 1H), 4.70 (d, J =

11.32 Hz, 1H), 4.45 (d, *J* = 11.35 Hz, 1H), 4.34 (q, *J* = 7.96 Hz, 1H), 4.11 (m, 2H), 3.85 (s, 1H), 3.74 (s, 3H), 1.11 (t, *J* = 7.13 Hz, 3H). ^{13}C NMR (101 MHz, $CDCl_3$) *δ* 170.4, 169.0, 163.33, 143.4, 135.8, 135.1, 133.7, 133.5, 133.1, 131.3, 131.0, 130.7, 129.5, 128.7, 128.6, 128.5, 127.0, 126.8, 121.6, 114.6, 85.5, 67.9, 61.4, 52.8, 13.9. **HRMS(ESI)** Calcd. for $C_{29}H_{28}Cl_3N_2O_6$ $(M+H)^+$ 605.1007, found 605.1001.

4-ethyl 1-methyl 3-((4-acrylamidophenyl)amino)-2-(2,4-dichlorophenyl)-2-((2-methoxybenzyl)oxy)succinate (4t)

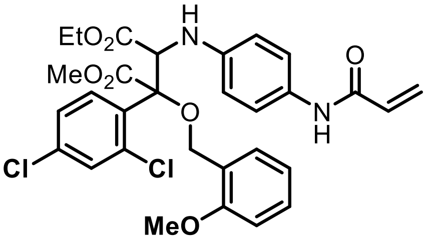

4t *syn/anti* isomer (3:1) ratio

White solid, 97 mg, 80% yield, R_f = 0.2 (PE/EA = 2/1), *syn*/*anti* isomer (2:1) ratio. Mixture of (*syn*/*anti*) isomers of **4t**: ^{1}H NMR (400 MHz, Chloroform-*d*) δ 7.61 (d, *J* = 8.58 Hz, 1H), 7.50 (d, *J* = 7.43 Hz, 1H), 7.40 (d, *J* = 10.34 Hz, 2H), 7.31 (d, *J* = 8.50 Hz, 2H), 7.27 – 7.23 (m, 2H), 6.98 (d, *J* = 7.30 Hz, 1H), 6.85 (d, *J* = 8.05 Hz, 1H), 6.62 (d, *J* = 8.37 Hz, 2H), 6.38 – 6.30 (m, 1H), 6.18 (dd, *J* = 16.88, 9.79 Hz, 1H), 5.66 (dd, *J* = 10.43, 5.19 Hz, 1H), 5.10 (d, *J* = 10.12 Hz, 1H), 4.71 (d, *J* = 12.21 Hz, 1H), 4.61 (d, *J* = 11.97 Hz, 1H), 4.40 (d, *J* = 10.29 Hz, 1H), 4.18 – 4.05 (m, 2H), 3.78 (s, 3H), 3.73 (s, 3H), 1.13 (t, *J* = 7.12 Hz, 3H), 0.99 (t, *J* = 7.12 Hz, 1H). ^{13}C NMR (101 MHz, $CDCl_3$) δ 170.73, 169.04, 163.27, 156.53, 143.50, 134.92, 133.74, 133.52, 131.35, 131.28, 130.47, 129.32, 128.73, 128.53, 128.07, 126.94, 126.71, 125.80, 121.64, 120.58, 114.48, 110.01, 84.83, 63.72, 61.38, 55.29, 52.73, 13.92. **HRMS(ESI)** Calcd. for $C_{30}H_{31}Cl_2N_2O_7$ $(M+H)^+$ 601.1503, found 601.1504.

4-ethyl 1-methyl 3-((4-acrylamidophenyl)amino)-2-(2,4-dichlorophenyl)-2-((4-methylbenzyl)oxy)succinate (4u)

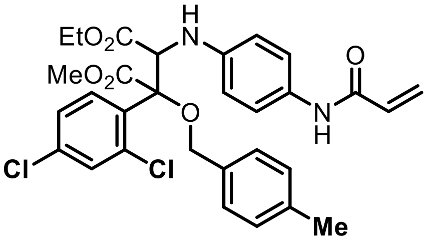

4u *syn/anti* isomer (3:1) ratio

White solid, 98 mg, 84% yield, R_f = 0.2 (PE/EA = 2/1), *syn*/*anti* isomer (2:1) ratio. Mixture of (*syn*/*anti*) isomers of **4u**: ^{1}H NMR (400 MHz, Chloroform-*d*) *δ* 7.57 – 7.49 (m, 2H), 7.40 (dd, *J* = 8.02, 2.36 Hz, 2H), 7.33 – 7.20 (m, 6H), 7.16 (d, *J* = 7.92 Hz, 3H), 6.62 (dd, *J* = 18.83, 8.51 Hz, 3H), 6.45 – 6.29 (m, 1H), 6.27 – 6.11 (m, 1H), 5.65 (dd, *J* = 10.12, 1.57 Hz, 1H), 5.12 (d, *J* = 29.25 Hz, 1H), 4.69 (d, *J* = 10.76 Hz, 1H), 4.46 (d, *J* = 10.79 Hz, 2H), 4.12 (t, *J* = 7.18 Hz, 2H), 4.02 – 3.90 (m, 1H), 3.82 (s, 1H), 3.74 (s, 3H), 2.34 (d, *J* = 5.27 Hz, 4H), 1.14 (t, *J* = 7.12 Hz, 3H), 1.04 (t, *J* = 7.11 Hz, 1H). ^{13}C NMR (101 MHz, $CDCl_3$) *δ* 170.7, 170.5, 170.1, 169.0, 163.4, 163.3, 143.4, 142.8, 137.6, 137.3, 135.5, 134.9, 134.6, 134.2, 133.6, 133.5, 132.7, 131.3, 131.1, 131.0, 130.5, 129.4, 129.1, 129.0, 127.6, 127.3, 127.0, 126.9, 126.7, 121.9, 121.6, 114.5, 114.0, 84.9, 83.4, 68.6, 67.7, 61.5, 61.4, 61.2, 60.2, 52.9, 52.8, 13.9, 13.8. **HRMS(ESI)** Calcd. for $C_{30}H_{31}Cl_2N_2O_6$ $(M+H)^+$ 585.1554, found 585.1555.

4-ethyl 1-methyl 3-((4-acrylamidophenyl)amino)-2-((2-chlorobenzyl)oxy)-2-(2,4-dichlorophenyl)succinate (4v)

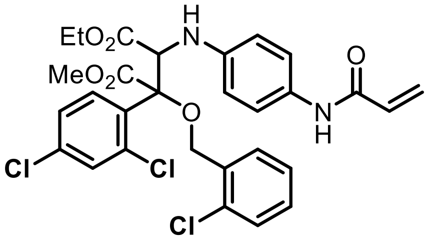

anti-**4v**

White solid, 95 mg, 79% yield, R_f = 0.2 (PE/EA = 2/1), *syn*/*anti* isomer (2:1) ratio. Mixture of (*syn*/*anti*) isomers of **4v**: ^{1}H NMR (400 MHz, Chloroform-*d*) δ 7.68 (d, *J* = 10.53 Hz, 1H), 7.62 (d, *J* = 7.58 Hz, 1H), 7.52 (d, *J* = 8.68 Hz, 1H), 7.40 (s, 2H), 7.32 (t, *J* = 6.76 Hz, 3H), 7.25 (dd, *J* = 11.69, 6.45 Hz, 3H), 6.64 (d, *J* = 8.31 Hz, 2H), 6.41 – 6.27 (m, 1H), 6.20 (dd, *J* = 16.86, 9.84 Hz, 1H), 5.64 (d, *J* = 10.06 Hz, 1H), 5.25 – 5.12 (m, 1H), 4.82 (d, *J* = 12.68 Hz, 1H), 4.66 (d, *J* = 12.75 Hz, 1H), 4.18 – 4.04 (m, 2H), 3.72 (s, 3H), 1.11 (t, *J* = 7.16 Hz, 3H). ^{13}C NMR (101 MHz, $CDCl_3$) δ 170.5, 169.0, 163.4, 143.4, 135.1, 133.8, 132.9, 132.1, 131.3, 131.1, 130.7, 129.5, 129.1, 128.8, 128.7, 126.9, 126.8, 121.6, 114.6, 85.5, 65.7, 61.5, 52.8, 13.9. **HRMS(ESI)** Calcd. for $C_{29}H_{28}Cl_3N_2O_6$ $(M+H)^+$ 605.1007, found 605.1001.

4-ethyl 1-(prop-2-yn-1-yl) 3-((4-acrylamidophenyl)amino)-2-((2-chlorobenzyl)oxy)-2-(2,4-dichlorophenyl)succinate (4w)

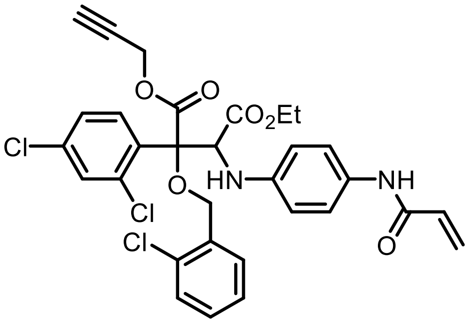

anti-**4w**

White solid, 93 mg, 74% yield, R_f = 0.2 (PE/EA = 2/1), *syn*/*anti* isomer (2:1) ratio. Mixture of (*syn*/*anti*) isomers of **4w**: ^{1}H NMR (400 MHz, Chloroform-*d*) δ 7.60 (d, *J* = 7.48 Hz, 1H), 7.51 (d, *J* = 8.61 Hz, 1H), 7.43 (s, 1H), 7.32 (d, *J* = 8.03 Hz, 3H), 7.25 (q, *J* = 8.83 Hz, 4H), 6.69 (d, *J* = 8.32 Hz, 2H), 6.37 (d, *J* = 16.84 Hz, 1H), 6.19 (dd, *J* = 16.83, 10.17 Hz, 1H), 5.69 (d, *J* = 10.17 Hz, 1H), 5.22 (d, *J* = 8.07 Hz, 1H), 4.85 (dd, *J* = 22.71, 14.12 Hz, 2H), 4.68 (d, *J* = 13.29 Hz, 2H), 4.36 (d, *J* = 9.74 Hz, 1H), 4.10 (s, 2H), 2.48 (s, 1H), 1.09 (t, *J* = 7.13 Hz, 3H). ^{13}C NMR (101 MHz, $CDCl_3$) δ 170.2, 167.9, 163.2, 143.5, 135.3, 135.2, 133.9, 132.4, 132.1, 131.2, 131.1, 130.7, 129.4, 129.0, 128.8, 128.7, 127.1, 126.9, 121.6, 114.8, 85.9, 76.3, 76.0, 65.8, 61.7, 61.6, 53.3, 13.8. **HRMS(ESI)** Calcd. for $C_{31}H_{28}Cl_3N_2O_6$ $(M+H)^+$ 629.1007, found 629.1012.

Methyl2-acrylamido-2-(6-chloropyridin-3-yl)-3-(4-methoxyphenyl)-3-(phenylamino)propanoate (5a)

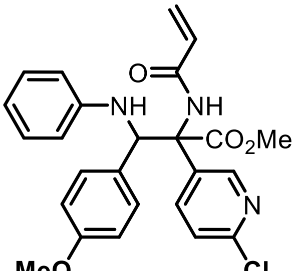

anti-**5a**

White solid, 75 mg, 81% yield, R_f = 0.3 (PE/EA = 5/1), **5a**: ^{1}H NMR (400 MHz, Chloroform-*d*) δ 8.37 (d, *J* = 2.7 Hz, 1H), 7.64 (dd, *J* = 8.5, 2.7 Hz, 1H), 7.27 (d, *J* = 8.6 Hz, 1H), 7.14 (d, *J* = 8.3 Hz, 2H), 7.05 (t, *J* = 7.7 Hz, 2H), 7.02 (s, 1H), 6.92 (d, *J* = 6.7 Hz, 1H), 6.83 (d, *J* = 8.3 Hz, 2H), 6.60 (t, *J* = 7.3 Hz, 1H), 6.45 (d, *J* = 7.9 Hz, 2H), 6.35 (d, *J* = 16.8 Hz, 1H), 6.22 (dd, *J* = 16.9, 10.1 Hz, 1H), 5.79 (d, *J* = 10.0 Hz, 1H), 5.58 (d, *J* = 6.6 Hz, 1H), 3.77 (d, *J* = 1.7 Hz, 6H). ^{13}C NMR (101 MHz, $CDCl_3$) δ 170.5, 165.9, 159.6, 151.1, 148.7, 146.3, 138.0, 130.9, 130.0, 129.5, 129.1, 129.1, 128.8, 124.0, 117.1, 114.1, 112.6, 69.6, 61.5, 55.2, 54.3. **HRMS(ESI)** Calcd. for $C_{25}H_{25}ClN_3O_4$ $(M+H)^+$ 466.1528, found 466.1524.

Methyl2-acrylamido-3-(4-chlorophenyl)-2-(6-chloropyridin-3-yl)-3-(phenylamino)propanoate (5b)

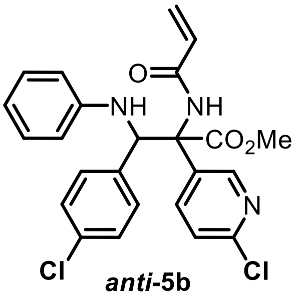

anti-**5b**

White solid, 70 mg, 75% yield, R_f = 0.3 (PE/EA = 5/1), **5b**: ^{1}H NMR (400 MHz, Chloroform-*d*) δ 8.36 (d, *J* = 2.7 Hz, 1H), 7.63 (dd, *J* = 8.5, 2.7 Hz, 1H), 7.30 (d, *J* = 2.6 Hz, 1H), 7.27 (d, *J* = 2.8 Hz, 2H), 7.20 – 7.15 (m, 2H), 7.10 – 7.03 (m, 2H), 6.98 (d, *J* = 7.2 Hz, 2H), 6.63 (t, *J* = 7.3 Hz, 1H), 6.43 (d, *J* = 7.9 Hz, 2H), 6.36 (dd, *J* = 16.9, 1.3 Hz, 1H), 6.22 (dd, *J* = 16.8, 10.1 Hz, 1H), 5.82 (dd, *J* = 10.1, 1.3 Hz, 1H), 5.62 (d, *J* = 6.8 Hz, 1H), 3.79 (s, 3H). ^{13}C NMR (101 MHz, $CDCl_3$) δ 170.3, 165.9, 151.3, 148.6, 145.9, 137.9, 136.6, 134.3, 130.5, 129.7, 129.4, 129.2, 129.0, 124.1, 117.4, 112.6, 69.5, 61.5, 54.4. **HRMS(ESI)** Calcd. for $C_{24}H_{22}Cl_2N_3O_3$ $(M+H)^+$ 470.1033, found 470.1039.

Methyl2-acrylamido-2-(6-chloropyridin-3-yl)-3-(4-cyanophenyl)-3-(phenylamino)propanoate (5c)

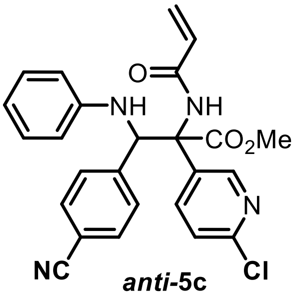

anti-**5c**

White solid, 63 mg, 68% yield, R_f = 0.3 (PE/EA = 5/1), **5c**: ^{1}H NMR (400 MHz, Chloroform-*d*) δ 8.36 (d, *J* = 2.7 Hz, 1H), 7.62 (d, *J* = 2.6 Hz, 1H), 7.60 (d, *J* = 3.1 Hz, 2H), 7.37 (d, *J* = 8.1 Hz, 2H), 7.29 (d, *J* = 8.5 Hz, 1H), 7.09 (s, 1H), 7.06 (d, *J* = 7.5 Hz, 2H), 6.97 (s, 1H), 6.65 (t, *J* = 7.3 Hz, 1H), 6.42 (s, 1H), 6.38 (d, *J* = 17.0 Hz, 2H), 6.22 (dd, *J* = 16.9, 10.1 Hz, 1H), 5.84 (d, *J* = 10.1 Hz, 1H), 5.72 (d, *J* = 6.9 Hz, 1H), 3.82 (s, 3H). ^{13}C NMR (101 MHz, $CDCl_3$) δ 170.1, 166.0, 151.5, 148.5, 145.6, 144.0, 137.8, 132.6, 130.2, 129.8, 129.5, 129.3, 128.5, 124.2, 118.3, 117.8, 112.5, 69.4, 61.9, 54.7. **HRMS(ESI)** Calcd. for $C_{25}H_{22}ClN_4O_3$ $(M+H)^+$ 461.1375, found 461.1369.

Methyl2-acrylamido-2-(6-chloropyridin-3-yl)-3-phenyl-3-(p-tolylamino)propanoate (5d)

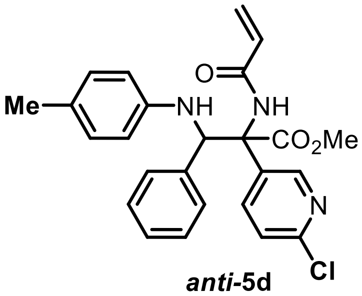

anti-**5d**

White solid, 68 mg, 76% yield, R_f = 0.3 (PE/EA = 5/1), **5d**: ^{1}H NMR (400 MHz, Chloroform-*d*) δ 8.37 (d, *J* = 2.7 Hz, 1H), 7.65 (dd, *J* = 8.5, 2.7 Hz, 1H), 7.27 (d, *J* = 8.6 Hz, 1H), 7.10 (s, 4H), 7.06 (d, *J* = 7.3 Hz, 1H), 7.04 (d, *J* = 7.2 Hz, 1H), 6.99 (s, 1H), 6.92 (d, *J* = 6.8 Hz, 1H), 6.60 (t, *J* = 7.3 Hz, 1H), 6.45 (d, *J* = 7.7 Hz, 2H), 6.35 (dd, *J* = 16.9, 1.3 Hz, 1H), 6.22 (dd, *J* = 16.9, 10.1 Hz, 1H), 5.79 (dd, *J* = 10.1, 1.3 Hz, 1H), 5.58 (d, *J* = 6.8 Hz, 1H), 3.78 (s, 3H), 2.31 (s, 3H). ^{13}C NMR (101 MHz, $CDCl_3$) δ 170.5, 165.9, 151.1, 148.7, 146.3, 138.2, 138.0, 134.7, 130.8, 130.0, 129.5, 129.1, 129.0, 127.6, 124.0, 117.1, 112.6, 69.5, 61.9, 54.2, 21.2. **HRMS(ESI)** Calcd. for $C_{25}H_{25}ClN_3O_3$ $(M+H)^+$ 450.1579, found 450.1571.

Methyl2-acrylamido-3-((4-bromophenyl)amino)-2-(6-chloropyridin-3-yl)-3-phenylpropanoate (5e)

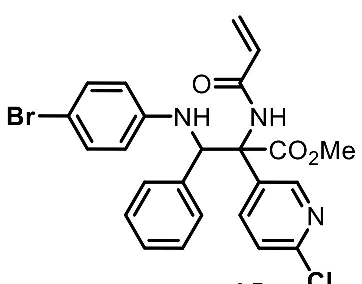

anti-**5e**

White solid, 79 mg, 77% yield, R_f = 0.3 (PE/EA = 5/1), **5e**: ^{1}H NMR (400 MHz, Chloroform-*d*) δ 8.37 (d, *J* = 2.7 Hz, 1H), 7.62 (dd, *J* = 8.5, 2.7 Hz, 1H), 7.33 (d, *J* = 1.5 Hz 1H), 7.31 (d, *J* = 1.8 Hz, 2H), 7.29 (s, 1H), 7.22 (d, *J* = 4.0 Hz, 1H), 7.20 (d, *J* = 4.9 Hz, 2H), 6.99 (s, 1H), 6.89 (t, *J* = 8.0 Hz, 1H), 6.72 (dd, *J* = 7.9, 0.9 Hz, 1H), 6.59 (t, *J* = 2.1 Hz, 1H), 6.38 (d, *J* = 2.0 Hz, 1H), 6.35 (dd, *J* = 7.4, 1.8 Hz, 1H), 6.21 (dd, *J* = 16.9, 10.1 Hz, 1H), 5.81 (dd, *J* = 10.1, 1.2 Hz, 1H), 5.61 (d, *J* = 7.0 Hz, 1H), 3.81 (s, 3H). ^{13}C NMR (101 MHz, $CDCl_3$) δ 170.2, 166.1, 151.3, 148.6, 147.5, 137.8, 137.2, 130.5, 130.5, 129.7, 129.4, 128.9, 128.7, 127.5, 124.1, 123.2, 120.0, 115.1, 111.4, 69.6, 61.8, 54.4. **HRMS(ESI)** Calcd. for $C_{24}H_{22}BrClN_3O_3$ $(M+H)^+$ 514.0528, found 514.0534.

Methyl2-acrylamido-3-(4-chlorophenyl)-3-((4-chlorophenyl)amino)-2-(6-chloropyridin-3-yl)propanoate (5f)

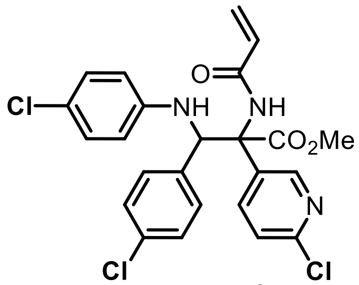

anti-**5f**

White solid, 75 mg, 74% yield, R_f = 0.3 (PE/EA = 5/1), **5f**: ^{1}H NMR (400 MHz, Chloroform-*d*) *δ* 8.36 (d, *J* = 2.7 Hz, 1H), 7.59 (dd, *J* = 8.5, 2.7 Hz, 1H), 7.31 (s, 1H), 7.29 (s, 2H), 7.15 (d, *J* = 8.5 Hz, 2H), 7.11 (d, *J* = 6.9 Hz, 1H), 7.01 (s, 1H), 6.99 (d, *J* = 1.7 Hz, 2H), 6.37 (d, *J* = 12.4 Hz, 1H), 6.34 (d, *J* = 5.5 Hz, 2H), 6.23 (dd, *J* = 16.8, 10.1 Hz, 1H), 5.83 (d, *J* = 11.3 Hz, 1H), 5.59 (d, *J* = 6.8 Hz, 1H), 3.80 (s, 3H). ^{13}C NMR (101 MHz, $CDCl_3$) *δ* 170.1, 166.1, 151.4, 148.6, 144.5, 137.7, 136.1, 134.5, 130.3, 129.6, 129.6, 129.1, 129.1, 128.9, 124.2, 122.0, 113.6, 69.5, 61.6, 54.5. **HRMS(ESI)** Calcd. for $C_{24}H_{21}Cl_3N_3O_3$ $(M+H)^+$ 504.0643, found 504.0645.

Methyl2-acrylamido-3-((4-chlorophenyl)amino)-2-(6-chloropyridin-3-yl)-3-(3,4-dichlorophenyl)propanoate (5g)

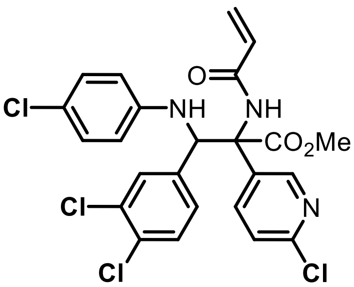

anti-**5g**

White solid, 76 mg, 71% yield, R_f = 0.3 (PE/EA = 5/1), **5g**: ^{1}H NMR (400 MHz, Chloroform-*d*) *δ* 8.34 (d, *J* = 2.8 Hz, 1H), 7.57 (dd, *J* = 8.5, 2.6 Hz, 1H), 7.40 (d, *J* = 8.2 Hz, 1H), 7.30 (d, *J* = 7.0 Hz, 2H), 7.12 (d, *J* = 6.7 Hz, 1H), 7.07 (d, *J* = 8.5 Hz, 1H), 7.03 (d, *J* = 9.1 Hz, 2H), 7.01 (s, 1H), 6.38 (d, *J* = 16.3 Hz, 2H), 6.34 (s, 1H), 6.24 (dd, *J* = 16.9, 10.1 Hz, 1H), 5.85 (d, *J* = 10.1 Hz, 1H), 5.56 (d, *J* = 6.7 Hz, 1H), 3.82 (s, 3H). ^{13}C NMR (101 MHz, $CDCl_3$) *δ* 167.0, 166.2, 151.5, 148.5, 144.3, 138.1, 137.6, 133.2, 132.9, 130.9, 130.1, 129.9, 129.6, 129.4, 129.2, 126.8, 124.2, 122.3, 113.6, 69.4, 61.4, 54.6. **HRMS(ESI)** Calcd. for $C_{24}H_{20}Cl_4N_3O_3$ $(M+H)^+$ 538.0253, found 538.0251.

Methyl2-acrylamido-3-(4-chlorophenyl)-2-(6-chloropyridin-3-yl)-3-(p-tolylamino)propanoate (5h)

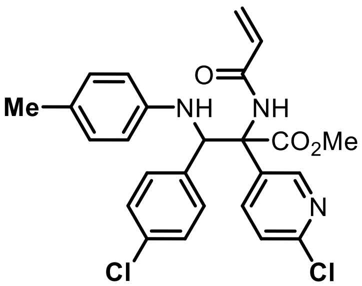

anti-**5h**

White solid, 73 mg, 75% yield, R_f = 0.3 (PE/EA = 5/1), **5h**: ^{1}H NMR (400 MHz, Chloroform-*d*) δ 8.35 (d, *J* = 2.7 Hz, 1H), 7.63 (dd, *J* = 8.5, 2.8 Hz, 1H), 7.30 – 7.27 (m, 2H), 7.26 (d, *J* = 3.5 Hz, 1H), 7.17 (d, *J* = 8.1 Hz, 2H), 6.99 (s, 1H), 6.87 (d, *J* = 7.9 Hz, 2H), 6.81 (d, *J* = 6.8 Hz, 1H), 6.36 (t, *J* = 6.6 Hz, 2H), 6.33 (s, 1H), 6.22 (dd, *J* = 16.8, 10.1 Hz, 1H), 5.80 (d, *J* = 10.1 Hz, 1H), 5.59 (d, *J* = 6.9 Hz, 1H), 3.78 (s, 3H), 2.17 (s, 3H). ^{13}C NMR (101 MHz, $CDCl_3$) δ 170.4, 165.9, 151.2, 148.6, 143.6, 138.0, 136.7, 134.2, 130.6, 129.8, 129.7, 129.3, 129.0, 129.0, 126.5, 124.1, 112.6, 69.4, 61.7, 54.4, 20.4. **HRMS(ESI)** Calcd. for $C_{25}H_{24}Cl_2N_3O_3$ $(M+H)^+$ 484.1189, found 484.1194.

Methyl2-acrylamido-3-(4-chlorophenyl)-2-(6-chloropyridin-3-yl)-3-((3,4-dichlorophenyl)amino)propanoate (5i)

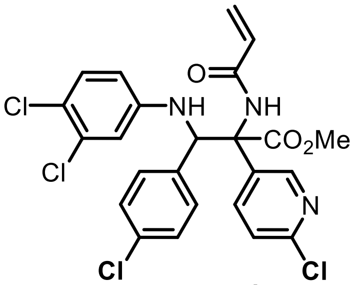

anti-**5i**

White solid, 83 mg, 77% yield, R_f = 0.3 (PE/EA = 5/1), **5i**: ^{1}H NMR (400 MHz, Chloroform-*d*) δ 8.35 (d, *J* = 2.7 Hz, 1H), 7.58 (dd, *J* = 8.5, 2.7 Hz, 1H), 7.32 (s, 2H), 7.30 (s, 2H), 7.14 (d, *J* = 8.2 Hz, 2H), 7.08 (d, *J* = 8.8 Hz, 1H), 7.00 (s, 1H), 6.48 (d, *J* = 2.7 Hz, 1H), 6.38 (d, *J* = 16.9 Hz, 1H), 6.30 – 6.27 (m, 1H), 6.25 – 6.19 (m, 1H), 5.85 (d, *J* = 10.1 Hz, 1H), 5.58 (d, *J* = 6.9 Hz, 1H), 3.82 (s, 3H). ^{13}C NMR (101 MHz, $CDCl_3$) δ 169.9, 166.2, 151.5, 148.5, 145.4, 137.6, 135.5, 134.7, 132.8, 130.7, 130.1, 129.9, 129.5, 129.2, 128.8, 124.2, 120.0, 113.5, 112.5, 69.5, 61.4, 54.6. **HRMS(ESI)** Calcd. for $C_{24}H_{20}Cl_4N_3O_3$ $(M+H)^+$ 538.0253, found 538.0251.

Methyl2-acrylamido-3-((4-chlorophenyl)amino)-2-(6-chloropyridin-3-yl)-3-(4-methoxyphenyl)propanoate (5j)

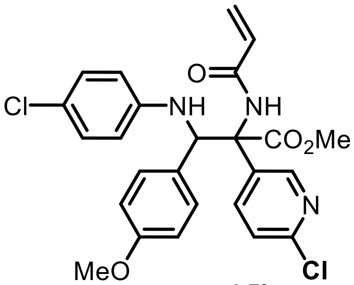

anti-**5j**

White solid, 80 mg, 80% yield, R_f = 0.3 (PE/EA = 5/1), **5j**: ^{1}H NMR (400 MHz, Chloroform-*d*) δ 8.36 (d, *J* = 2.7 Hz, 1H), 7.62 (dd, *J* = 8.5, 2.7 Hz, 1H), 7.29 (d, *J* = 2.3 Hz, 2H), 7.27 (d, *J* = 2.6 Hz, 1H), 7.17 (d, *J* = 8.2 Hz, 2H), 6.99 (s, 1H), 6.67 (s, 1H), 6.65 (t, *J* = 3.3 Hz, 2H), 6.38 (d, *J* = 2.8 Hz, 2H), 6.34 (d, *J* = 10.9 Hz, 1H), 6.22 (dd, *J* = 16.9, 10.1 Hz, 1H), 5.81 (dd, *J* = 10.0, 1.3 Hz, 1H), 5.54 (d, *J* = 6.7 Hz, 1H), 3.78 (s, 3H), 3.68 (s, 3H). ^{13}C NMR (101 MHz, $CDCl_3$) δ 170.4, 165.9, 151.8, 151.2, 148.6, 140.2, 137.9, 136.8, 134.2, 130.6, 129.8, 129.3, 129.1, 129.0, 124.1, 114.9, 113.5, 69.5, 62.3, 55.8, 54.4. **HRMS(ESI)** Calcd. for $C_{25}H_{24}Cl_2N_3O_4$ $(M+H)^+$ 500.1138, found 500.1133.

Methyl2-acrylamido-3-((4-chlorophenyl)amino)-2-(6-chloropyridin-3-yl)-3-(thiophen-2-yl)propanoate (5k)

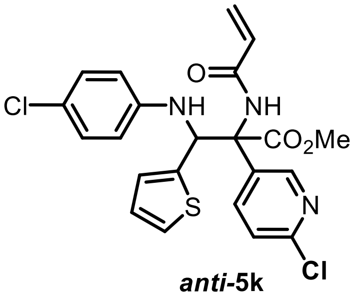

anti-**5k**

White solid, 66 mg, 69% yield, R_f = 0.3 (PE/EA = 5/1), **5k**: ^{1}H NMR (400 MHz, Chloroform-*d*) δ 8.35 (d, *J* = 2.7 Hz, 1H), 7.58 (dd, *J* = 8.5, 2.7 Hz, 1H), 7.29 (d, *J* = 8.5 Hz, 1H), 7.25 (d, *J* = 5.2 Hz, 1H), 7.15 (d, *J* = 7.2 Hz, 2H), 7.04 (d, *J* = 8.7 Hz, 2H), 6.96 (t,*J* = 7.2 Hz 1H), 6.89 (d, *J* = 3.5 Hz, 1H), 6.44 (d, *J* = 8.8 Hz, 2H), 6.36 (d, *J* = 16.9 Hz, 1H), 6.26 (dd, *J* = 16.9, 9.9 Hz, 1H), 5.90 (d, *J* = 6.4 Hz, 1H), 5.81 (dd, *J* = 9.9, 1.4 Hz, 1H), 3.82 (s, 3H). ^{13}C NMR (101 MHz, $CDCl_3$) δ 170.1, 166.1, 151.4, 148.4, 144.7, 142.0, 137.5, 130.1, 129.8, 129.4, 129.1, 127.1, 126.1, 125.9, 124.2, 122.2, 113.7, 69.8, 58.7, 54.6. **HRMS(ESI)** Calcd. for $C_{22}H_{20}Cl_2N_3O_3S$ $(M+H)^+$ 476.0597, found 476.0593.

Methyl2-acrylamido-2-(6-chloropyridin-3-yl)-3-((3,4-dichlorophenyl)amino)-3-(thiophen-2-yl)propanoate (5l)

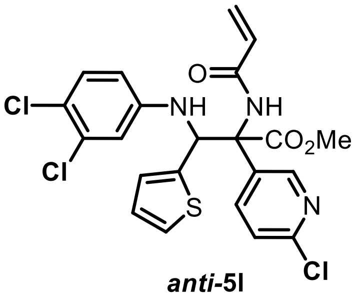

anti-**5l**

White solid, 67 mg, 66% yield, R_f = 0.3 (PE/EA = 5/1), **5l**: ^{1}H NMR (400 MHz, Chloroform-*d*) δ 8.35 (d, *J* = 2.6 Hz, 1H), 7.57 (dd, *J* = 8.5, 2.7 Hz, 1H), 7.38 (d, *J* = 6.5 Hz, 1H), 7.30 (d, *J* = 8.5 Hz, 1H), 7.27 (d, *J* = 4.1 Hz, 1H), 7.15 (s, 1H), 7.11 (d, *J* = 8.7 Hz, 1H), 6.98 (dd, *J* = 5.1, 3.5 Hz, 1H), 6.89 (d, *J* = 3.5 Hz, 1H), 6.59 (d, *J* = 2.7 Hz, 1H), 6.39 (dd, *J* = 6.7, 2.0 Hz, 1H), 6.36 (d, *J* = 2.6 Hz, 1H), 6.26 (dd, *J* = 16.9, 10.0 Hz, 1H), 5.89 (d, *J* = 6.5 Hz, 1H), 5.83 (dd, *J* = 9.9, 1.3 Hz, 1H), 3.84 (s, 3H). ^{13}C NMR (101 MHz, $CDCl_3$) δ 169.9, 166.2, 151.5, 148.4, 145.7, 141.2, 137.4, 132.8, 130.7, 129.9, 129.7, 129.6, 127.2, 126.3, 126.1, 124.3, 120.2, 113.6, 112.6, 69.8, 58.5, 54.7. **HRMS(ESI)** Calcd. for $C_{22}H_{19}Cl_3N_3O_3S$ $(M+H)^+$ 510.0207, found 510.0211.

Methyl2-acrylamido-2-(6-chloropyridin-3-yl)-3-(thiophen-2-yl)-3-((4-(trifluoromethyl)phenyl)amino)propanoate (5m)

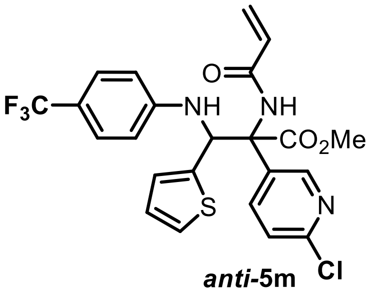

anti-**5m**

White solid, 65 mg, 64% yield, R_f = 0.3 (PE/EA = 5/1), **5m**: ^{1}H NMR (400 MHz, Chloroform-*d*) δ 8.25 (d, *J* = 2.7 Hz, 1H), 7.75 (dd, *J* = 8.5, 2.7 Hz, 1H), 7.40 (d, *J* = 8.3 Hz, 2H), 7.29 (d, *J* = 3.1 Hz, 1H), 7.27 (d, *J* = 3.8 Hz, 1H), 7.05 (s, 1H), 6.97 (dd, *J* = 5.1, 3.6 Hz, 1H), 6.88 (d, *J* = 3.6 Hz, 1H), 6.66 (d, *J* = 8.4 Hz, 2H), 6.45 (d, *J* = 8.9 Hz, 1H), 6.33 (d, *J* = 16.9 Hz, 1H), 6.17 (dd, *J* = 16.9, 10.2 Hz, 1H), 5.76 (d, *J* = 10.2 Hz, 1H), 5.37 (d, *J* = 8.9 Hz, 1H), 3.96 (s, 3H). ^{13}C NMR (101 MHz, $CDCl_3$) δ 170.3, 164.5, 151.3, 149.0, 147.8, 140.5, 139.4, 131.1, 130.1, 128.6, 127.2, 127.0, 126.9, 126.8, 126.0, 123.4, 112.9, 67.8, 54.4, 53.8. **HRMS(ESI)** Calcd. for $C_{23}H_{20}ClF_3N_3O_3S$ $(M+H)^+$ 510.0861, found 510.0865.

4-ethyl 1-methyl 2-((2-chlorobenzyl)oxy)-2-(2,4-dichlorophenyl)-3-(phenylamino)succinate (6)

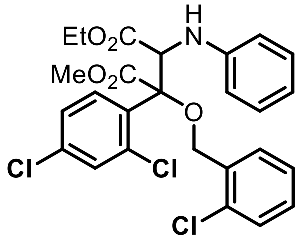

***anti*-6**

White solid, 76 mg, 71% yield, R_f = 0.3 (PE/EA = 5/1), **6**: ^{1}H NMR (400 MHz, Chloroform-*d*) δ 7.64 (d, J = 7.55 Hz, 1H), 7.52 (d, J = 8.59 Hz, 1H), 7.40 (d, J = 2.25 Hz, 1H), 7.32 (d, J = 7.71 Hz, 1H), 7.24 (dd, J = 17.32, 8.60, 4.70 Hz, 3H), 7.11 (t, J = 7.64 Hz, 2H), 6.70 (dd, J = 11.99, 7.54 Hz, 3H), 5.20 (d, J = 8.89 Hz, 1H), 4.83 (d, J = 12.74 Hz, 1H), 4.66 (d, J = 12.74 Hz, 1H), 4.39 (d, J = 8.92 Hz, 1H), 4.11 (dd, J = 7.17, 4.60 Hz, 2H), 3.72 (s, 3H), 1.11 (t, J = 7.13 Hz, 3H). ^{13}C NMR (101 MHz, $CDCl_3$) δ 170.5, 168.9, 146.3, 135.3, 135.1, 133.8, 133.0, 132.1, 131.1, 130.6, 129.1, 129.0, 128.8, 128.7, 126.9, 126.8, 119.8, 118.8, 114.3, 85.6, 65.7, 61.4, 61.1, 52.7, 13.9. **HRMS(ESI)** Calcd. for $C_{26}H_{25}Cl_3NO_5$ $(M+H)^+$ 536.0793, found 536.0799.

methyl 2-acrylamido-2-(3,5-dichlorophenyl)acetate (7)

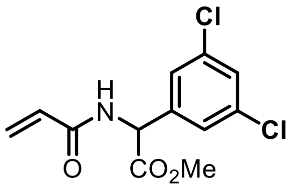

7

White solid, 55 mg, 95% yield, R_f = 0.3 (PE/EA = 3/1), **7**: ^{1}H NMR (400 MHz, Chloroform-*d*) δ 7.40 (d, J = 2.10 Hz, 1H), 7.36 (d, J = 8.29 Hz, 1H), 7.24 (dd, J = 8.30, 2.06 Hz, 1H), 6.98 (d, J = 6.96 Hz, 1H), 6.35 – 6.27 (m, 1H), 6.18 (dd, J = 16.95, 10.09 Hz, 1H), 5.94 (d, J = 6.94 Hz, 1H), 5.71 – 5.64 (m, 1H), 3.75 (s, 3H). ^{13}C NMR (101 MHz, $CDCl_3$) δ 170.4, 164.7, 135.0, 134.2, 133.3, 131.3, 129.9, 129.7, 127.9, 127.5, 54.2, 53.2. **HRMS(ESI)** Calcd. for $C_{12}H_{12}Cl_2NO_3$ $(M+H)^+$ 288.0189, found 288.0186.

NMR Spectra of Compounds

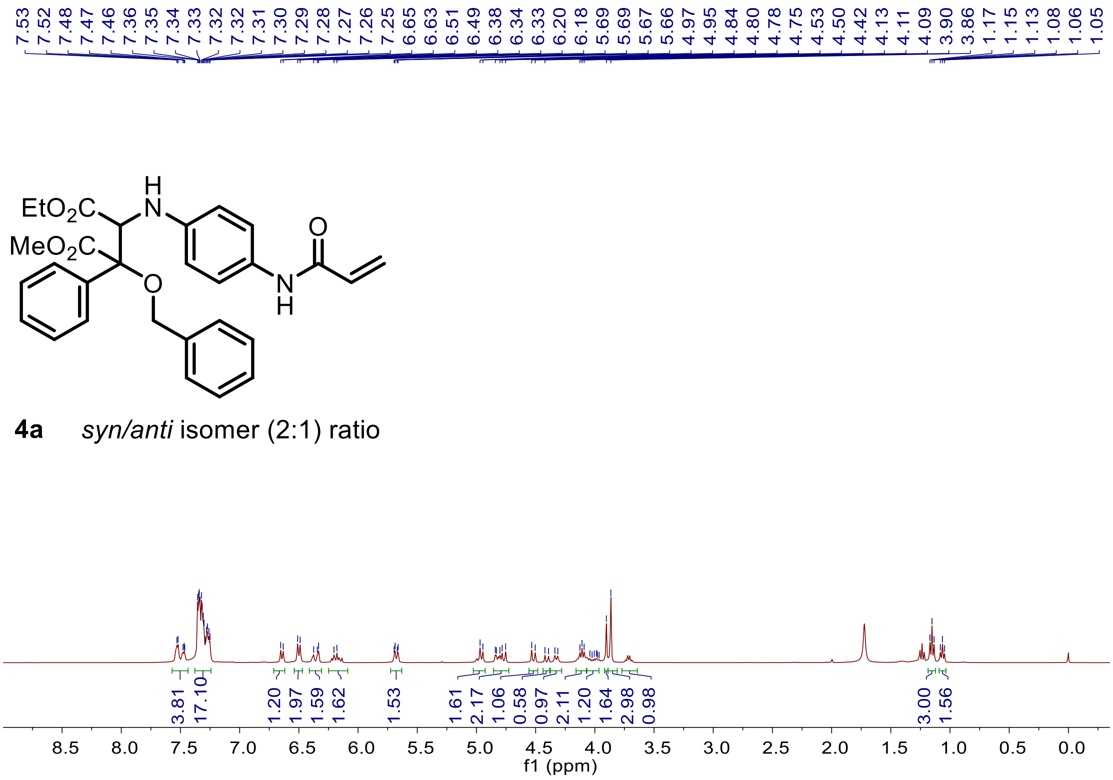

^{1}H NMR Spectrum of Compound **4a** (400MHz, $CDCl_3$)

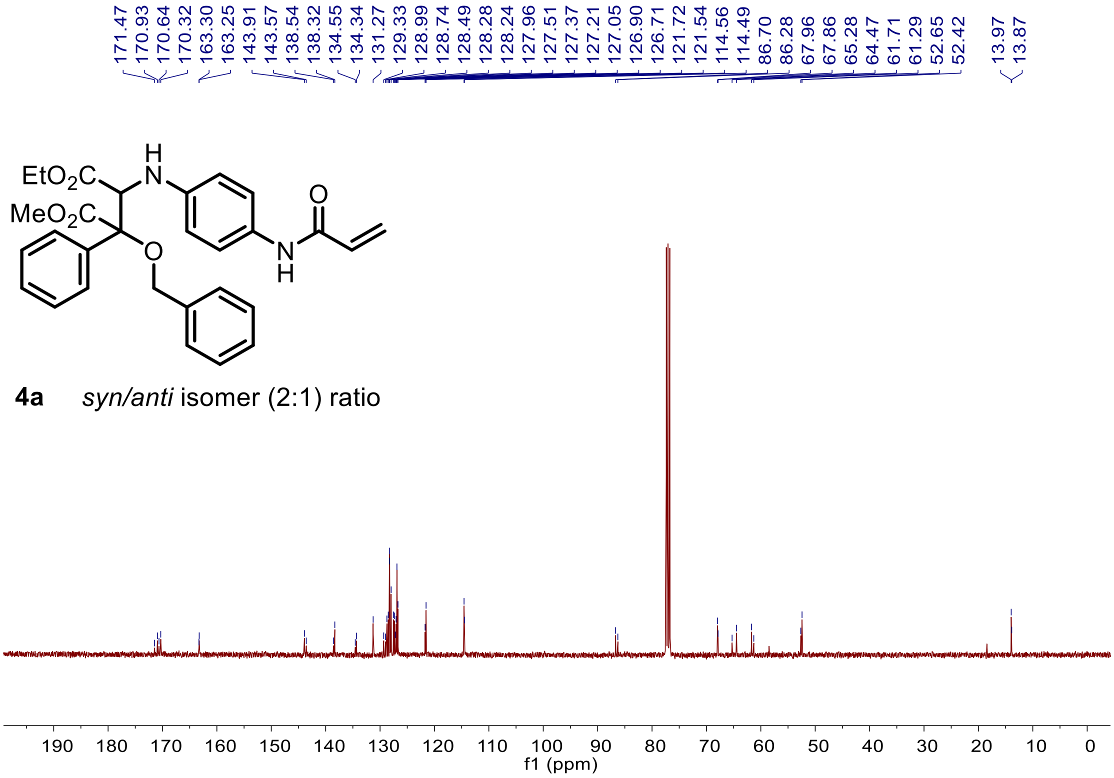

^{13}C NMR Spectrum of Compound **4a** (101MHz, $CDCl_3$)

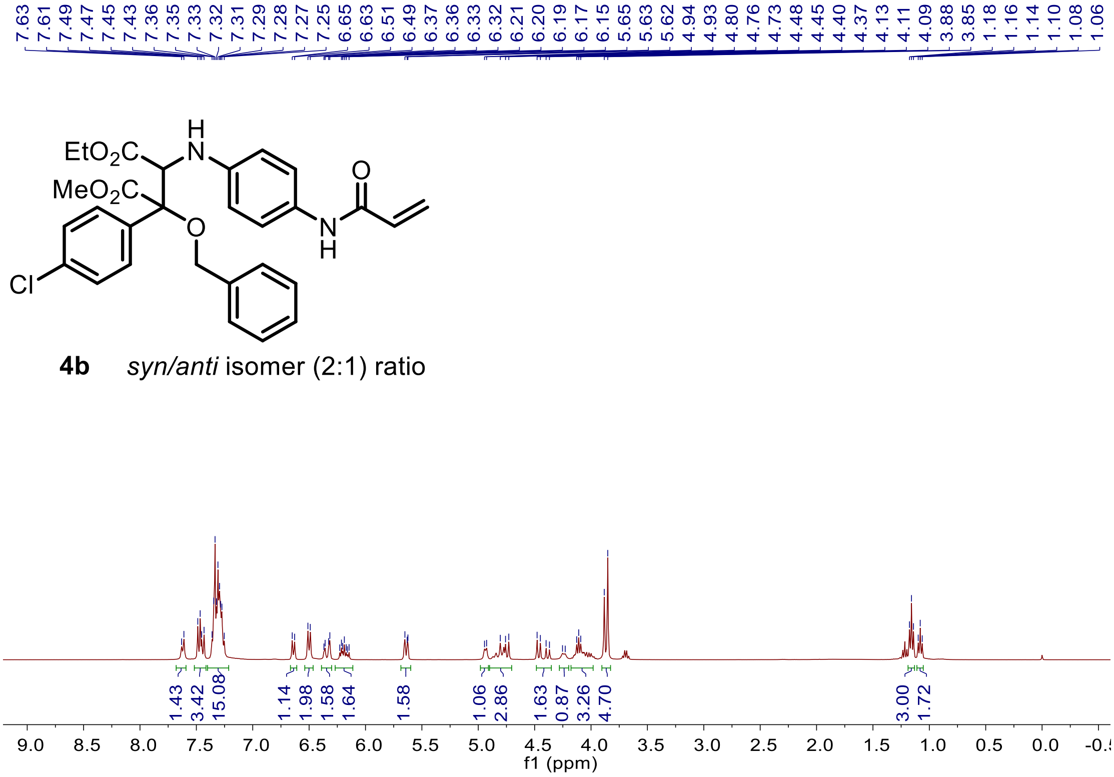

^{1}H NMR Spectrum of Compound **4b** (400MHz, $CDCl_3$)

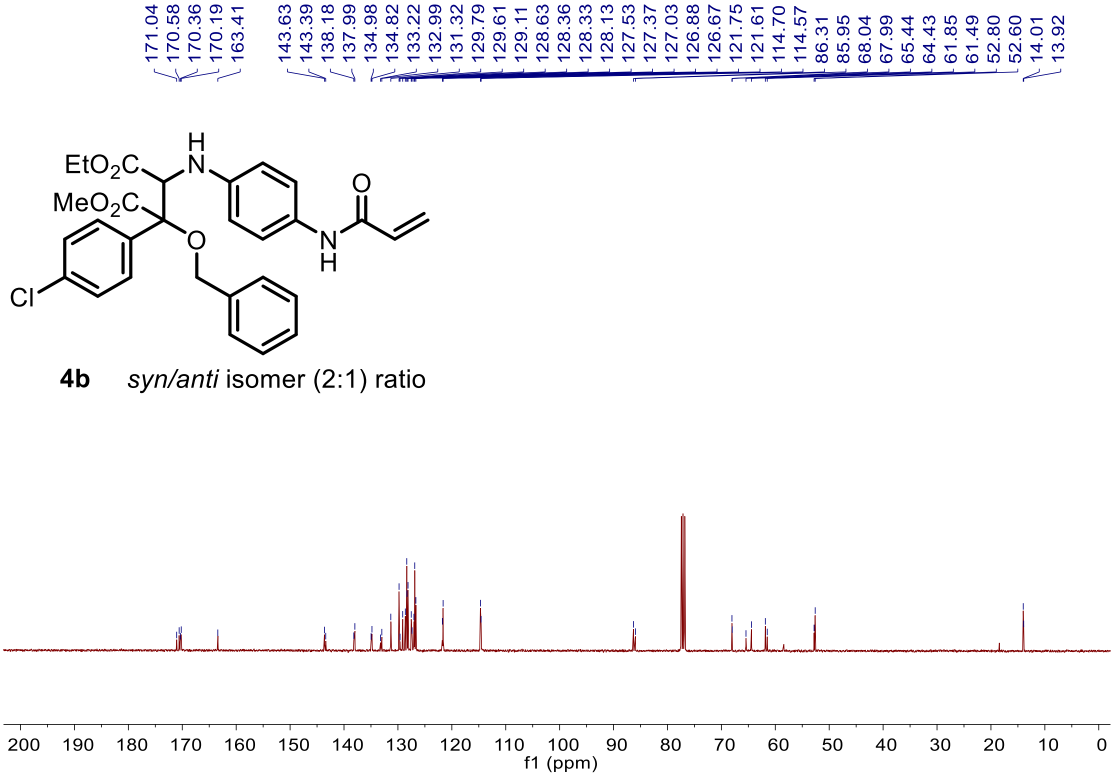

^{13}C NMR Spectrum of Compound **4b** (101MHz, $CDCl_3$)

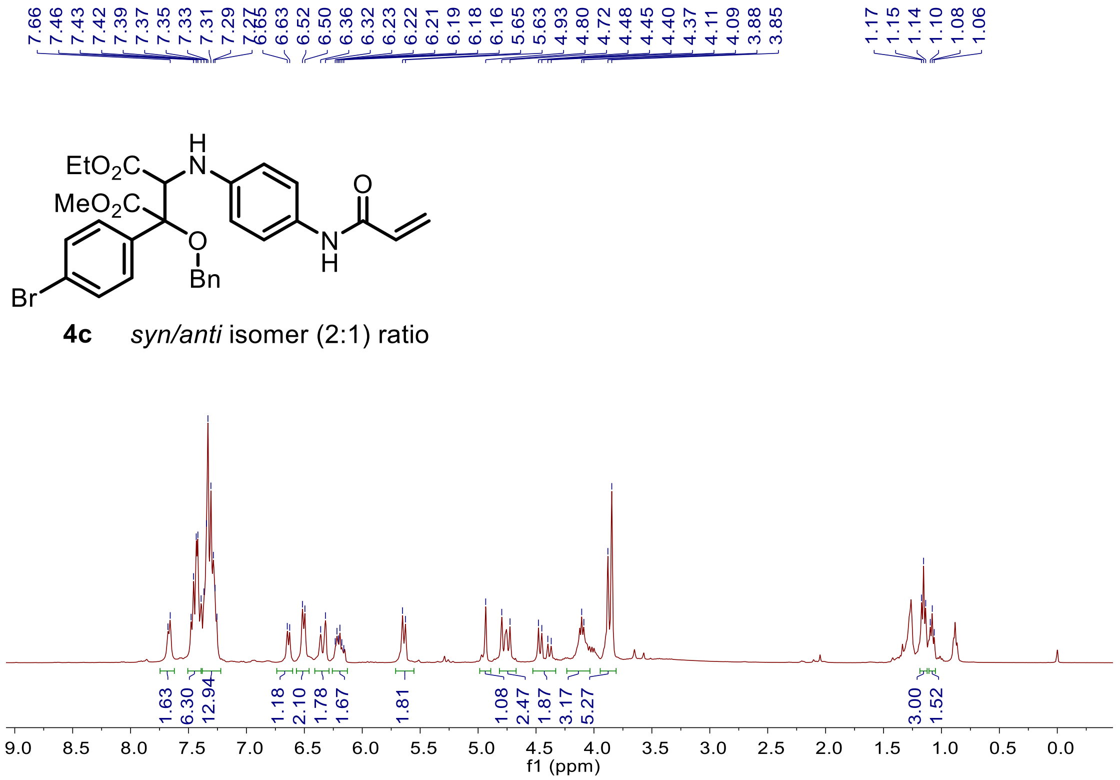

^{1}H NMR Spectrum of Compound **4c** (400MHz, $CDCl_3$)

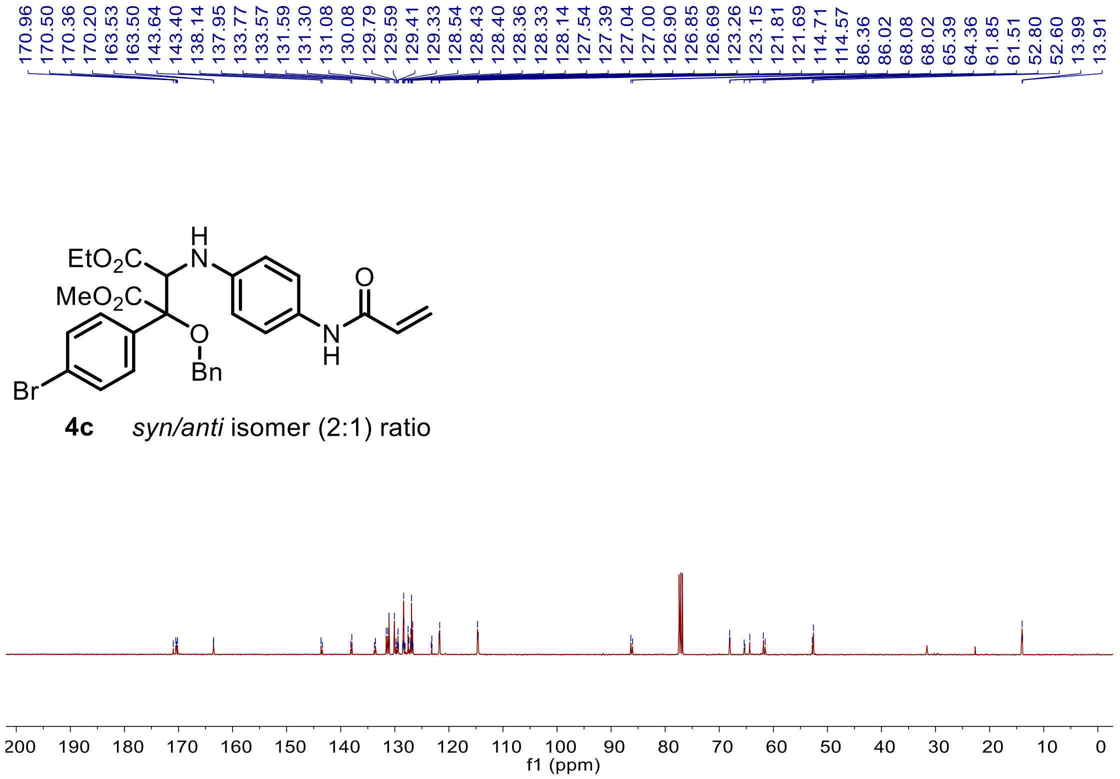

^{13}C NMR Spectrum of Compound **4c** (101MHz, $CDCl_3$)

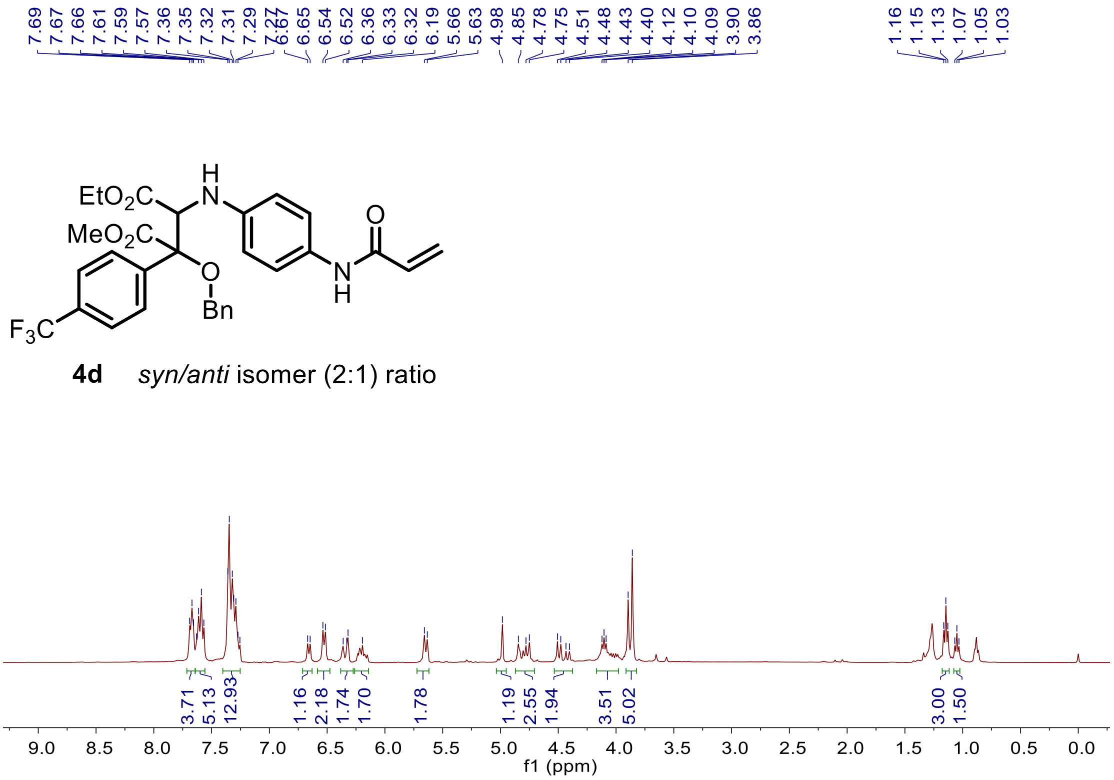

^{1}H NMR Spectrum of Compound **4d** (400MHz, $CDCl_3$)

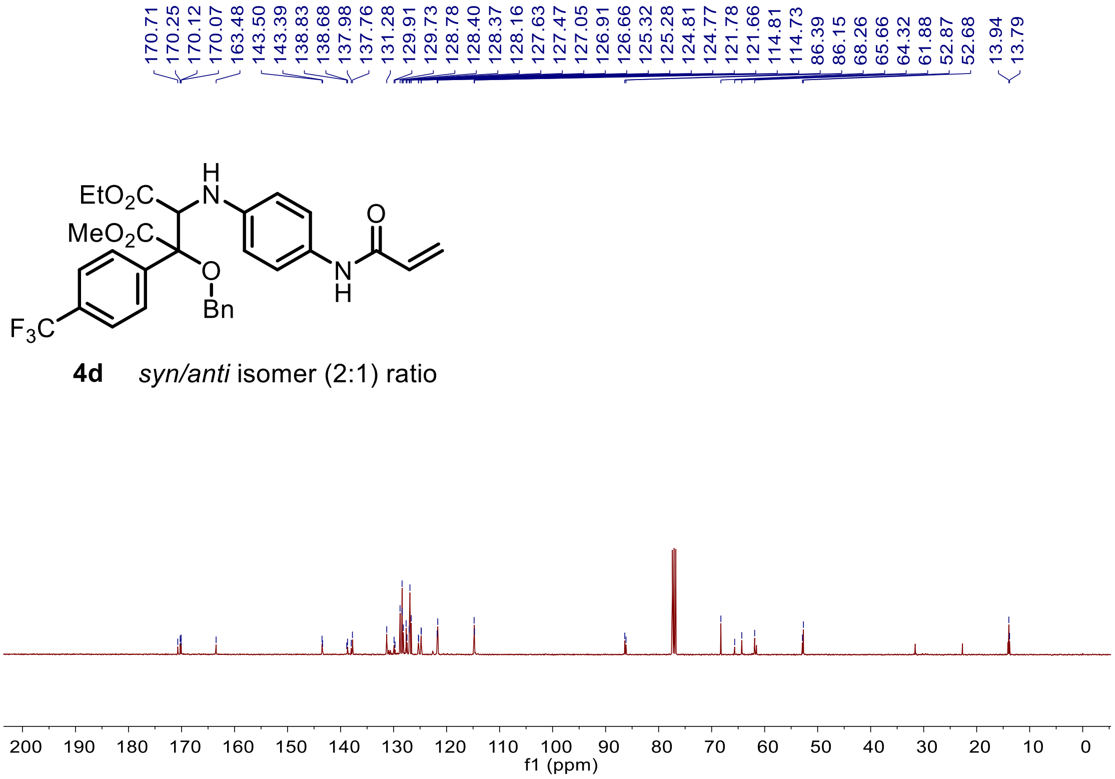

^{13}C NMR Spectrum of Compound **4d** (101MHz, $CDCl_3$)

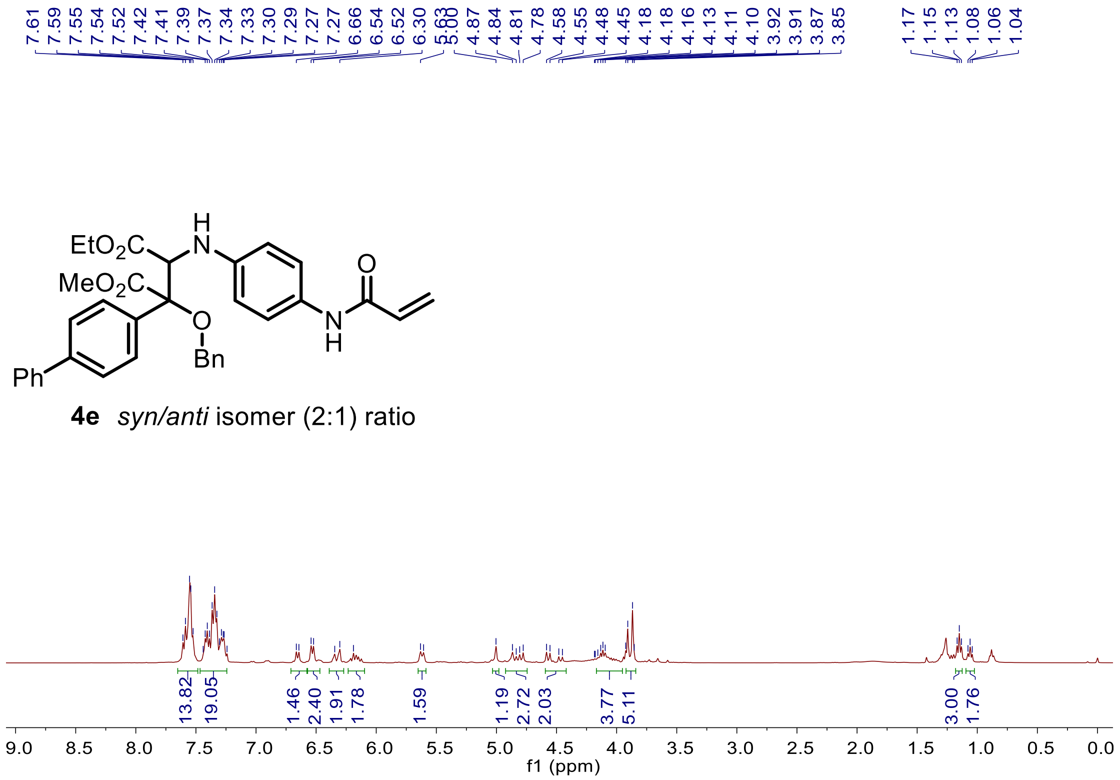

^{1}H NMR Spectrum of Compound **4e** (400MHz, $CDCl_3$)

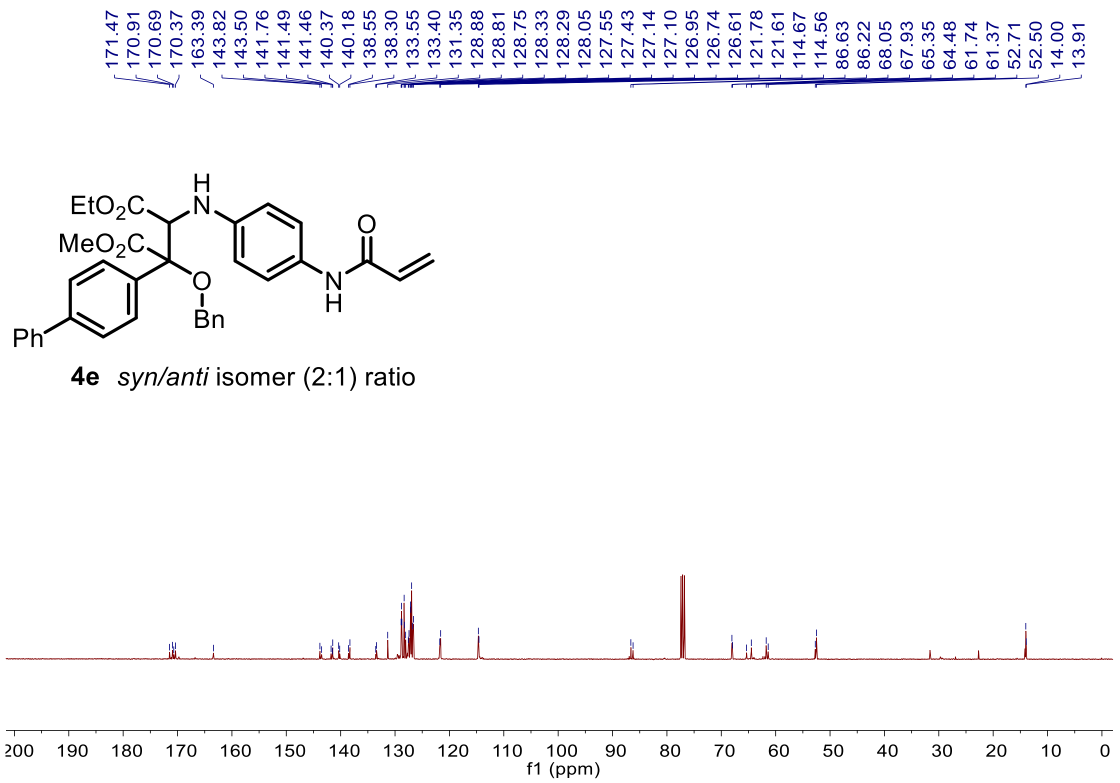

^{13}C NMR Spectrum of Compound **4e** (101MHz, $CDCl_3$)

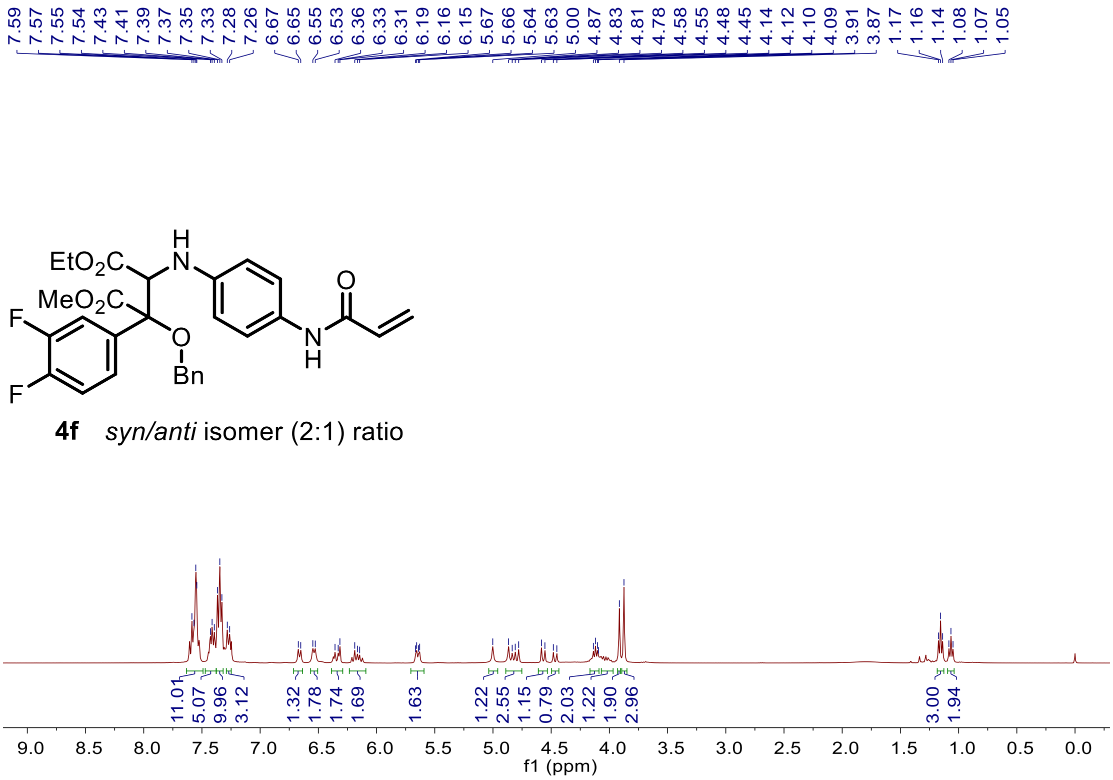

^{1}H NMR Spectrum of Compound **4f** (400MHz, $CDCl_3$)

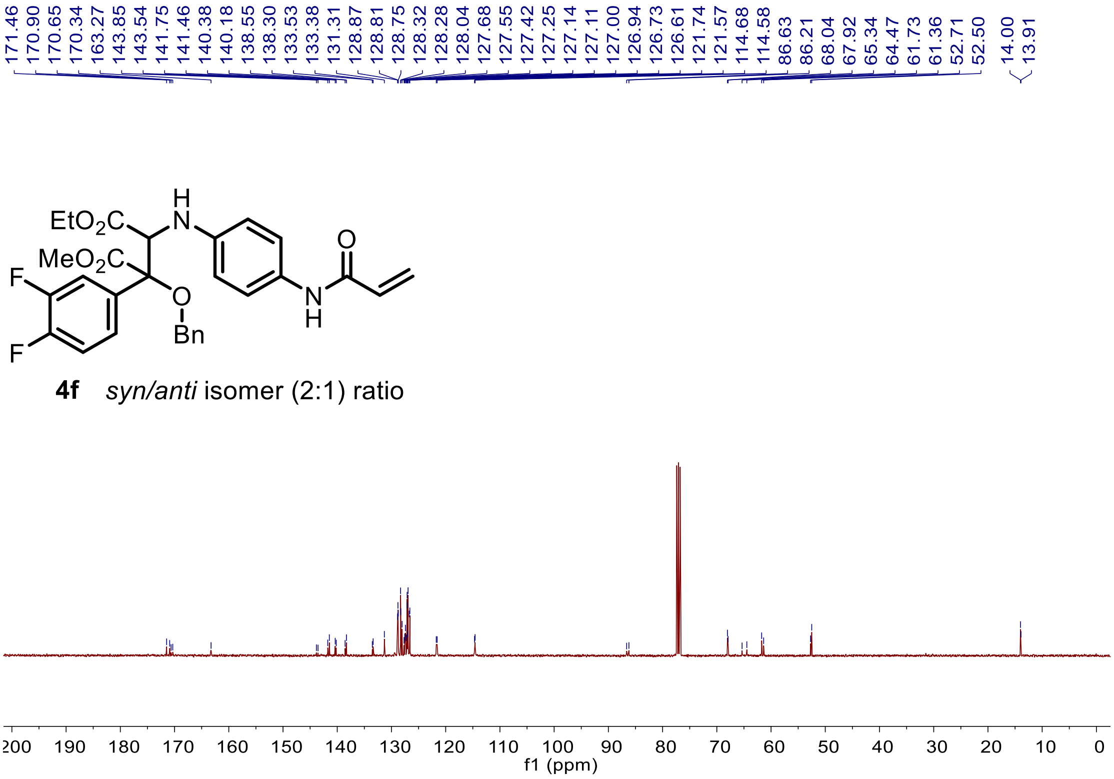

^{13}C NMR Spectrum of Compound **4f** (101MHz, $CDCl_3$)

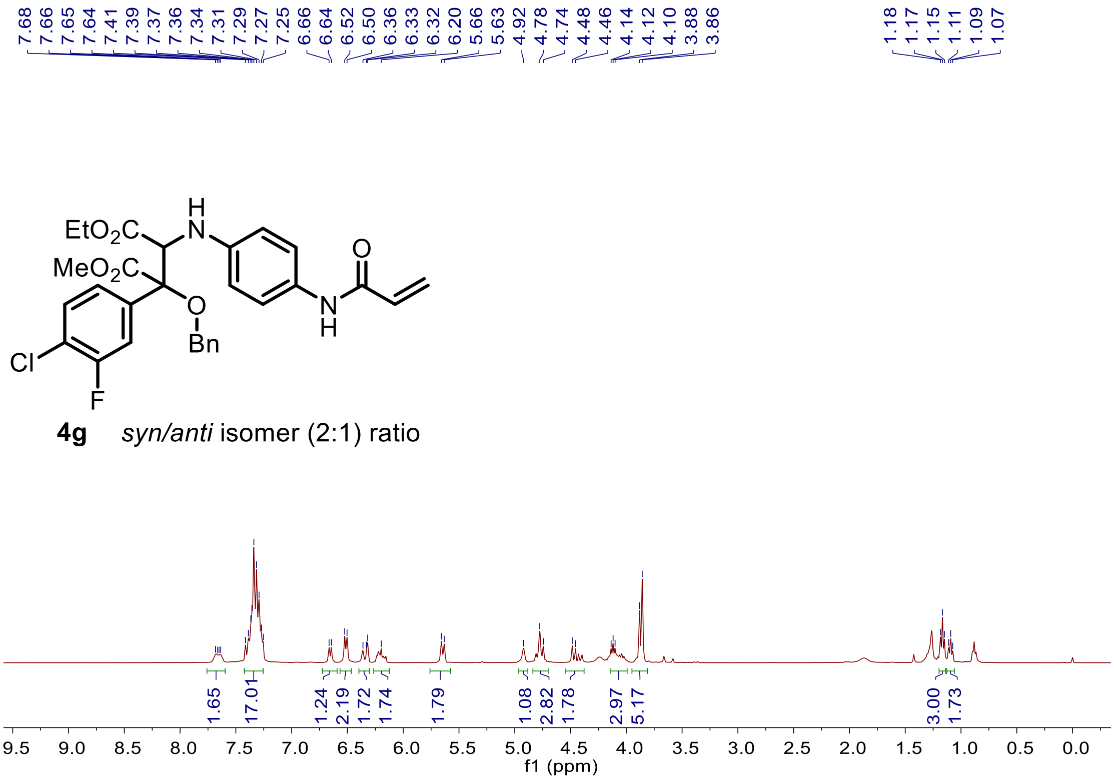

^{1}H NMR Spectrum of Compound **4g** (400MHz, $CDCl_3$)

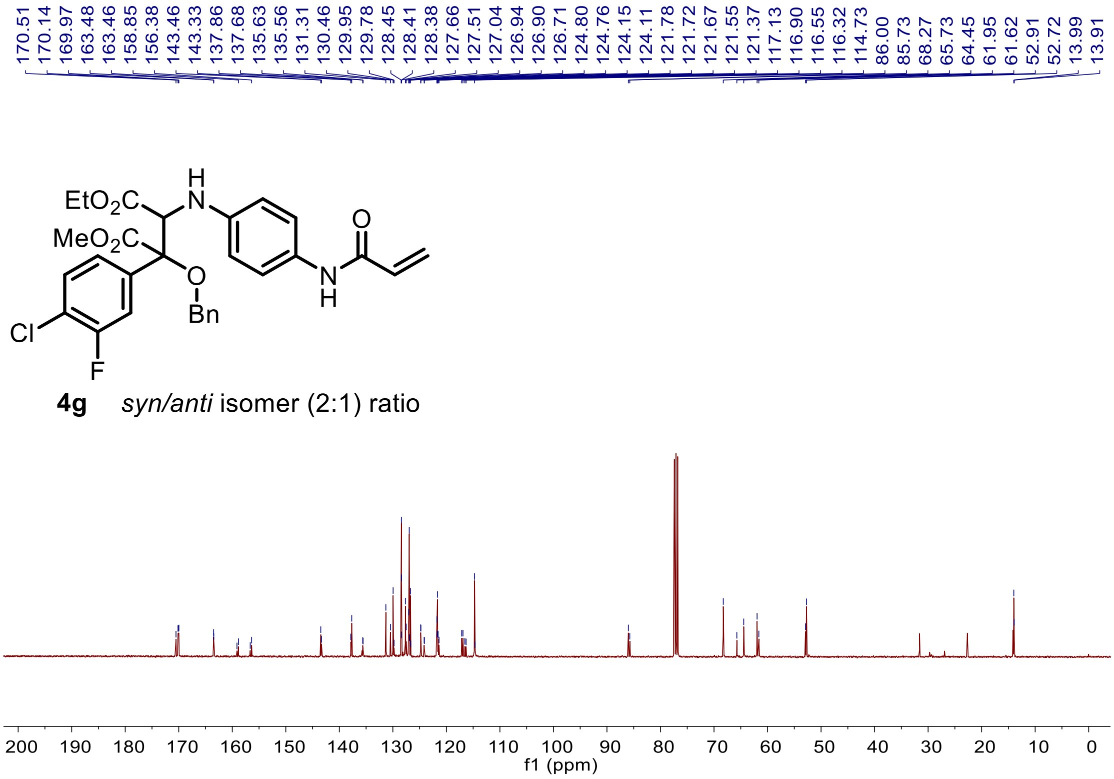

^{13}C NMR Spectrum of Compound **4g** (101MHz, $CDCl_3$)

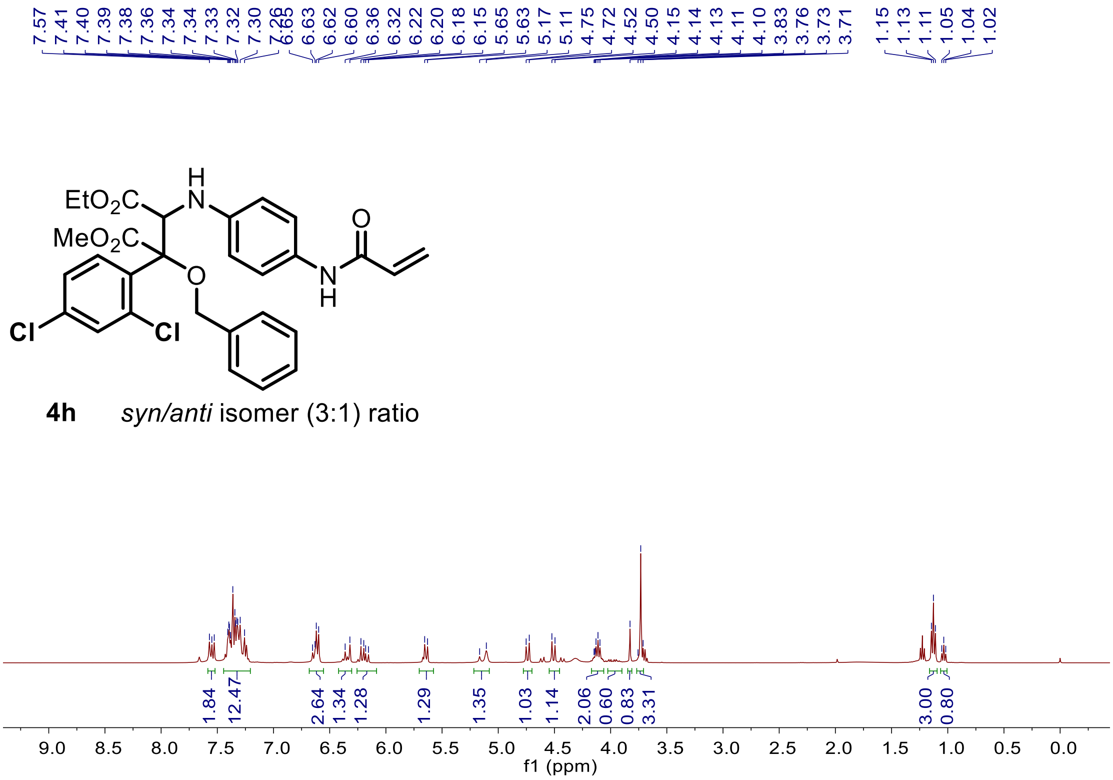

^{1}H NMR Spectrum of Compound **4h** (400MHz, $CDCl_3$)

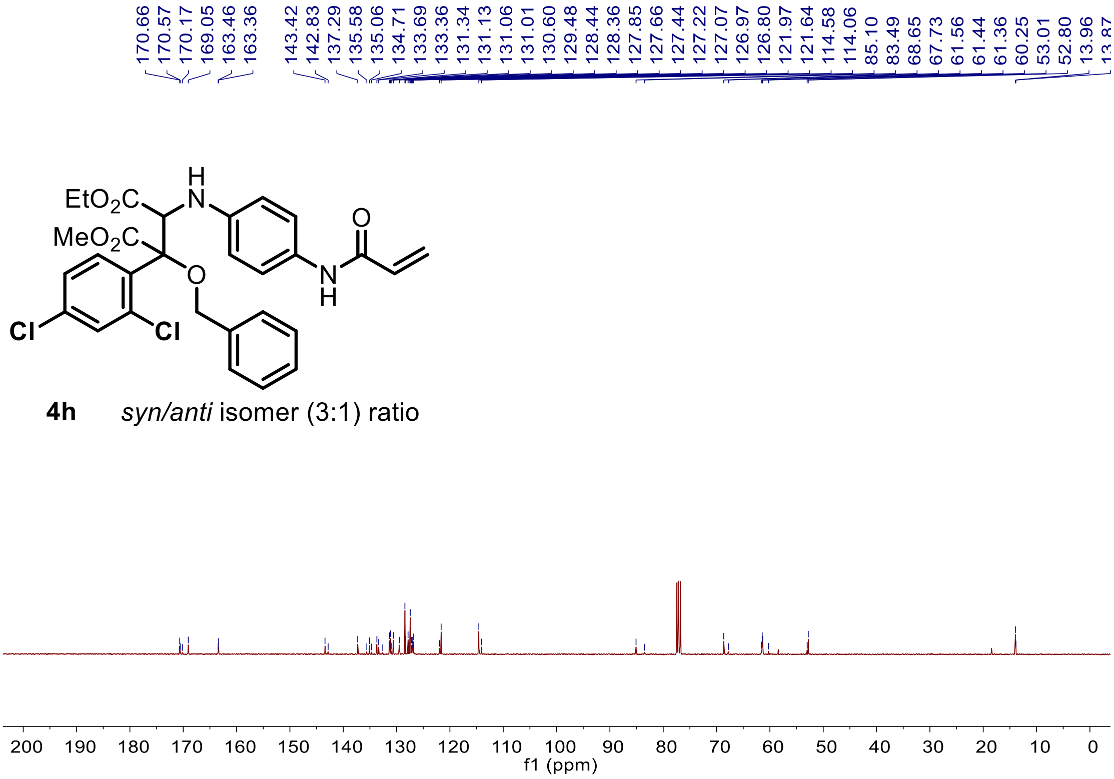

^{13}C NMR Spectrum of Compound **4h** (101MHz, $CDCl_3$)

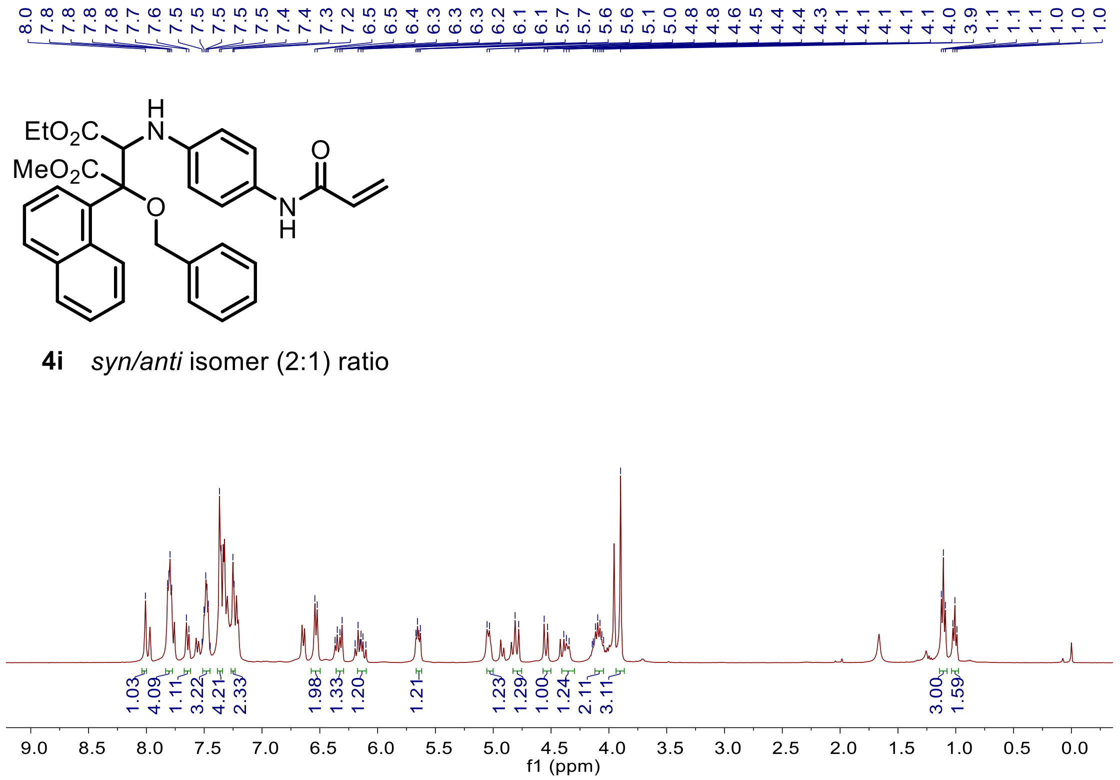

^{1}H NMR Spectrum of Compound **4i** (400MHz, $CDCl_3$)

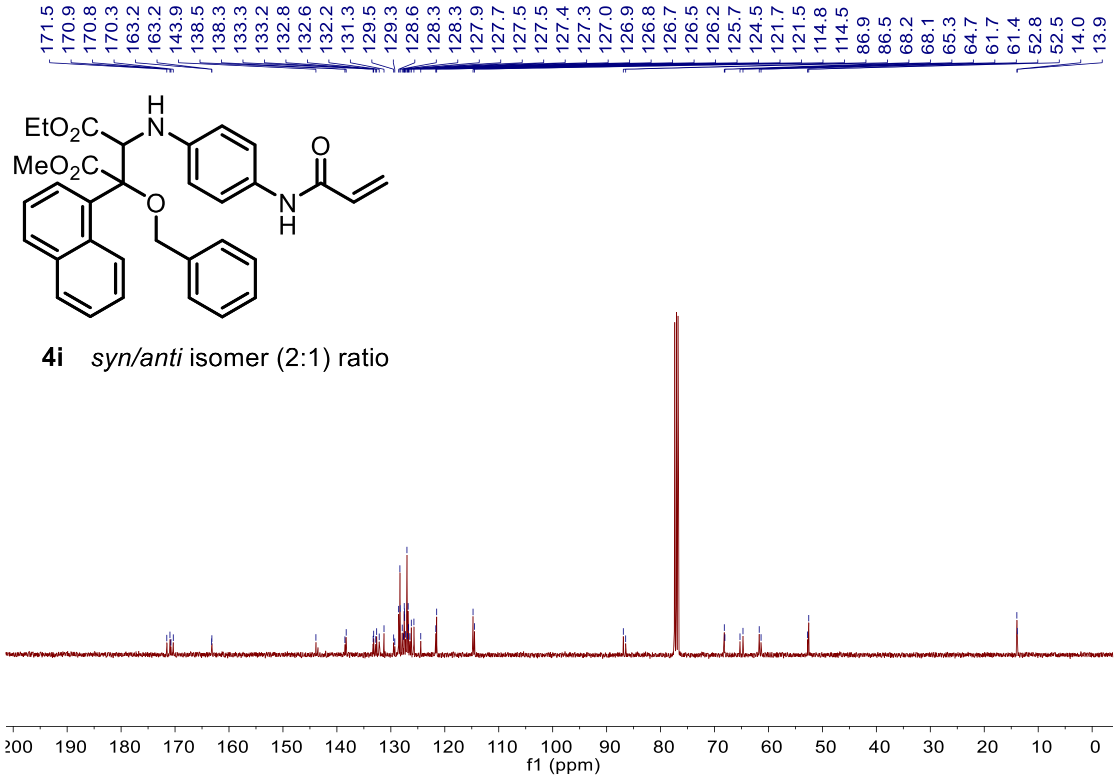

^{13}C NMR Spectrum of Compound **4i** (101MHz, $CDCl_3$)

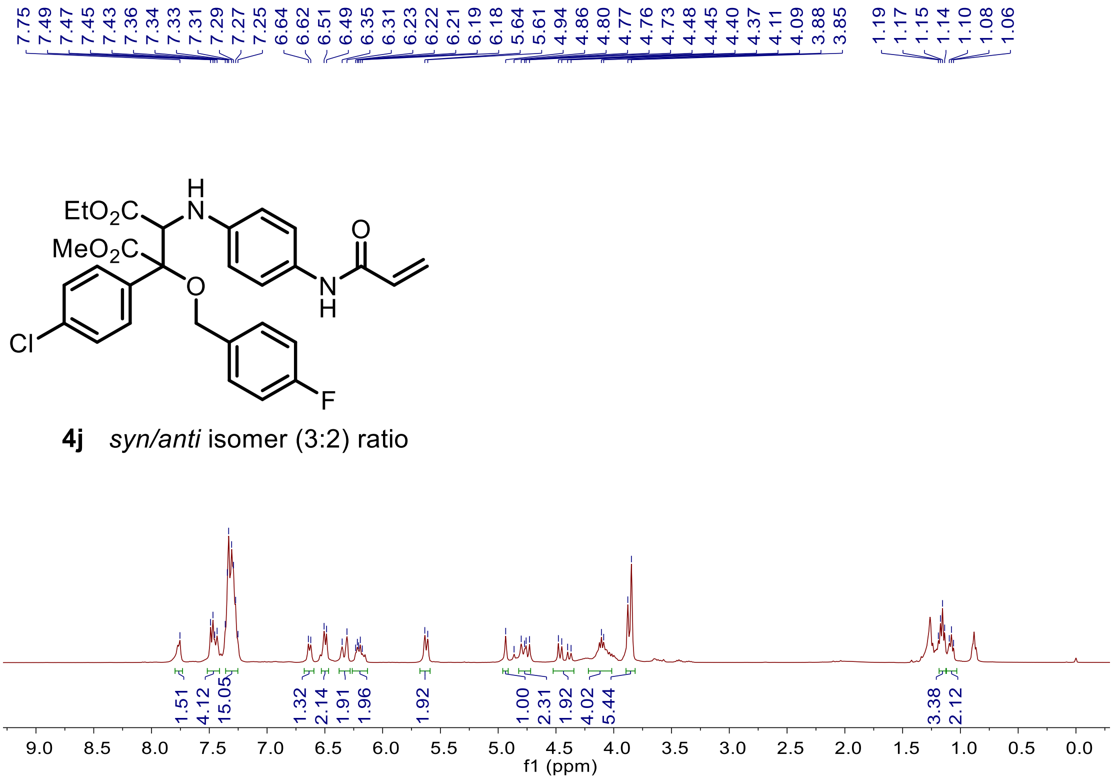

^{1}H NMR Spectrum of Compound **4j** (400MHz, $CDCl_3$)

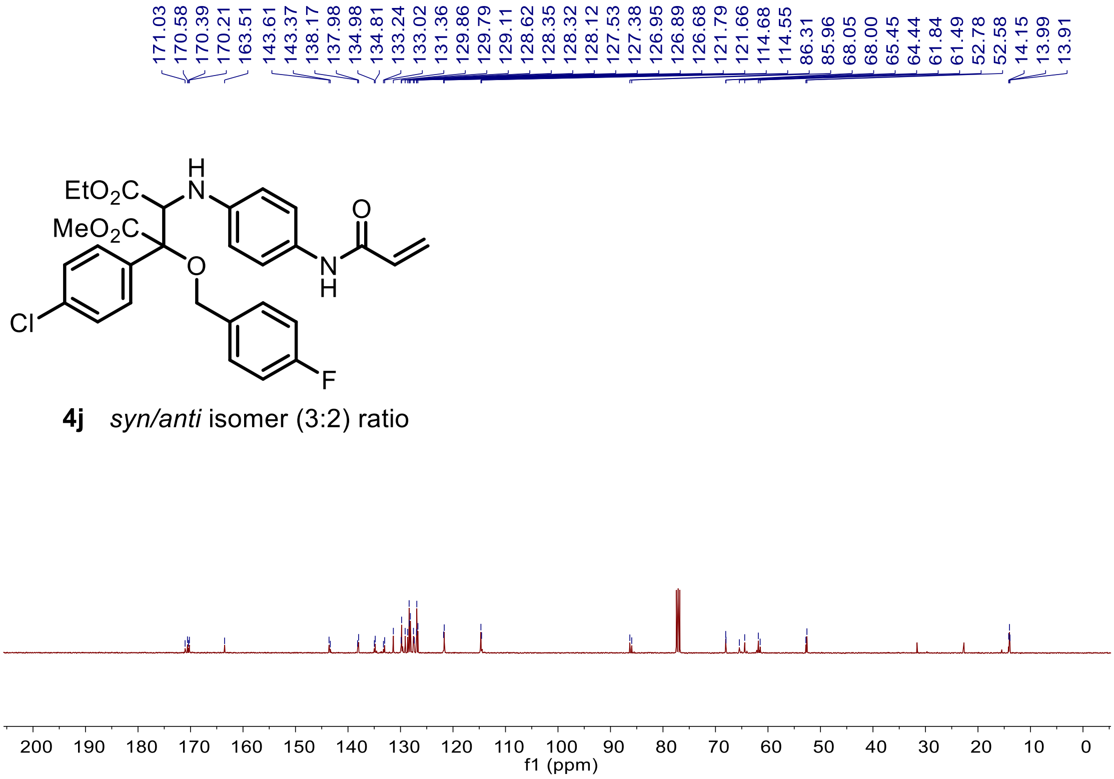

^{13}C NMR Spectrum of Compound **4j** (101MHz, $CDCl_3$)

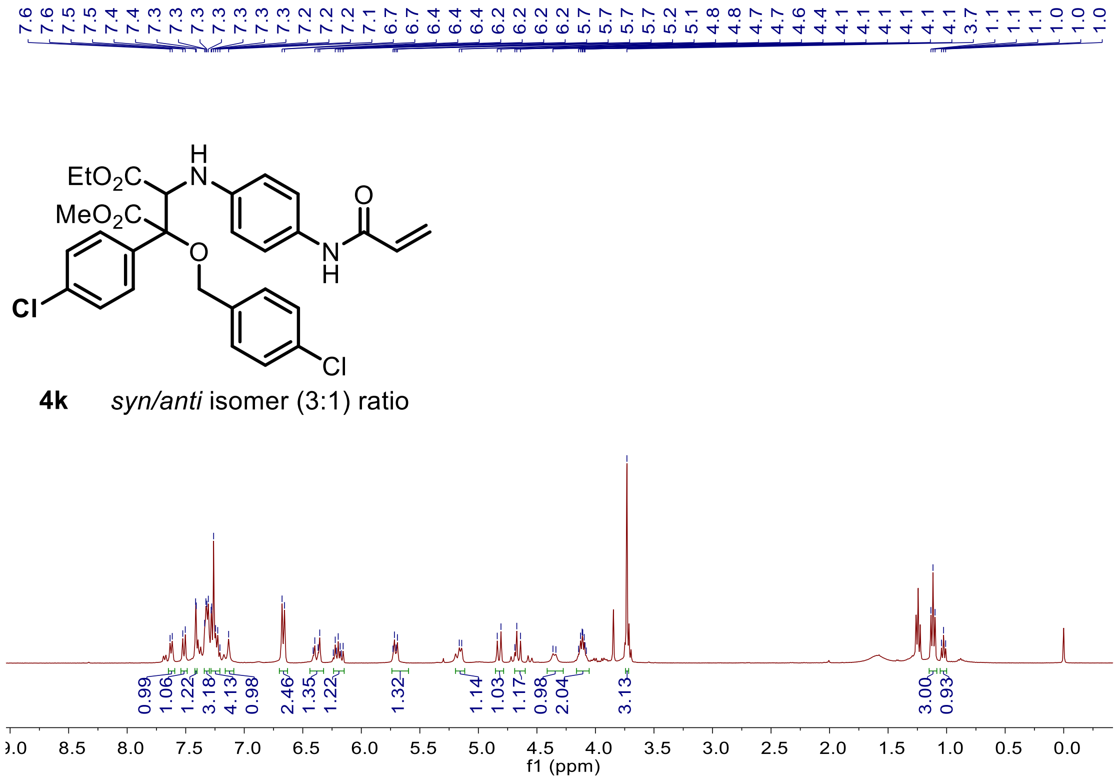

^{1}H NMR Spectrum of Compound **4k** (400MHz, $CDCl_3$)

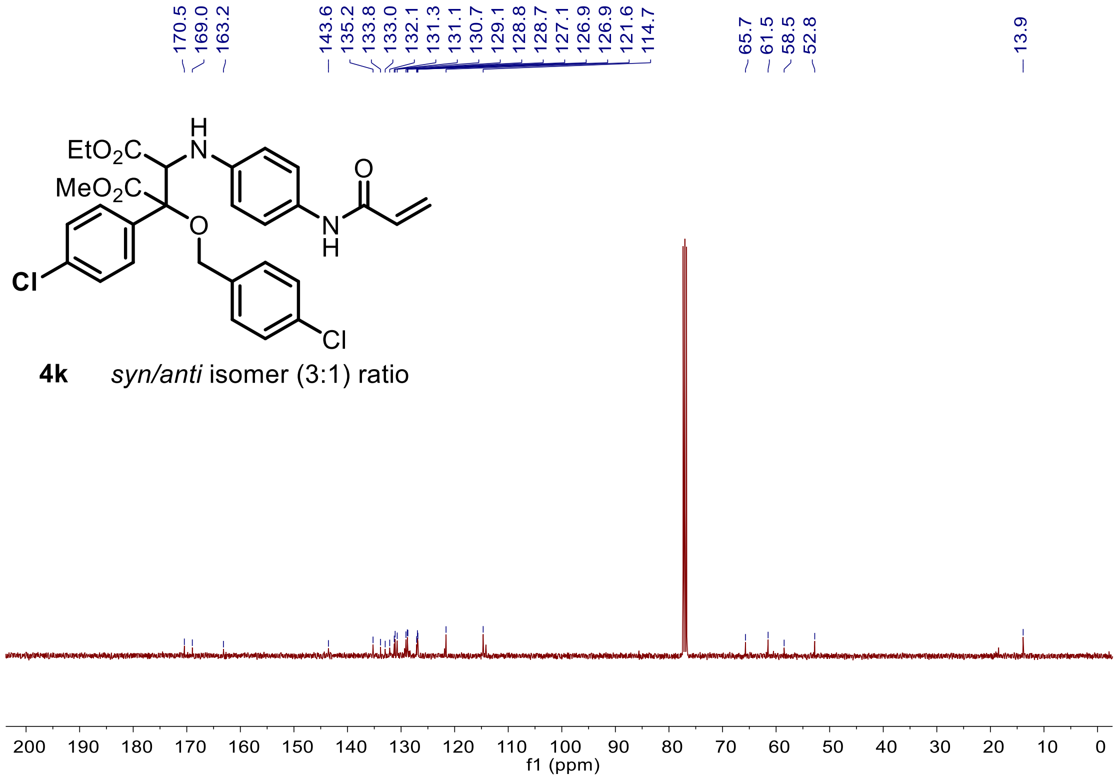

^{13}C NMR Spectrum of Compound **4k** (101MHz, $CDCl_3$)

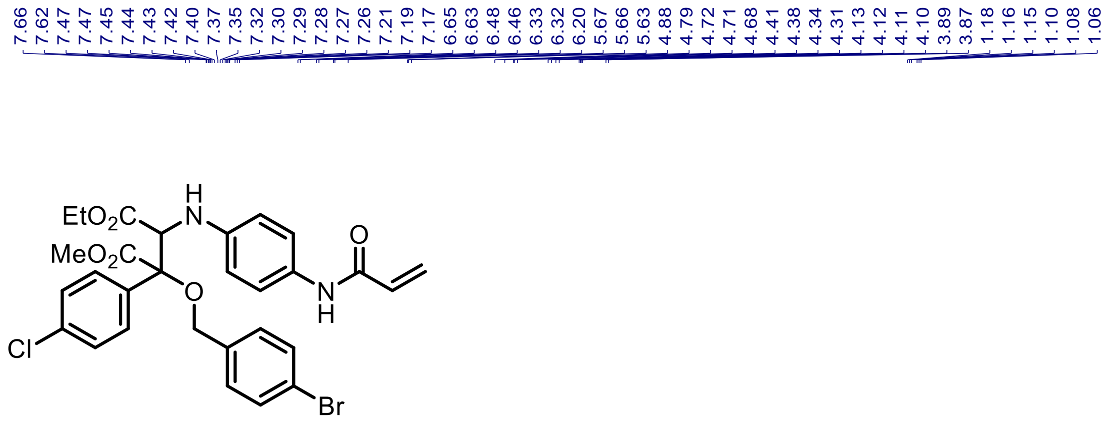

4l *syn/anti* isomer (3:2) ratio

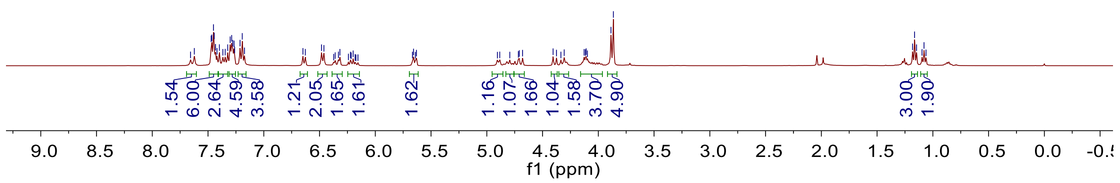

^{1}H NMR Spectrum of Compound **4l** (400MHz, $CDCl_3$)

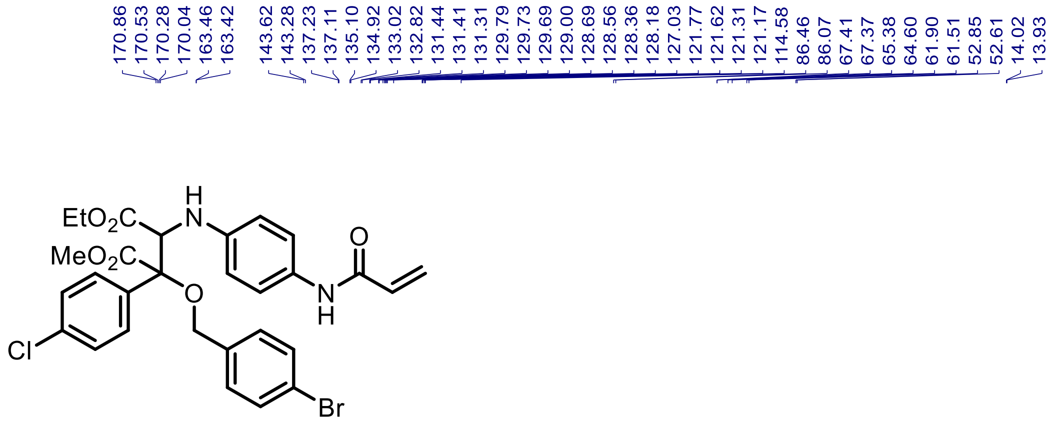

4l *syn/anti* isomer (3:2) ratio

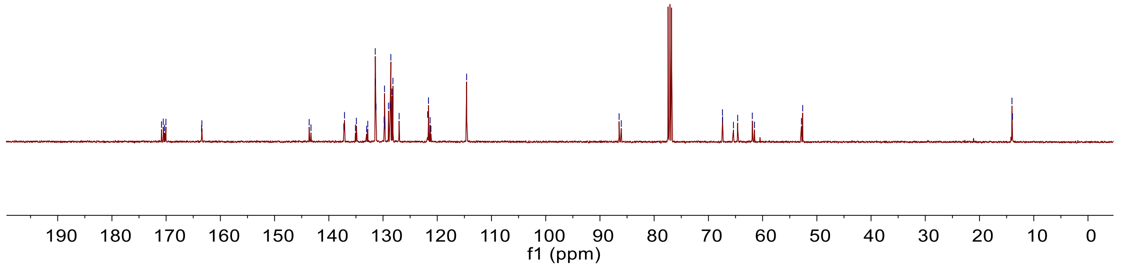

^{13}C NMR Spectrum of Compound **4l** (101MHz, $CDCl_3$)

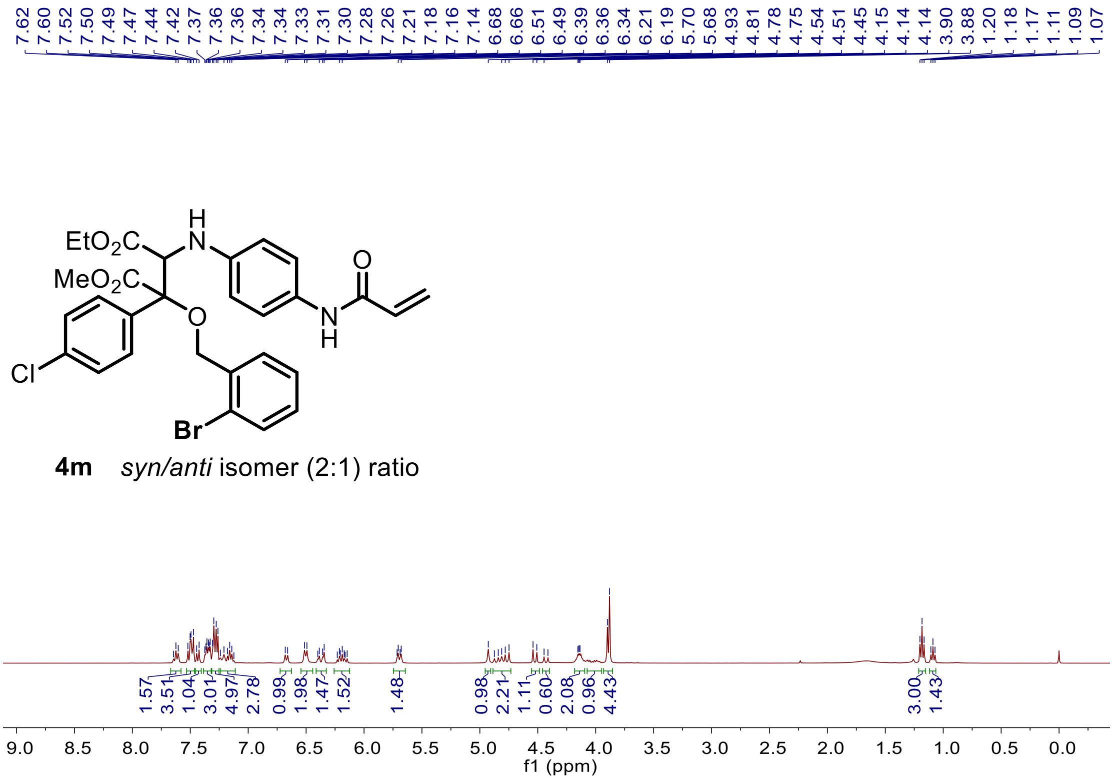

^{1}H NMR Spectrum of Compound **4m** (400MHz, $CDCl_3$)

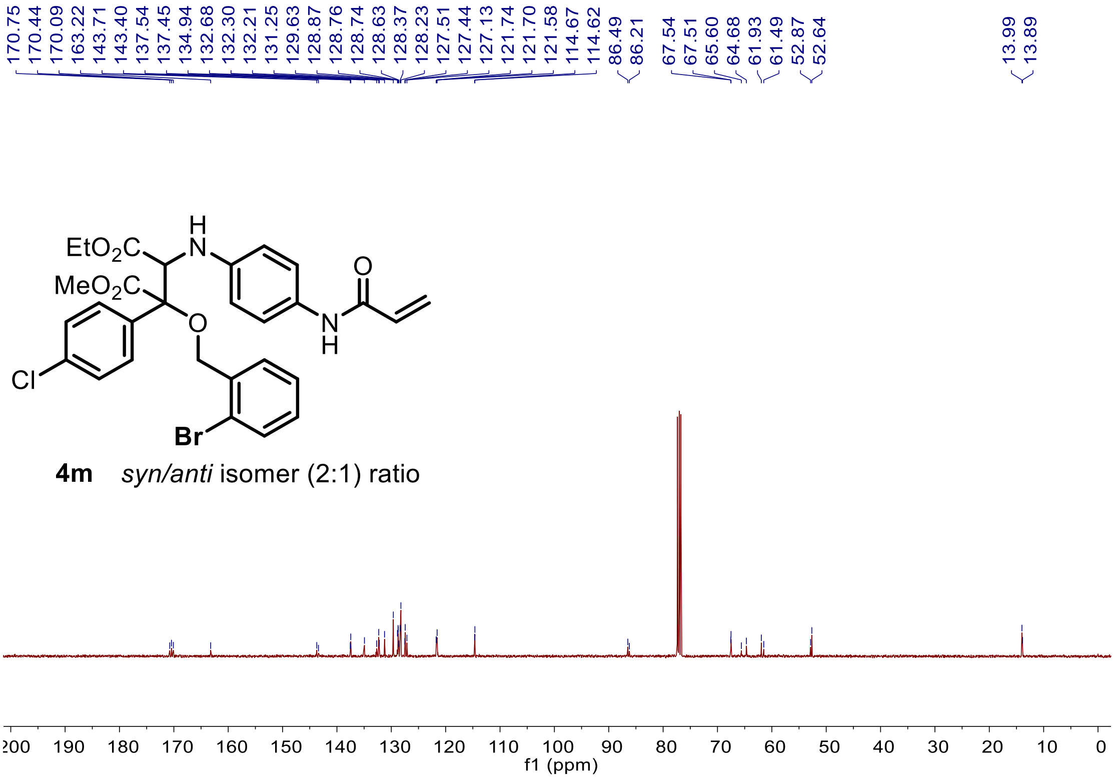

^{13}C NMR Spectrum of Compound **4m** (101MHz, $CDCl_3$)

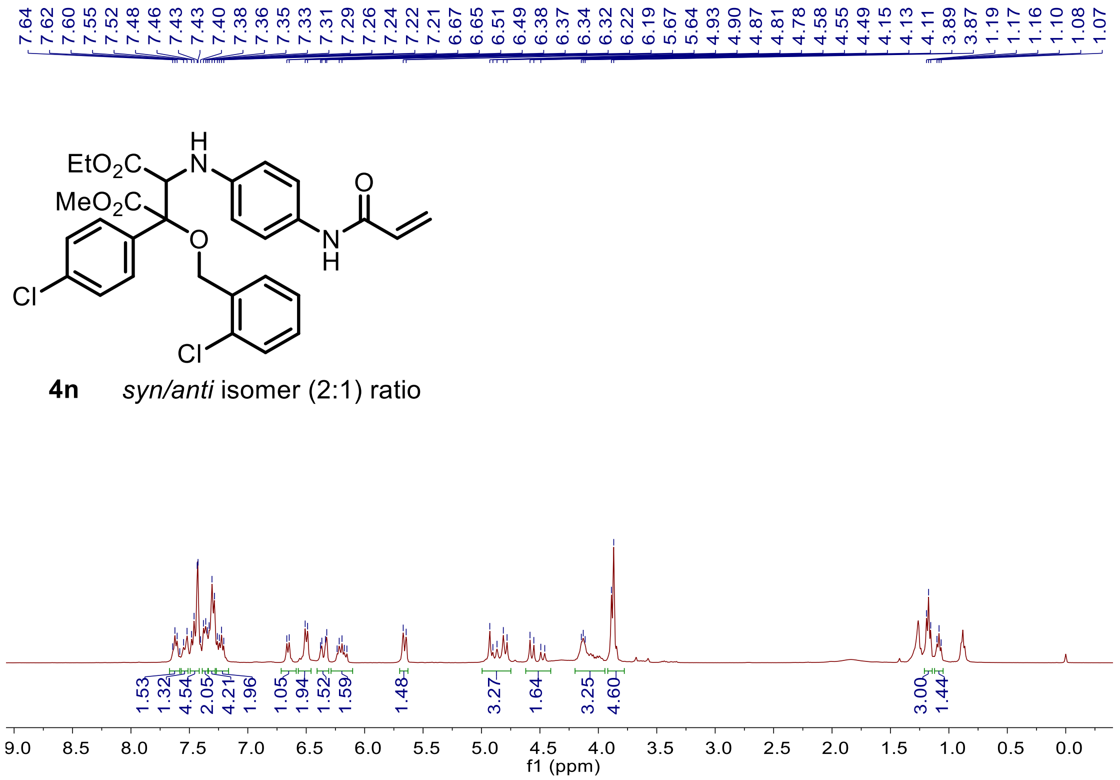

^{1}H NMR Spectrum of Compound **4n** (400MHz, $CDCl_3$)

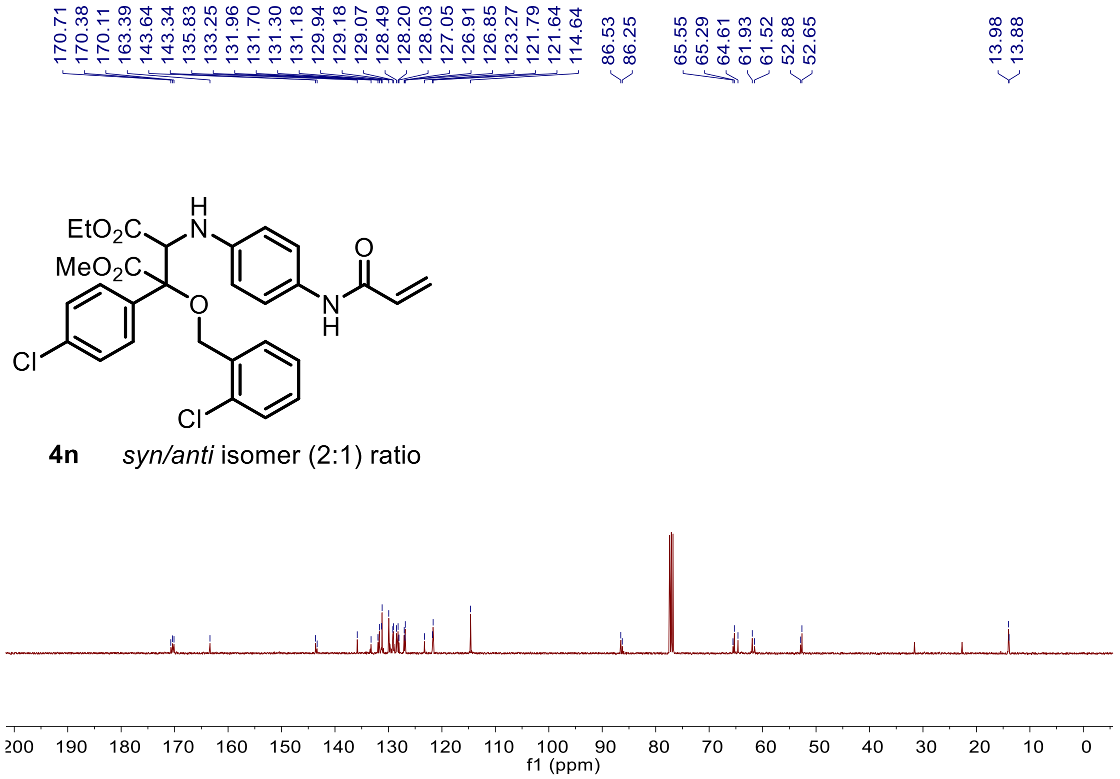

^{13}C NMR Spectrum of Compound **4n** (101MHz, $CDCl_3$)

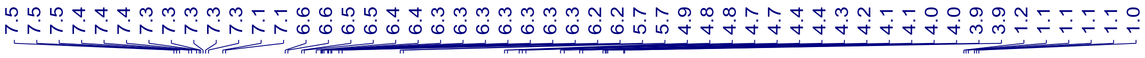

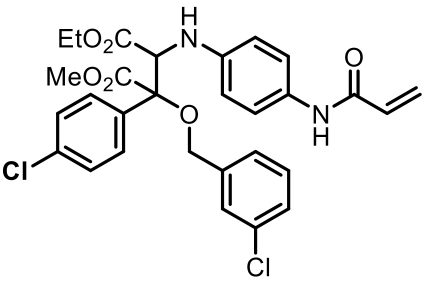

4o *syn/anti* isomer (3:2) ratio

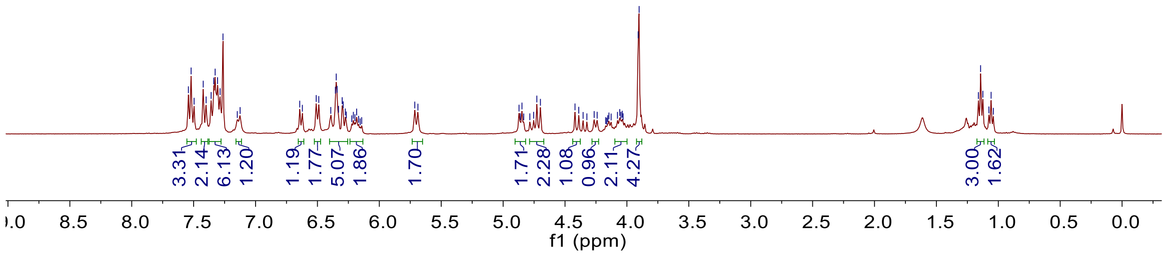

^{1}H NMR Spectrum of Compound **4o** (400MHz, $CDCl_3$)

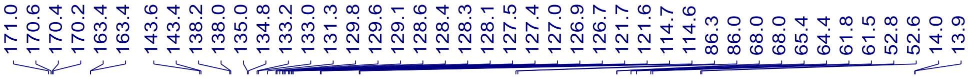

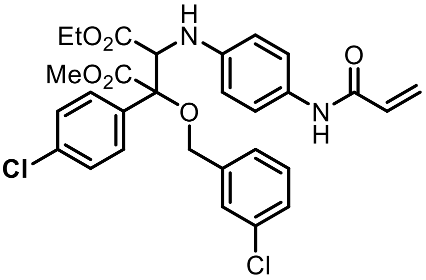

4o *syn/anti* isomer (3:2) ratio

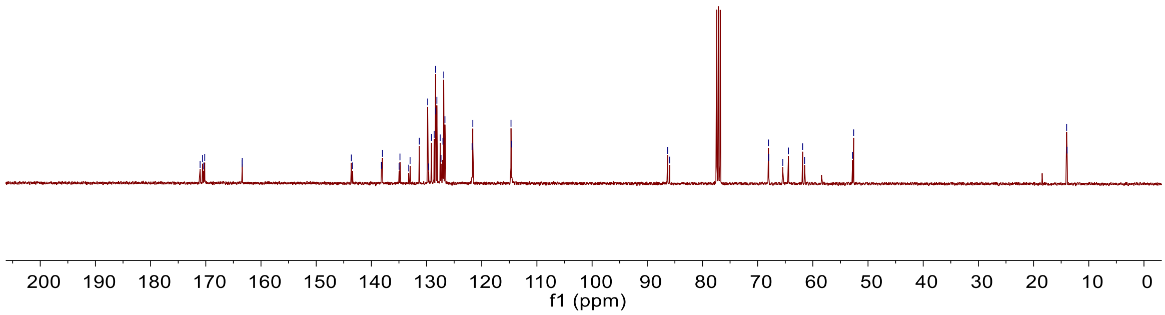

^{13}C NMR Spectrum of Compound **4o** (101MHz, $CDCl_3$)

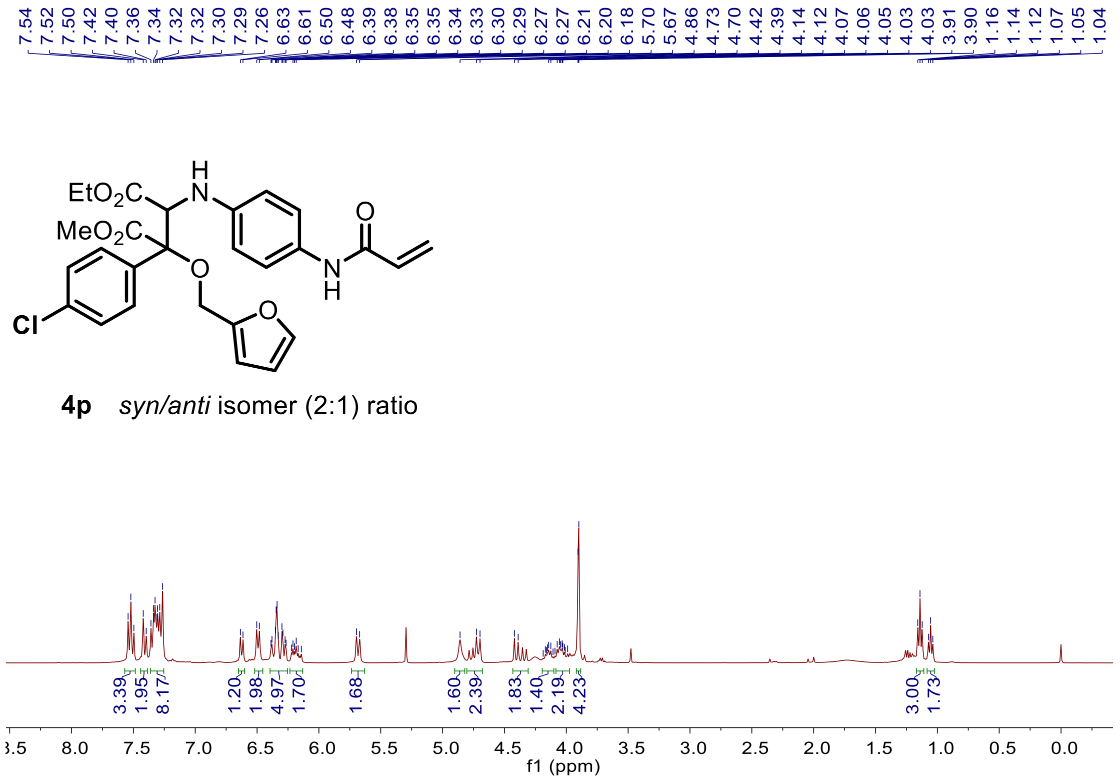

^{1}H NMR Spectrum of Compound **4p** (400MHz, $CDCl_3$)

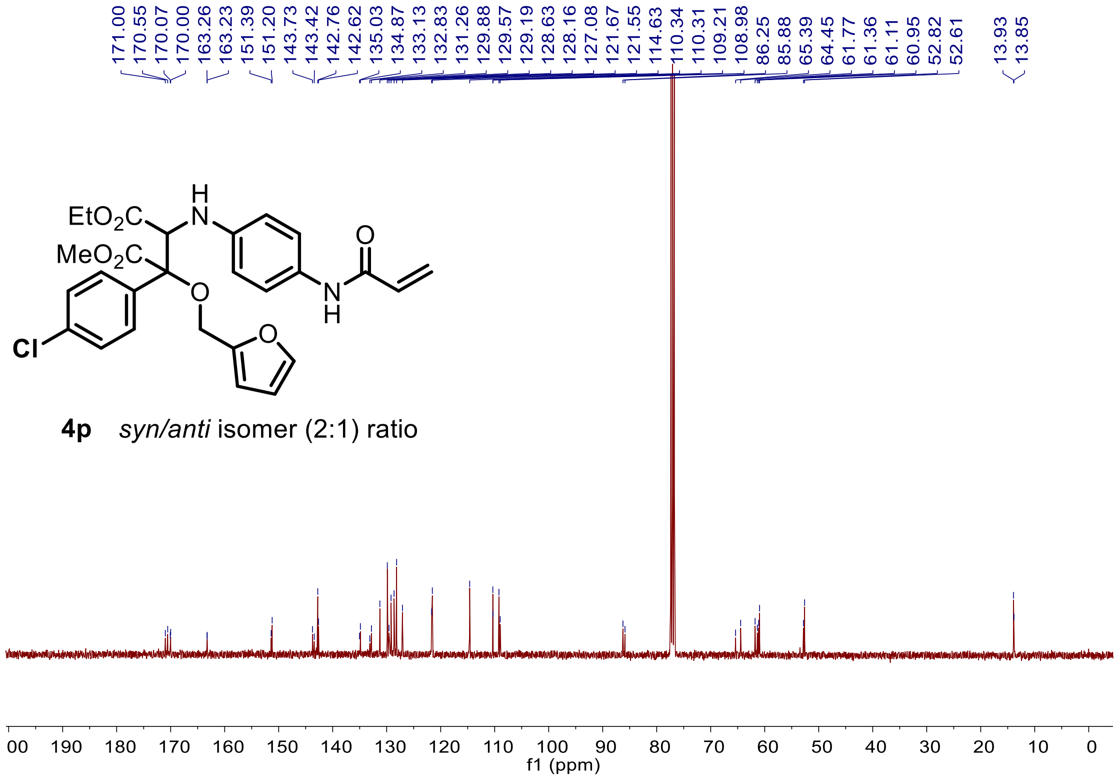

^{13}C NMR Spectrum of Compound **4p** (101MHz, $CDCl_3$)

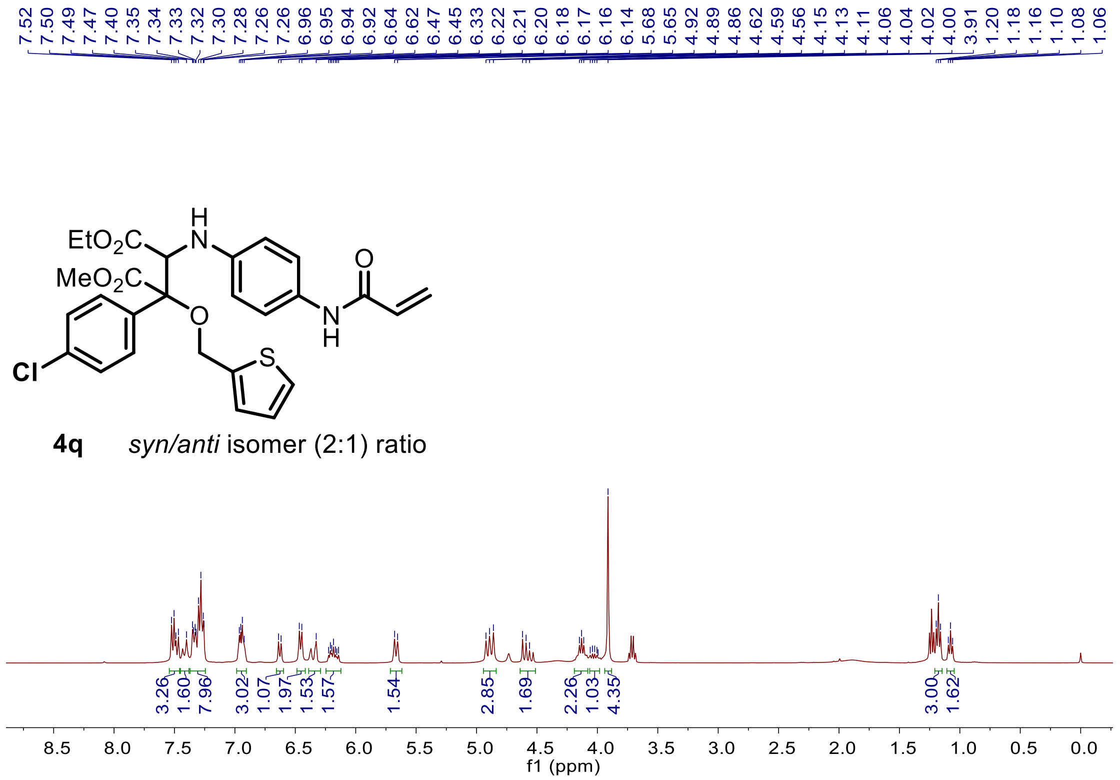

^1H NMR Spectrum of Compound **4q** (400MHz, $CDCl_3$)

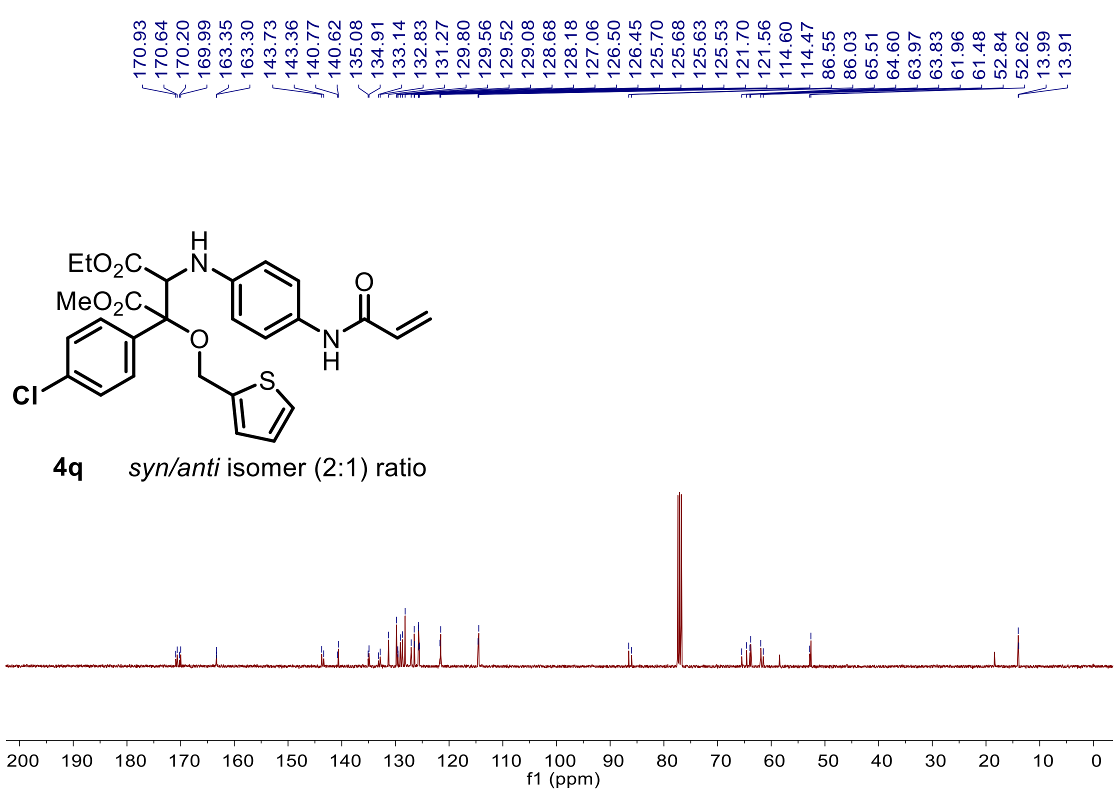

^{13}C NMR Spectrum of Compound **4q** (101MHz, $CDCl_3$)

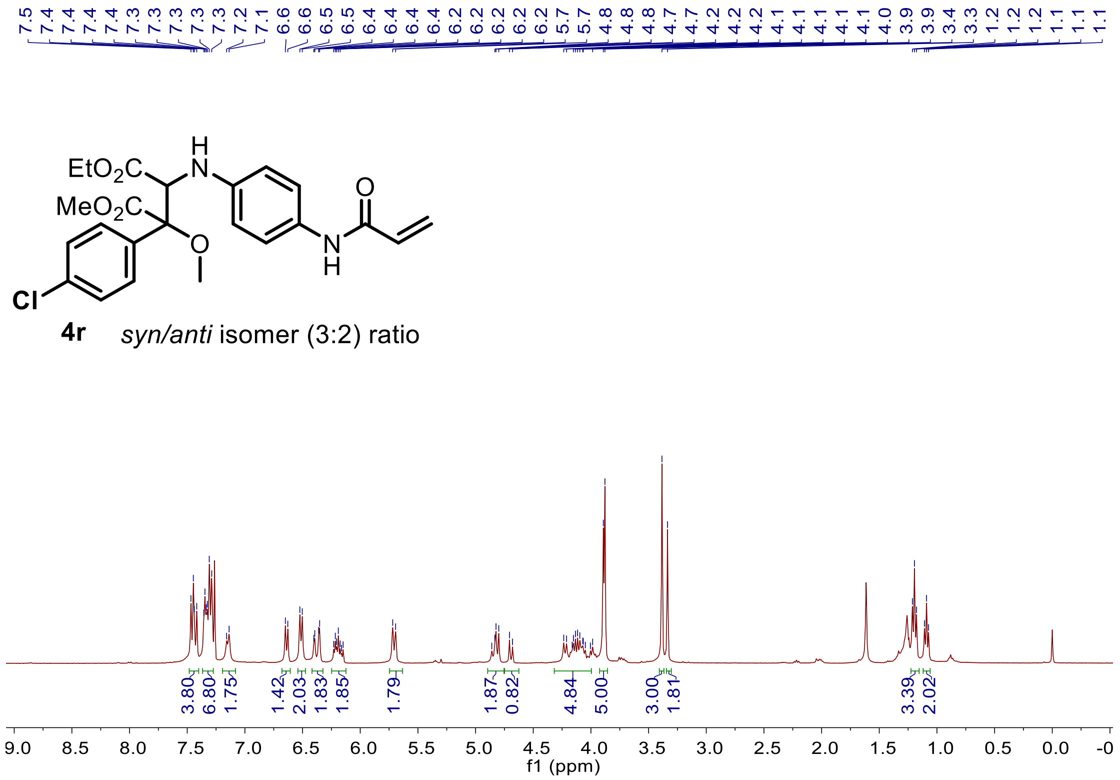

^{1}H NMR Spectrum of Compound **4r** (400MHz, $CDCl_3$)

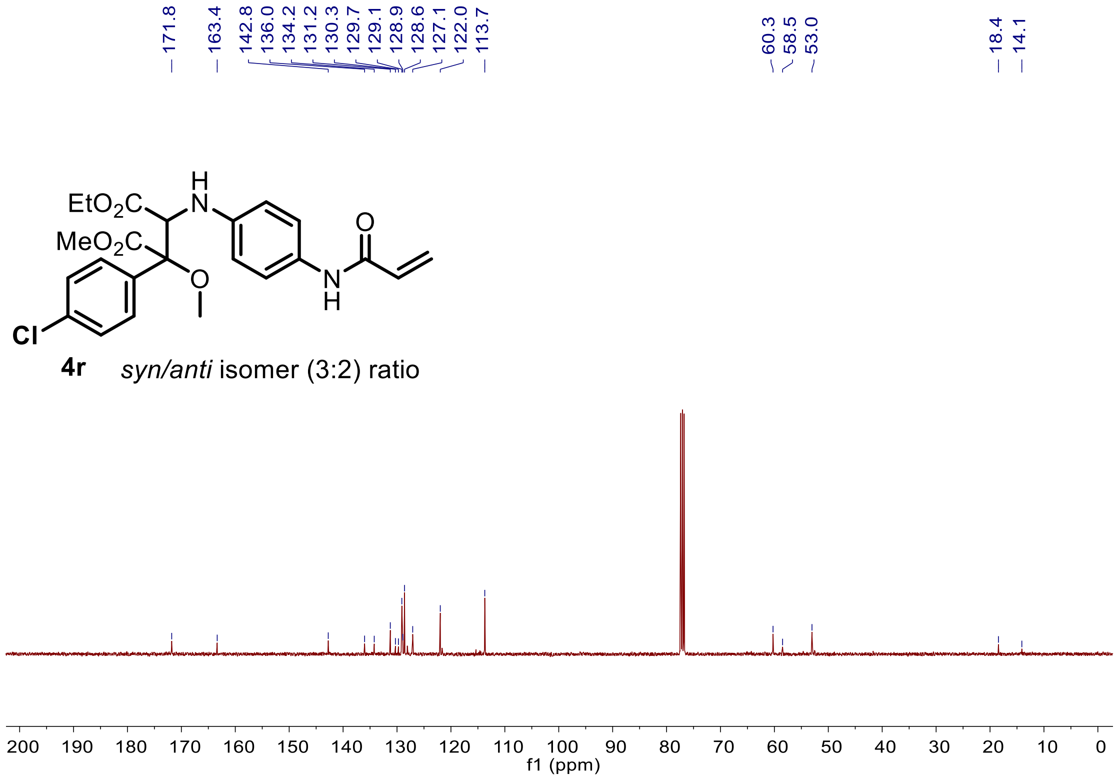

^{13}C NMR Spectrum of Compound **4r** (101MHz, $CDCl_3$)

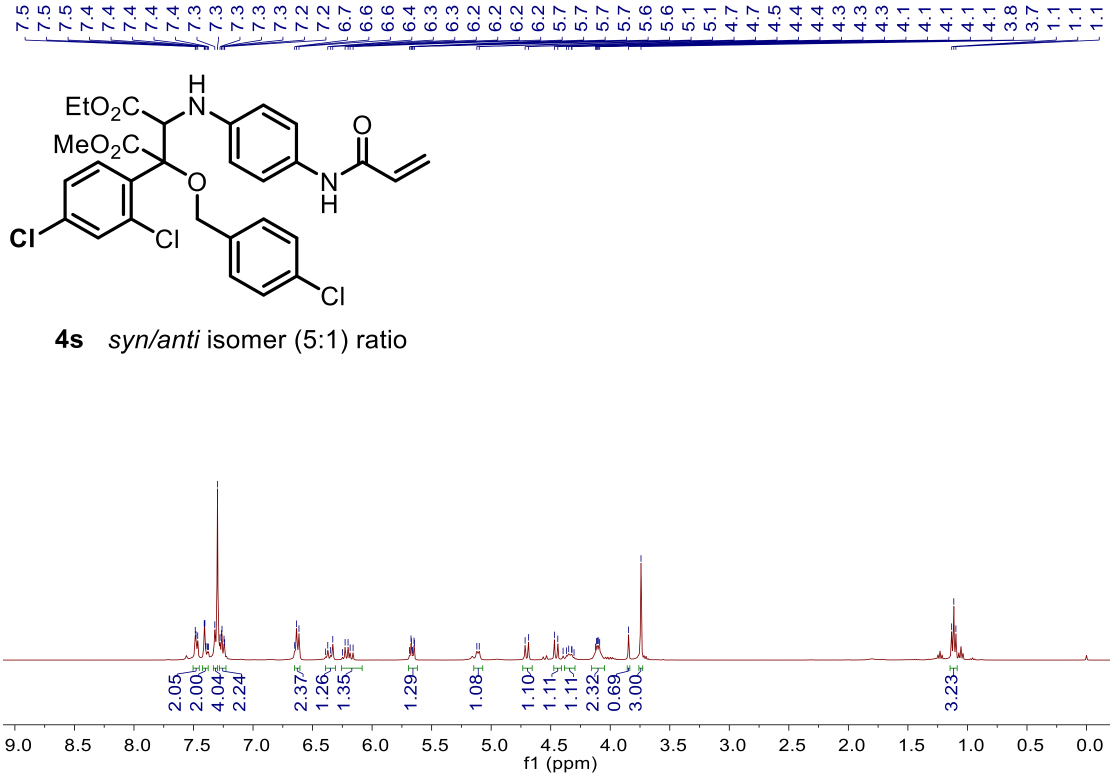

^{1}H NMR Spectrum of Compound **4s** (400MHz, $CDCl_3$)

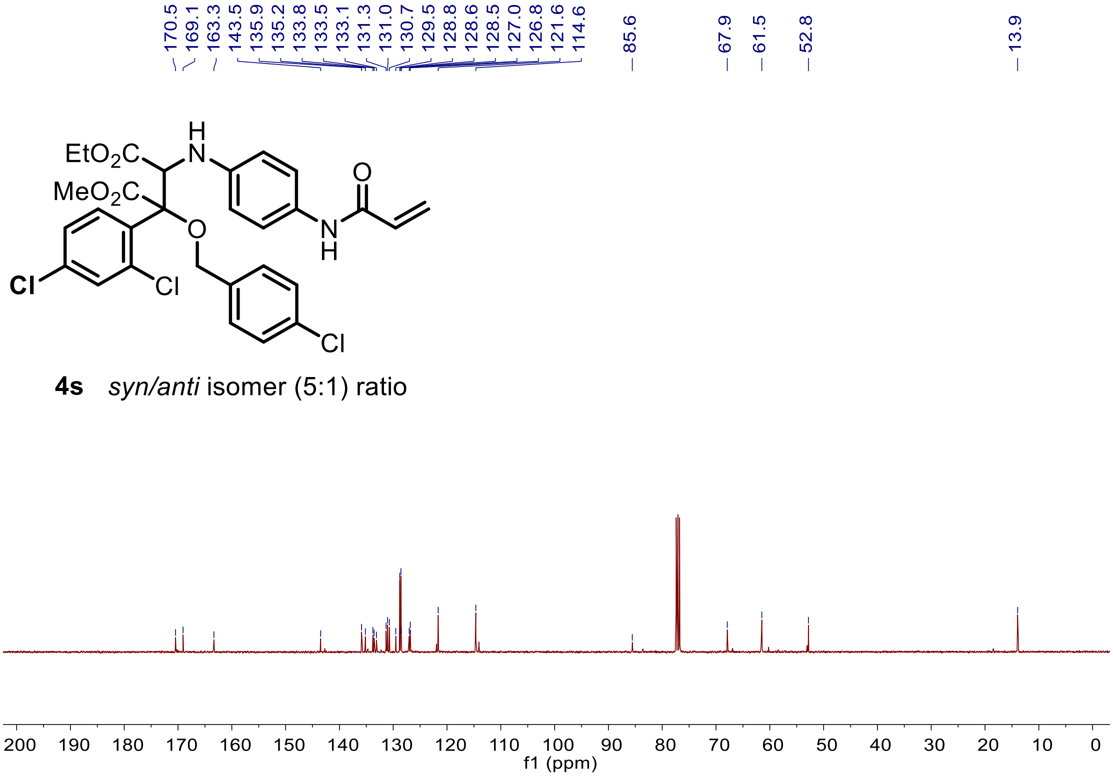

^{13}C NMR Spectrum of Compound **4s** (101MHz, $CDCl_3$)

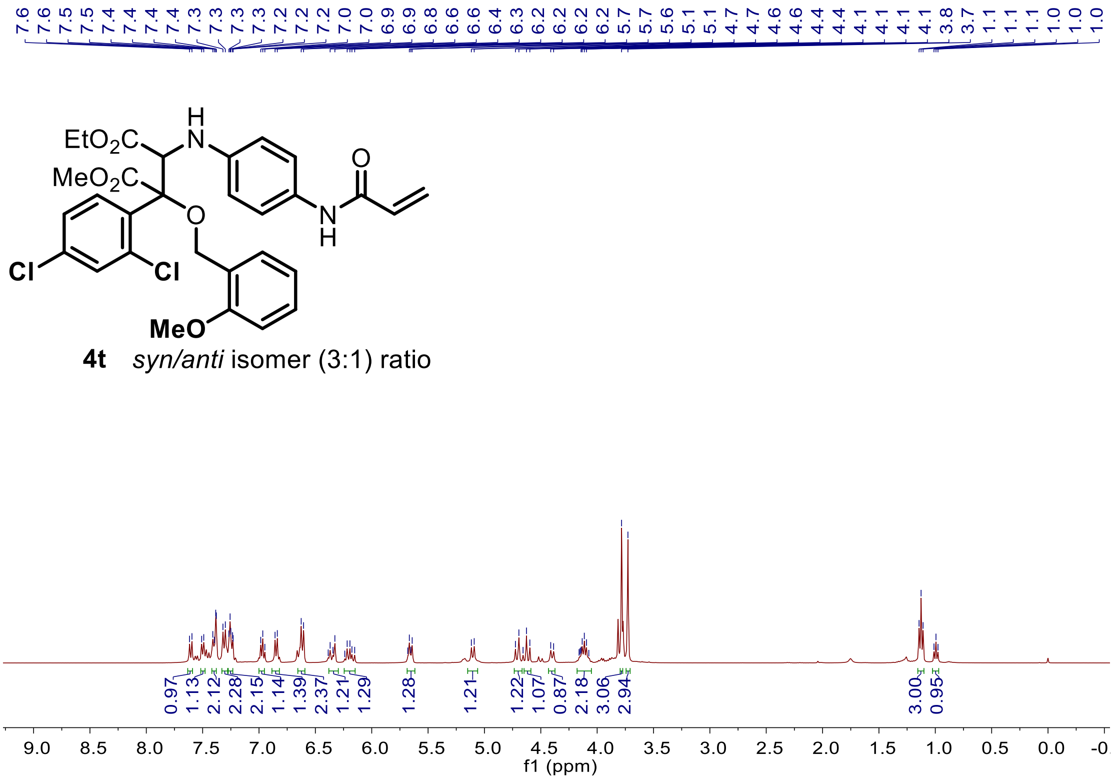

^{1}H NMR Spectrum of Compound **4t** (400MHz, $CDCl_3$)

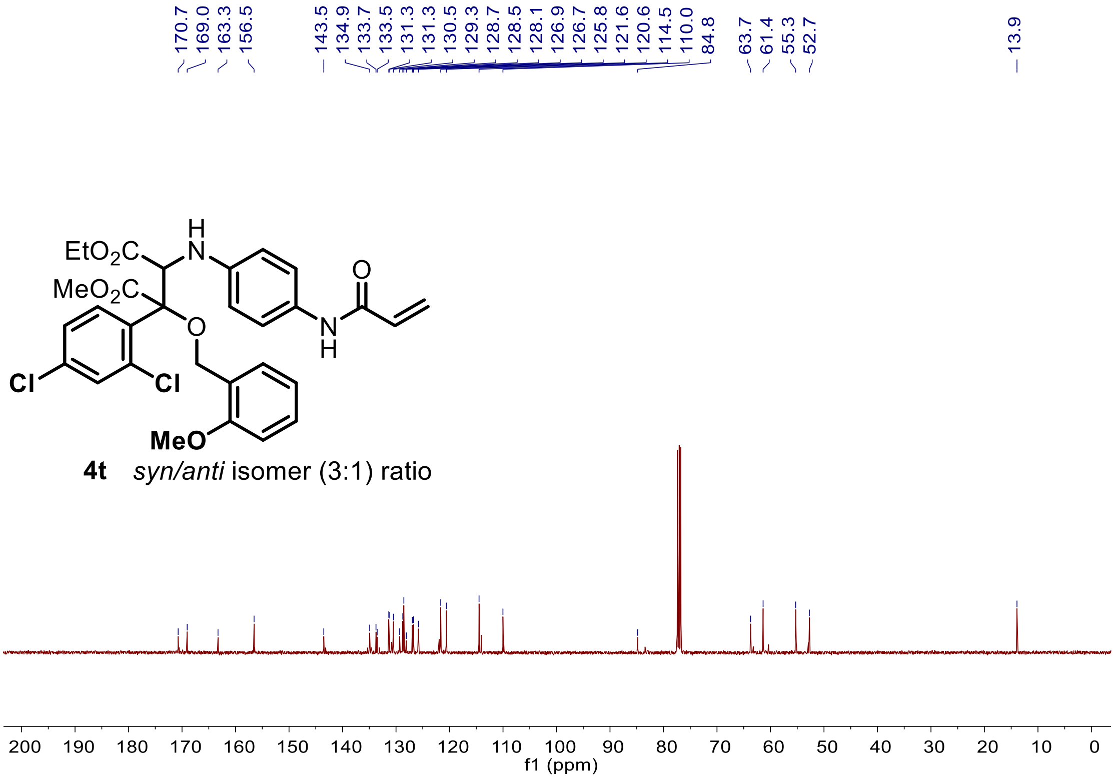

^{13}C NMR Spectrum of Compound **4t** (101MHz, $CDCl_3$)

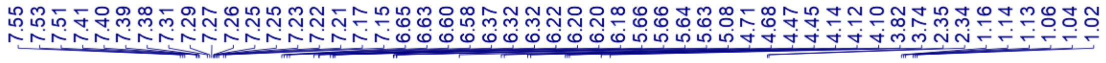

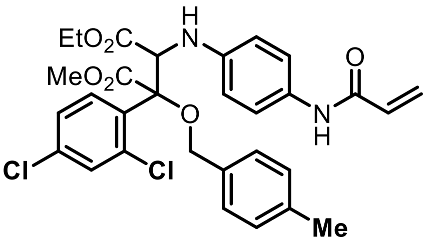

4u *syn/anti* isomer (3:1) ratio

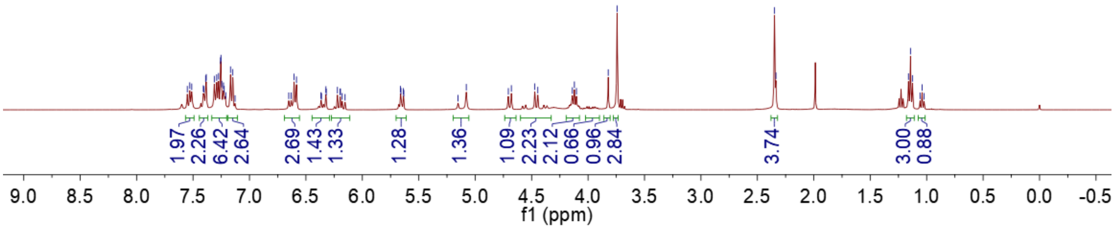

^{1}H NMR Spectrum of Compound **4u** (400MHz, $CDCl_3$)

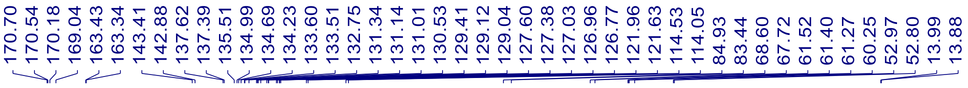

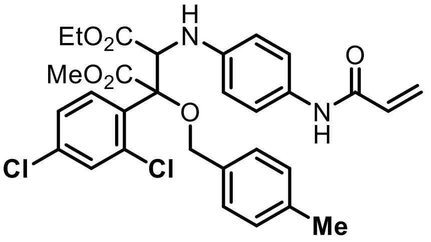

4u *syn/anti* isomer (3:1) ratio

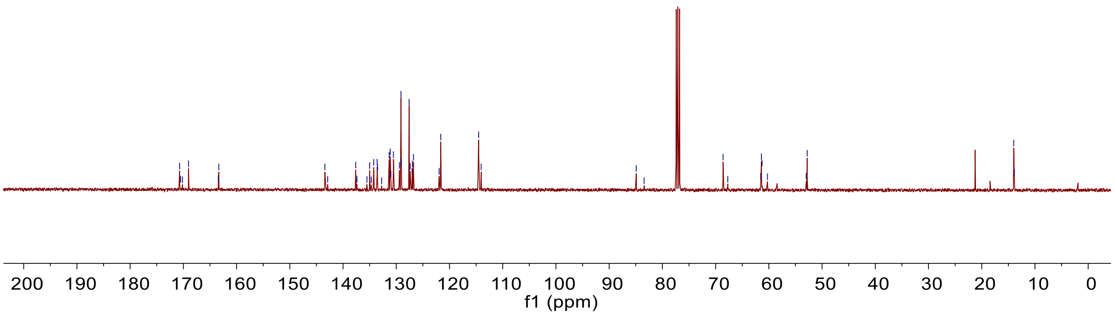

^{13}C NMR Spectrum of Compound **4u** (101MHz, $CDCl_3$)

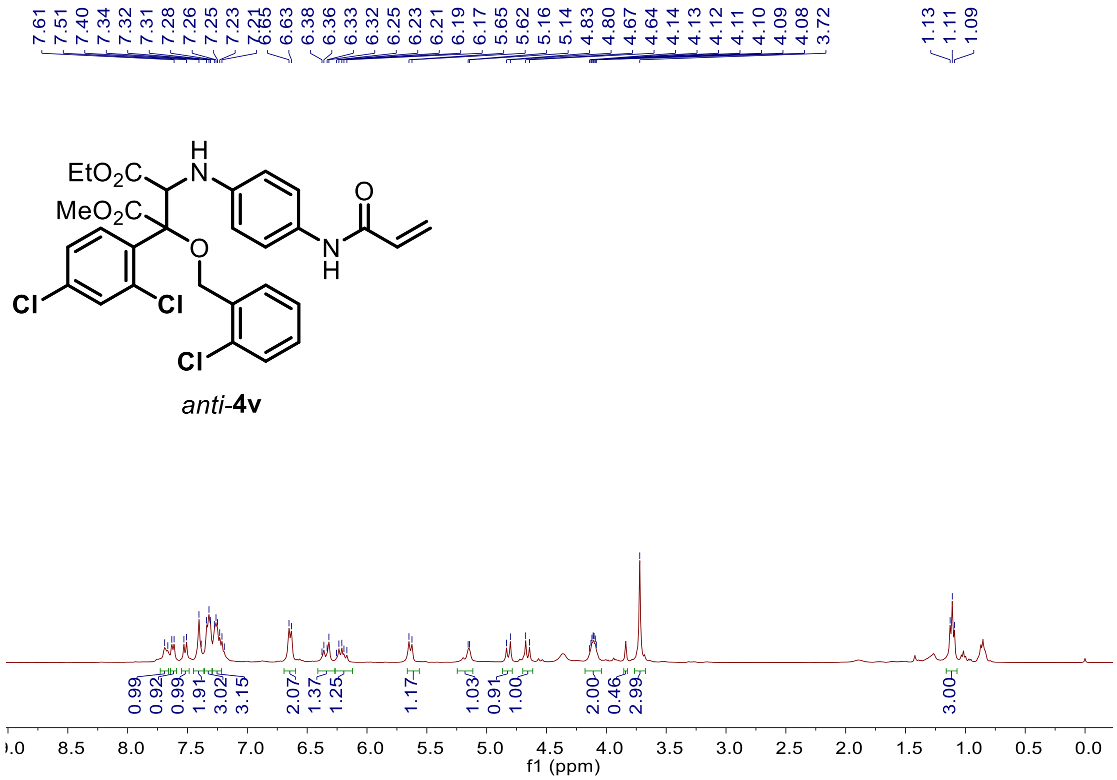

^{1}H NMR Spectrum of Compound **4v** (400MHz, $CDCl_3$)

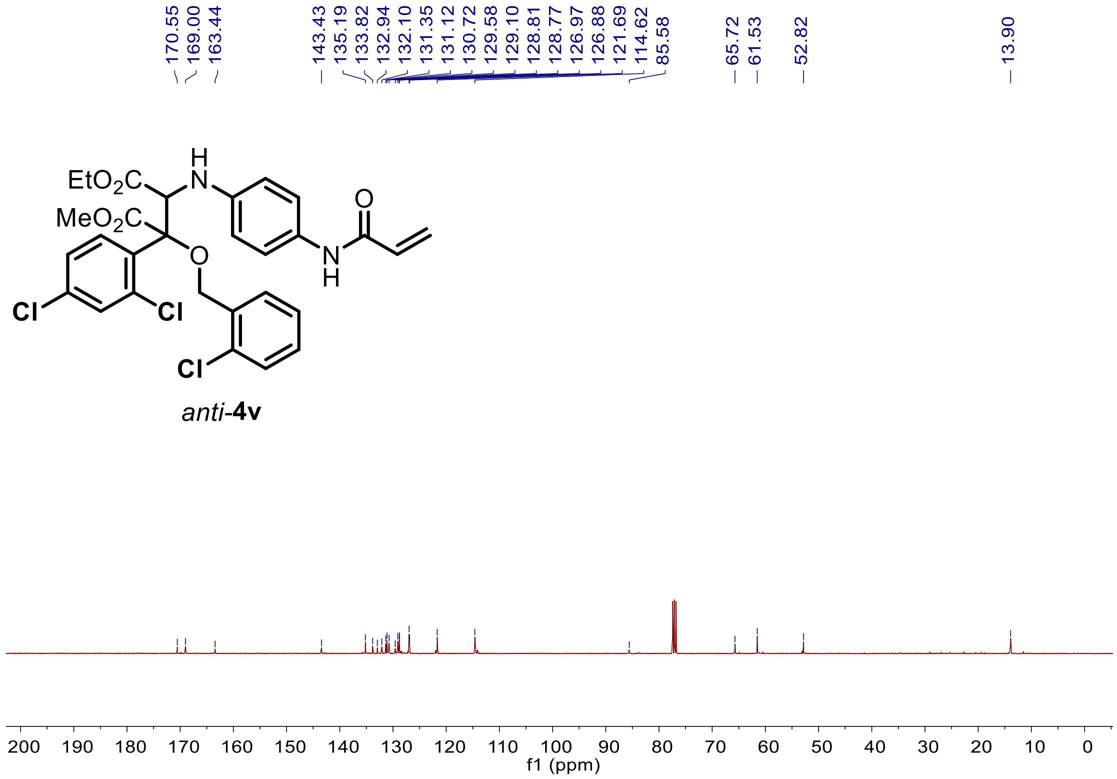

^{13}C NMR Spectrum of Compound **4v** (101MHz, $CDCl_3$)

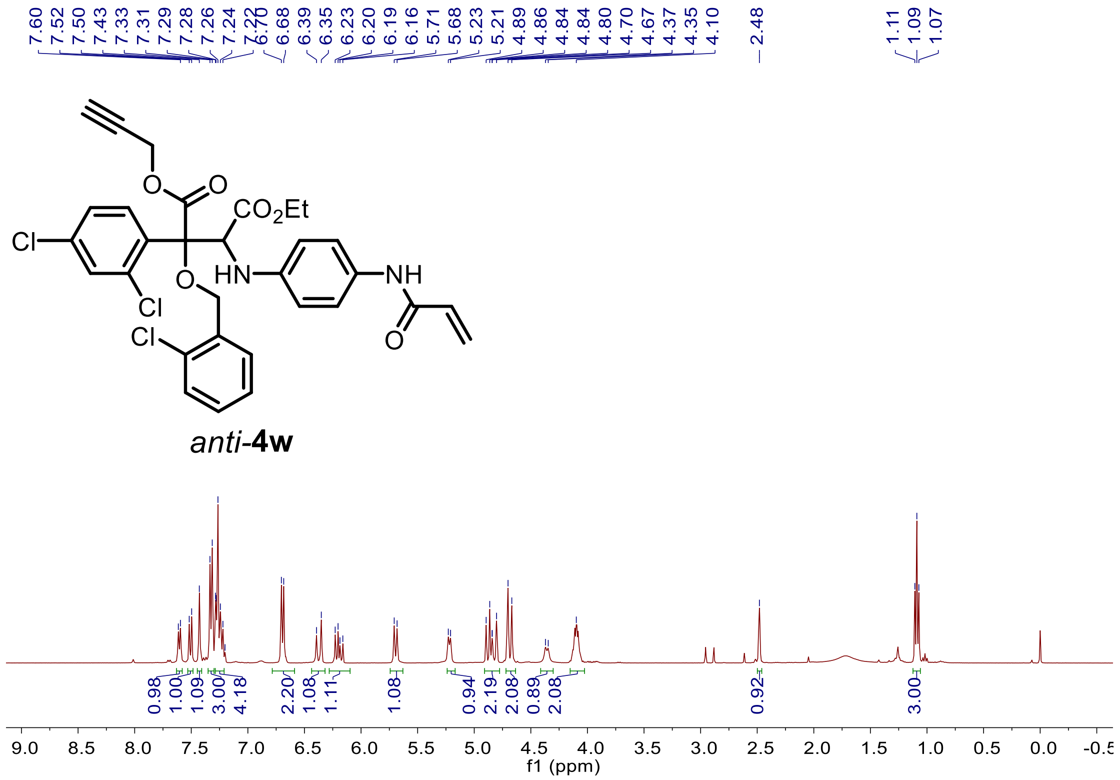

^{1}H NMR Spectrum of Compound **4w** (400MHz, $CDCl_3$)

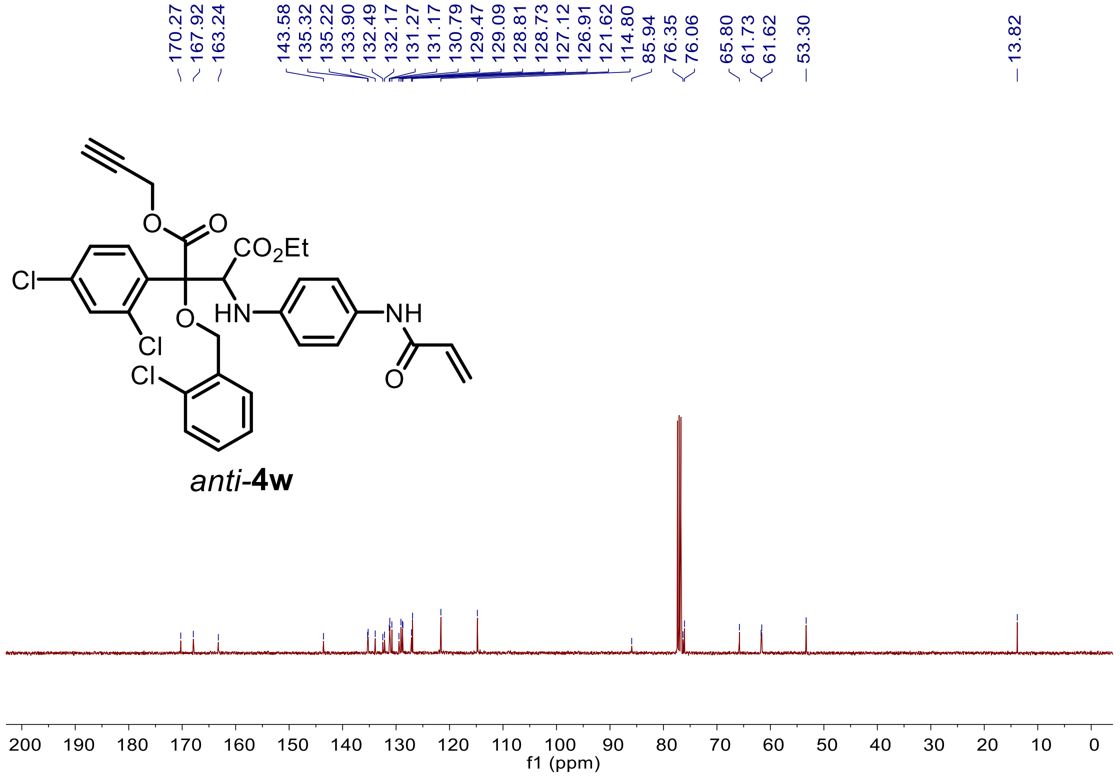

^{13}C NMR Spectrum of Compound **4w** (101MHz, $CDCl_3$)

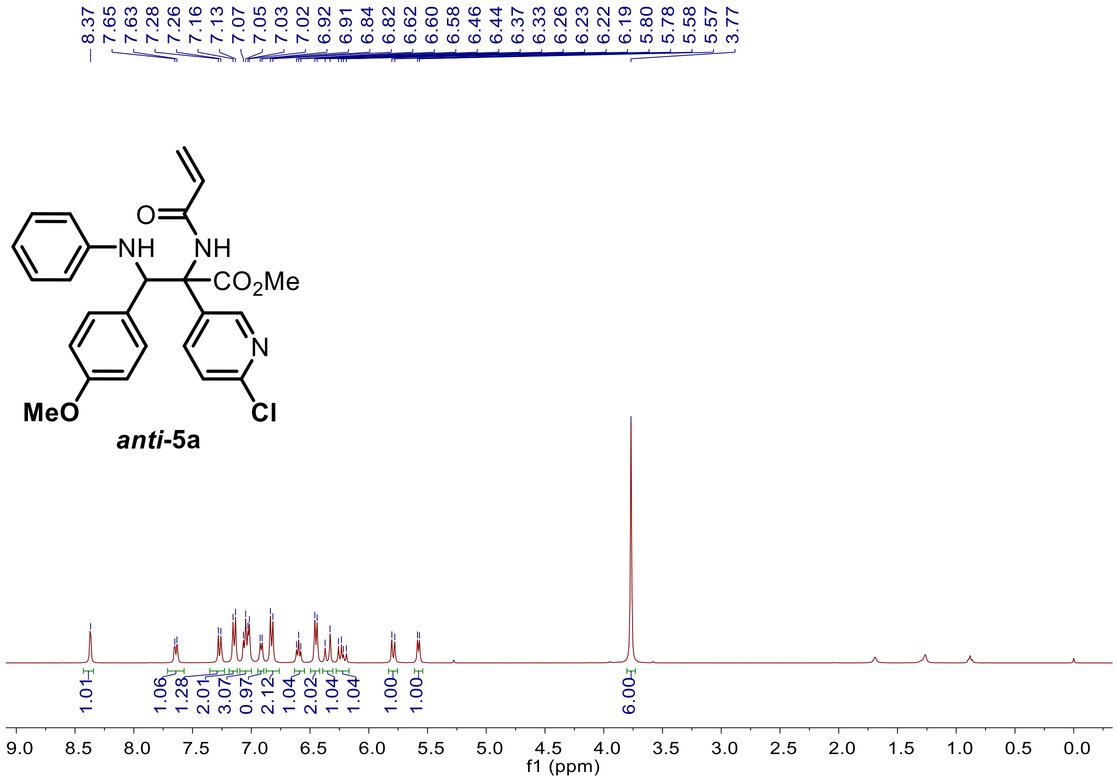

^{1}H NMR Spectrum of Compound **5a** (400MHz, $CDCl_3$)

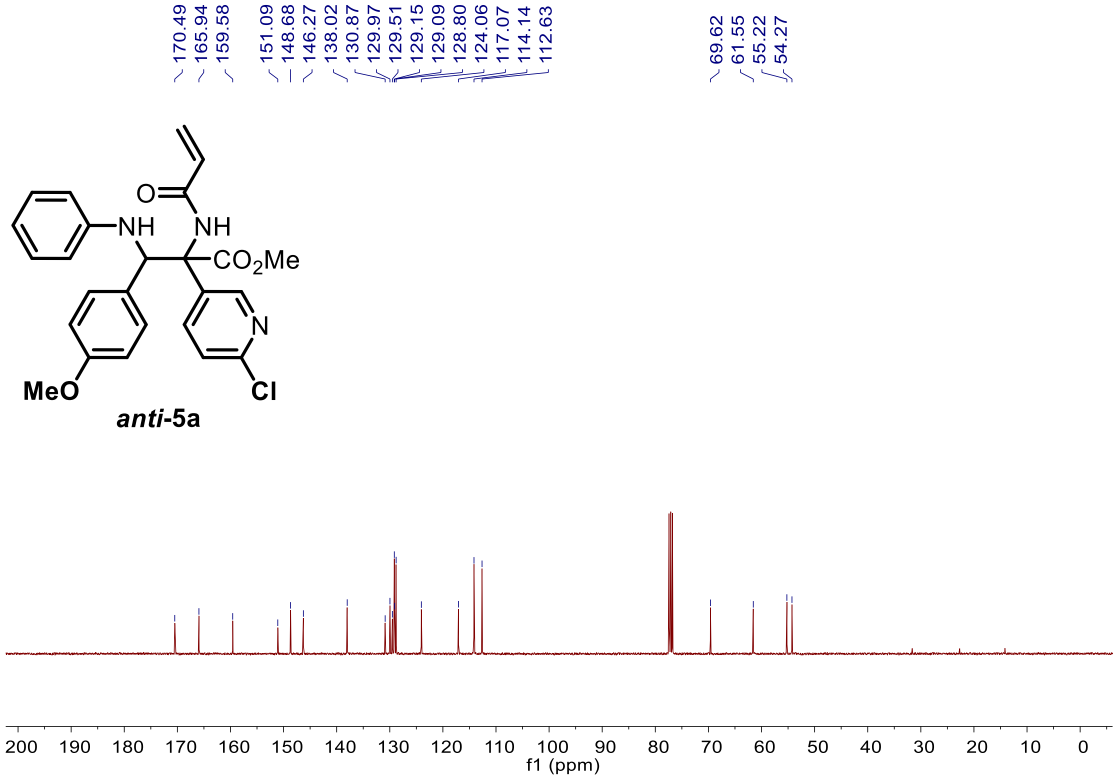

^{13}C NMR Spectrum of Compound **5a** (101MHz, $CDCl_3$)

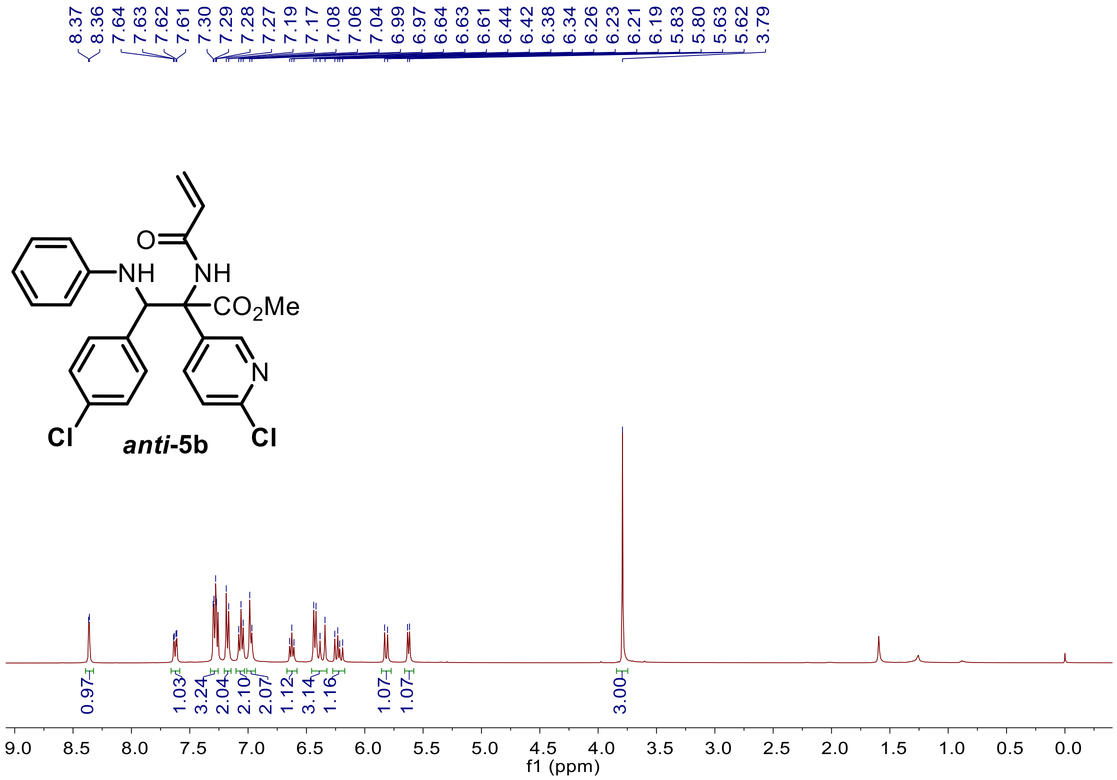

^{1}H NMR Spectrum of Compound **5b** (400MHz, $CDCl_3$)

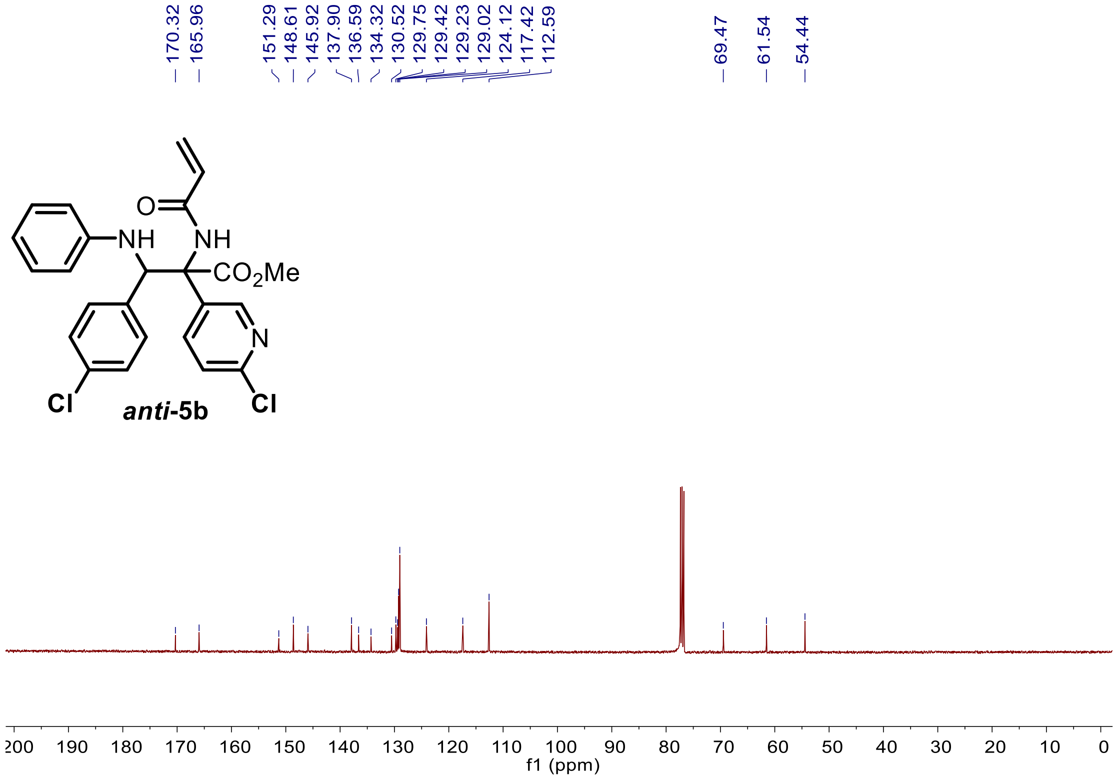

^{13}C NMR Spectrum of Compound **5b** (101MHz, $CDCl_3$)

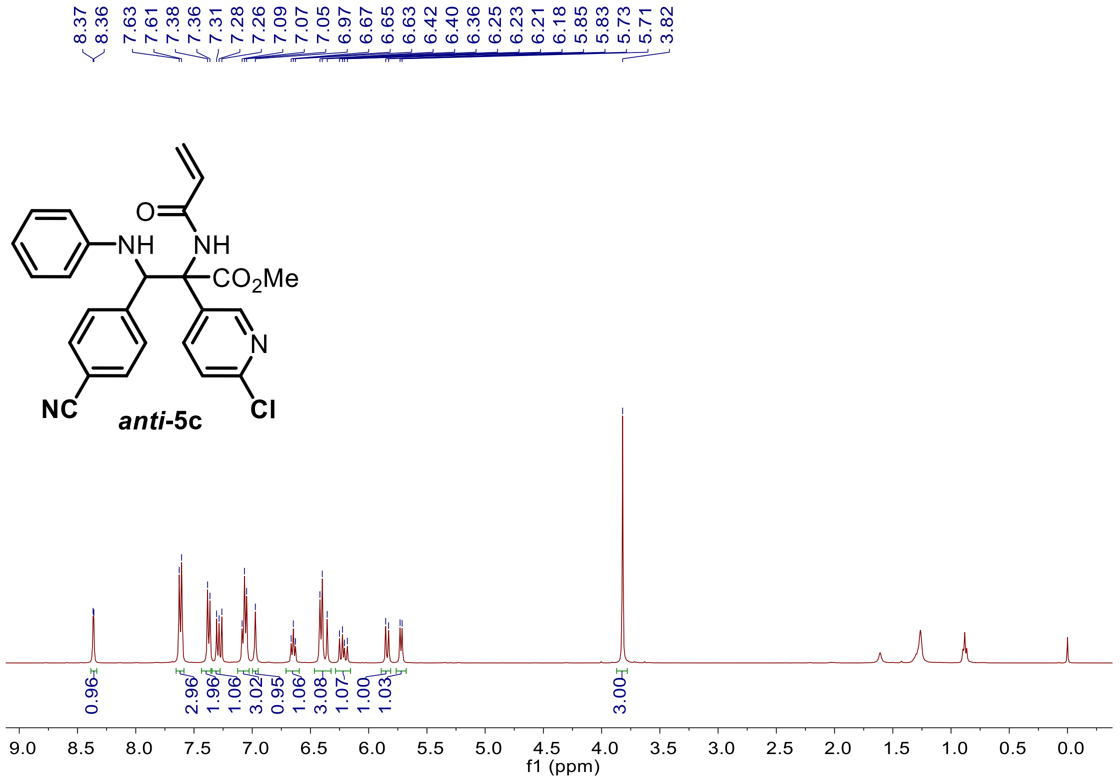

^{1}H NMR Spectrum of Compound **5c** (400MHz, $CDCl_3$)

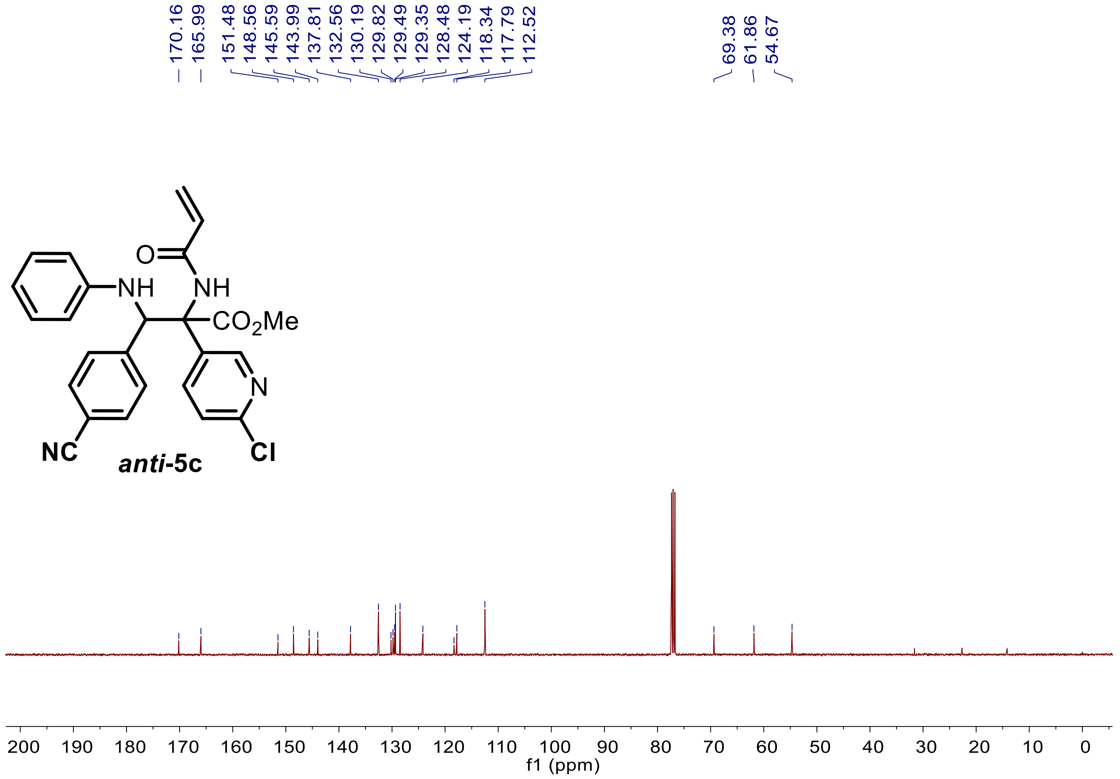

^{13}C NMR Spectrum of Compound **5c** (101MHz, $CDCl_3$)

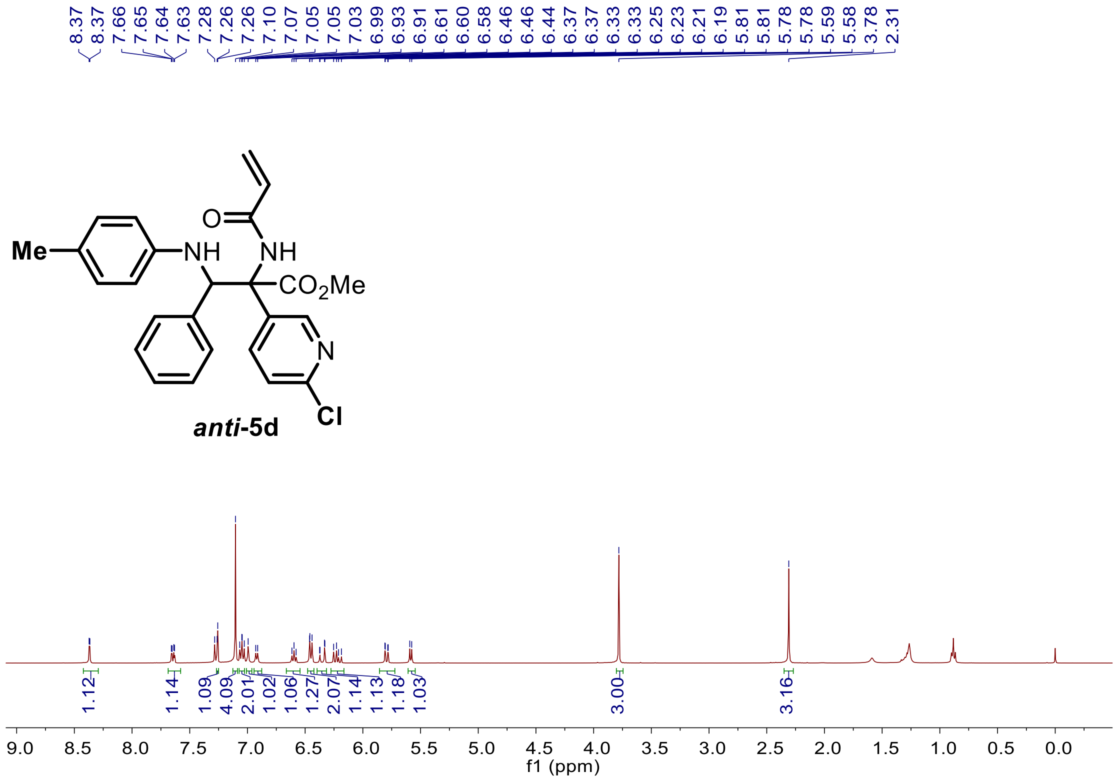

^{1}H NMR Spectrum of Compound **5d** (400MHz, $CDCl_3$)

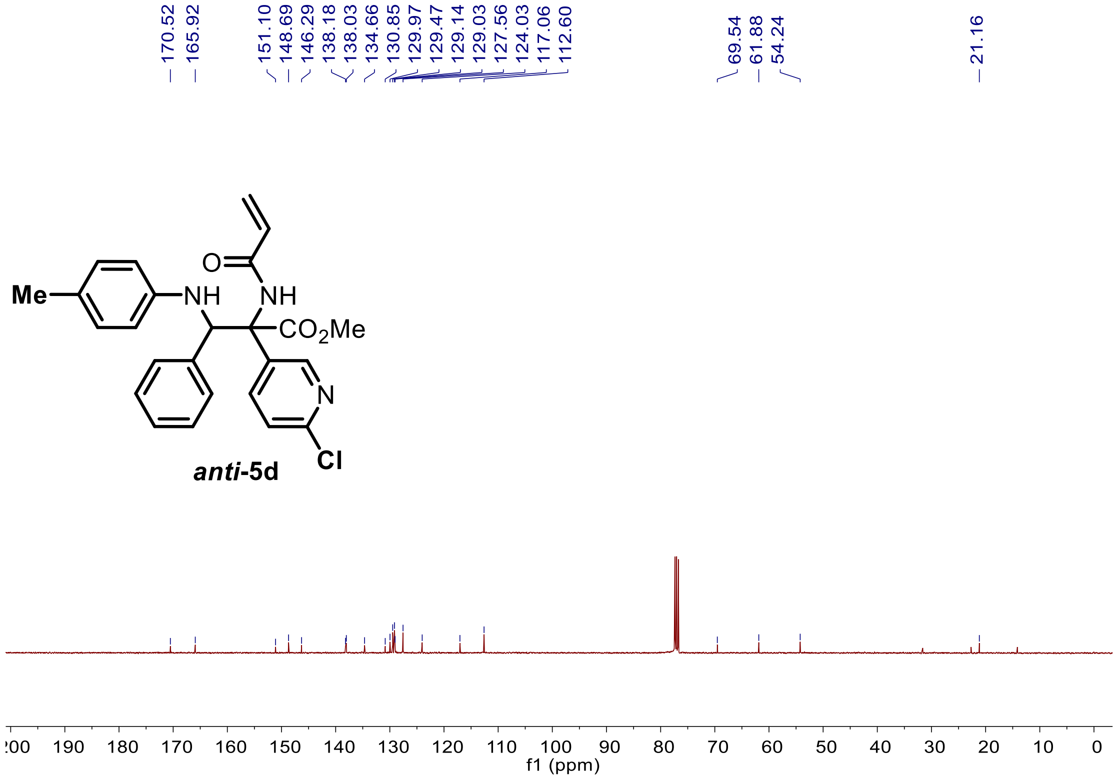

^{13}C NMR Spectrum of Compound **5d** (101MHz, $CDCl_3$)

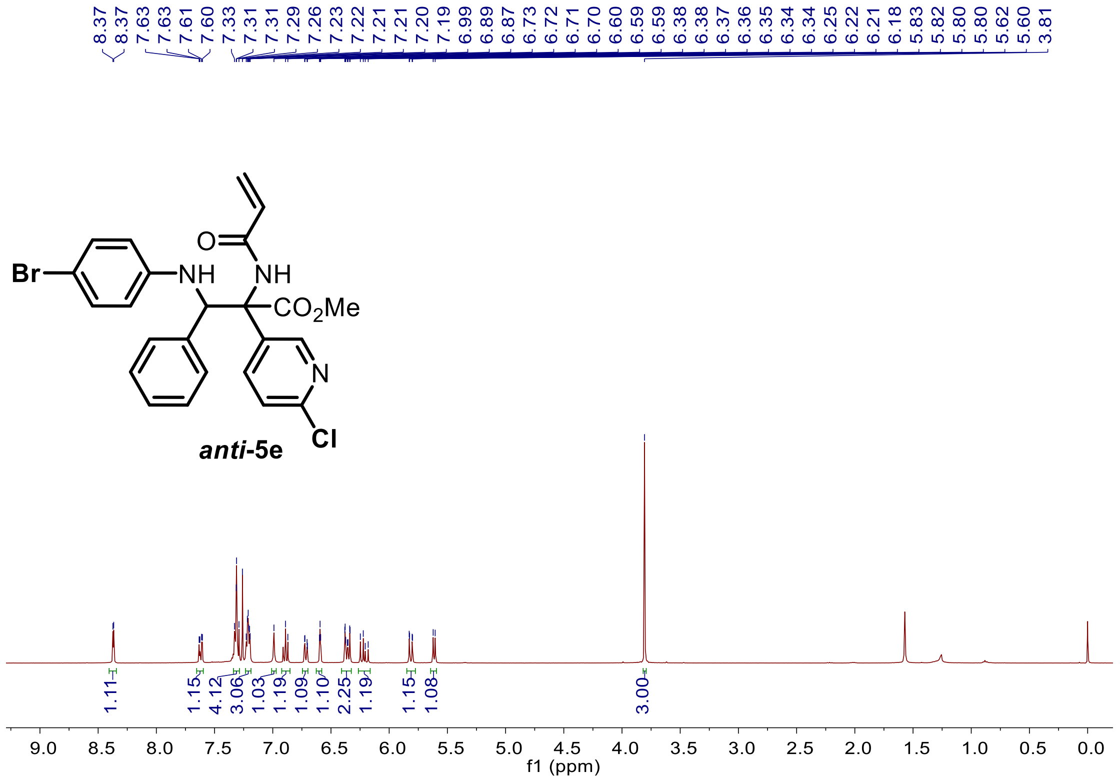

^{1}H NMR Spectrum of Compound **5e** (400MHz, $CDCl_3$)

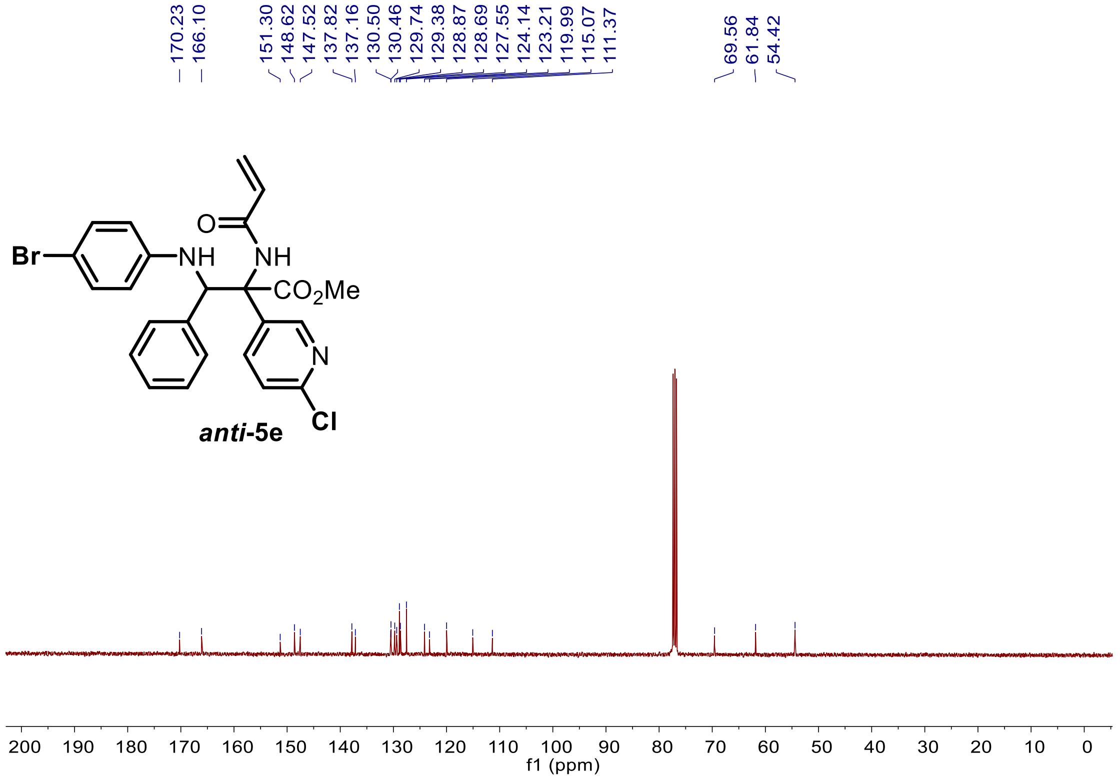

^{13}C NMR Spectrum of Compound **5e** (101MHz, $CDCl_3$)

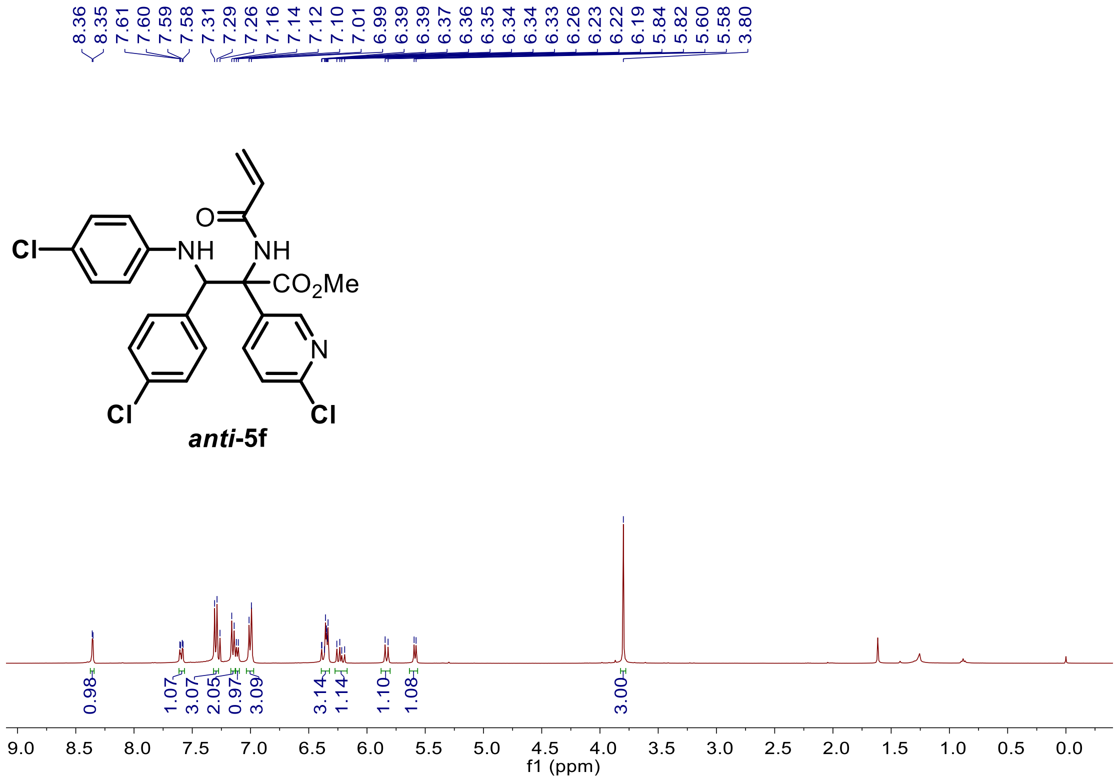

^{1}H NMR Spectrum of Compound **5f** (400MHz, $CDCl_3$)

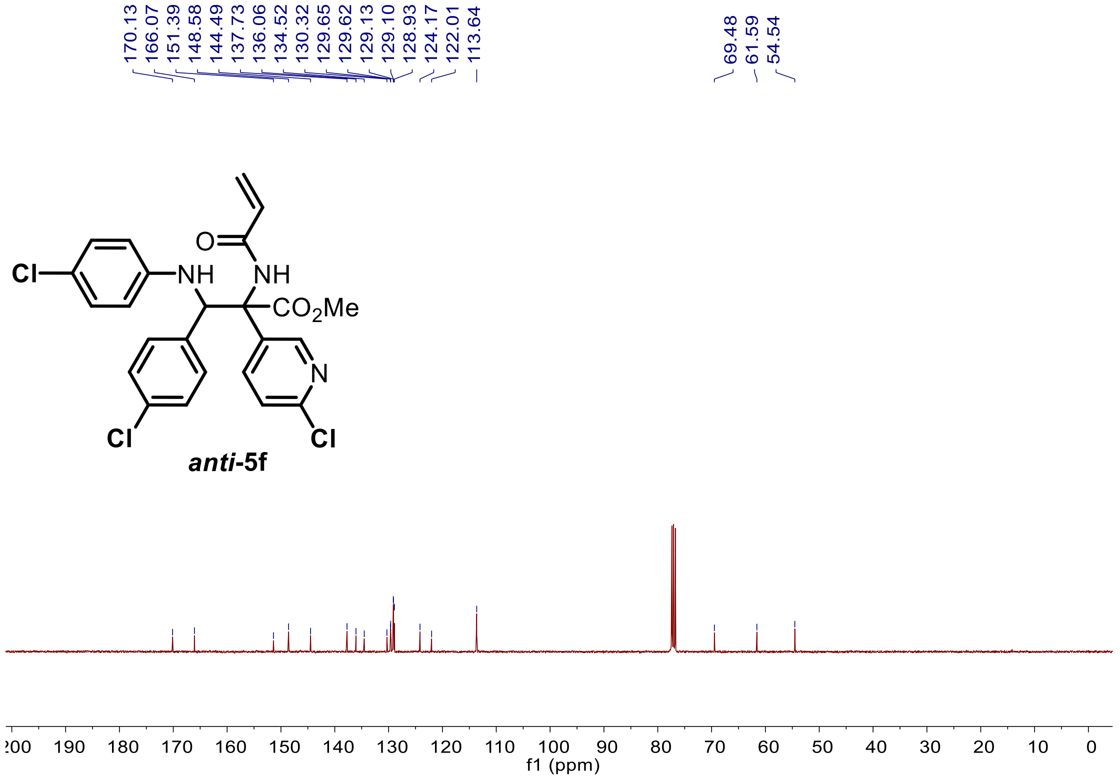

^{13}C NMR Spectrum of Compound **5f** (101MHz, $CDCl_3$)

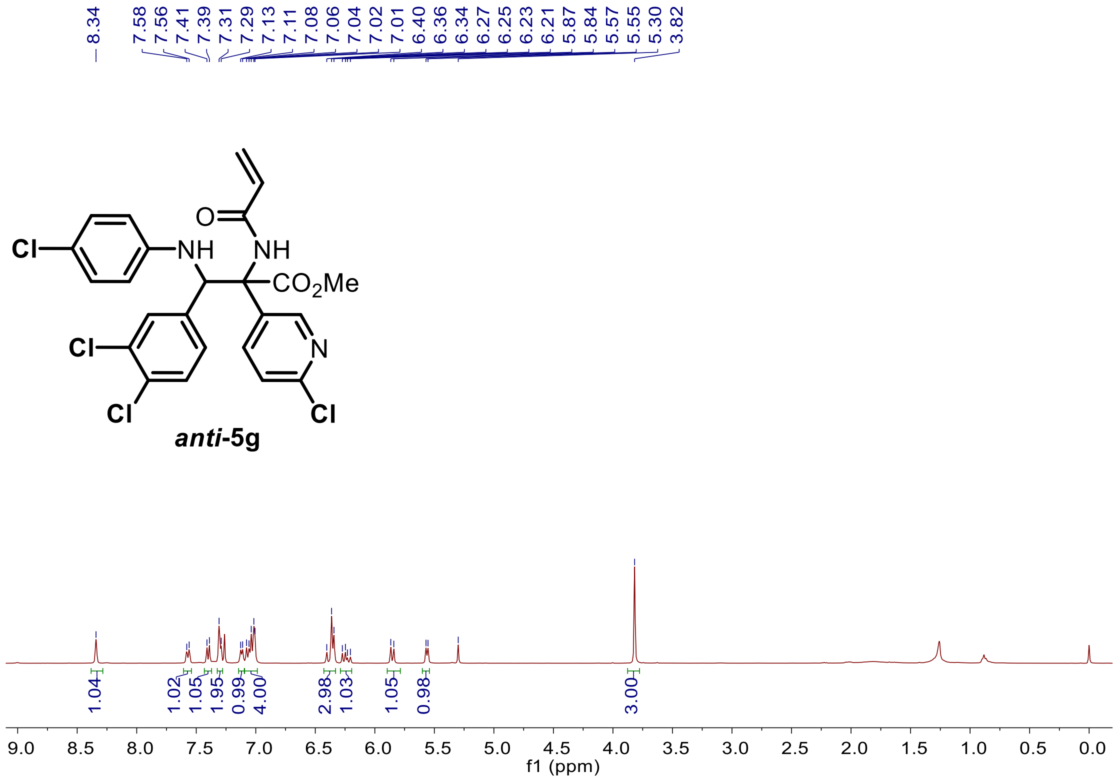

^{1}H NMR Spectrum of Compound **5g** (400MHz, $CDCl_3$)

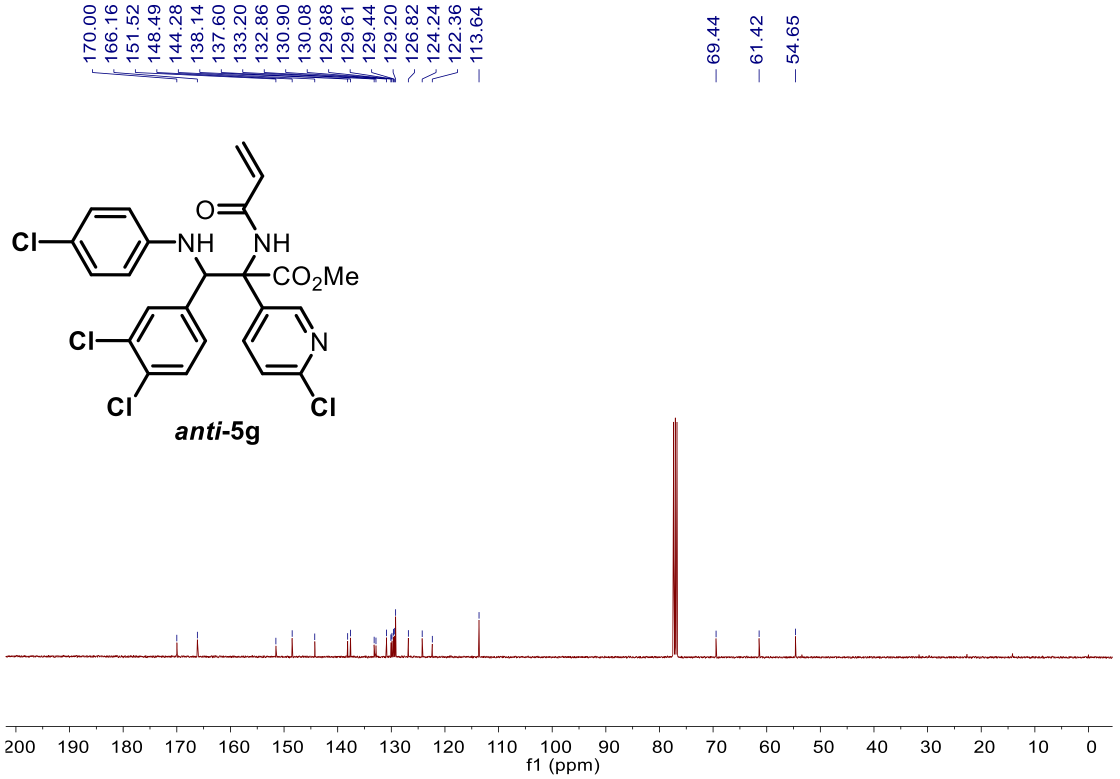

^{13}C NMR Spectrum of Compound **5g** (101MHz, $CDCl_3$)

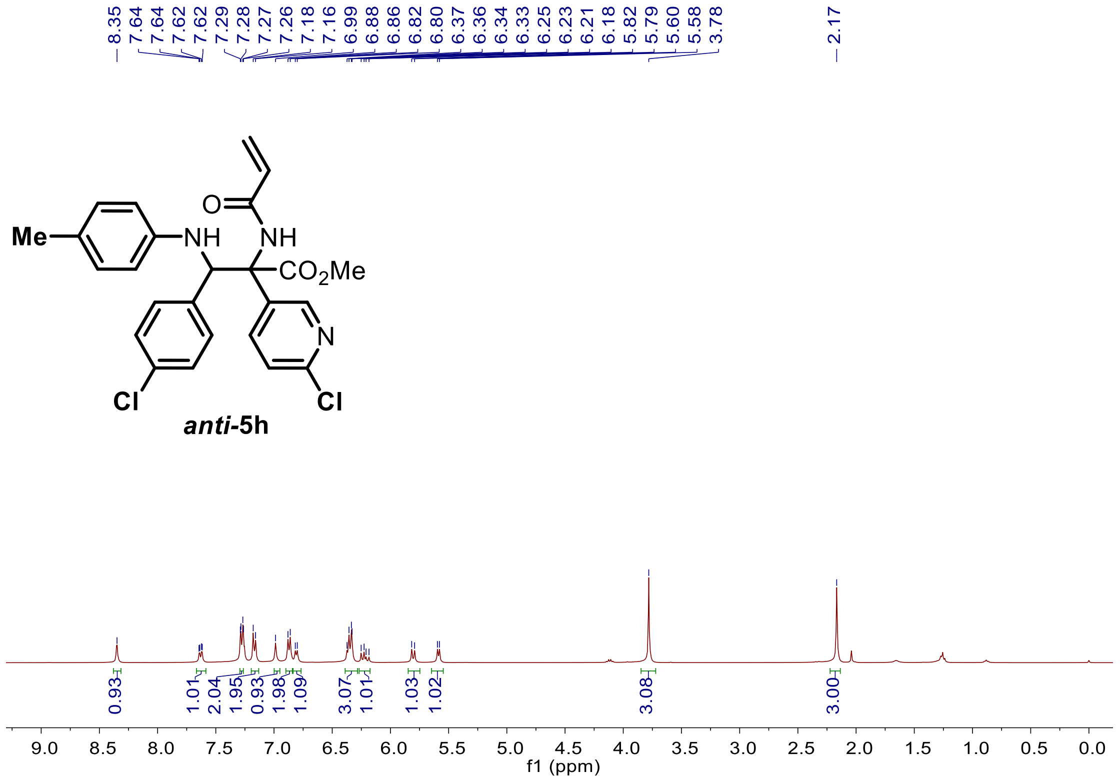

^{1}H NMR Spectrum of Compound **5h** (400MHz, $CDCl_3$)

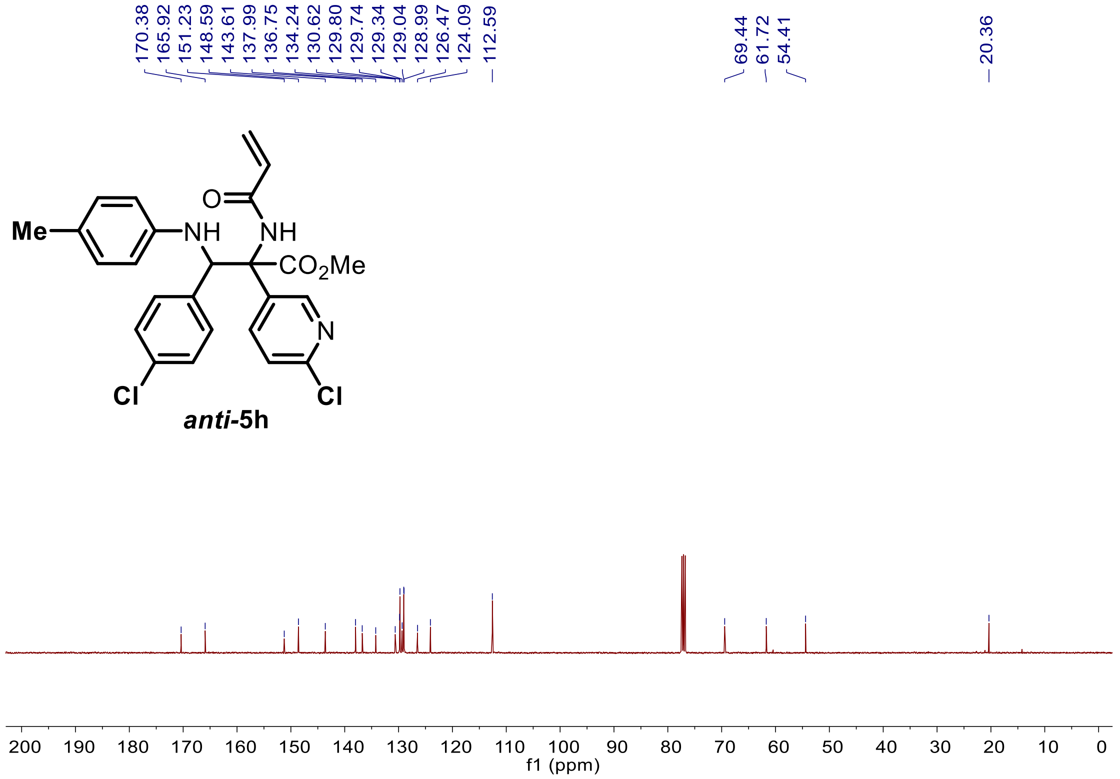

^{13}C NMR Spectrum of Compound **5h** (101MHz, $CDCl_3$)

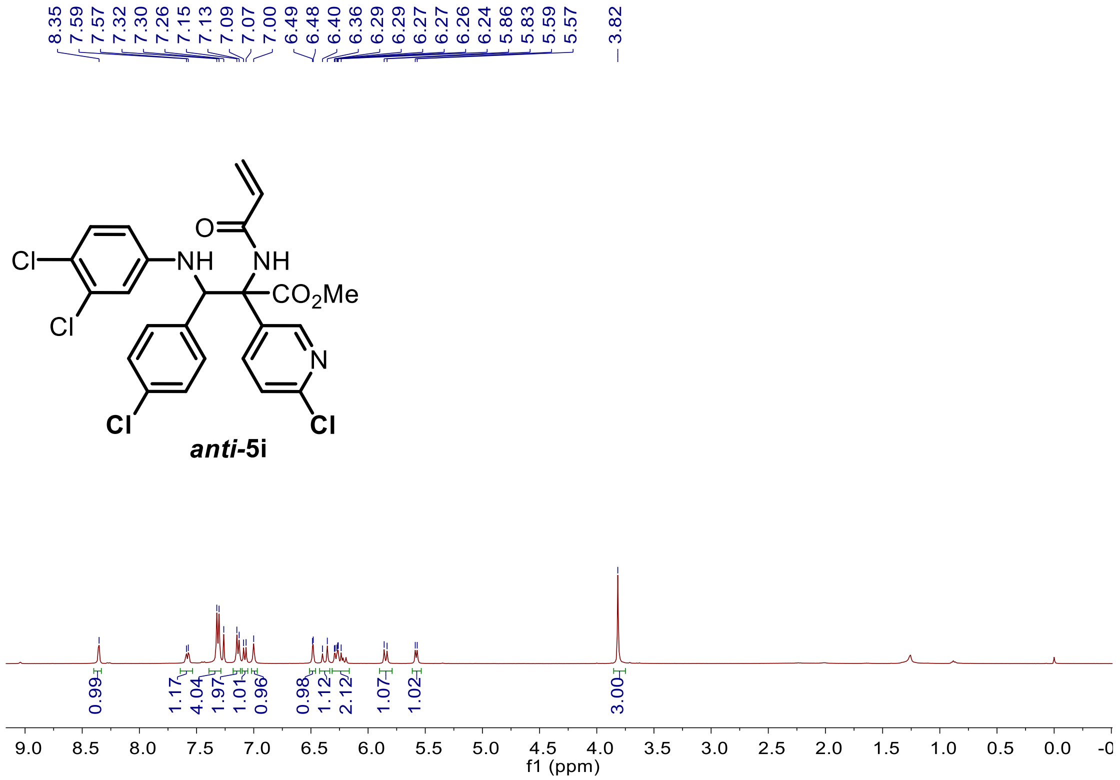

^{1}H NMR Spectrum of Compound **5i** (400MHz, $CDCl_3$)

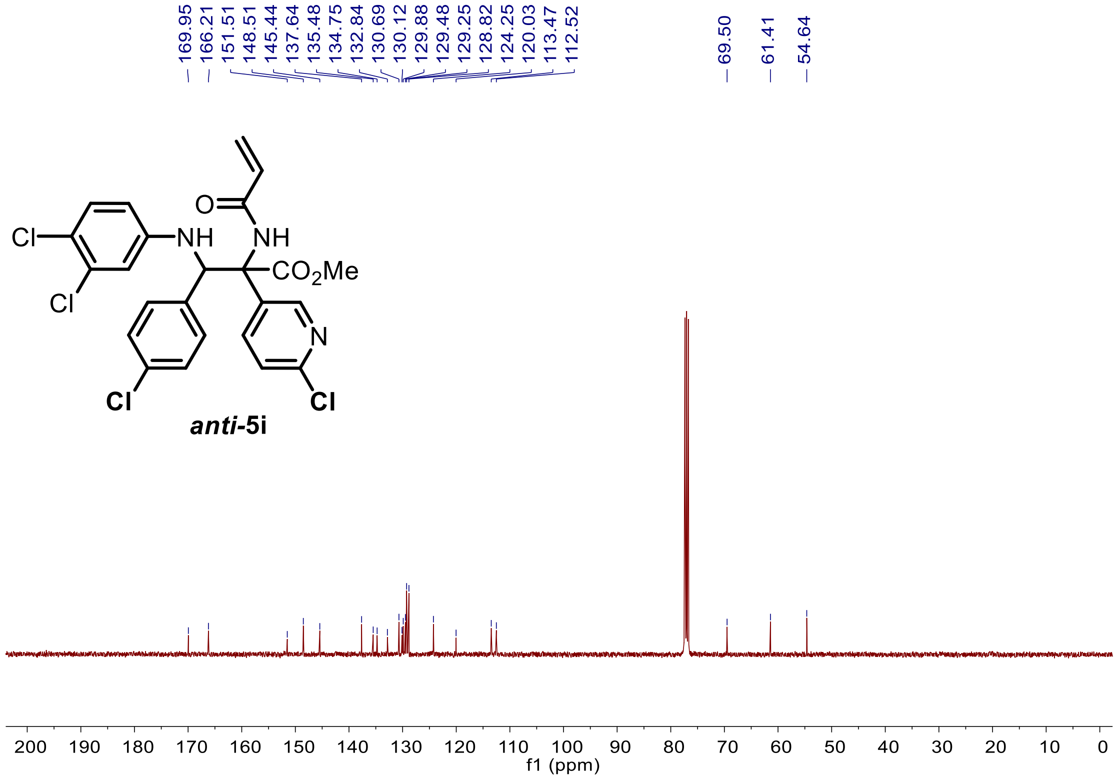

^{13}C NMR Spectrum of Compound **5i** (101MHz, $CDCl_3$)

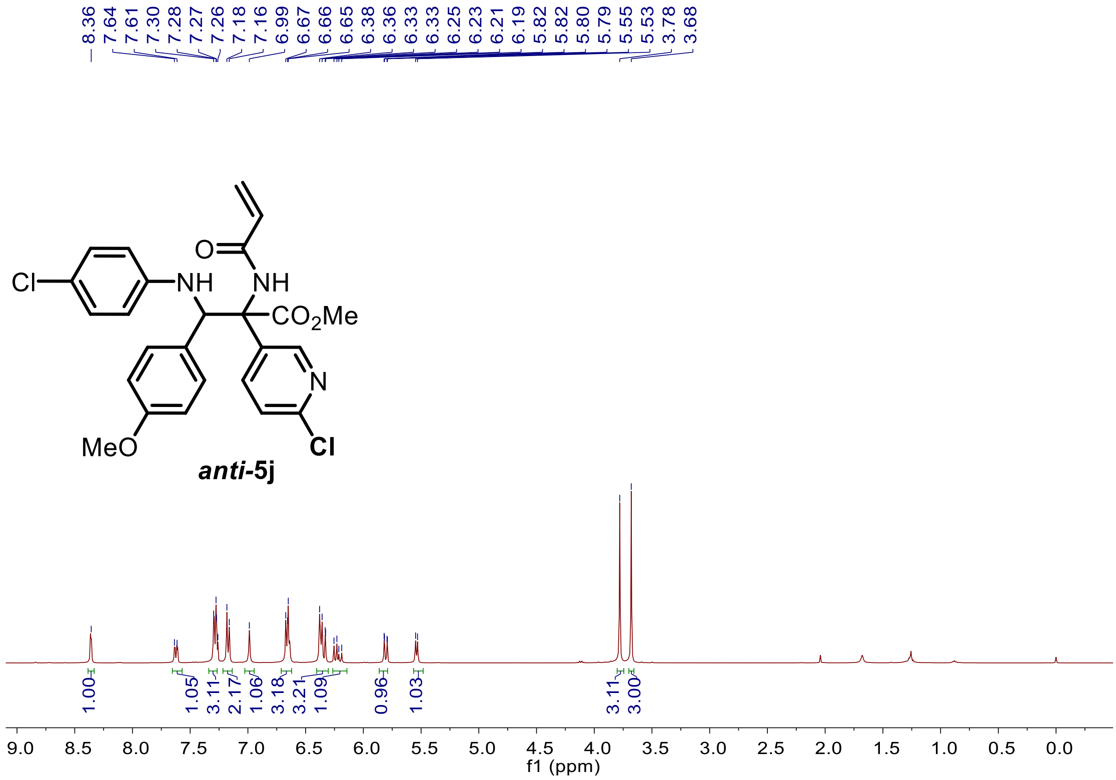

^{1}H NMR Spectrum of Compound **5j** (400MHz, $CDCl_3$)

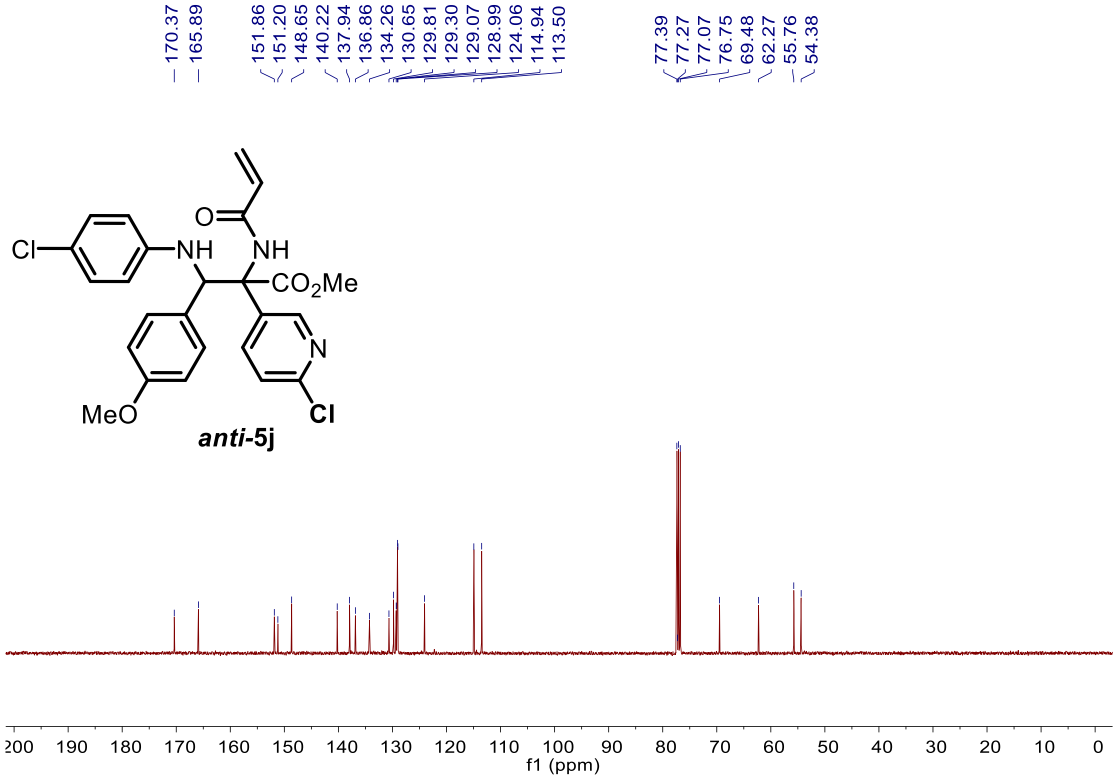

^{13}C NMR Spectrum of Compound **5j** (101MHz, $CDCl_3$)

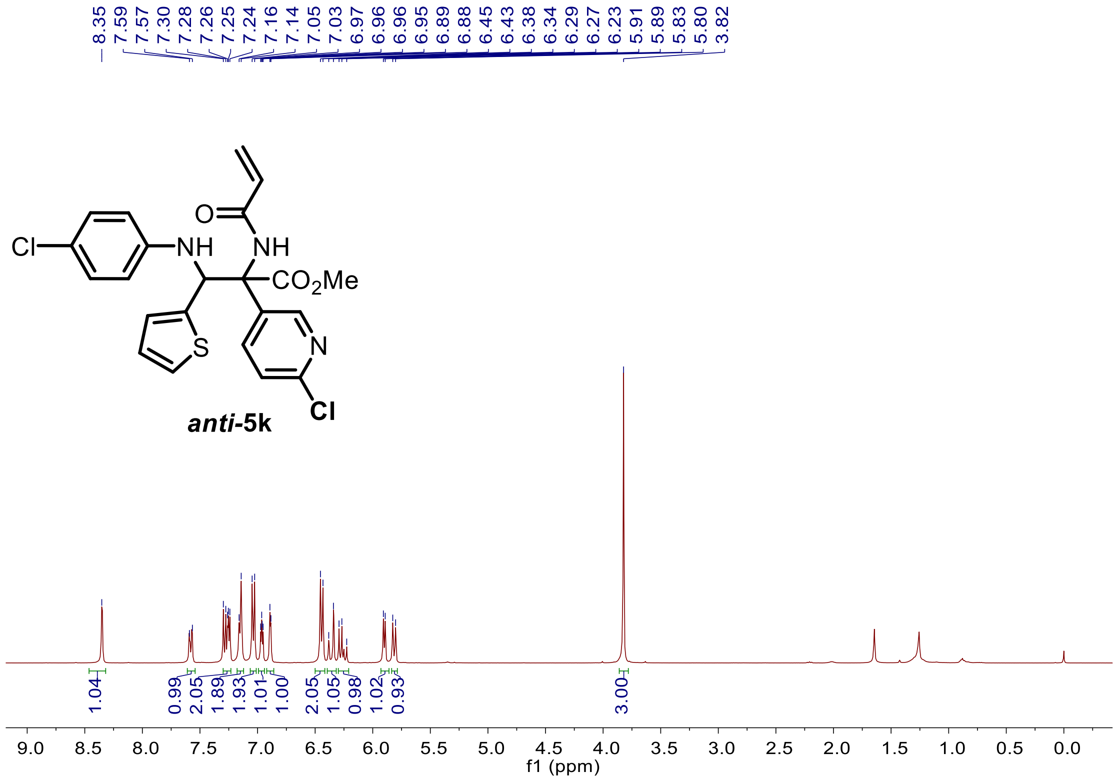

^{1}H NMR Spectrum of Compound **5k** (400MHz, $CDCl_3$)

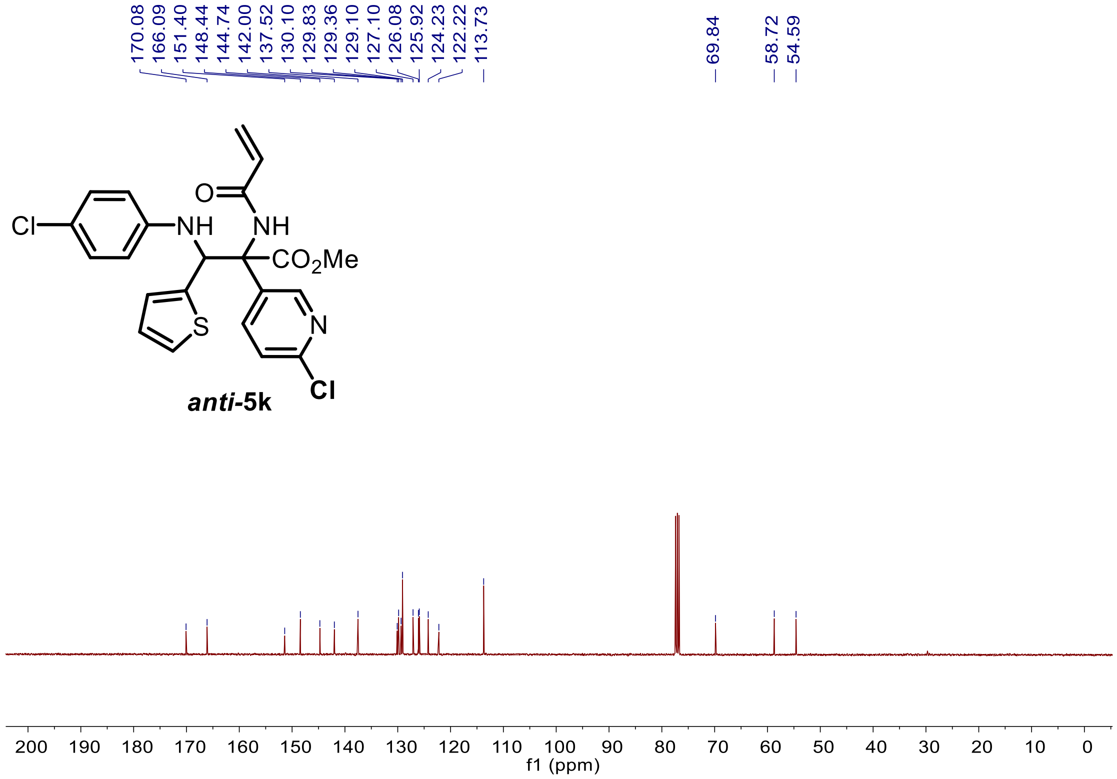

^{13}C NMR Spectrum of Compound **5k** (101MHz, $CDCl_3$)

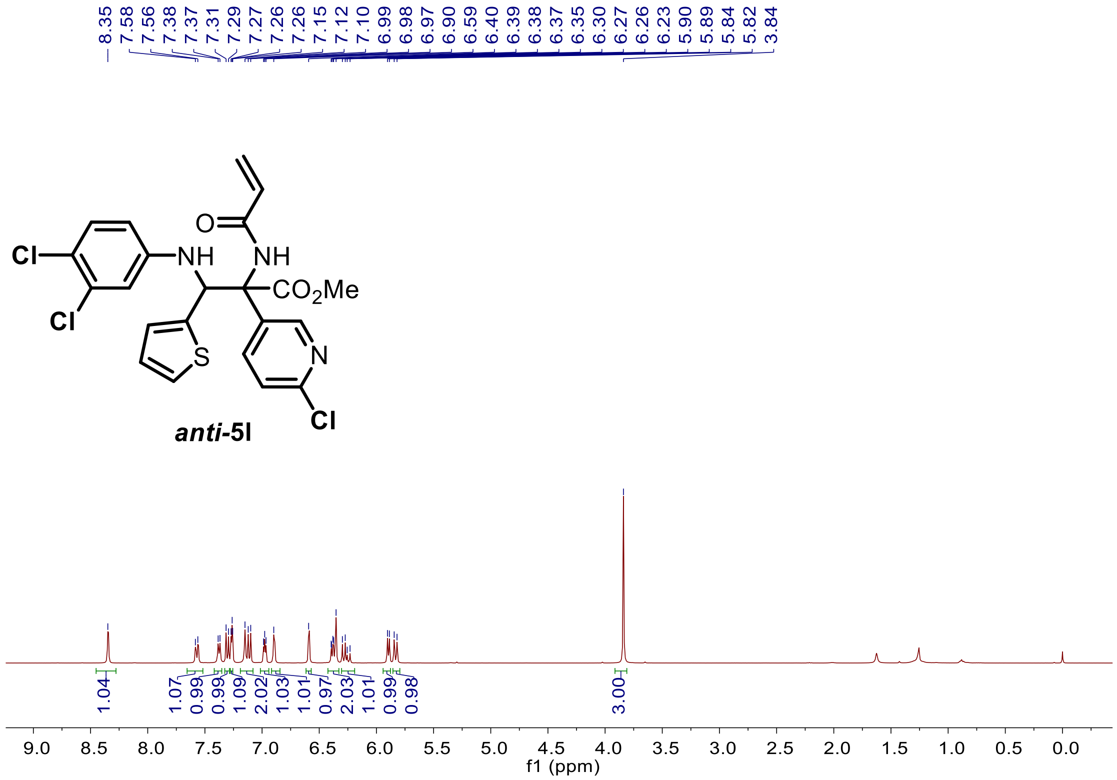

^{1}H NMR Spectrum of Compound **5l** (400MHz, $CDCl_3$)

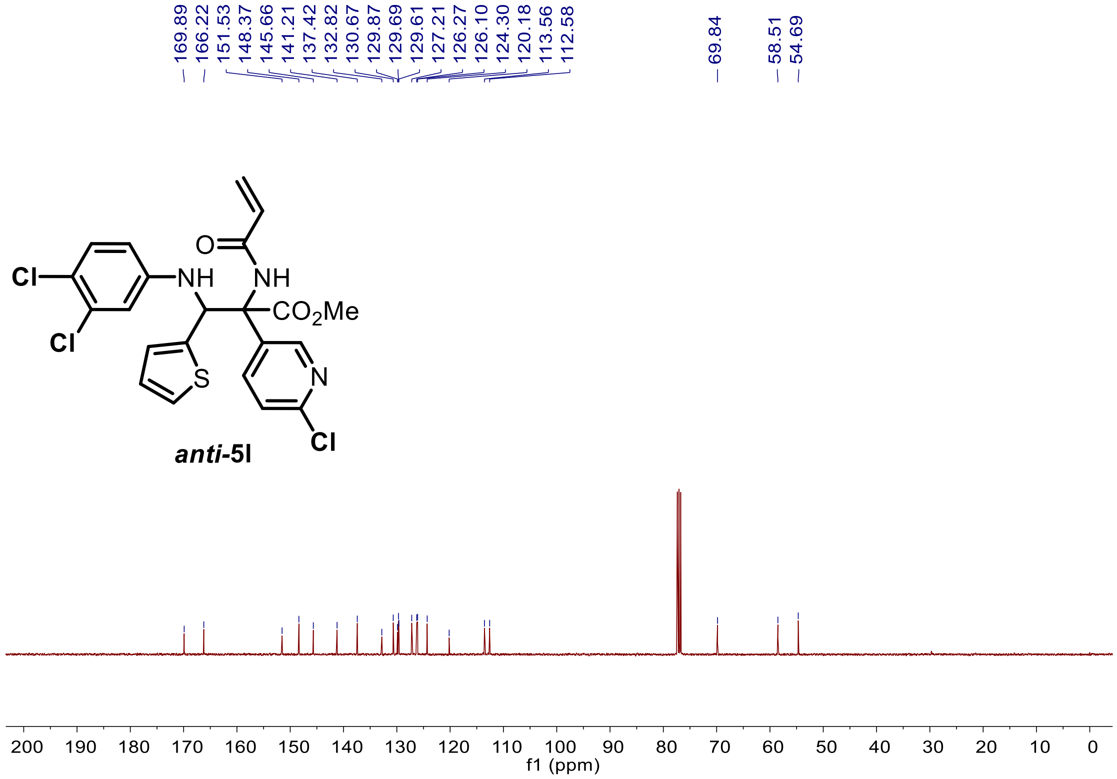

^{13}C NMR Spectrum of Compound **5l** (101MHz, $CDCl_3$)

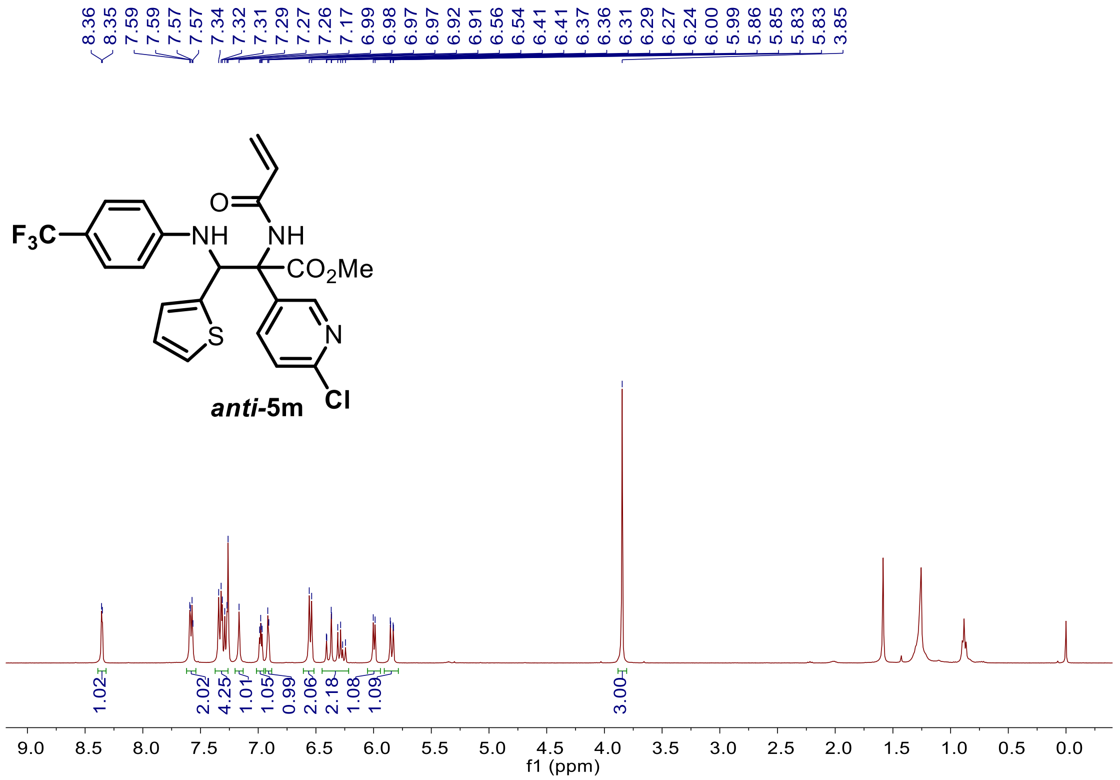

^{1}H NMR Spectrum of Compound **5m** (400MHz, $CDCl_3$)

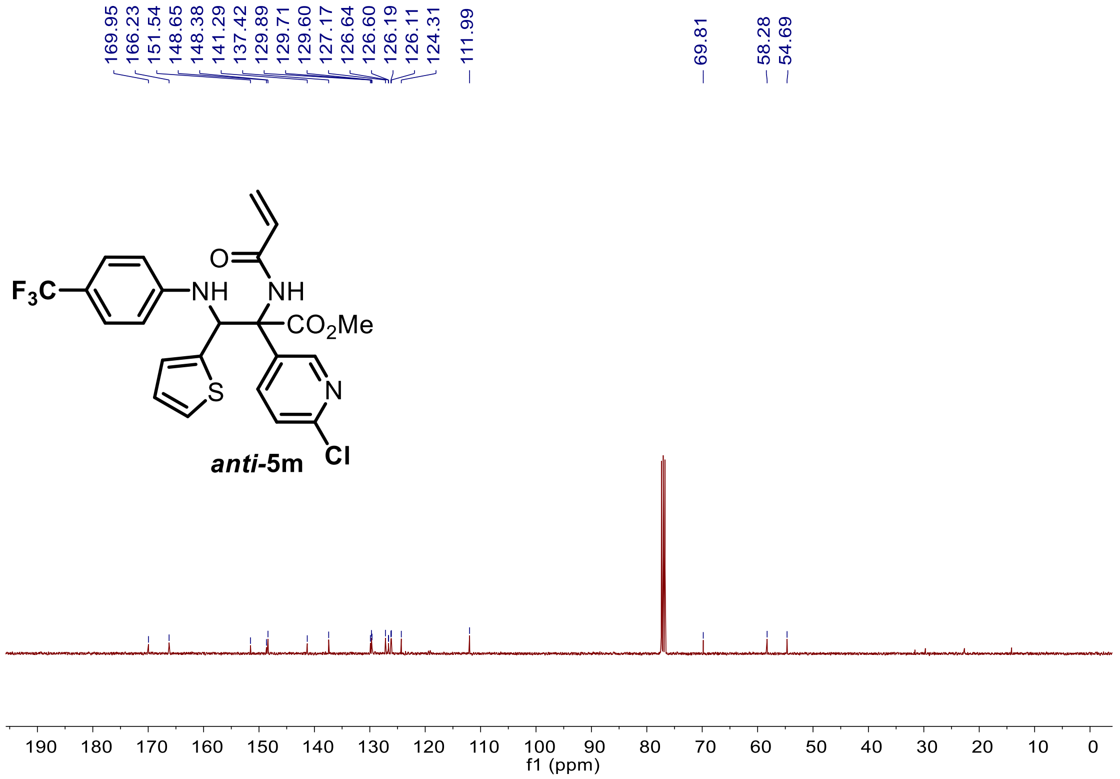

^{13}C NMR Spectrum of Compound **5m** (101MHz, $CDCl_3$)

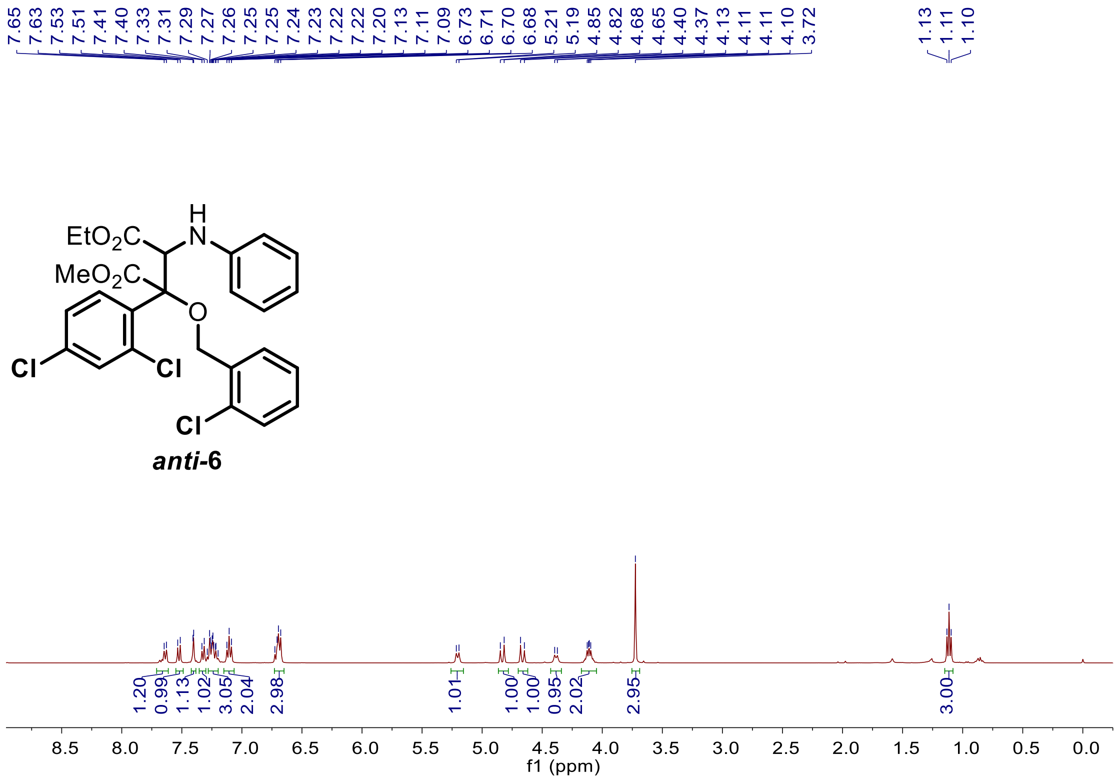

^{1}H NMR Spectrum of Compound **6** (400MHz, $CDCl_3$)

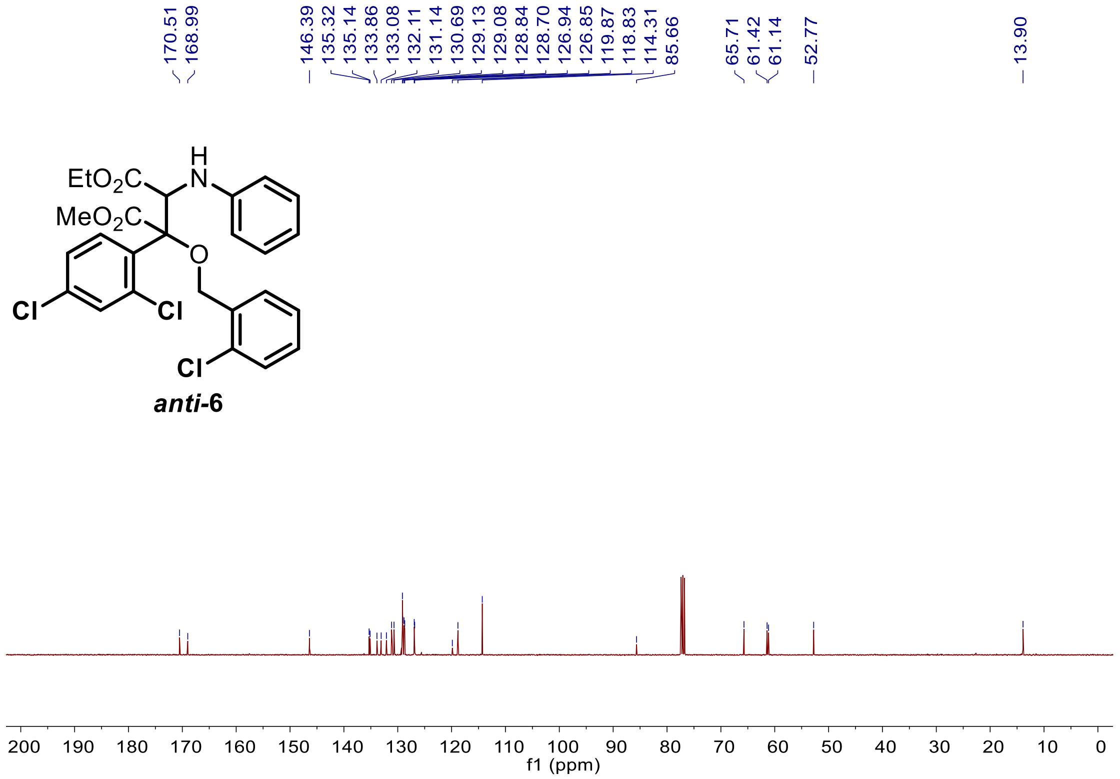

^{13}C NMR Spectrum of Compound **6** (101MHz, $CDCl_3$)

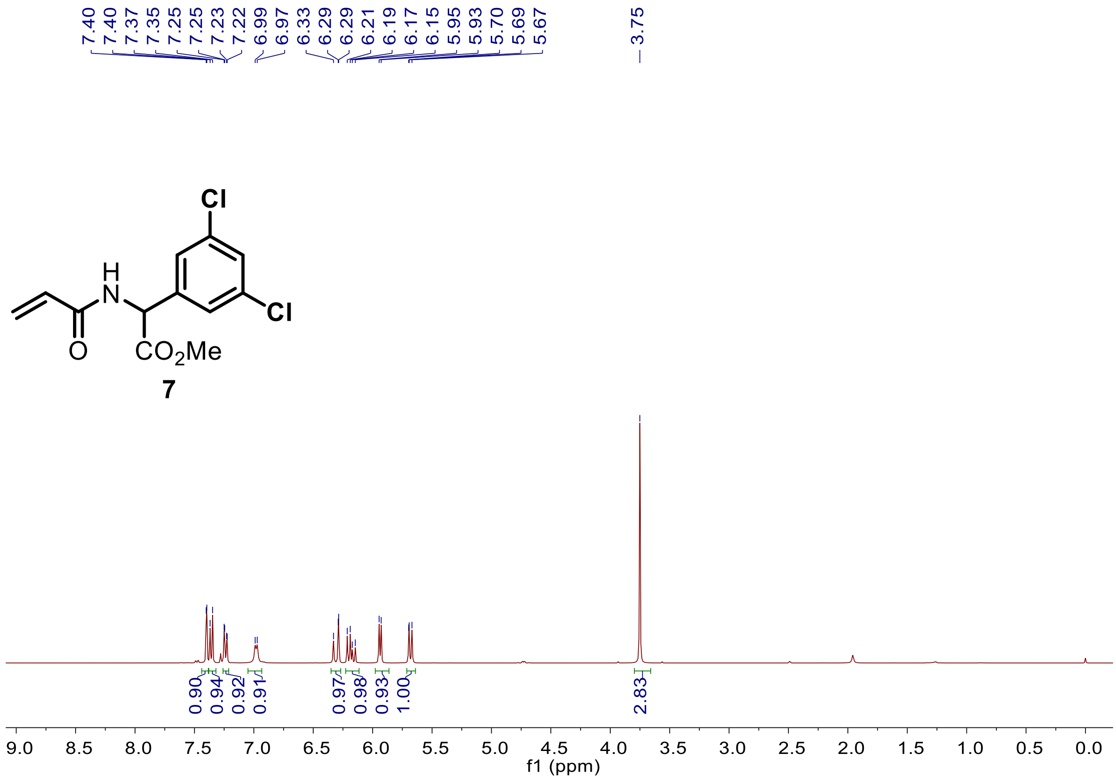

^{1}H NMR Spectrum of Compound **7** (400MHz, $CDCl_3$)

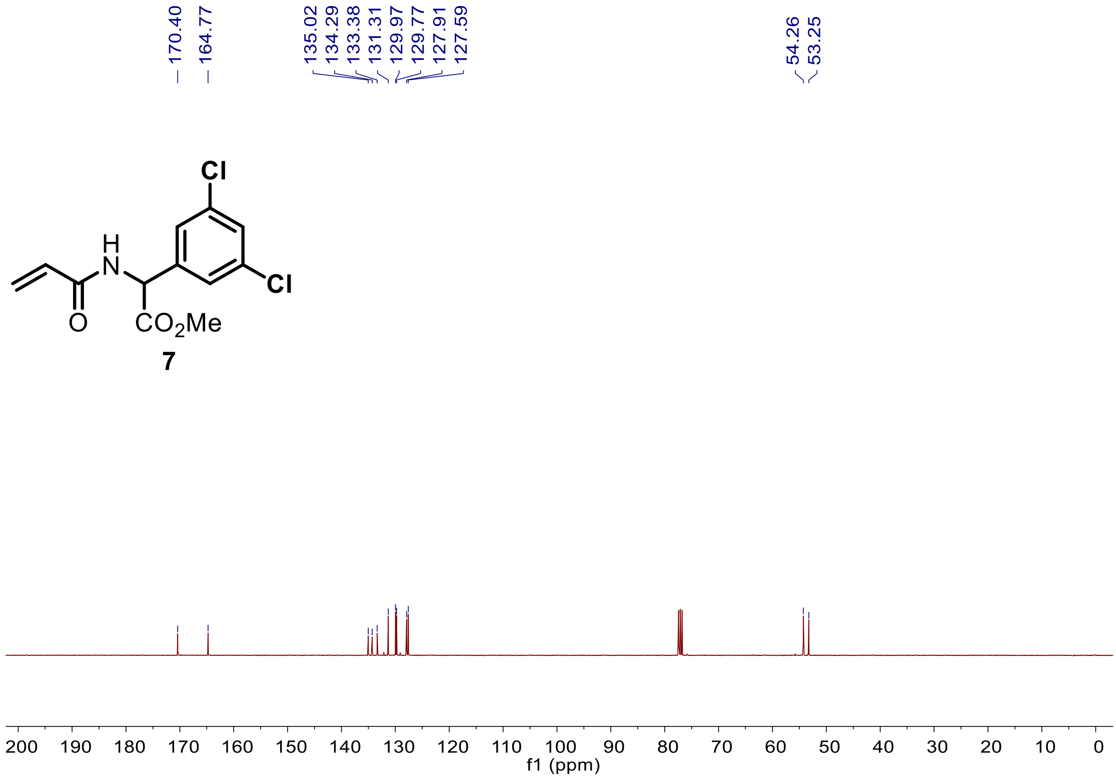

^{13}C NMR Spectrum of Compound **7** (101MHz, $CDCl_3$)
